# Supplementary material for: Endoparasitoid lifestyle promotes endogenization and domestication of dsDNA viruses
Source: eLife. 2023 Jun 6;12:e85993. doi: 10.7554/eLife.85993 (PMC10259472; doi:10.7554/eLife.85993)

Cluster9478 − families involved : ['Tombusviridae'] − putative protein : ['integral component of membrane [GO:0016021]', 'Uncharacterized protein']

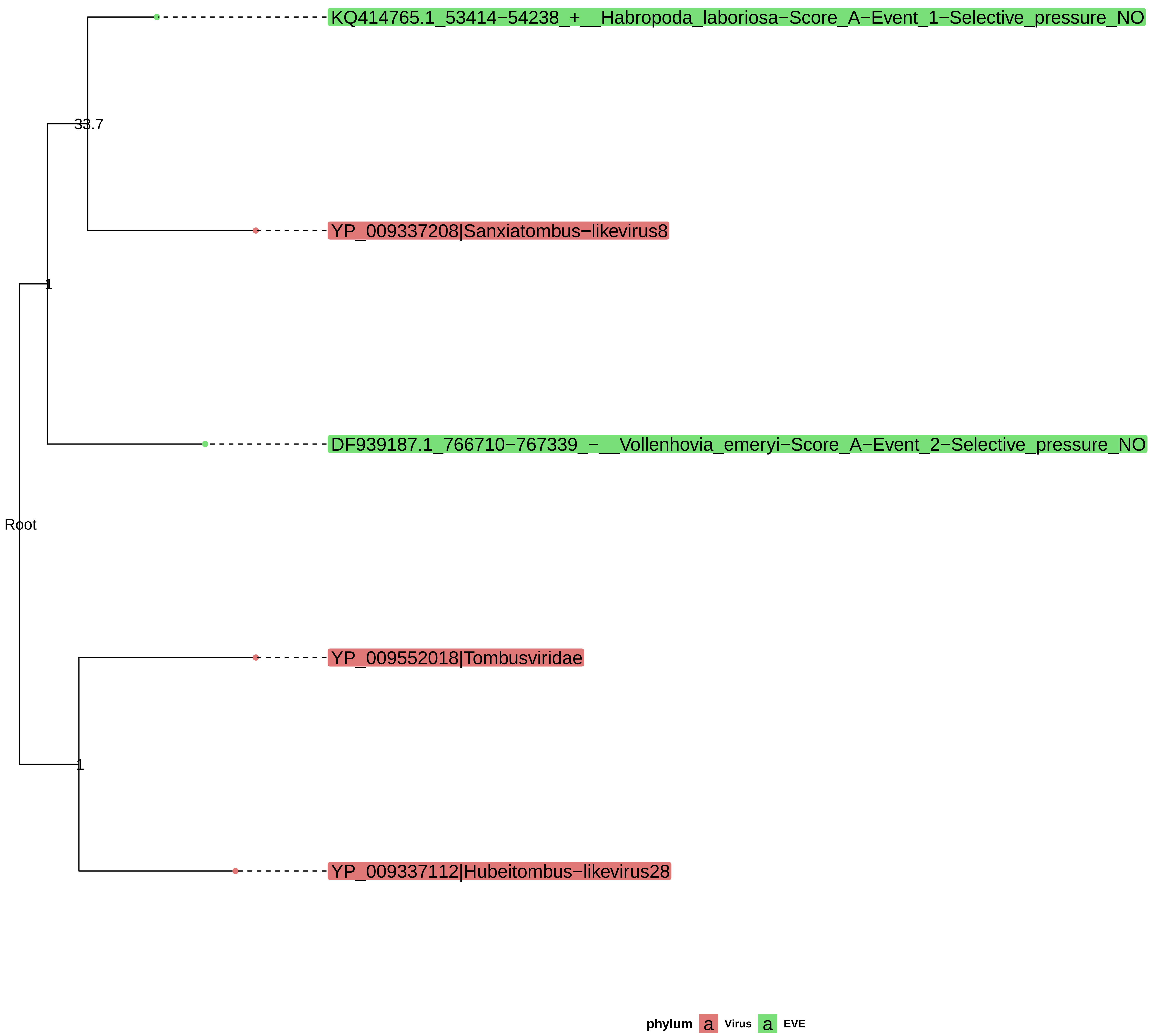

Cluster7388 − families involved : ['Asfarviridae', 'Poxviridae'] − putative protein : ['MyD116 homolog (Protein DP71L)']

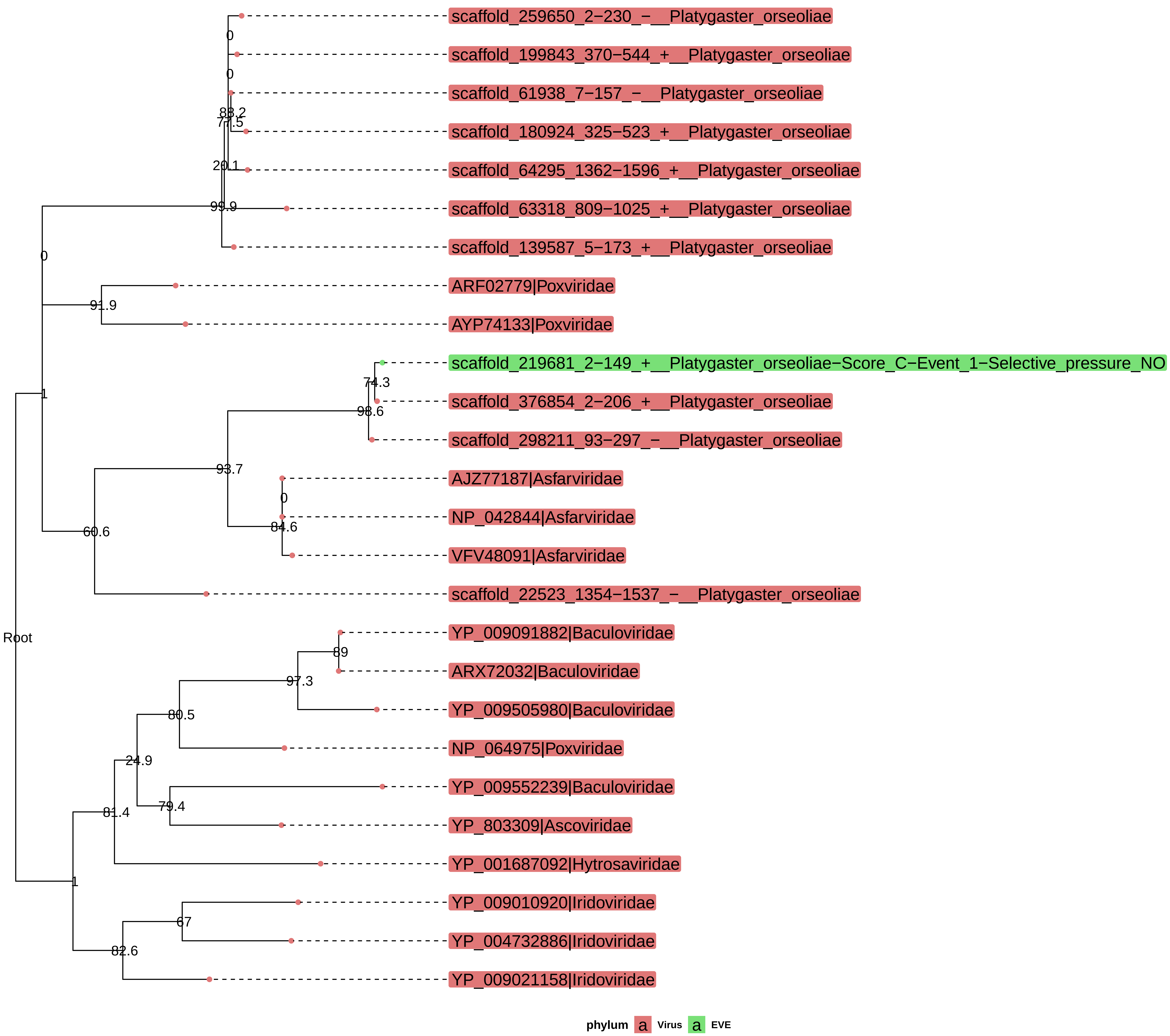

Cluster5650 − families involved : ['Baculoviridae'] − putative protein : ['Vef']

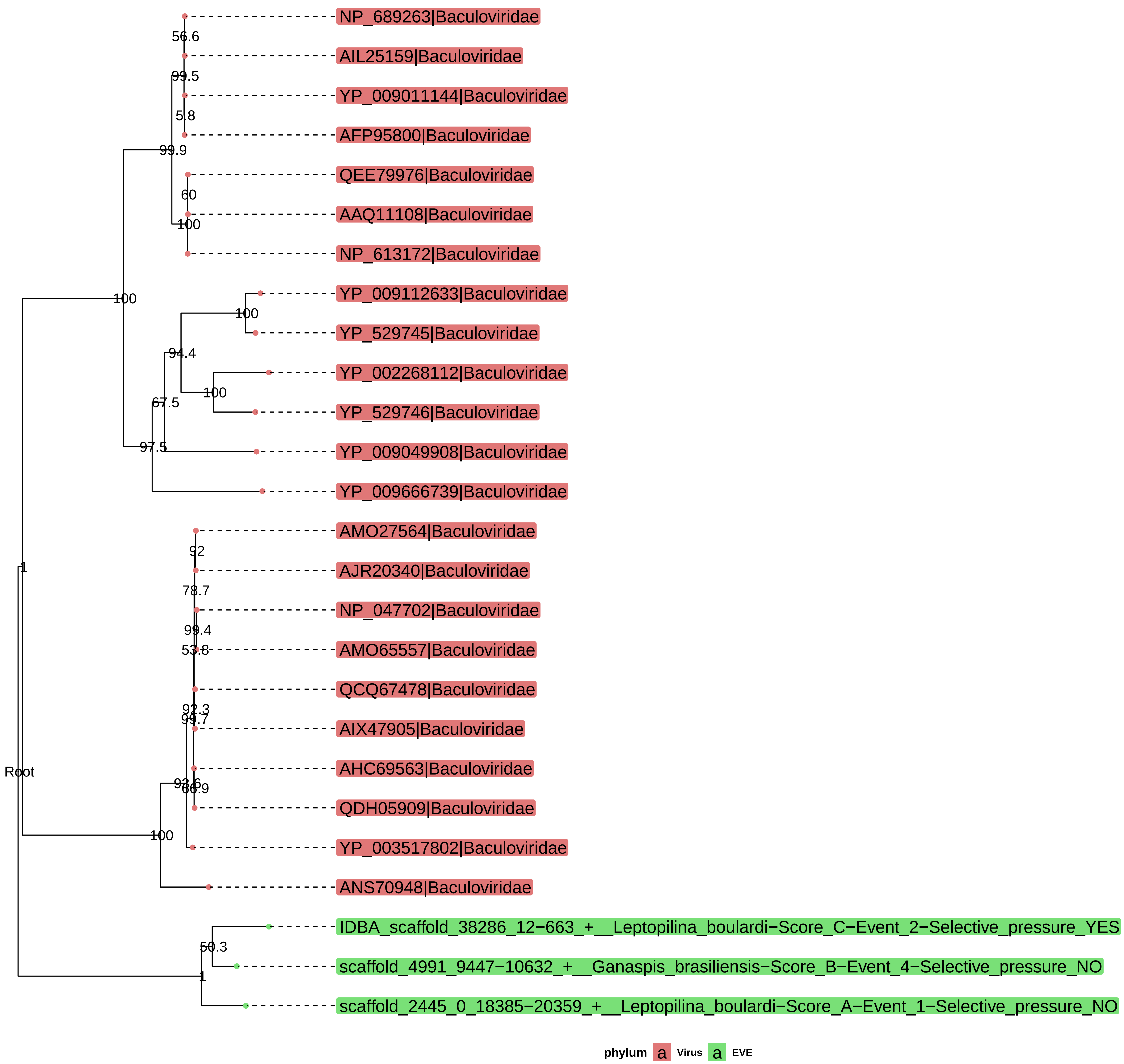

Cluster5250 − families involved : ['Phycodnaviridae'] − putative protein : ['Uncharacterized protein c156L']

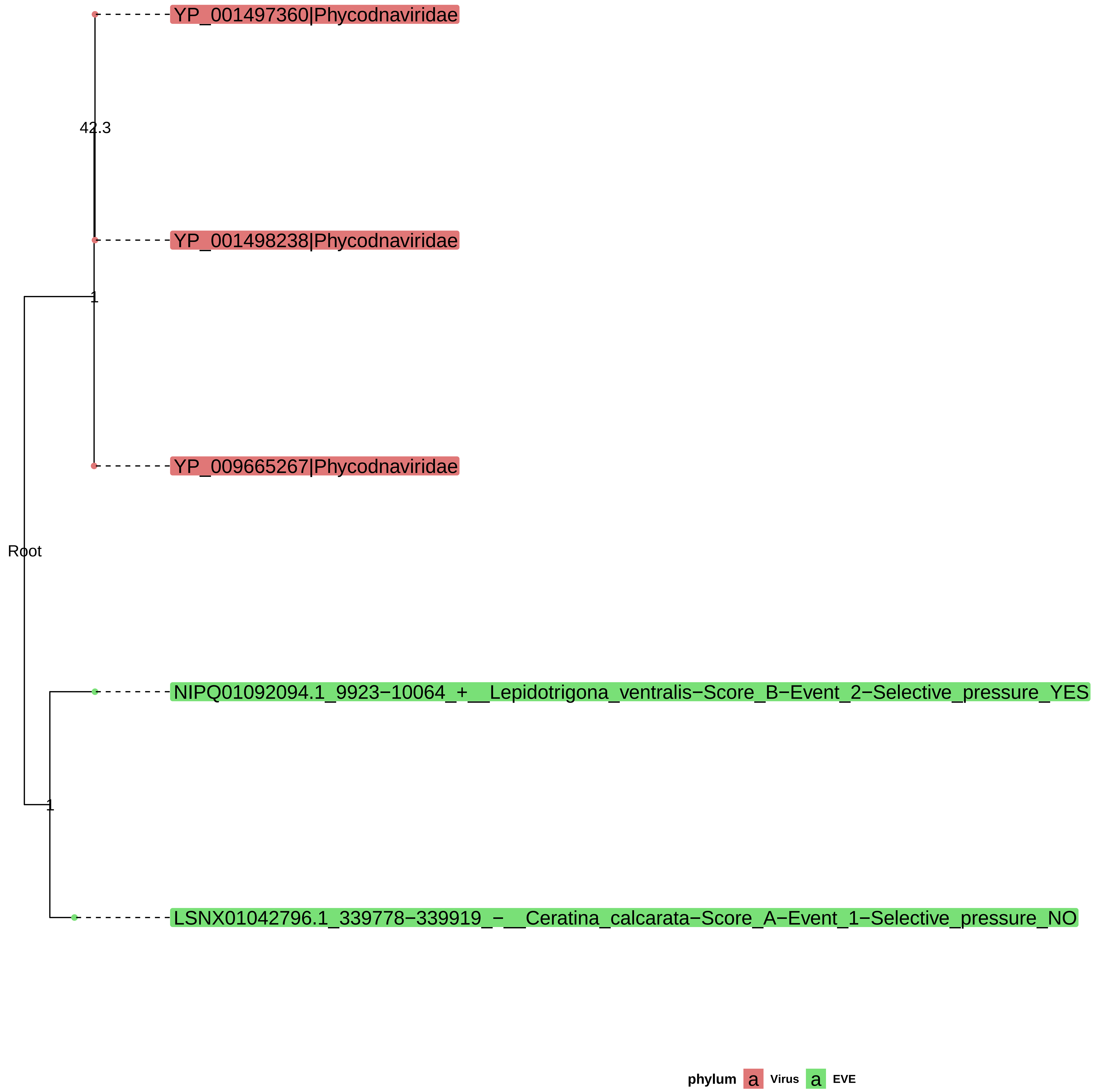

Cluster4884 − families involved : ['Phasmaviridae'] − putative protein : ['Glycoprotein']

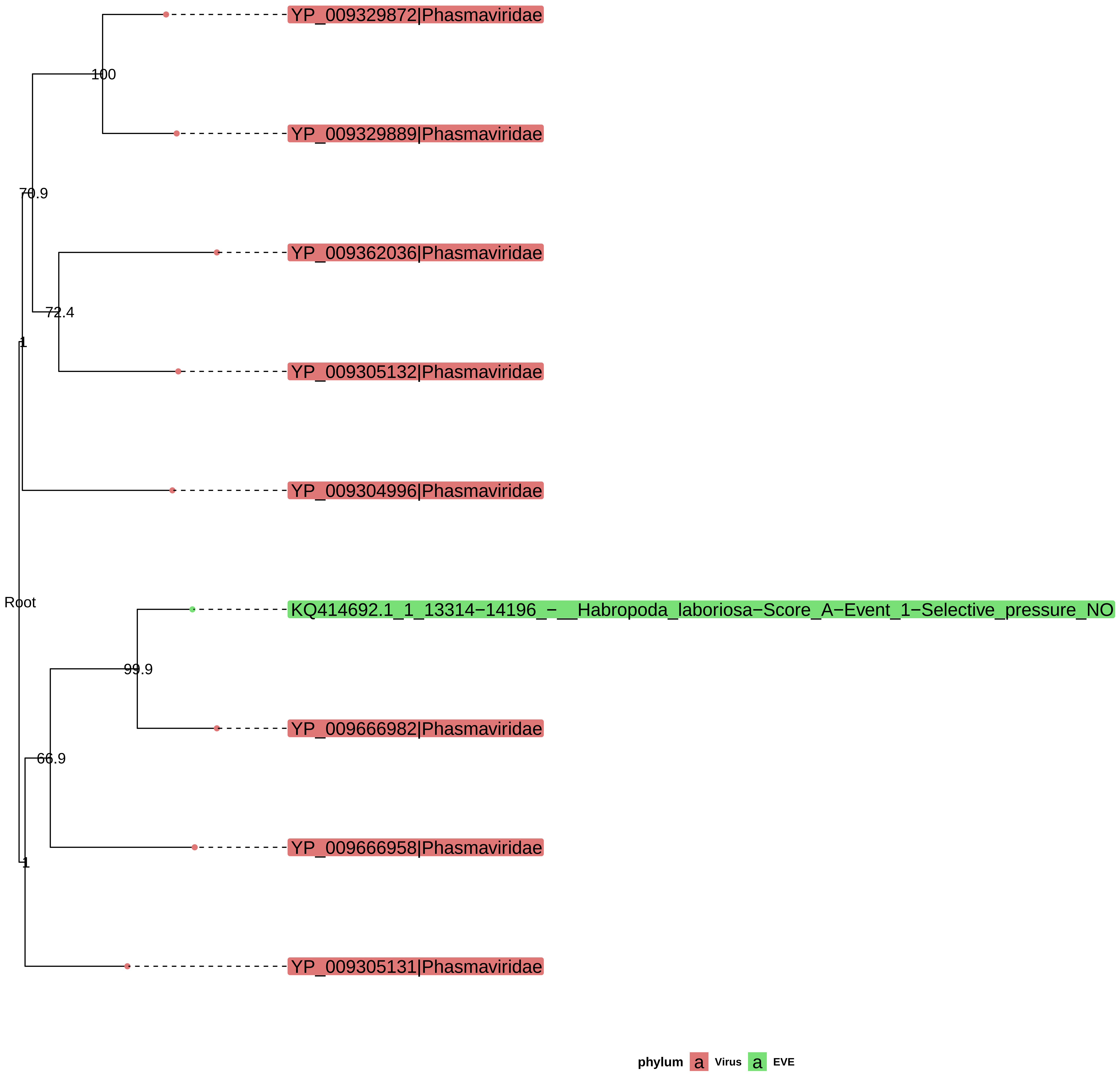

Cluster4292 − families involved : ['Mono−Chu'] − putative protein : ['Matrix protein']

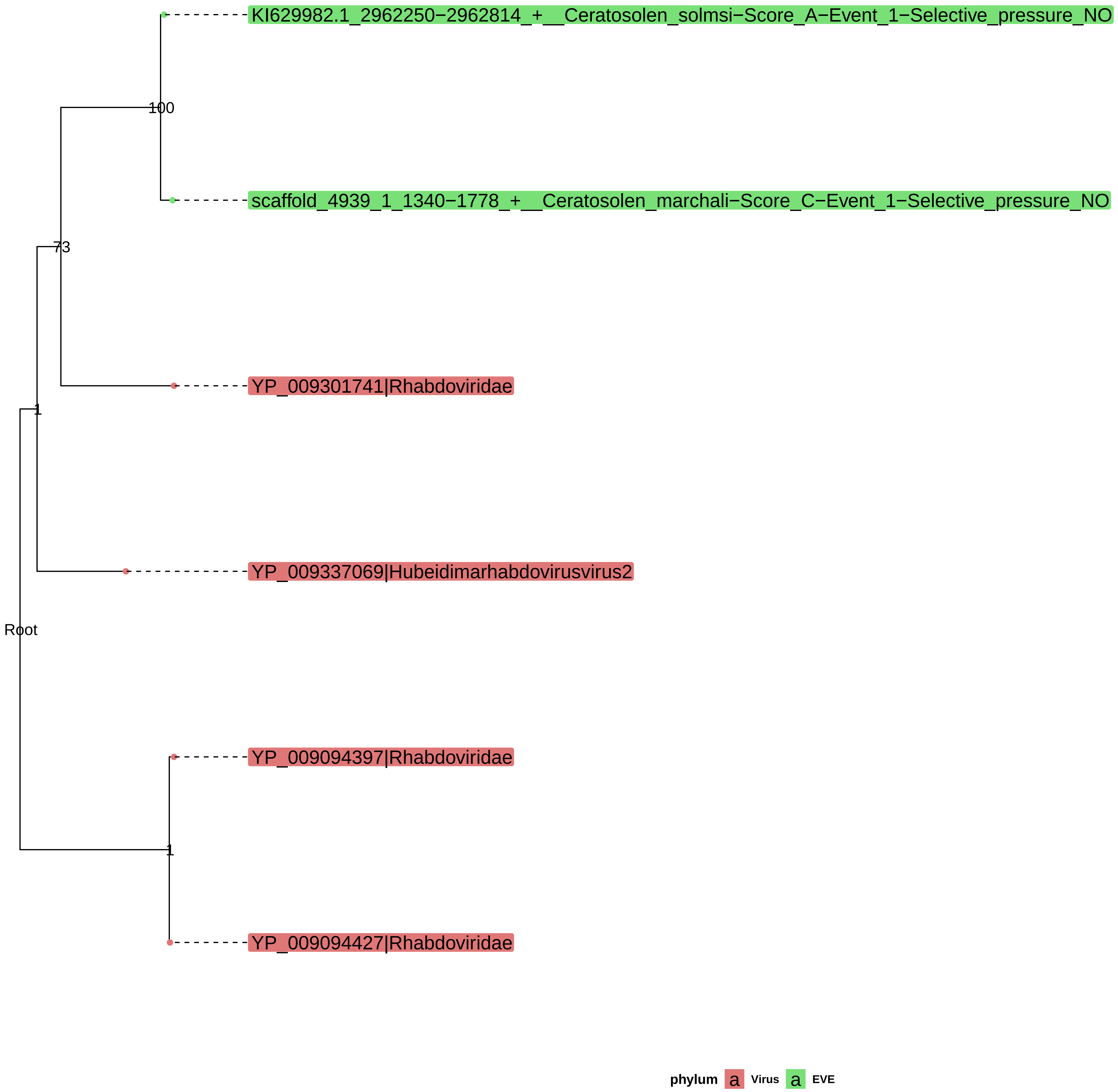

Cluster4230 − families involved : ['Unknown'] − putative protein : ['Family of unknown function (DUF5510)']

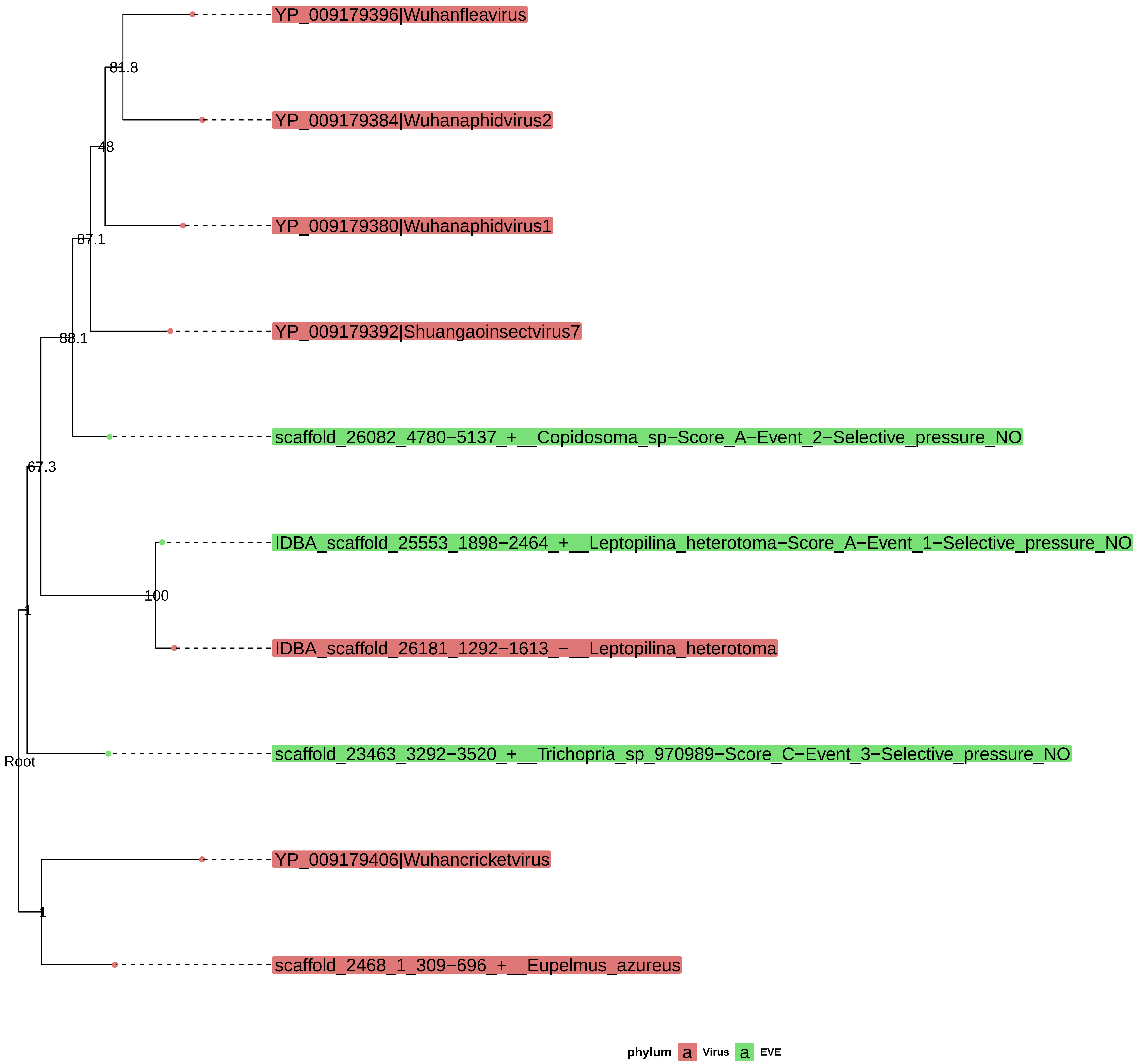

Cluster3516 − families involved : ['Parvoviridae'] − putative protein : ['Non−structural protein 2']

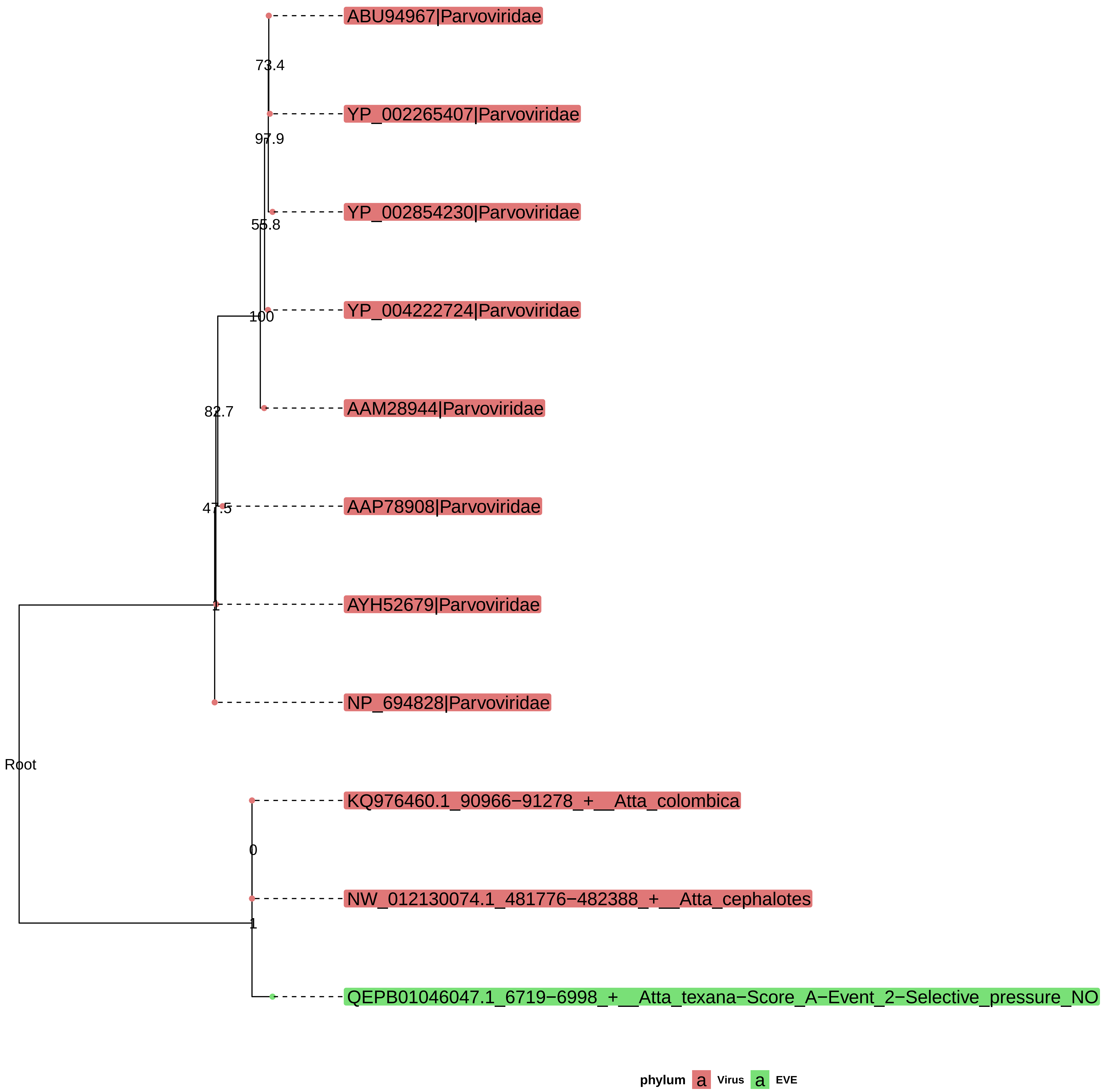

Cluster6002 − families involved : ['Baculoviridae'] − putative protein : ['Methyltransferase/RNA helicase (EC 3.6.4.13) (Replicase large subunit) (Replicase small subunit)']

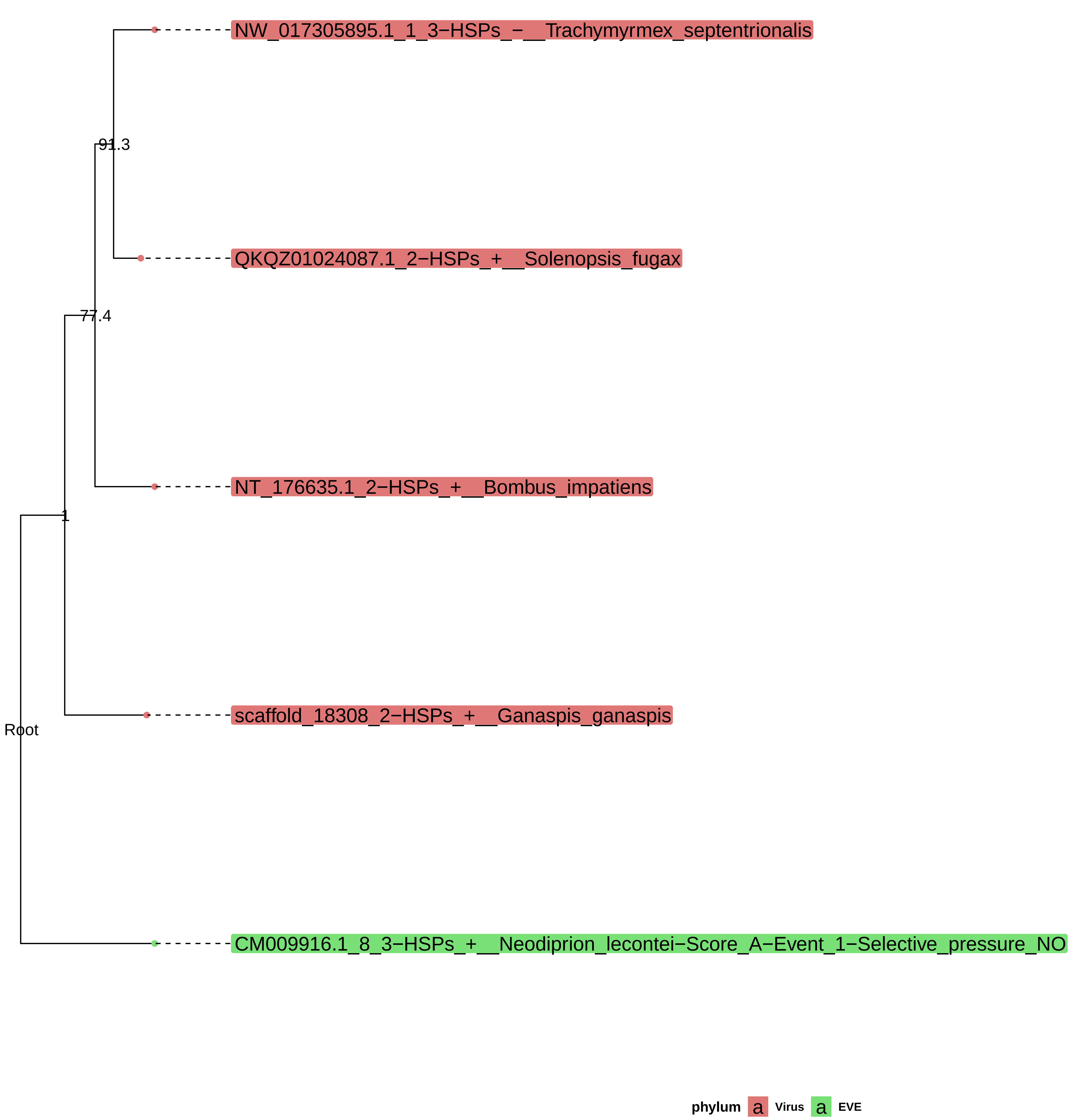

Cluster3240 − families involved : ['Baculoviridae'] − putative protein : ['Uncharacterized protein']

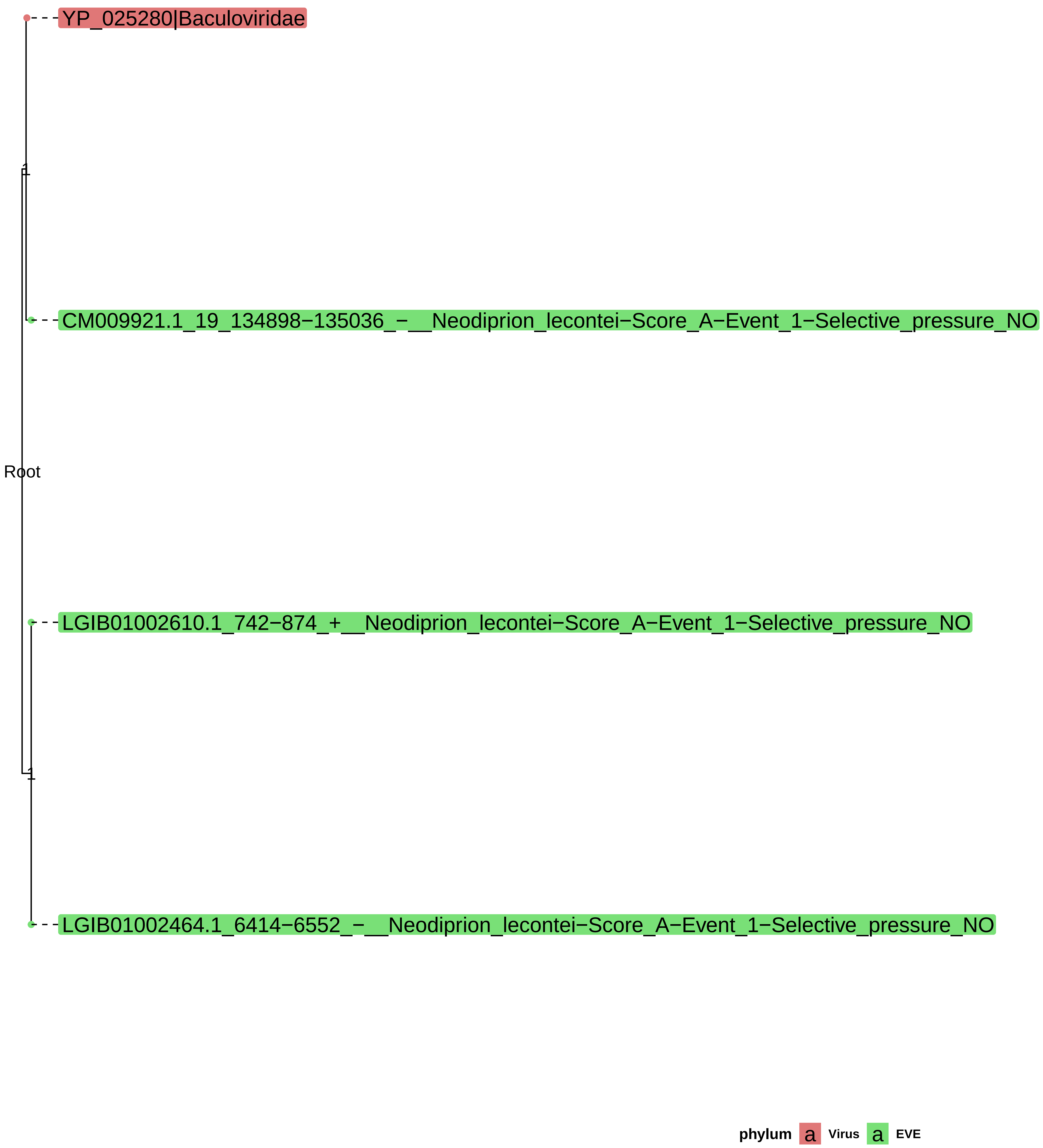

Cluster3102 − families involved : ['Rhabdoviridae'] − putative protein : ['Glycoprotein']

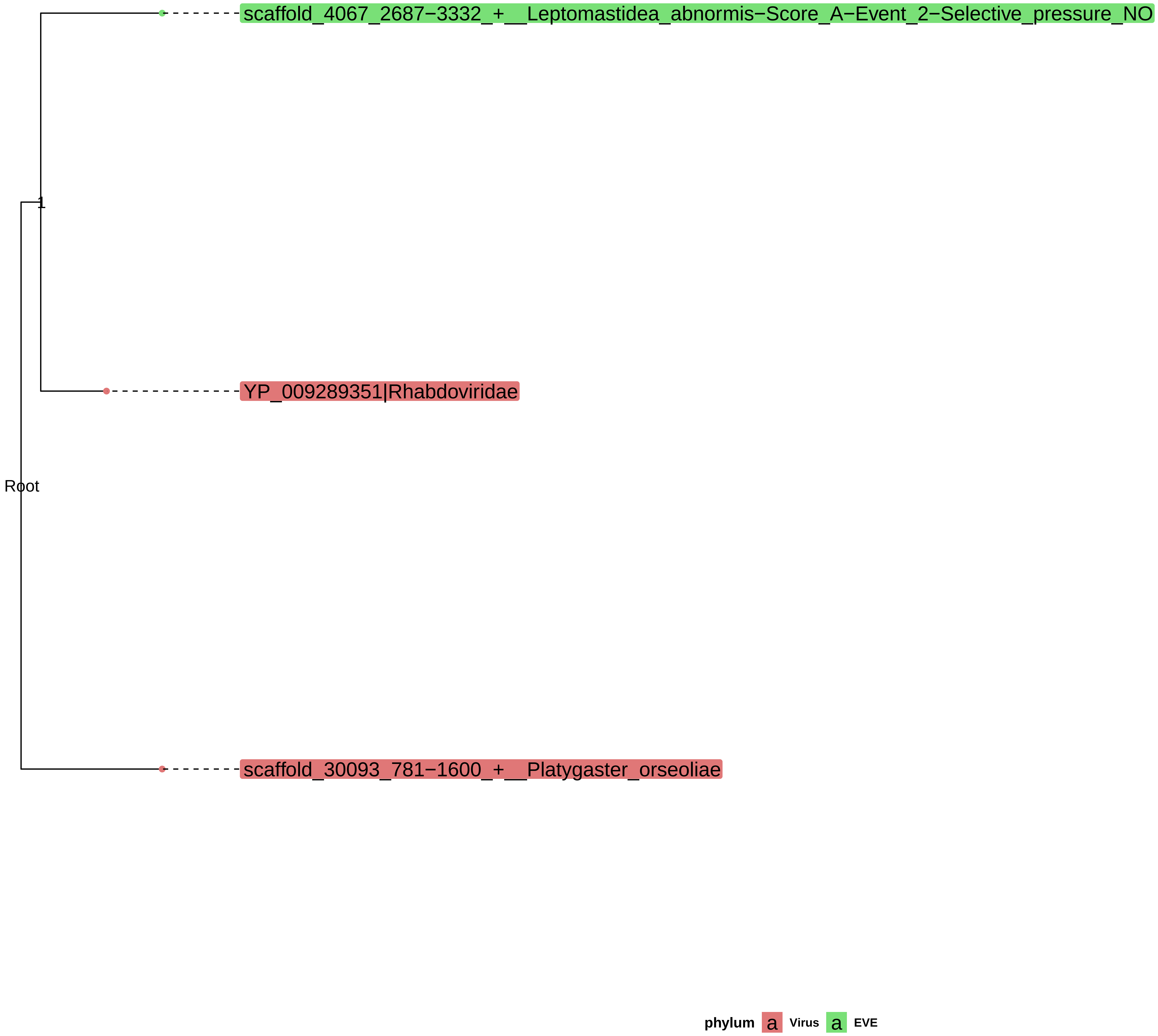

Cluster294 − families involved : ['Genomoviridae'] − putative protein : ['Capsid protein']

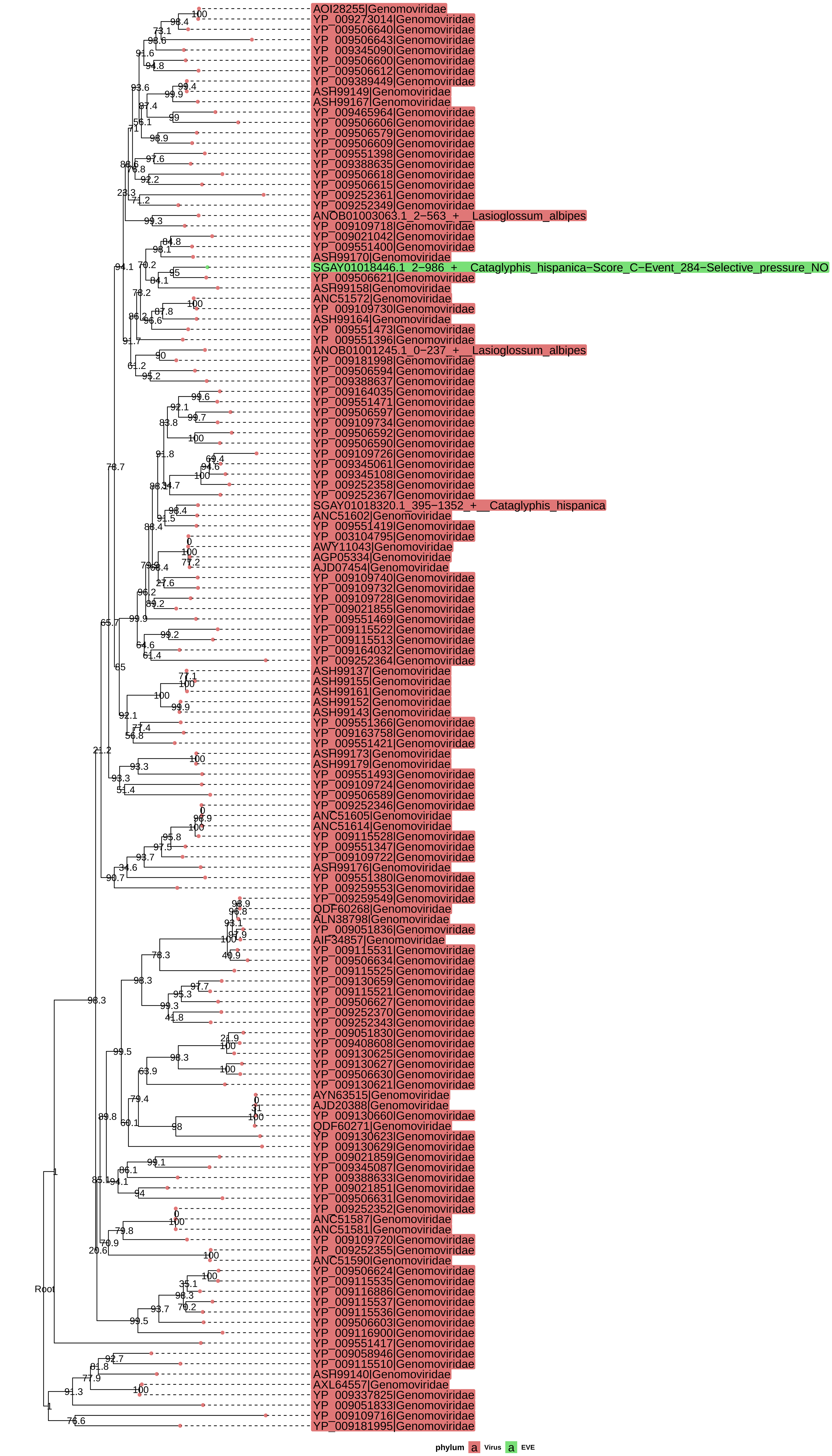

Cluster27505 − families involved : ['Partiti−Picobirna'] − putative protein : ['Uncharacterized protein']

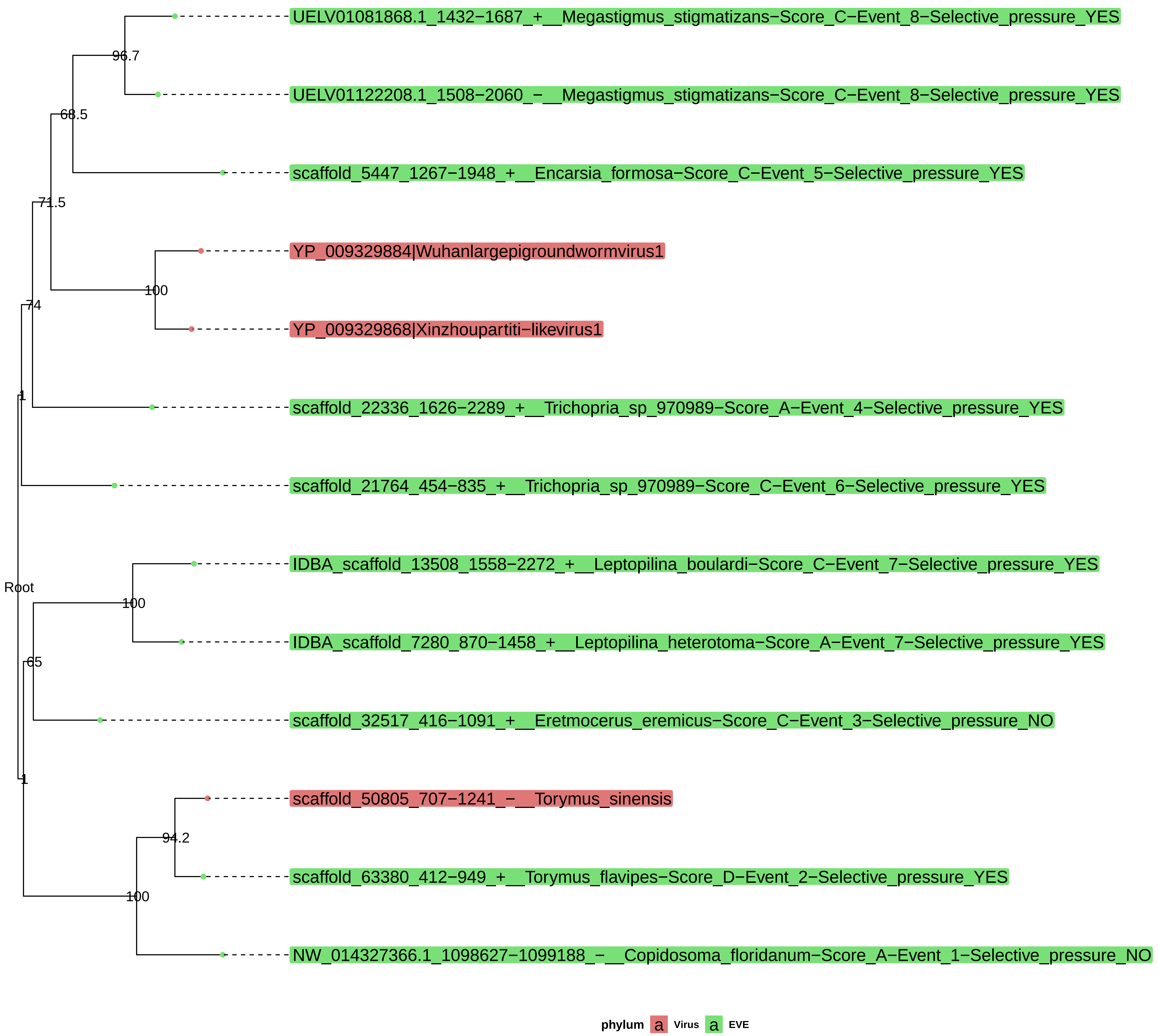

Cluster27410 − families involved : ['Iridoviridae'] − putative protein : ['Myristylated membrane protein']

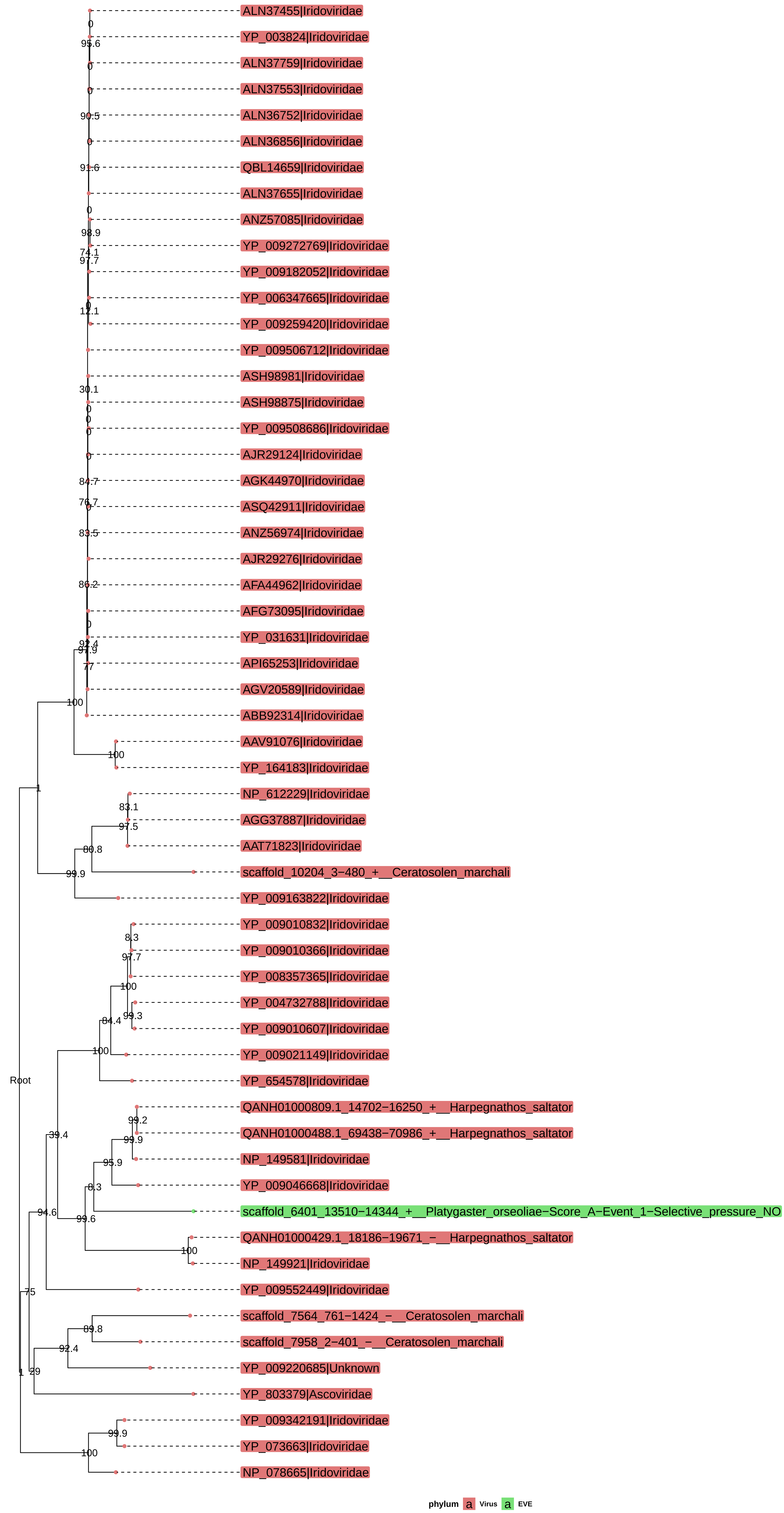

Cluster27285 − families involved : ['Luteo−Sobemo'] − putative protein : ['Family of unknown function (DUF5567)']

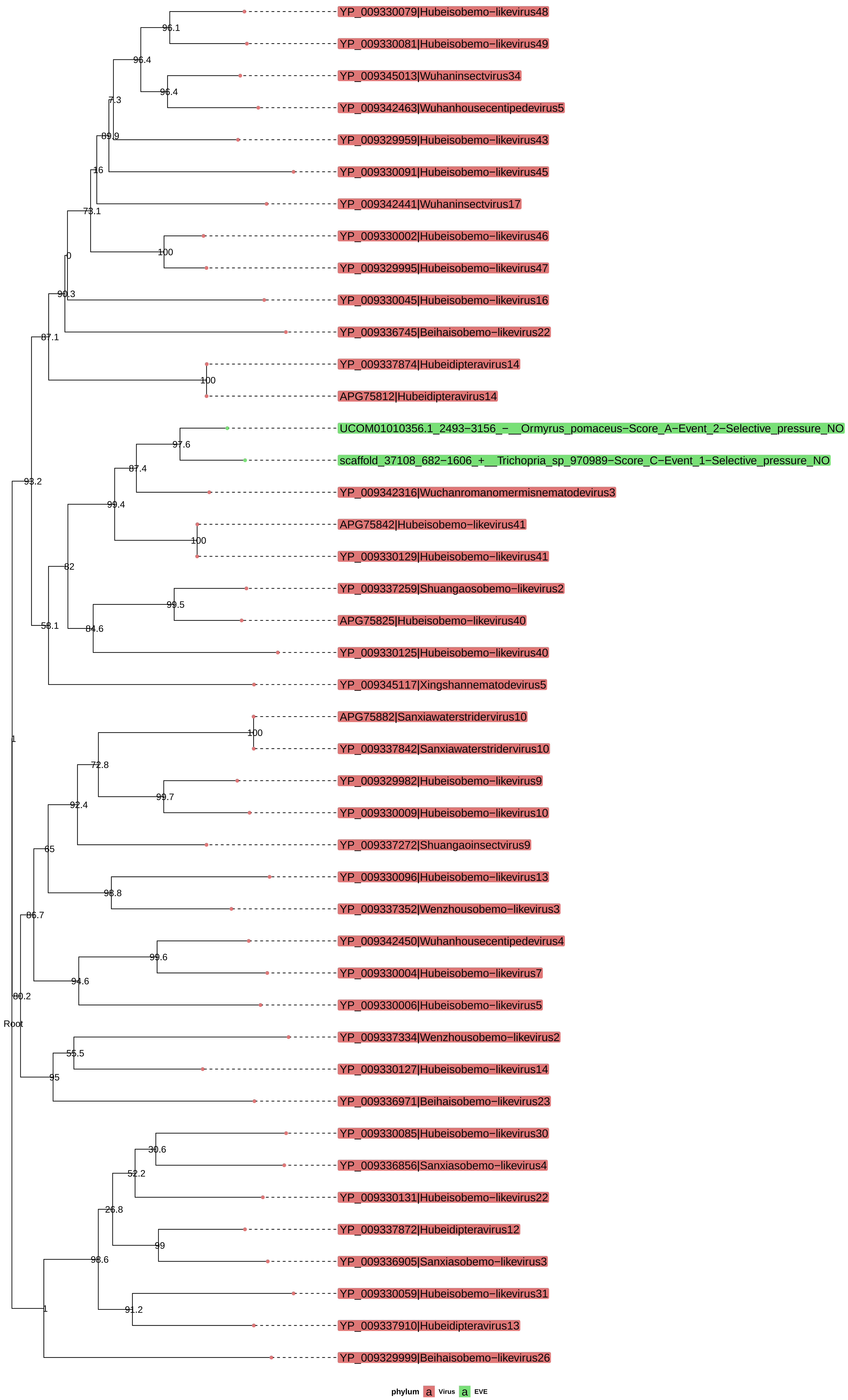

Cluster27053 − families involved : ['Poxviridae'] − putative protein : ['Lipid membrane protein of large eukaryotic DNA viruses']

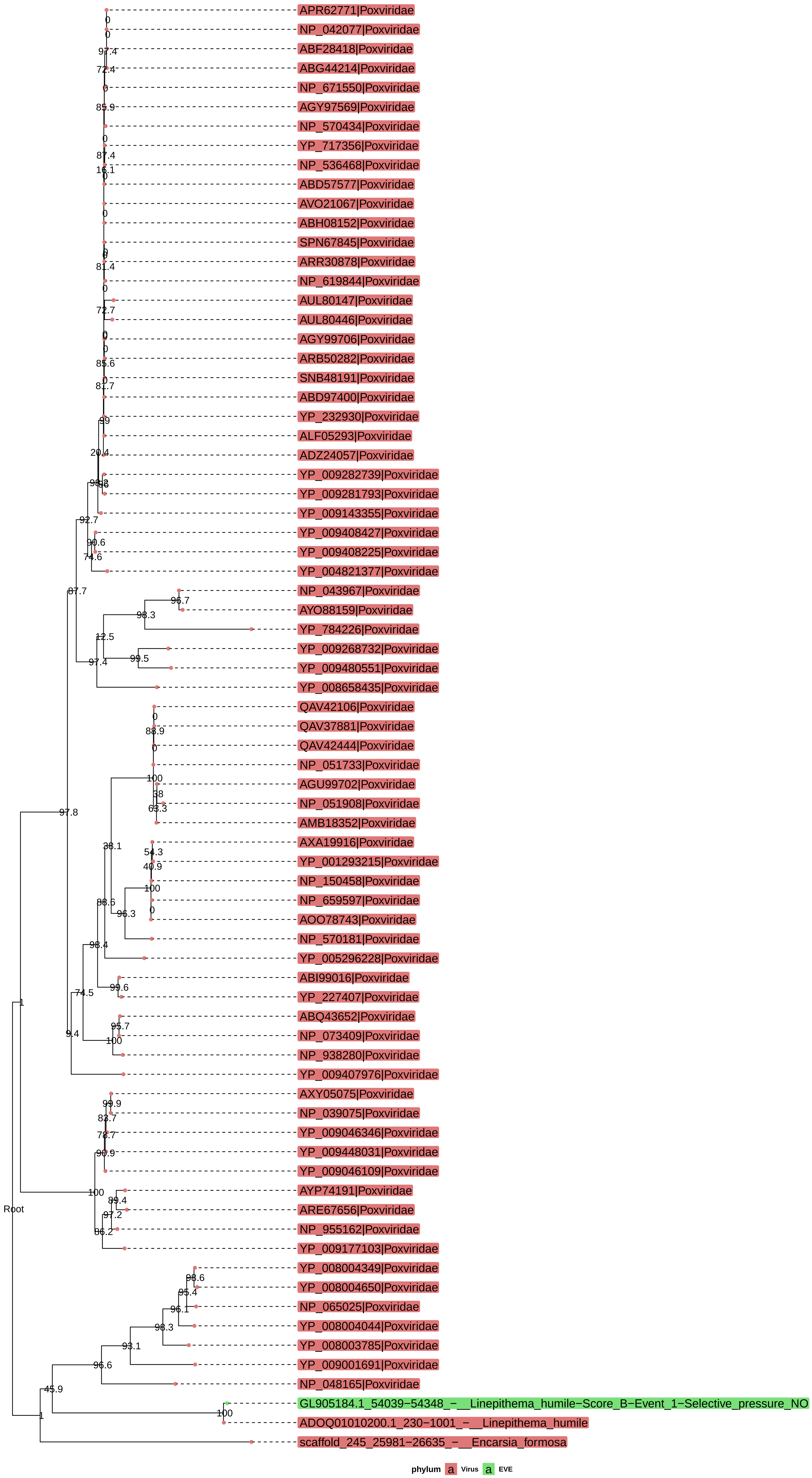

Cluster26053 − families involved : ['Qinviridae'] − putative protein : ['RNA−dependent RNA polymerase']

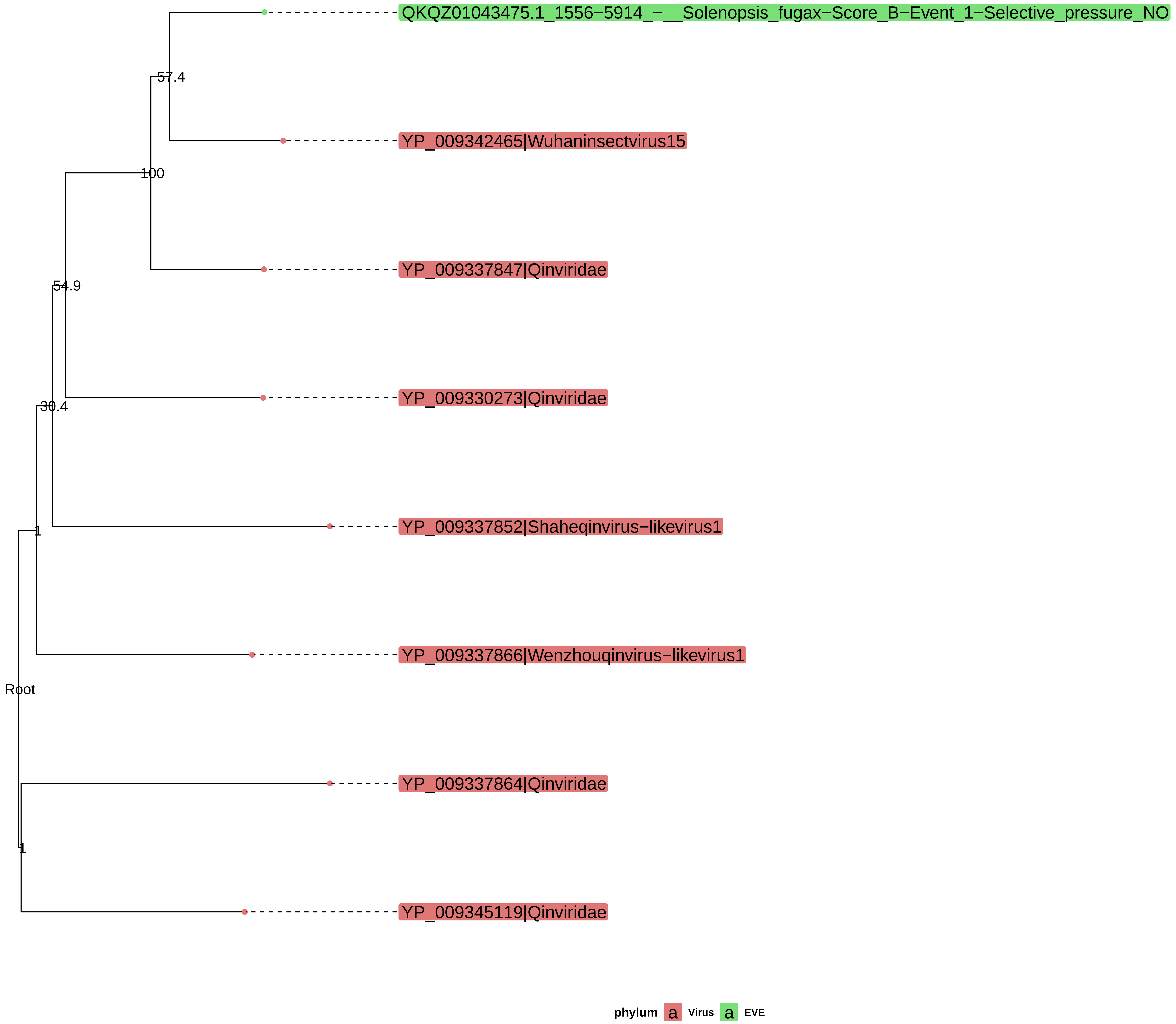

Cluster25561 − families involved : ['Totiviridae', 'Unknown', nan] − putative protein : ['RNA−directed RNA polymerase (EC 2.7.7.48)', nan]

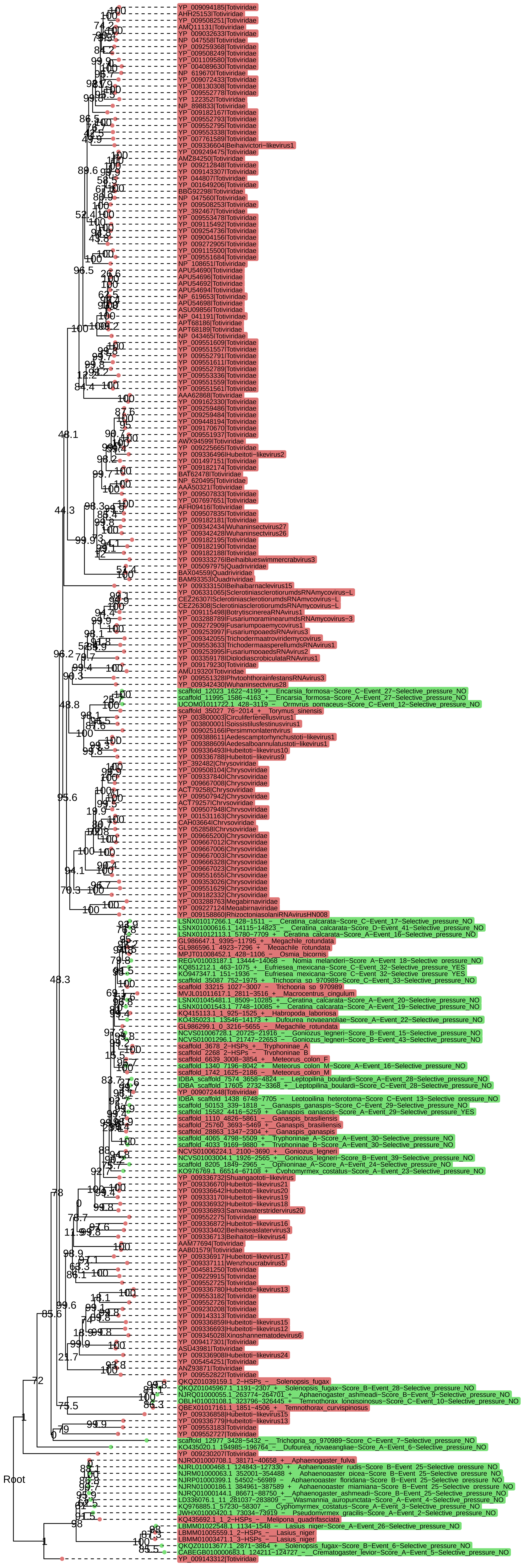

Cluster25147 − families involved : ['Parvoviridae'] − putative protein : ['NS3']

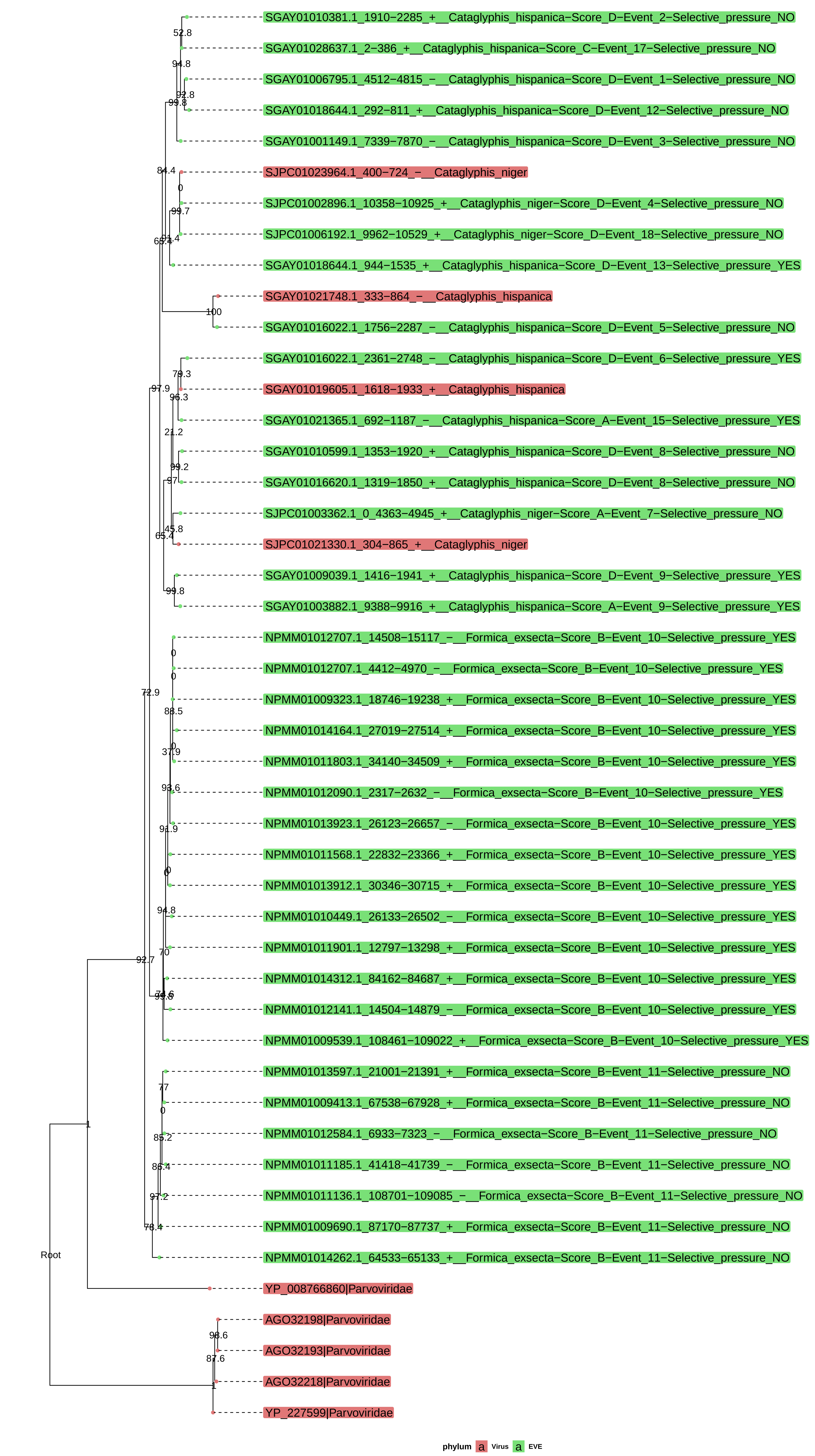

Cluster24263 − families involved : ['Phycodnaviridae'] − putative protein : ['Putative membrane protein']

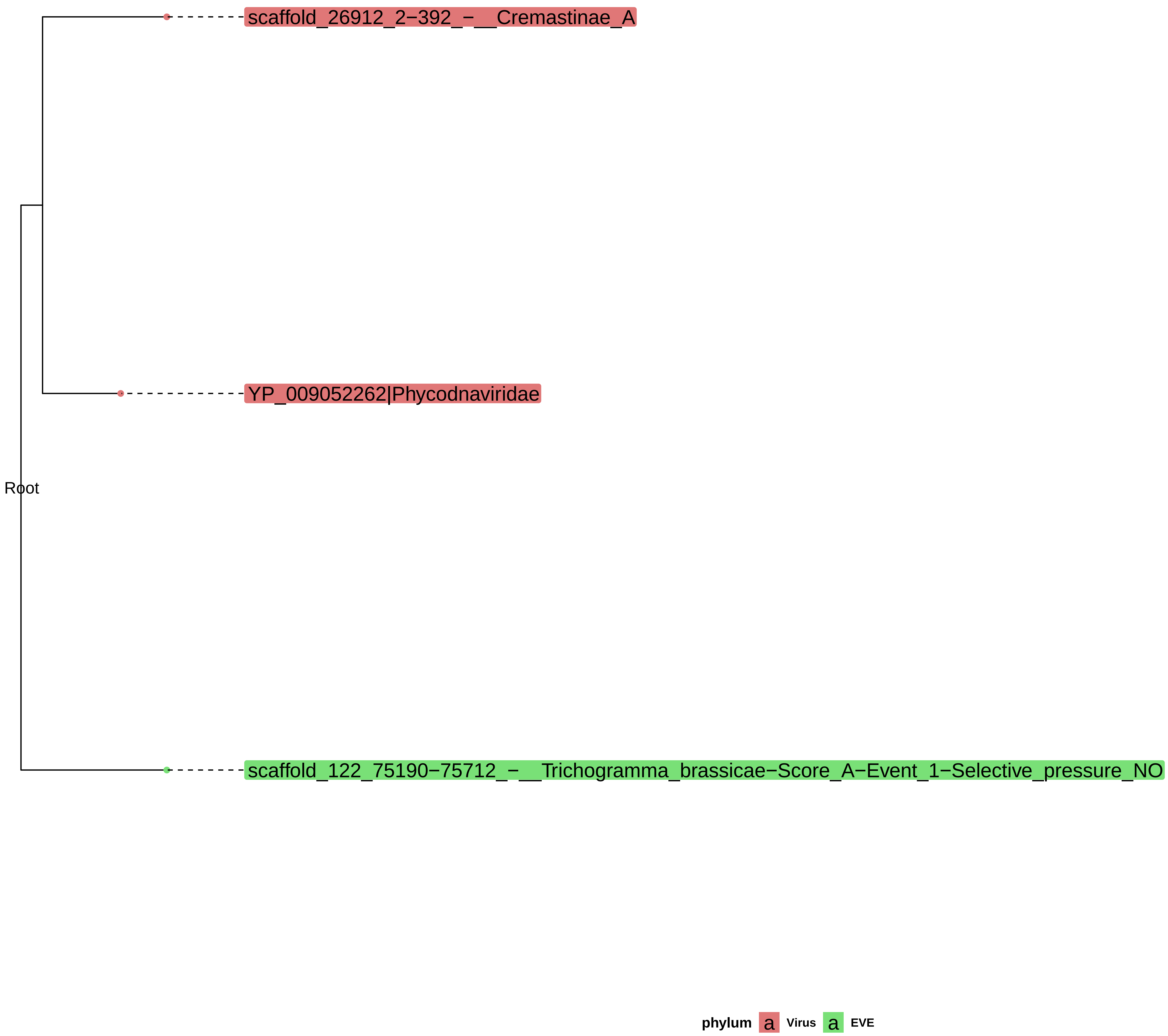

Cluster23952 − families involved : ['Reoviridae'] − putative protein : ['RNA−directed RNA polymerase (EC 2.7.7.48)']

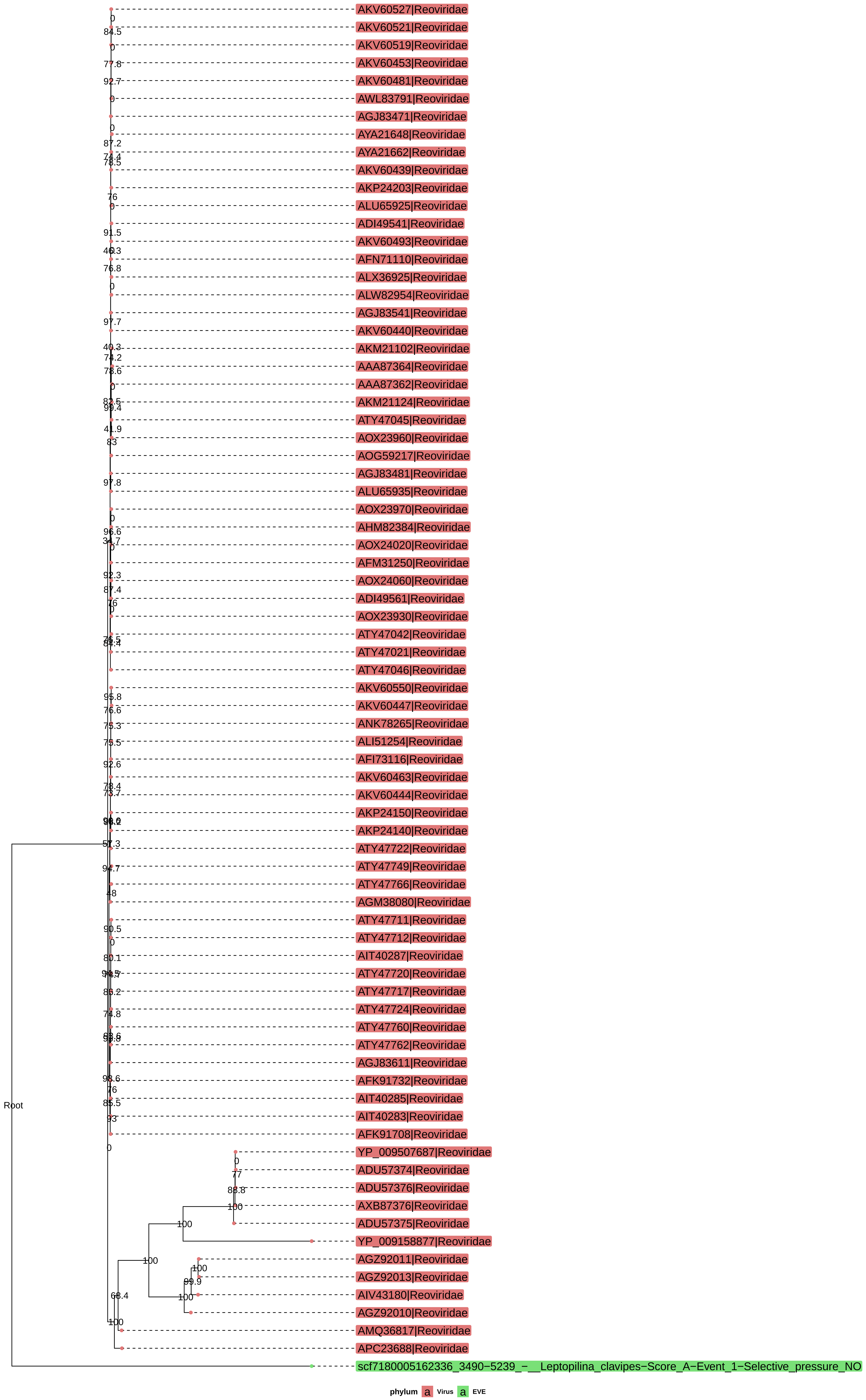

Cluster23500 − families involved : ['Poxviridae'] − putative protein : ['Uncharacterized protein']

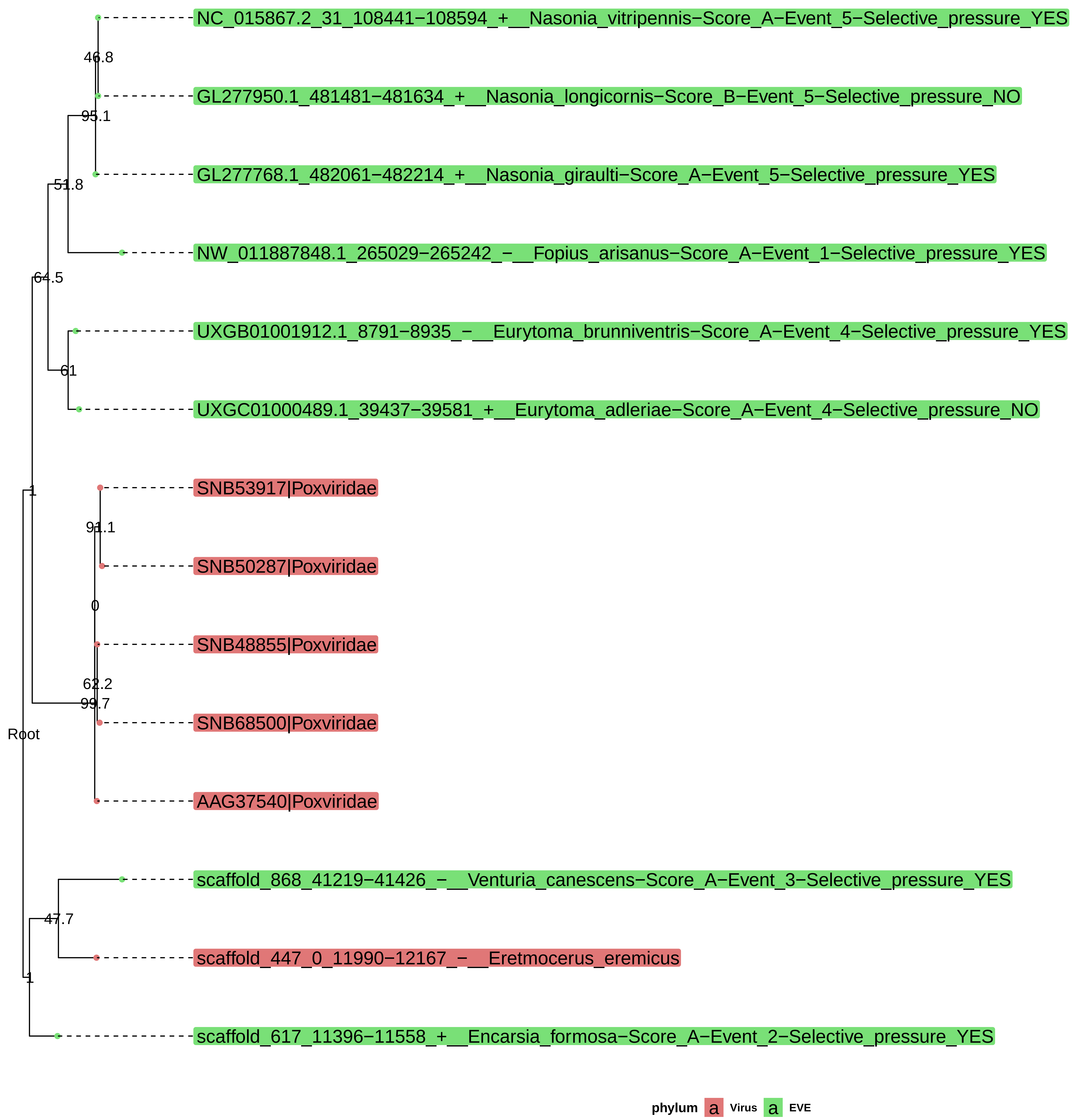

Cluster23367 − families involved : ['Tombusviridae'] − putative protein : ['Capsid protein']

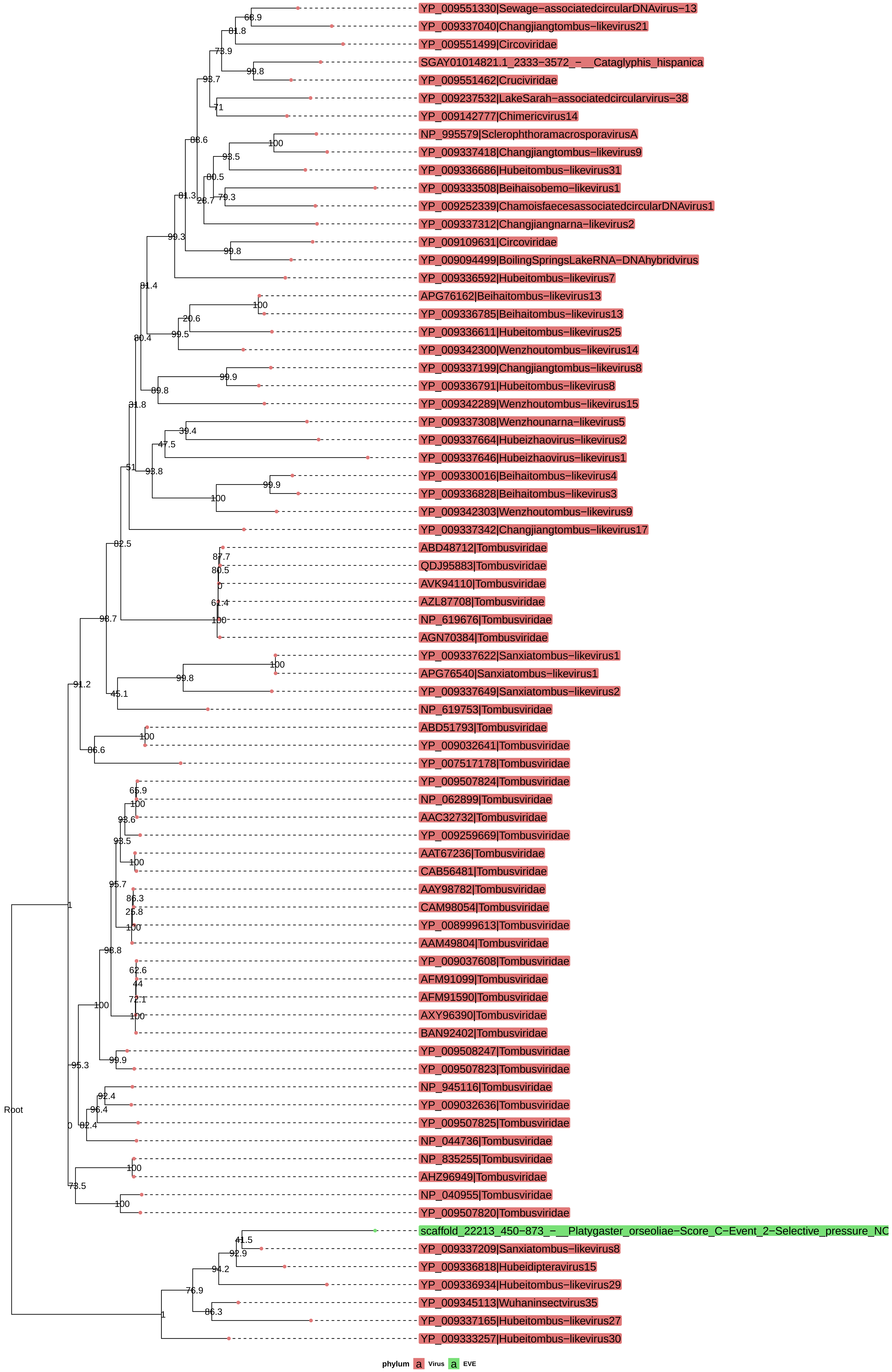

Cluster23306 − families involved : ['Poxviridae'] − putative protein : ['zinc−finger of a C2HC−type']

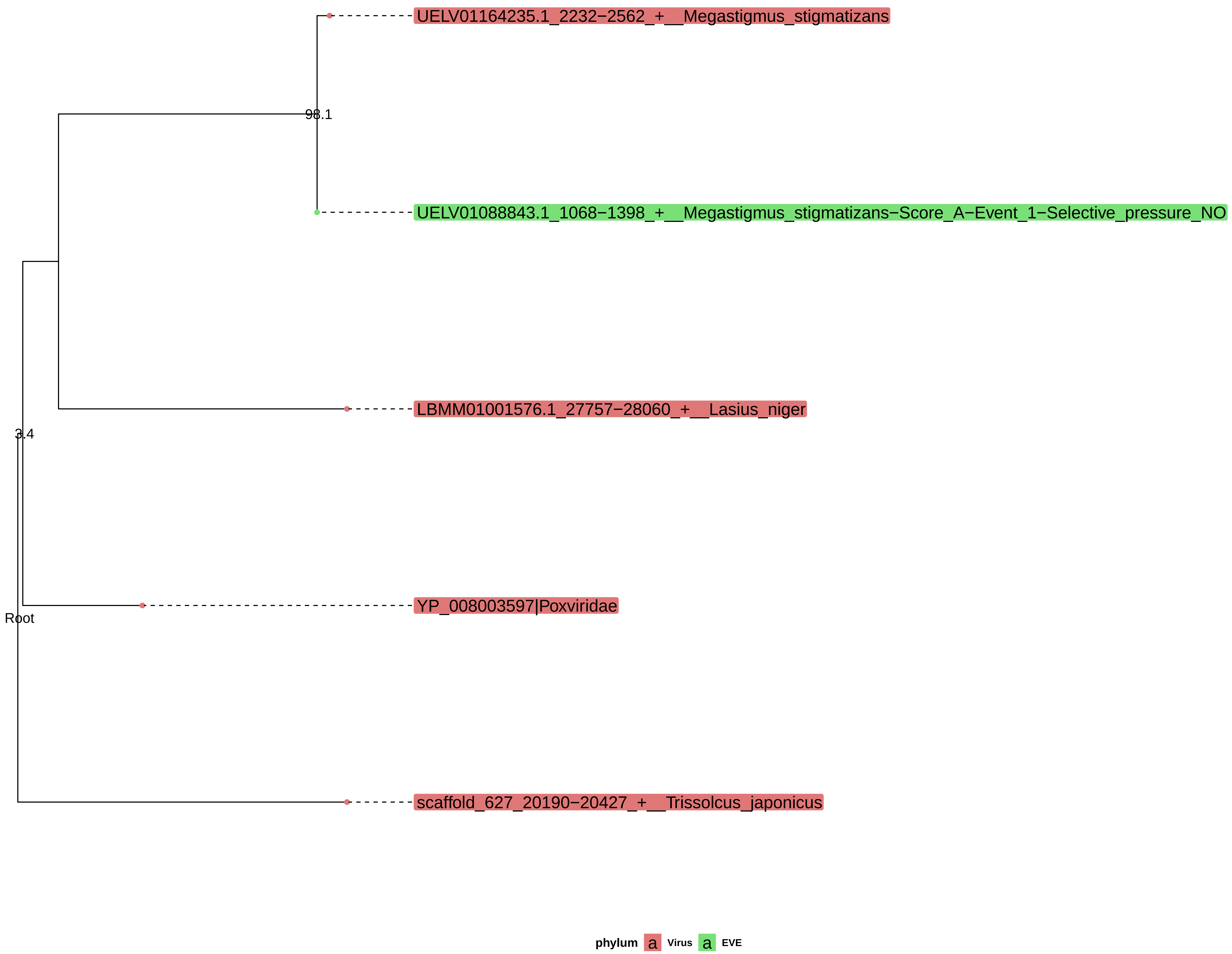

Cluster7293 − families involved : ['Circoviridae', 'Genomoviridae', nan] − putative protein : ['ATP−dependent helicase Rep (RepP) (Replication−associated protein)']

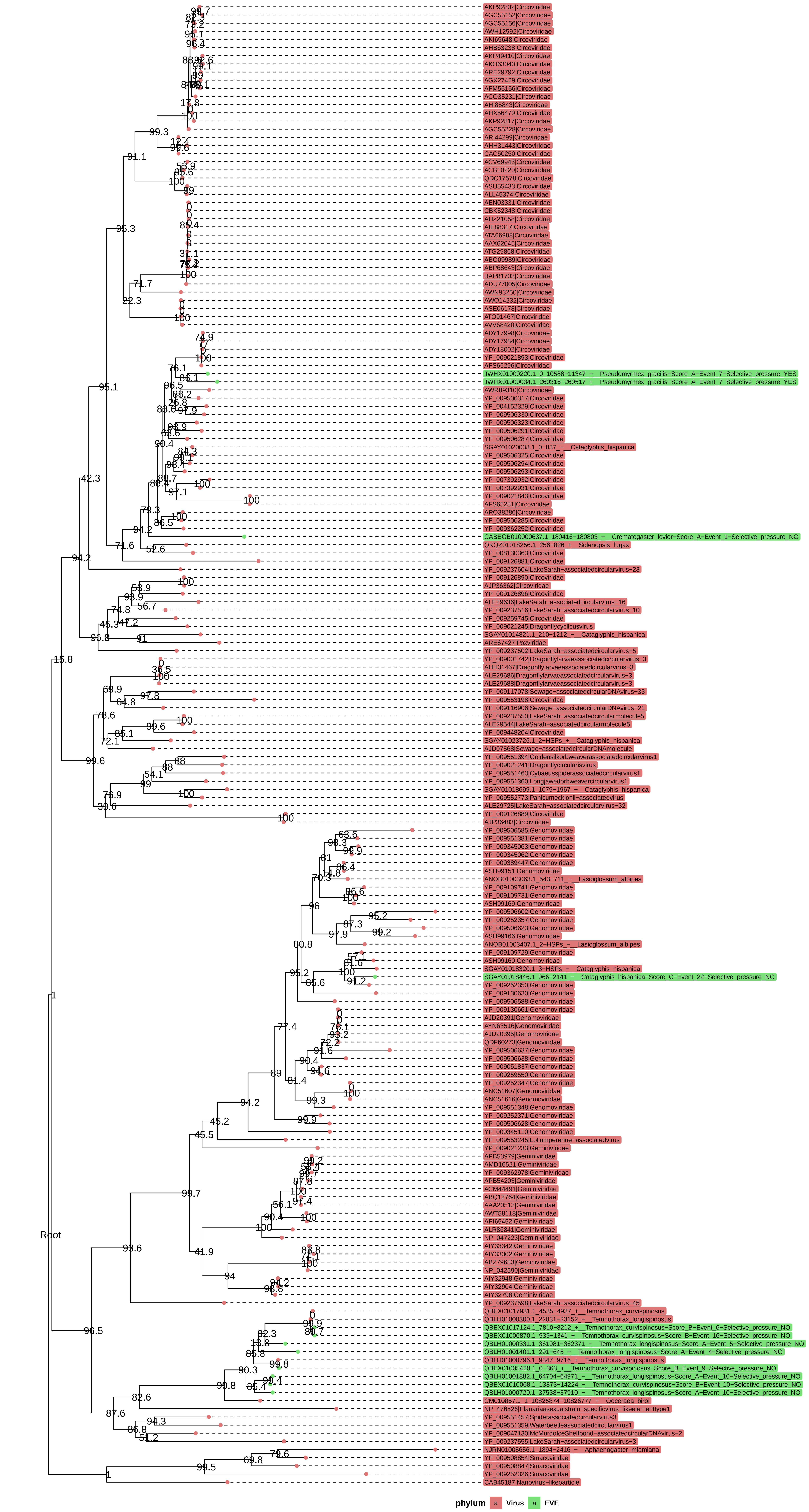

Cluster22627 − families involved : ['Circoviridae'] − putative protein : ['Capsid protein']

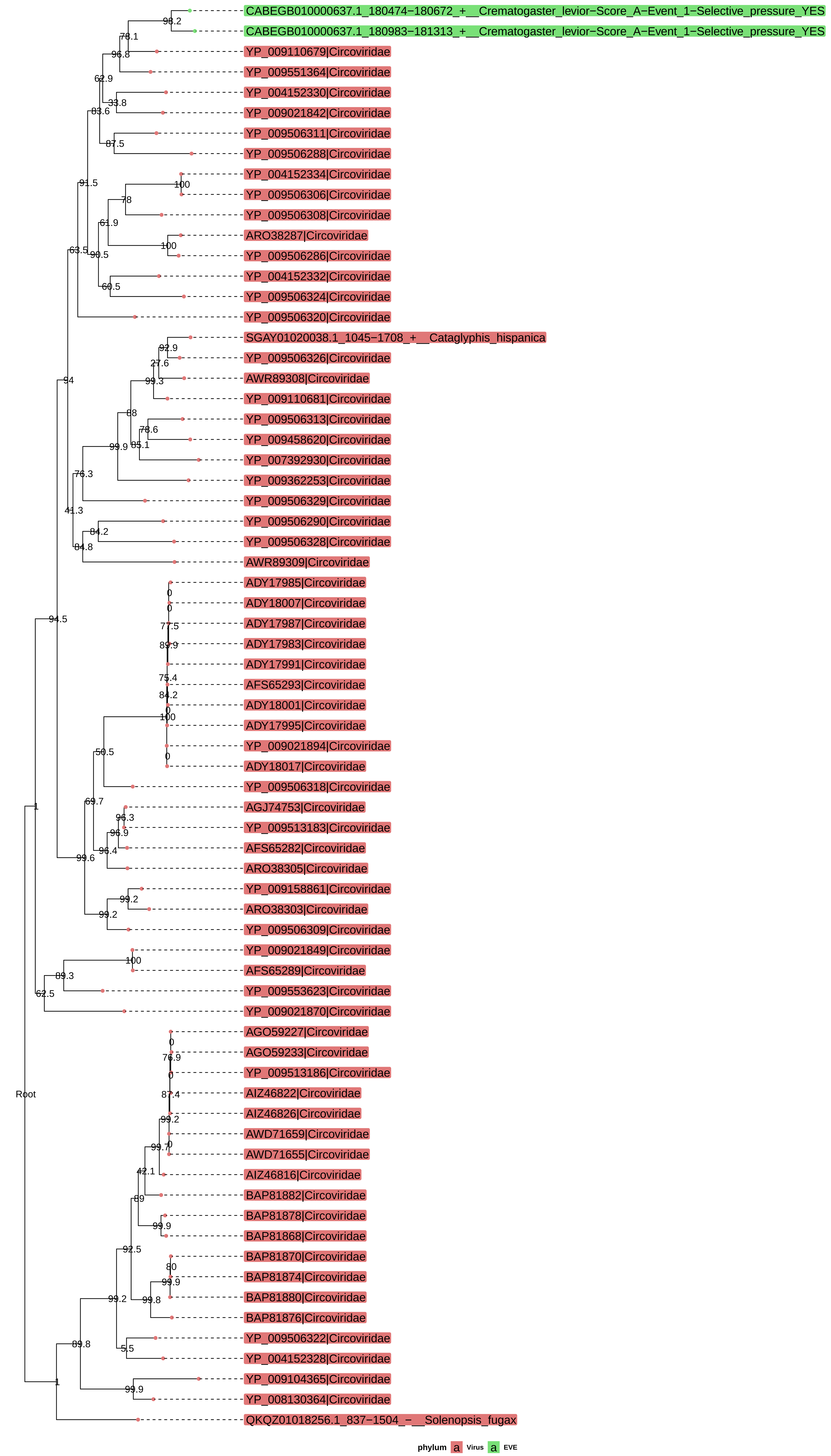

Cluster9821 − families involved : ['Nudiviridae'] − putative protein : ['GrBNV_gp43−like protein']

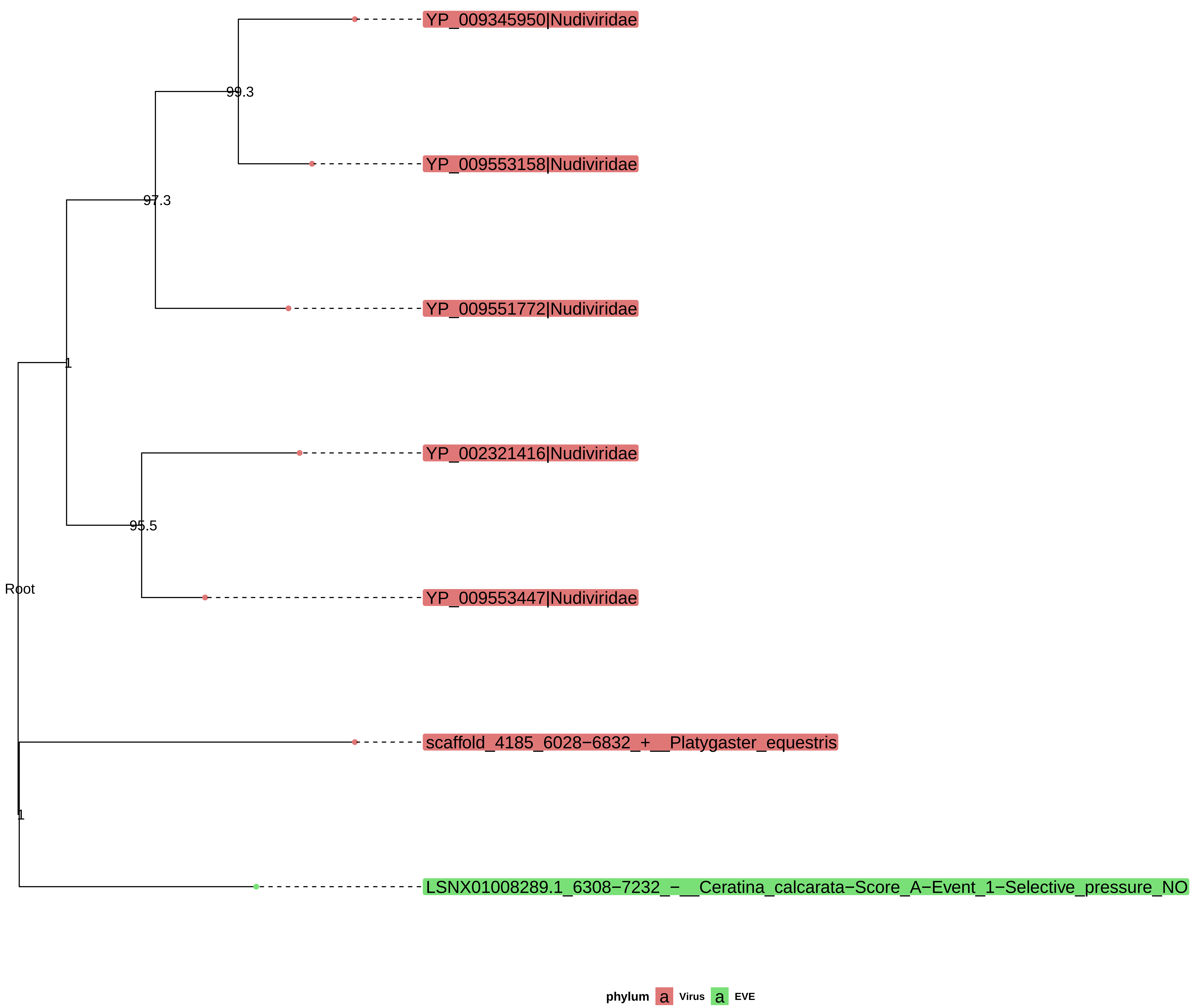

Cluster22770 − families involved : ['Nudiviridae'] − putative protein : ['Centromere protein H (CENP−H)']

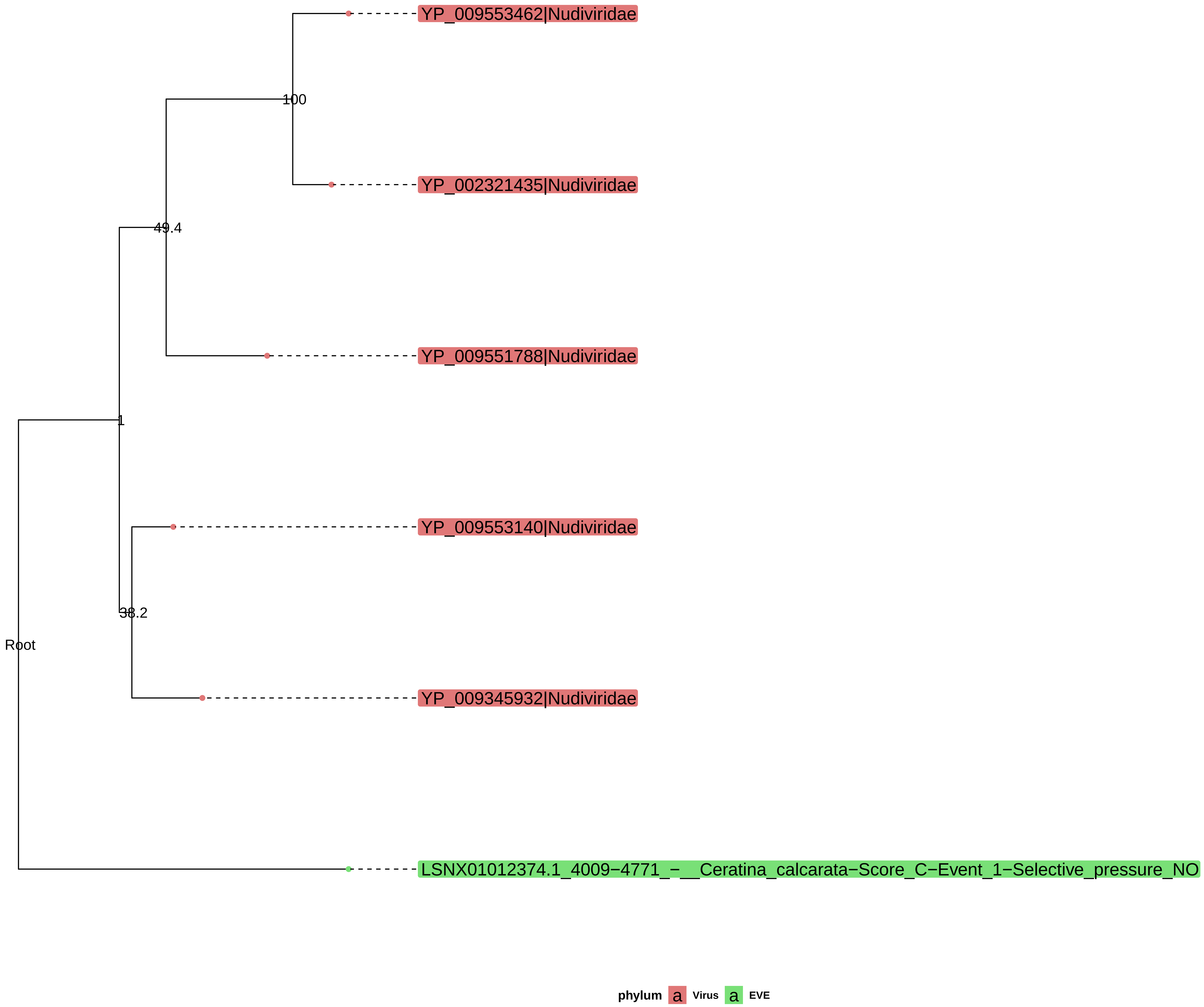

Cluster22177 − families involved : ['Partitiviridae'] − putative protein : ['Putative capsid protein']

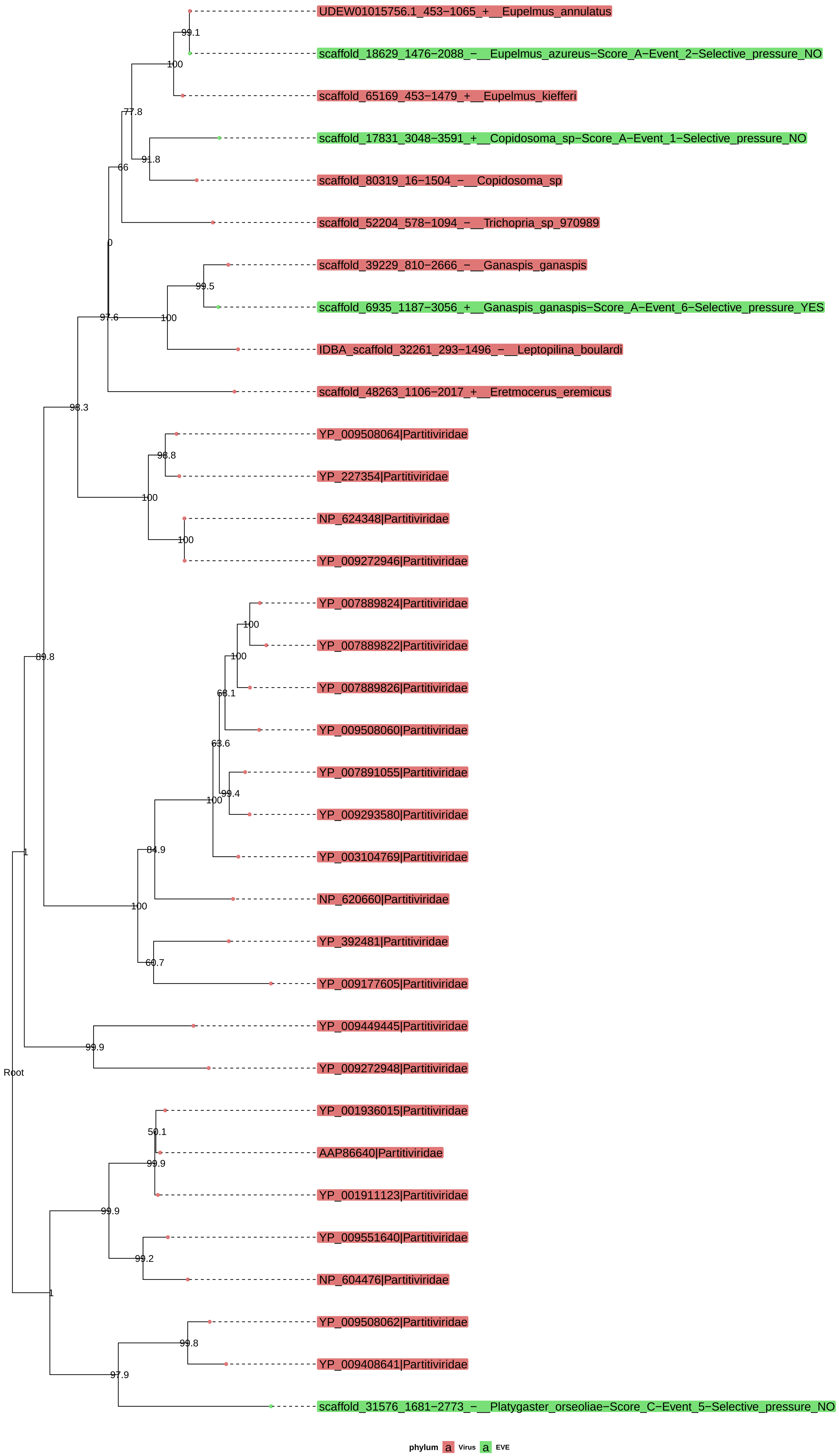

Cluster22017 − families involved : ['Herpesviridae'] − putative protein : [nan]

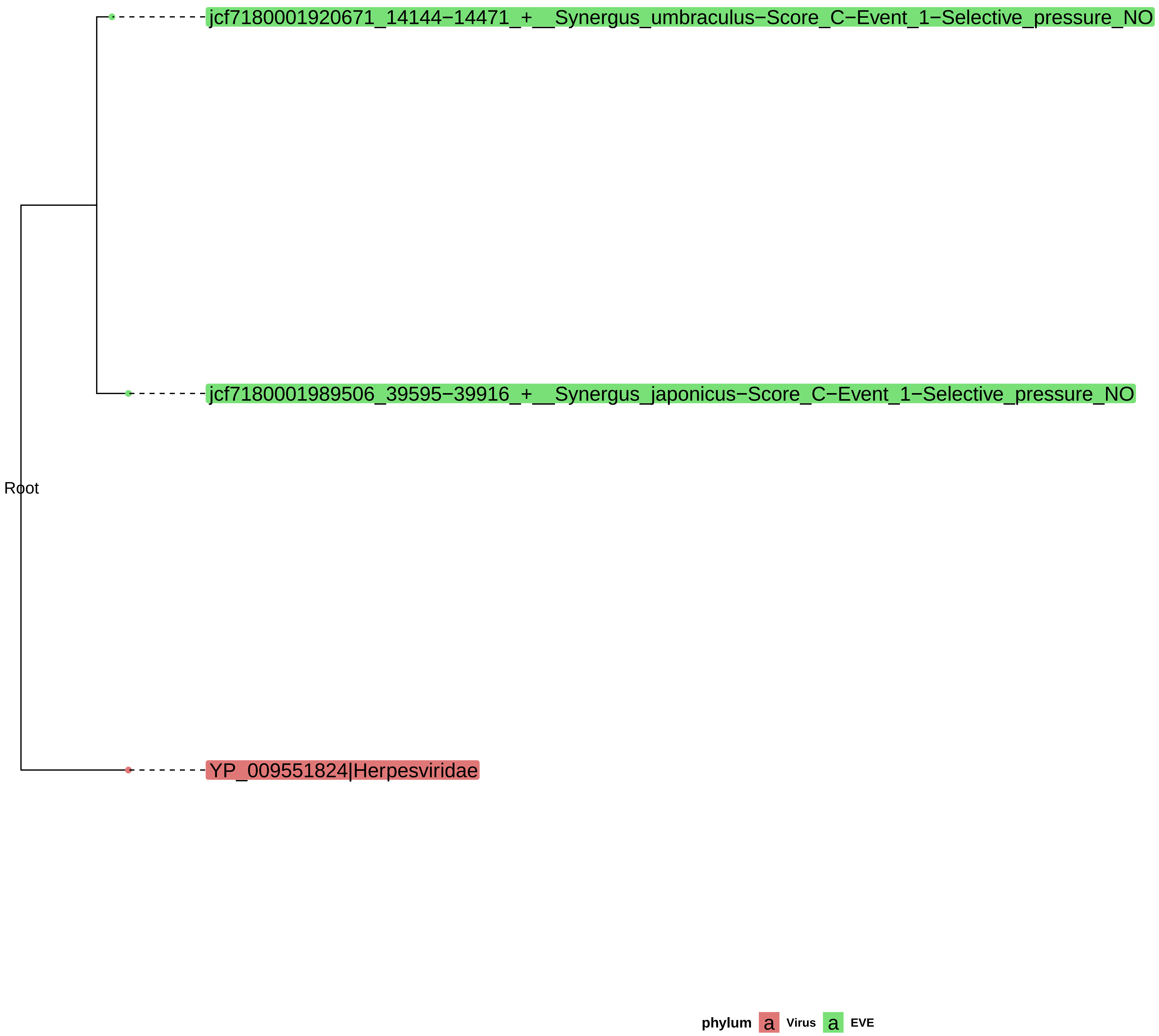

Cluster21728 − families involved : ['Orthomyxoviridae'] − putative protein : ['Nucleoprotein']

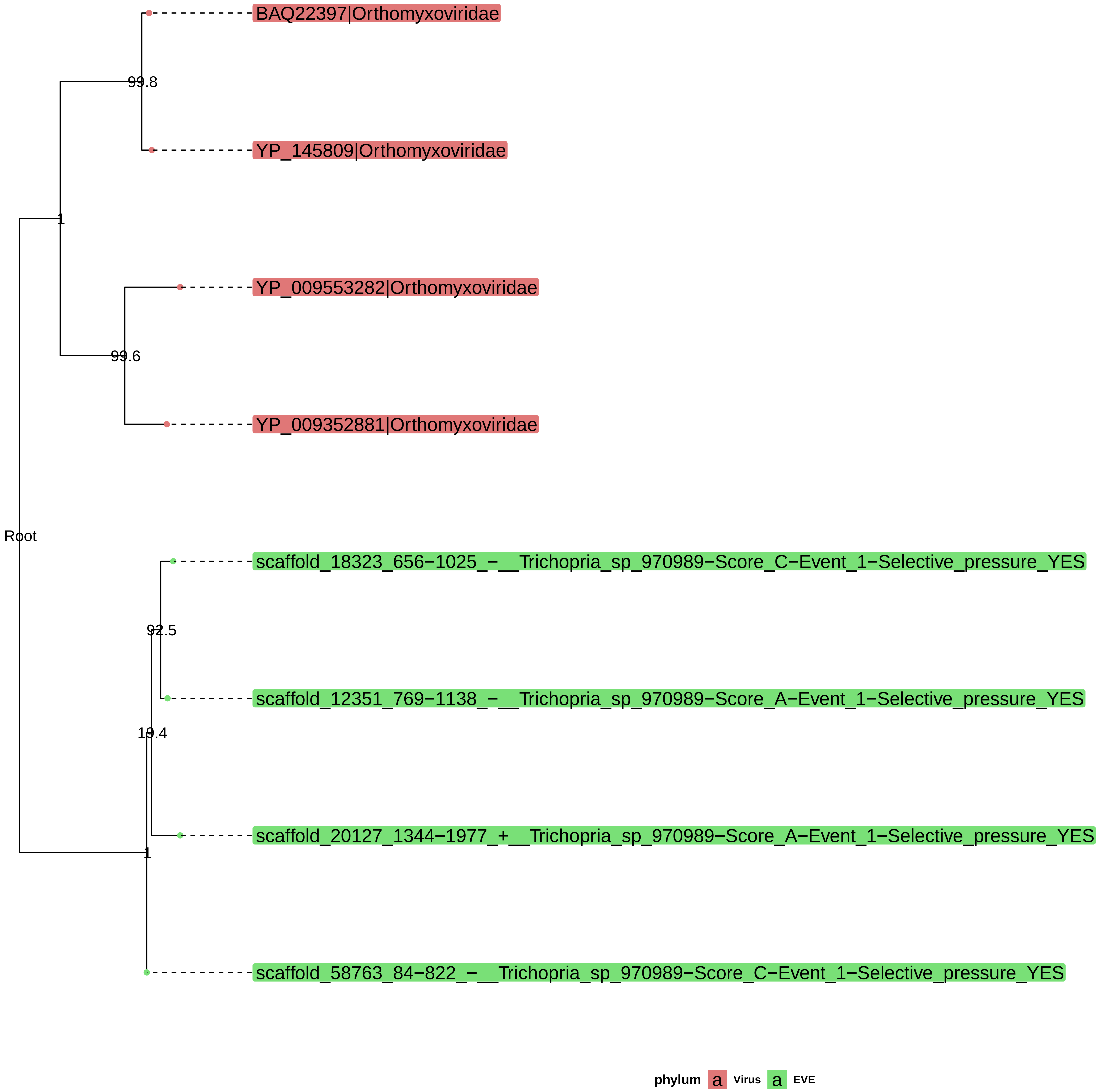

Cluster21304 − families involved : ['Unknown'] − putative protein : ['Proline−alanine−rich protein']

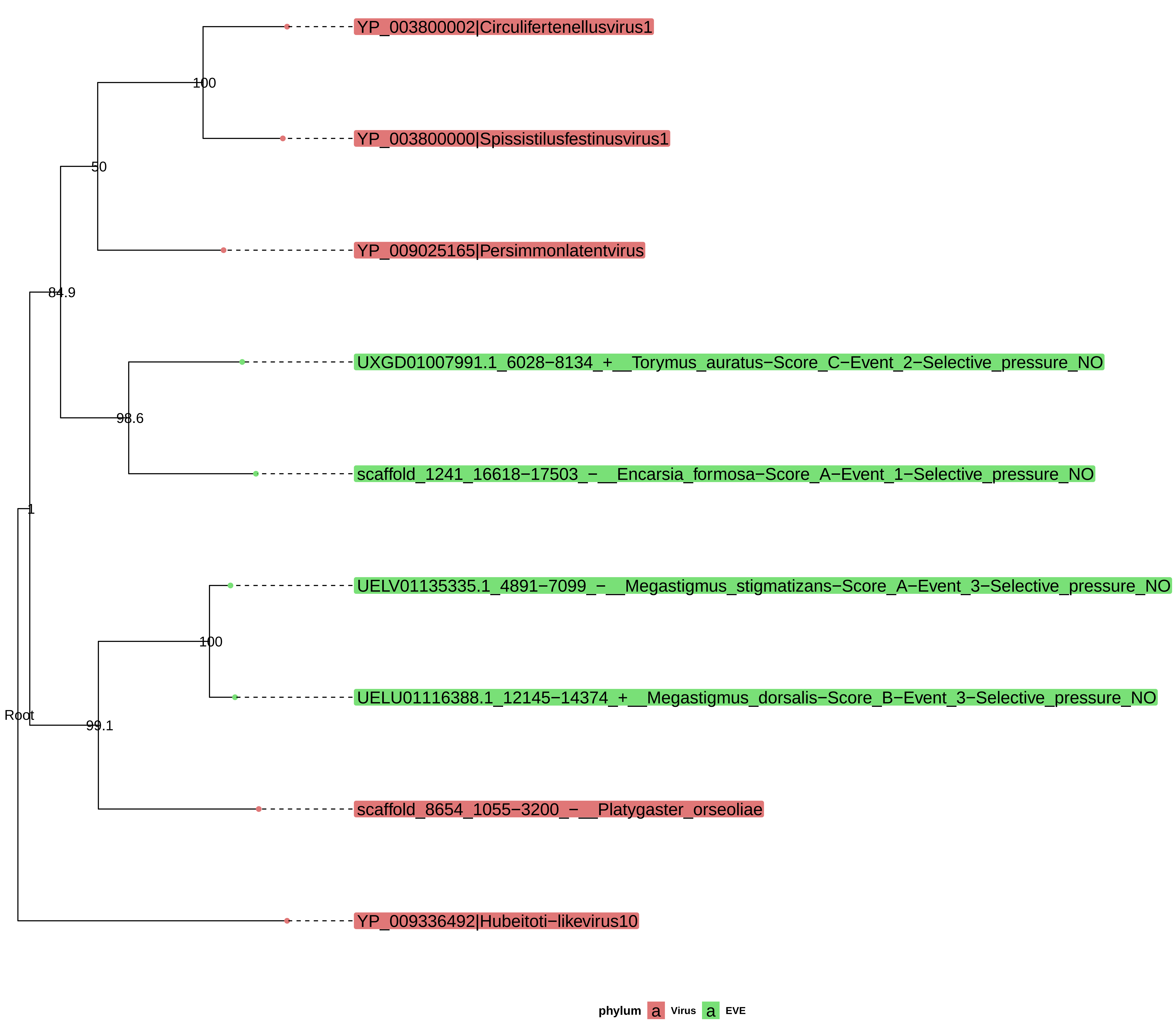

Cluster20940 − families involved : ['Chuviridae'] − putative protein : ['Myelin transcription factor 1']

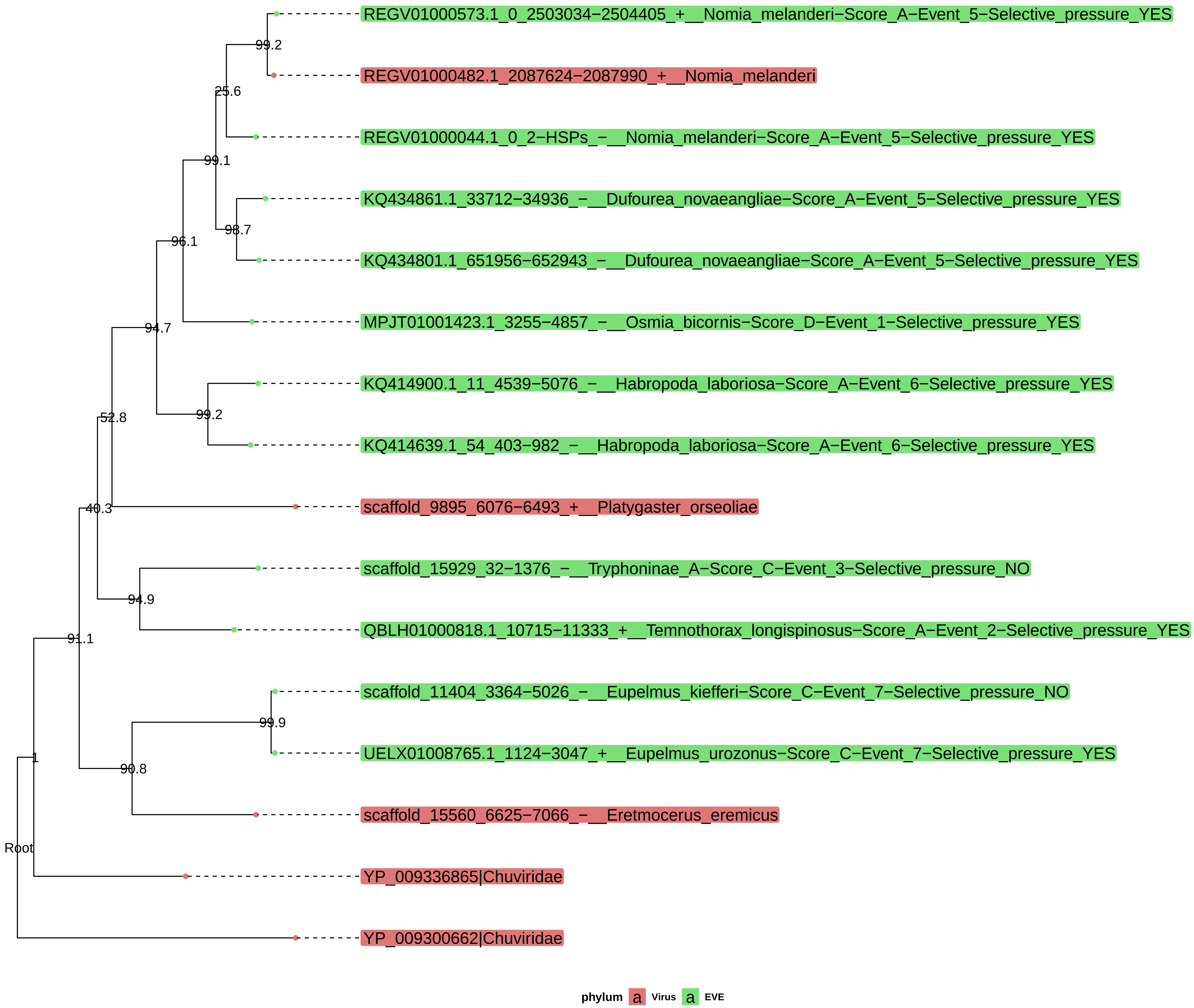

Cluster18385 − families involved : ['Baculoviridae'] − putative protein : ['Uncharacterized protein']

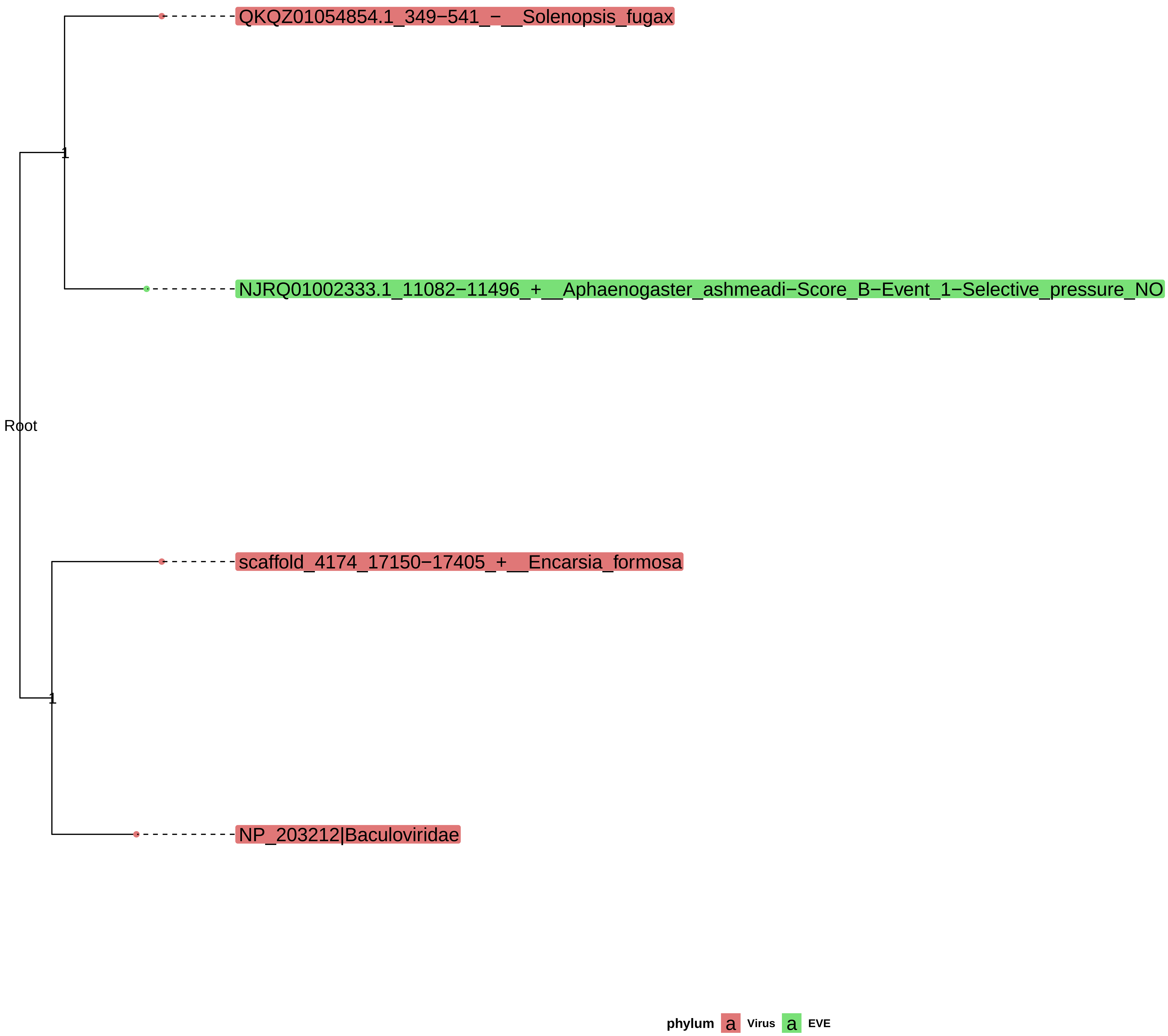

Cluster7695 − families involved : ['Narna−Levi'] − putative protein : ['Bcl−2 homology region 4']

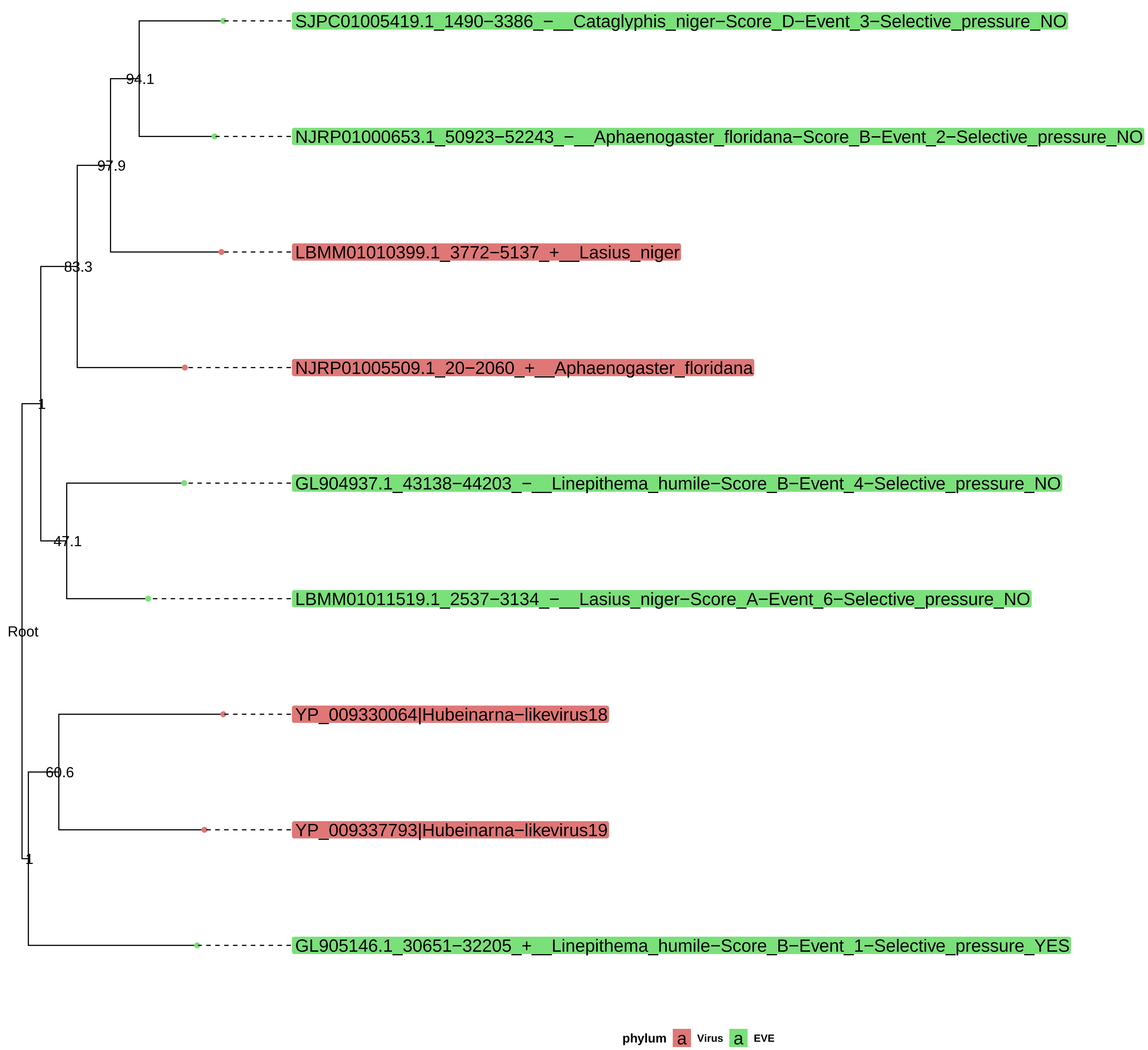

Cluster20094 − families involved : ['Narna−Levi'] − putative protein : ['RNA−dependent RNA polymerase']

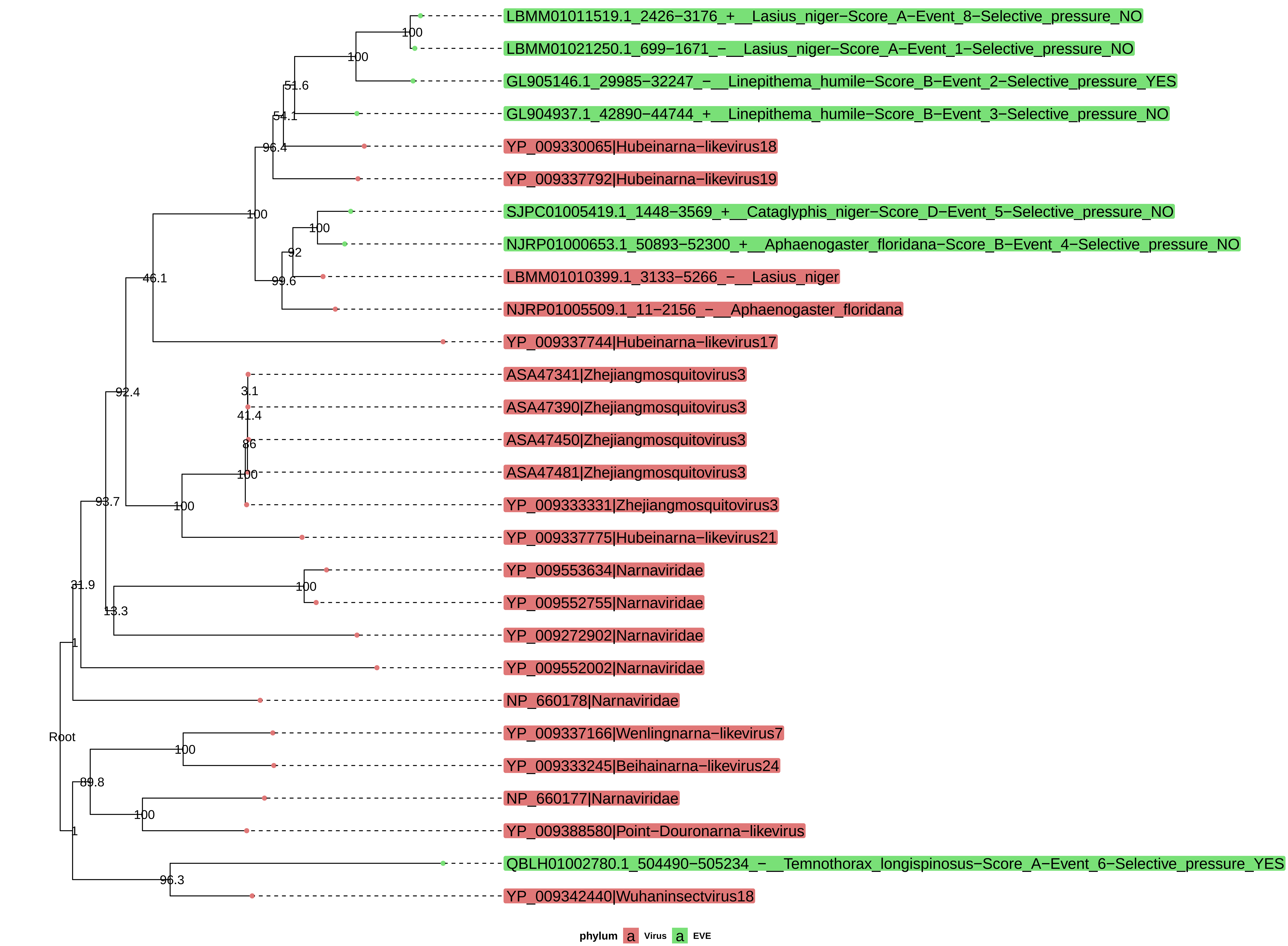

Cluster20057 − families involved : ['Hepe−Virga', 'Abisko−like'] − putative protein : ['Capsid protein']

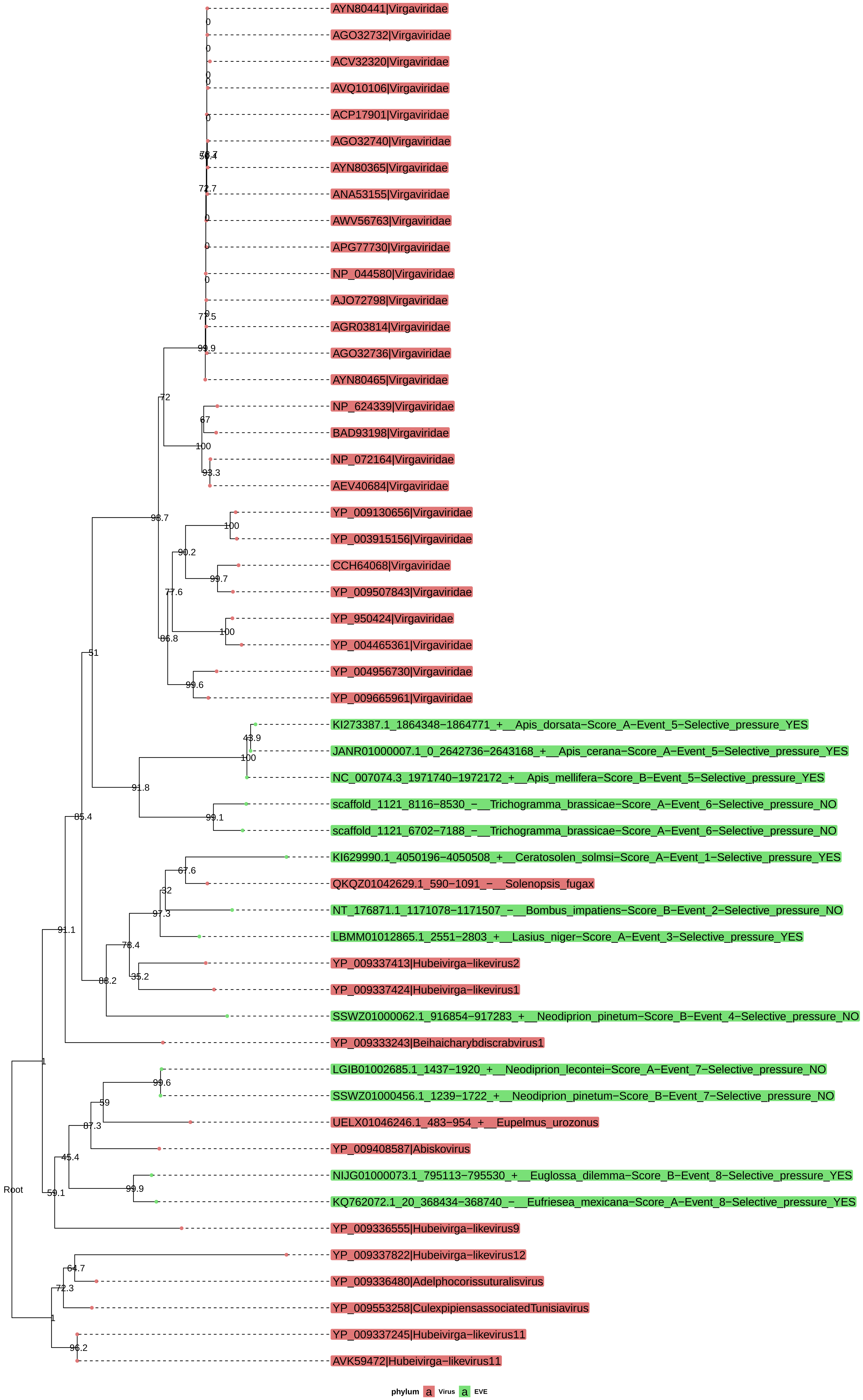

Cluster19346 − families involved : ['Reoviridae'] − putative protein : ['Uncharacterized protein']

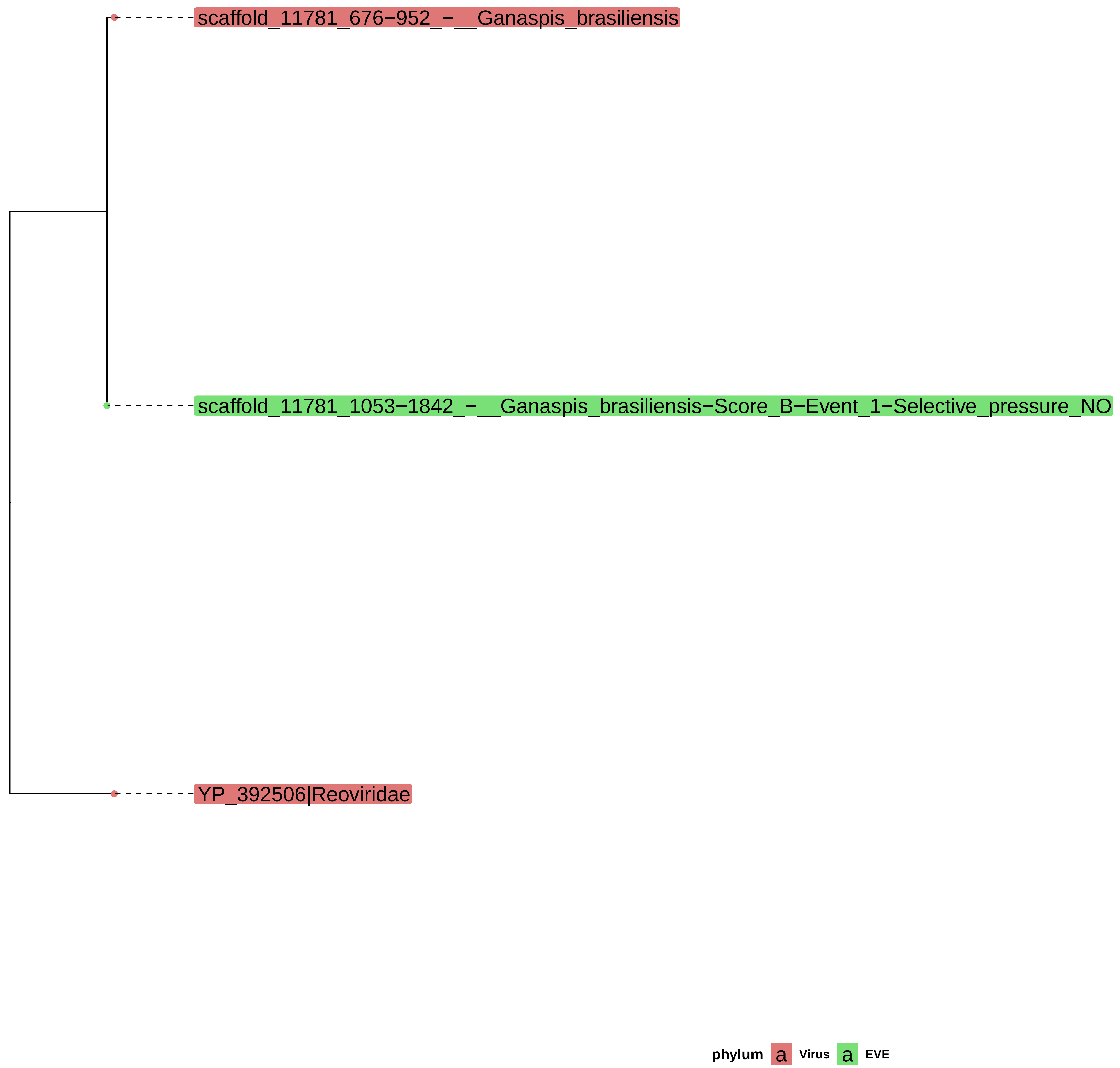

Cluster19345 − families involved : ['Nudiviridae'] − putative protein : ['p51 late protein']

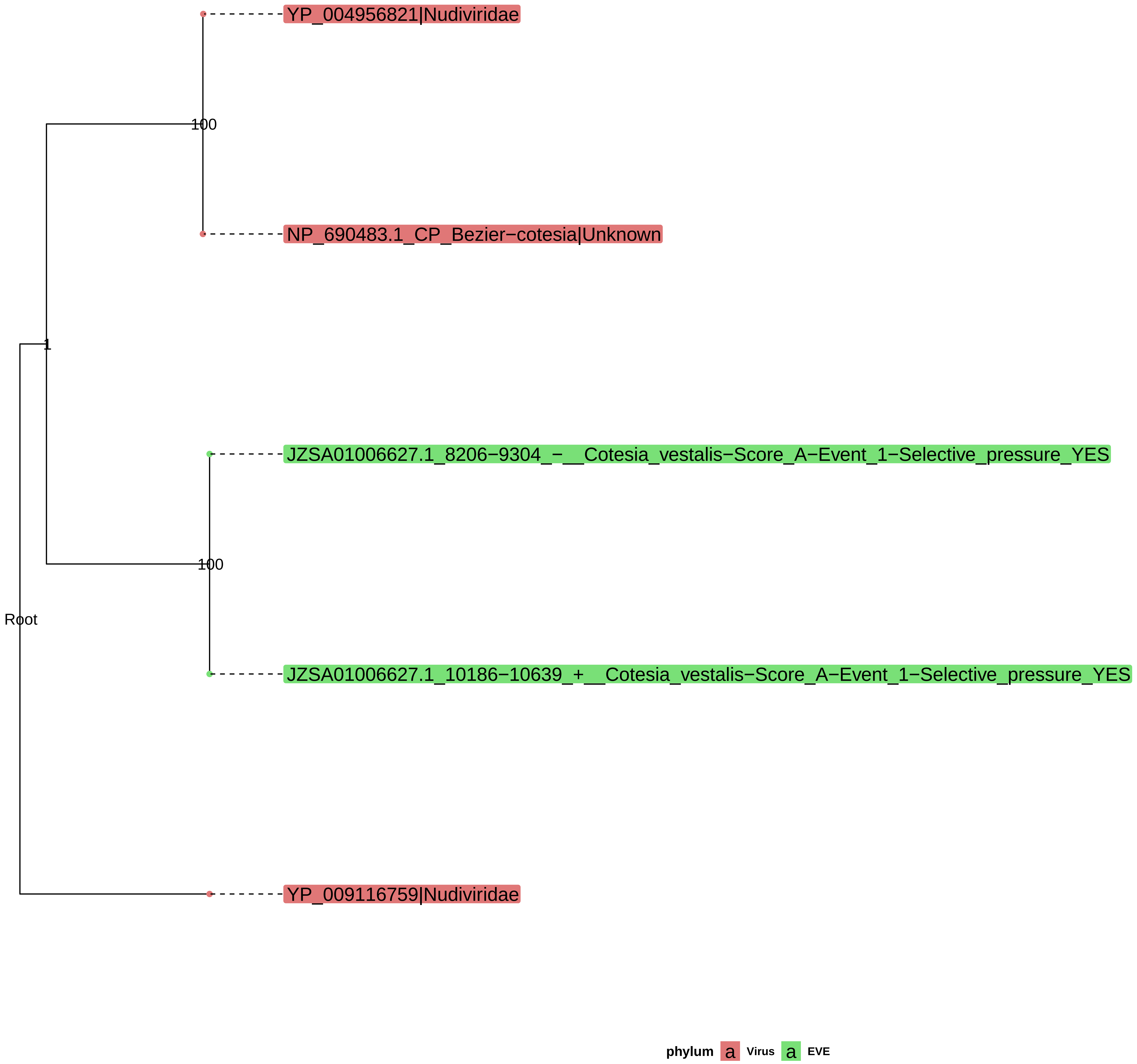

Cluster19040 − families involved : ['Baculoviridae'] − putative protein : ['p35']

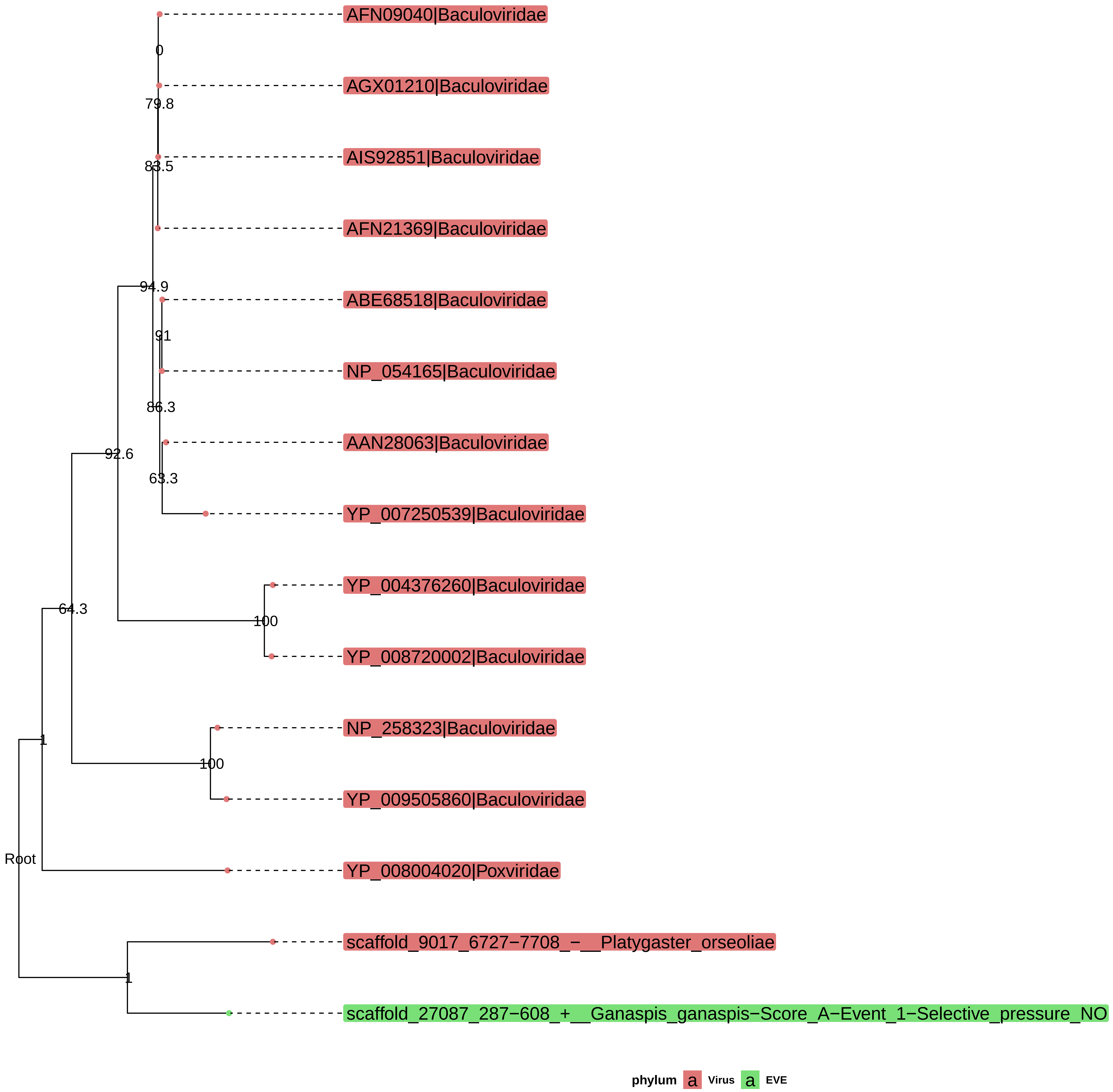

Cluster25342 − families involved : ['Reoviridae'] − putative protein : ['RNA binding protein']

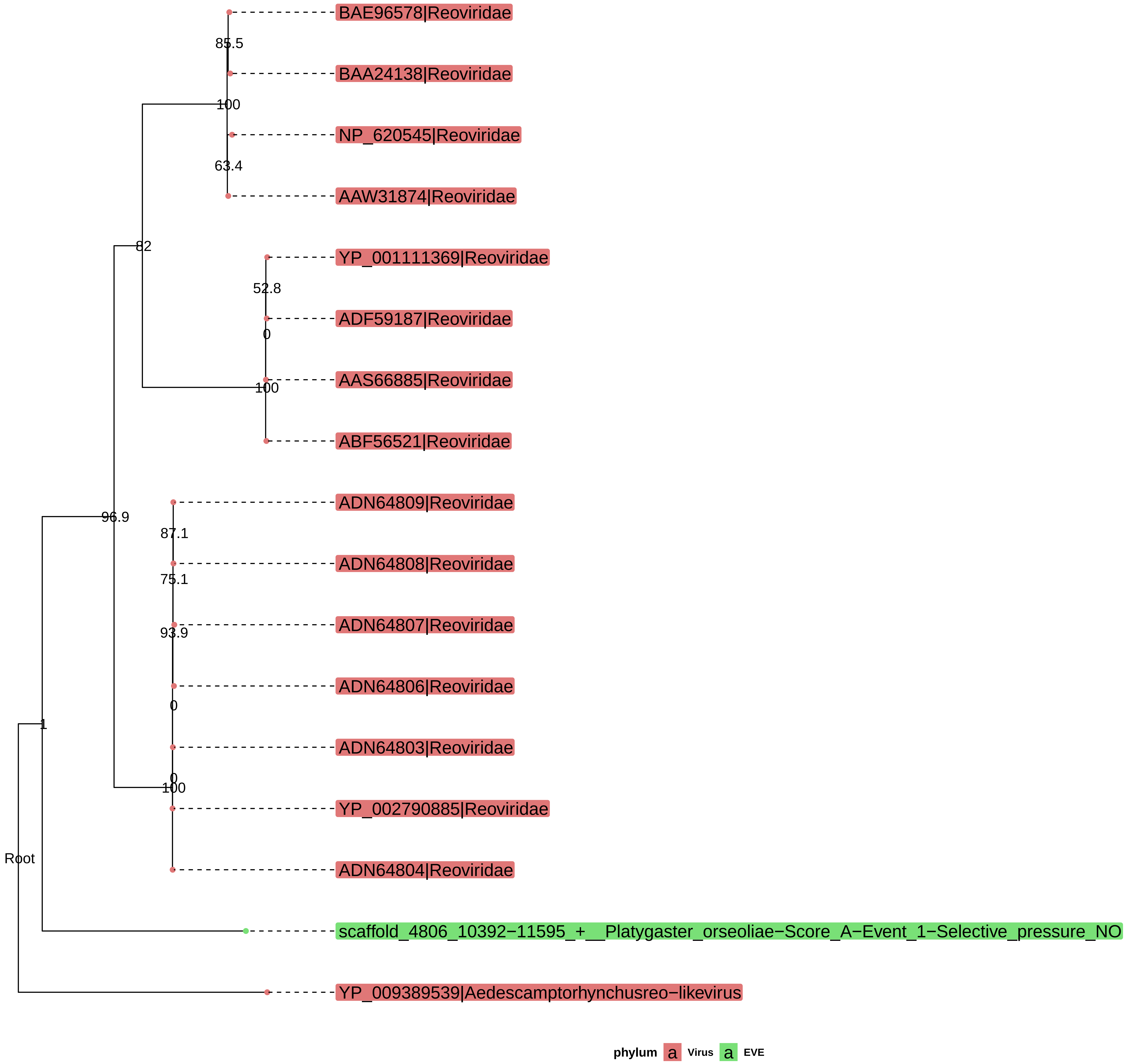

Cluster20626 − families involved : ['Reoviridae'] − putative protein : ['FKBP26_C−terminal']

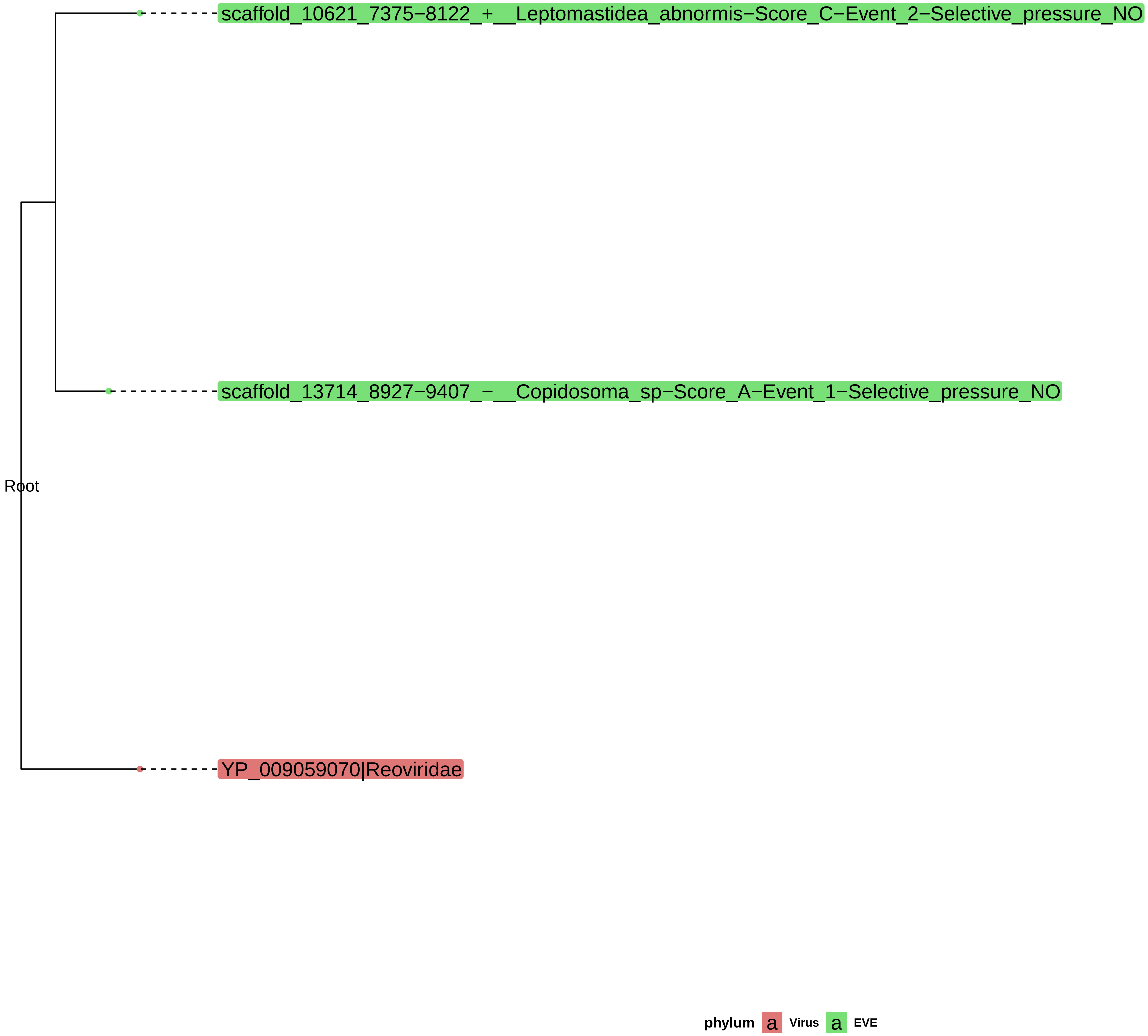

Cluster18836 − families involved : ['Reoviridae'] − putative protein : ['Uncharacterized protein']

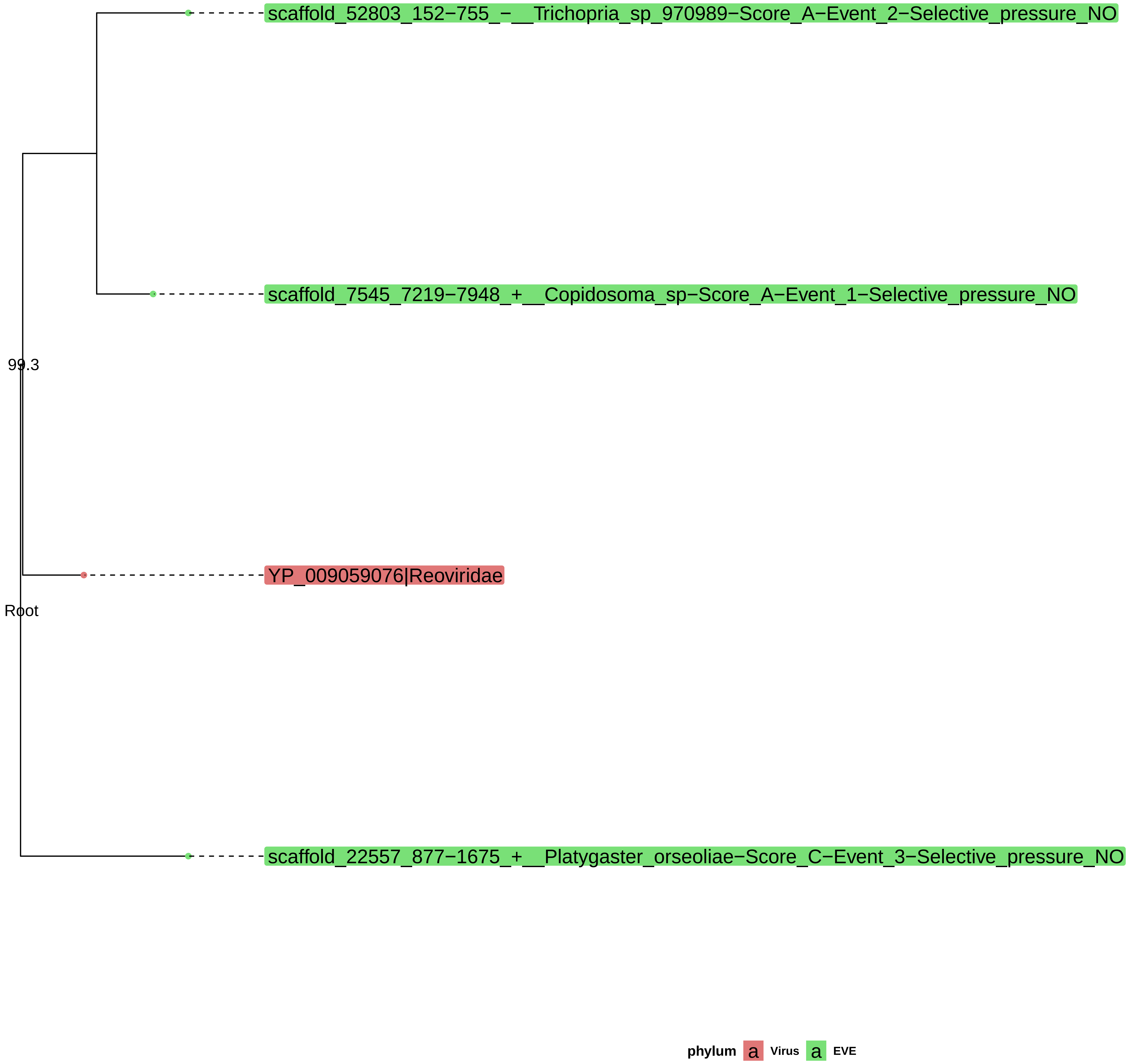

Cluster1870 − families involved : ['Reoviridae'] − putative protein : ['Major core protein']

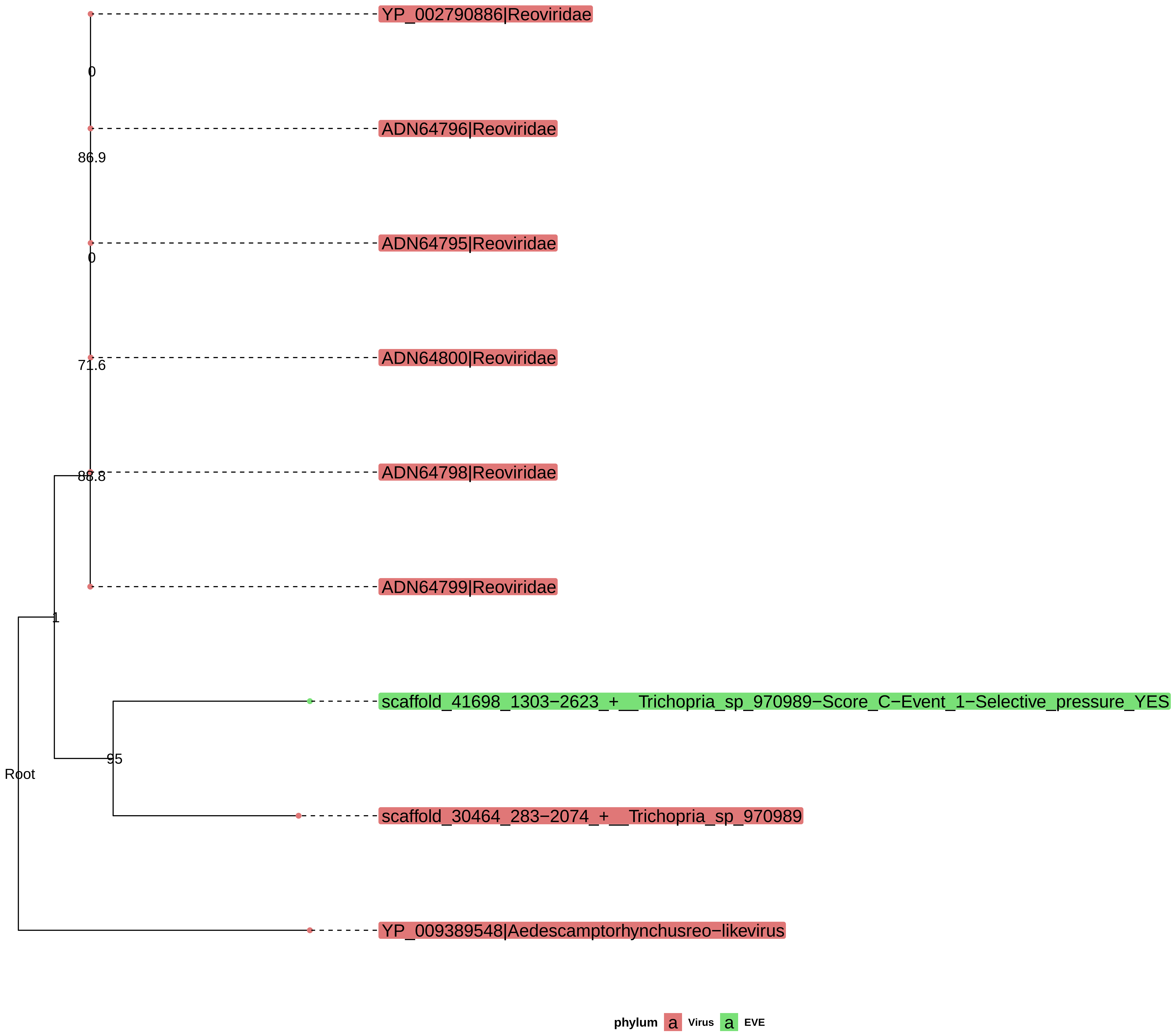

Cluster3348 − families involved : ['Nudiviridae'] − putative protein : ['GrBNV_gp61−like protein']

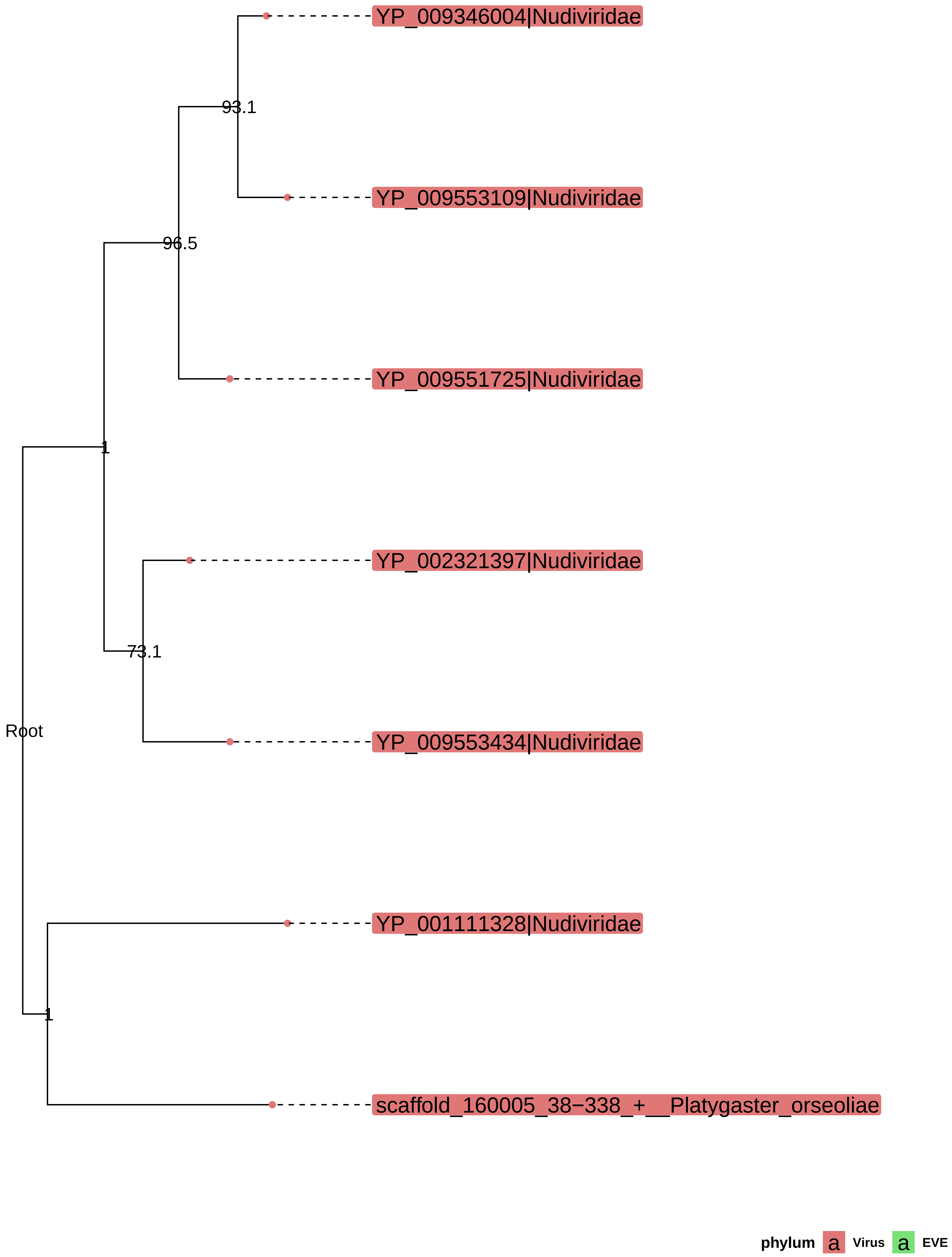

Cluster18256 − families involved : ['Partiti−Picobirna'] − putative protein : ['Uncharacterized protein']

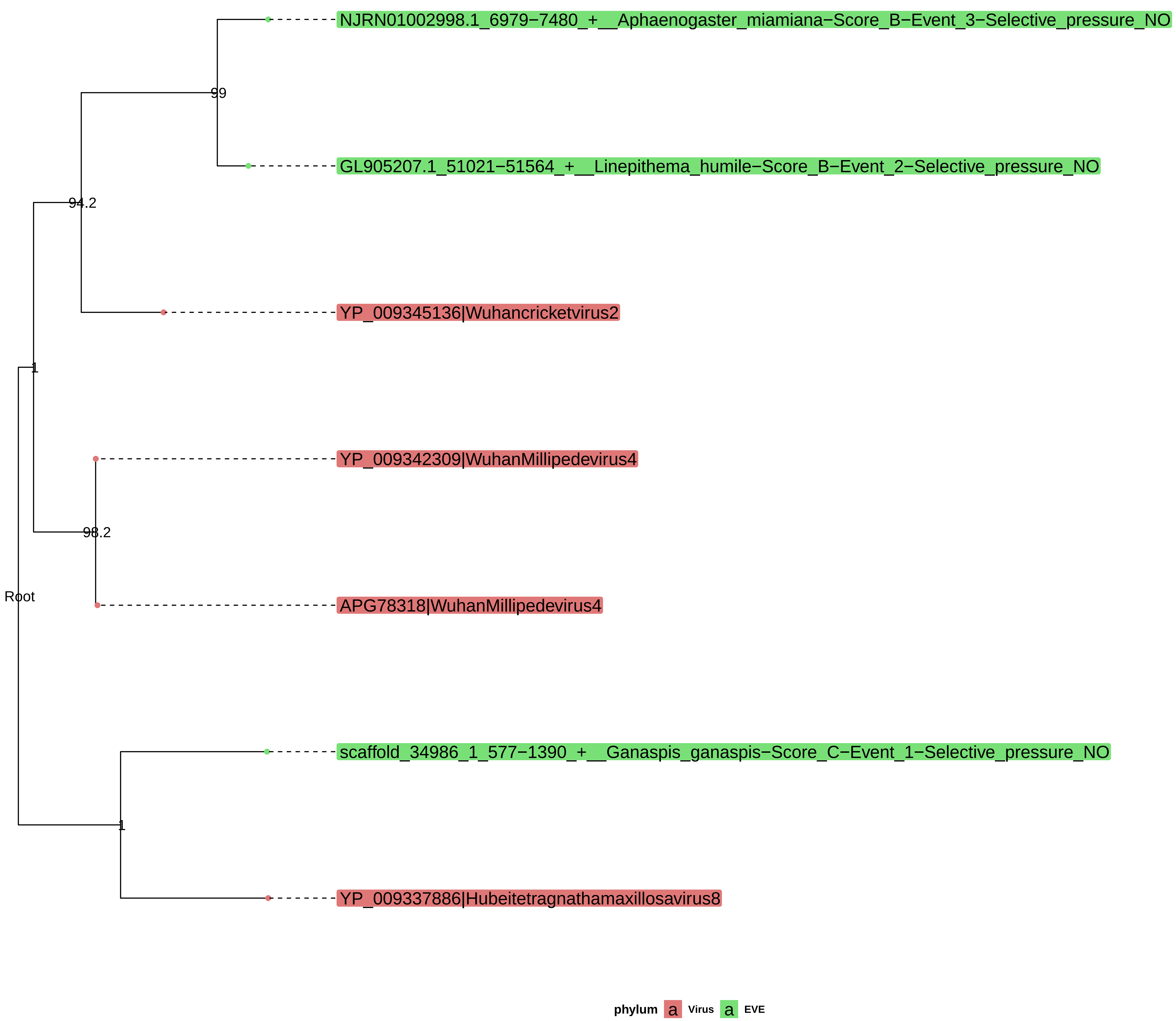

Cluster18042 − families involved : ['Mono−Chu'] − putative protein : ['Uncharacterized protein']

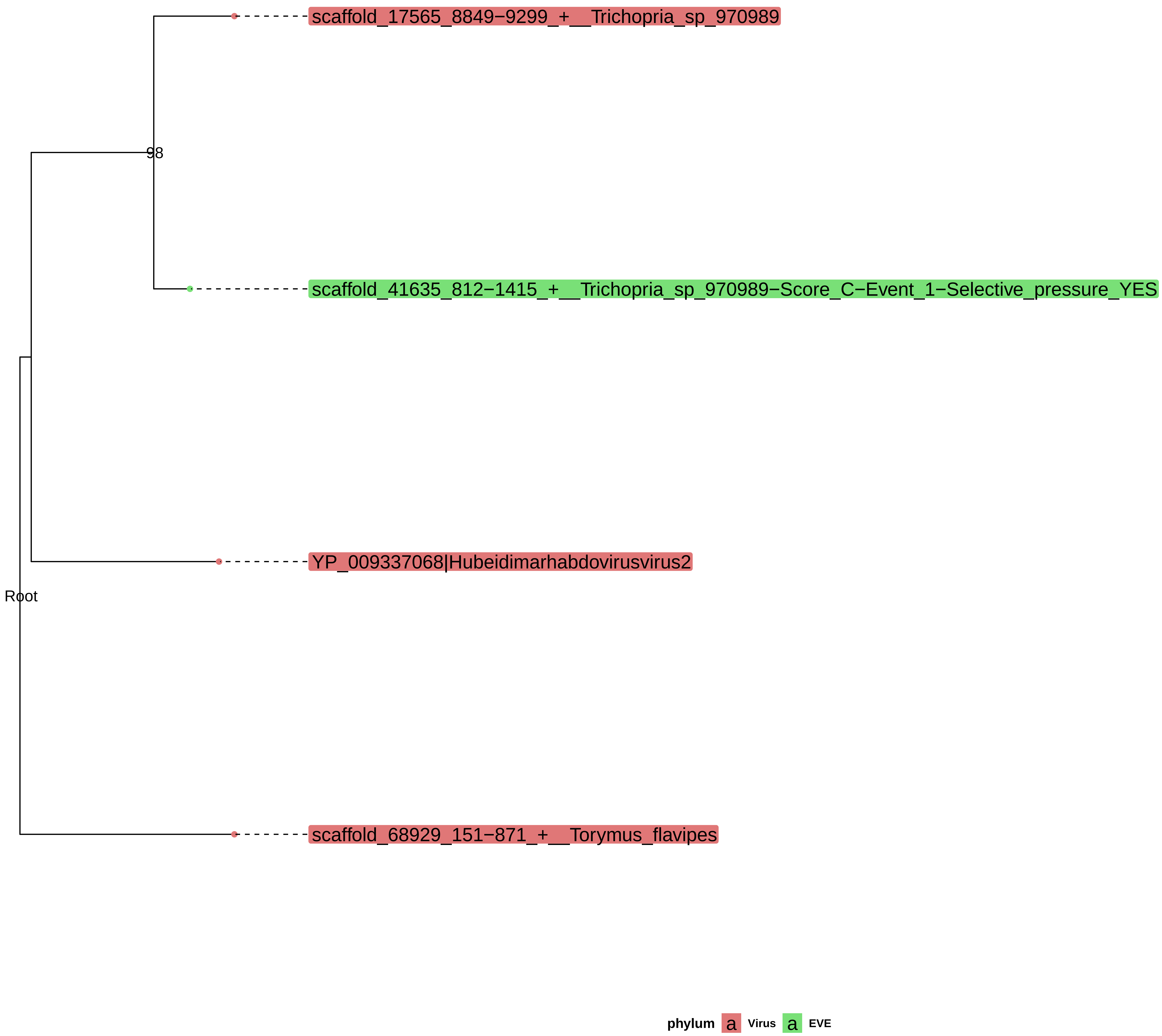

Cluster18023 − families involved : ['Nyamiviridae'] − putative protein : ['Nucleocapsid']

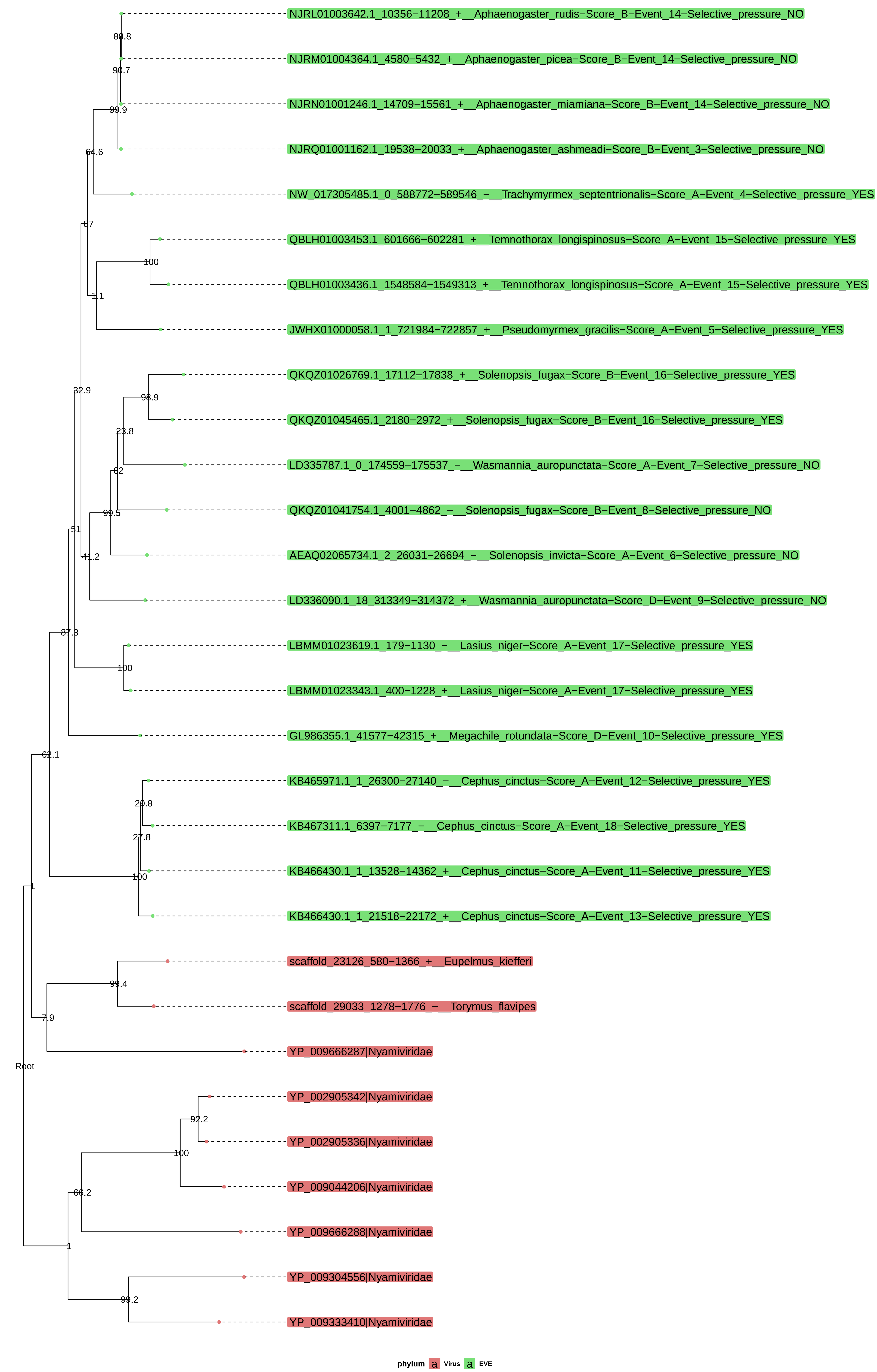

Cluster17945 − families involved : ['Iridoviridae'] − putative protein : ['Immune reactive putative protease inhibitor']

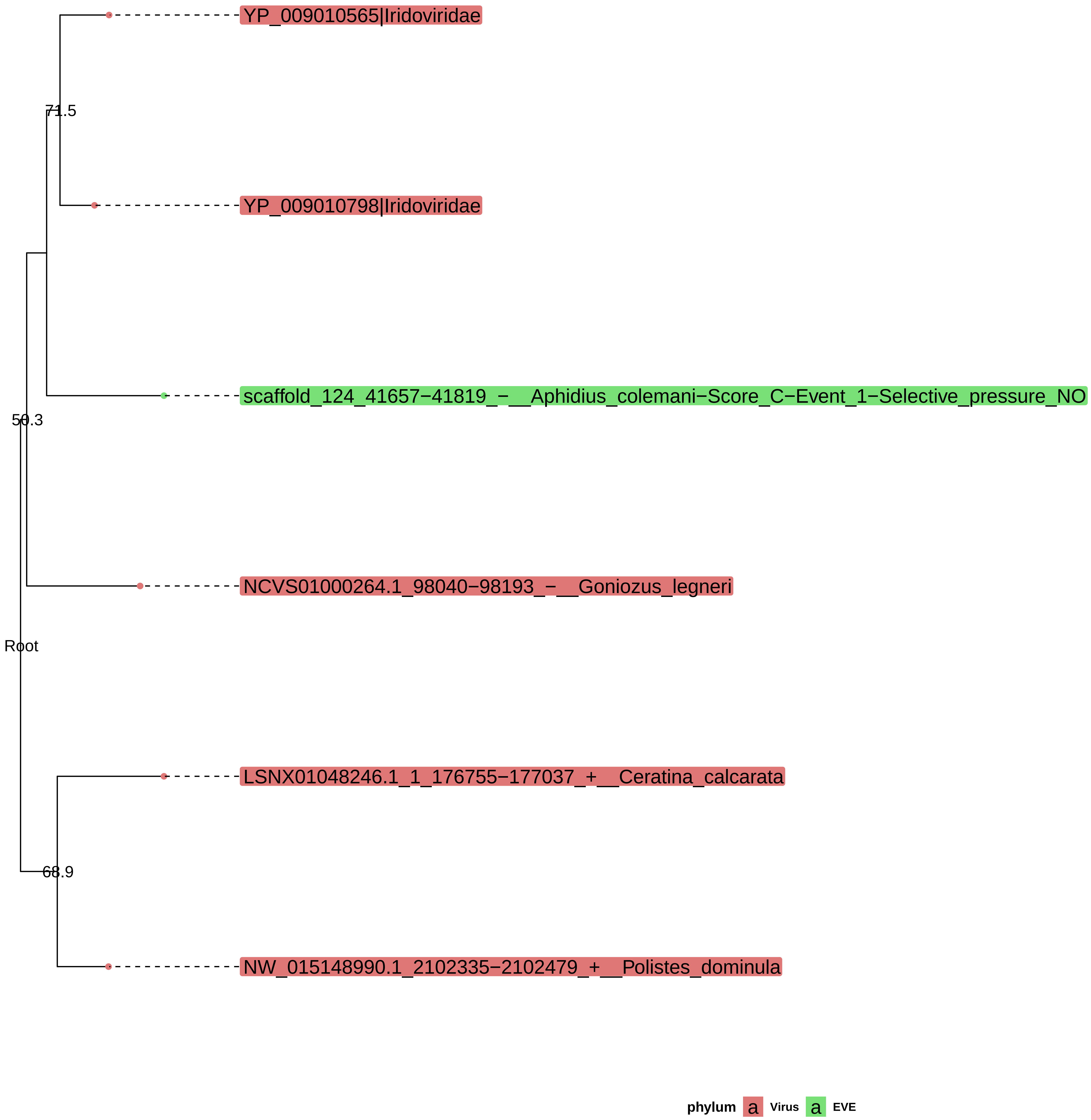

Cluster17775 − families involved : ['Baculoviridae'] − putative protein : ['p26']

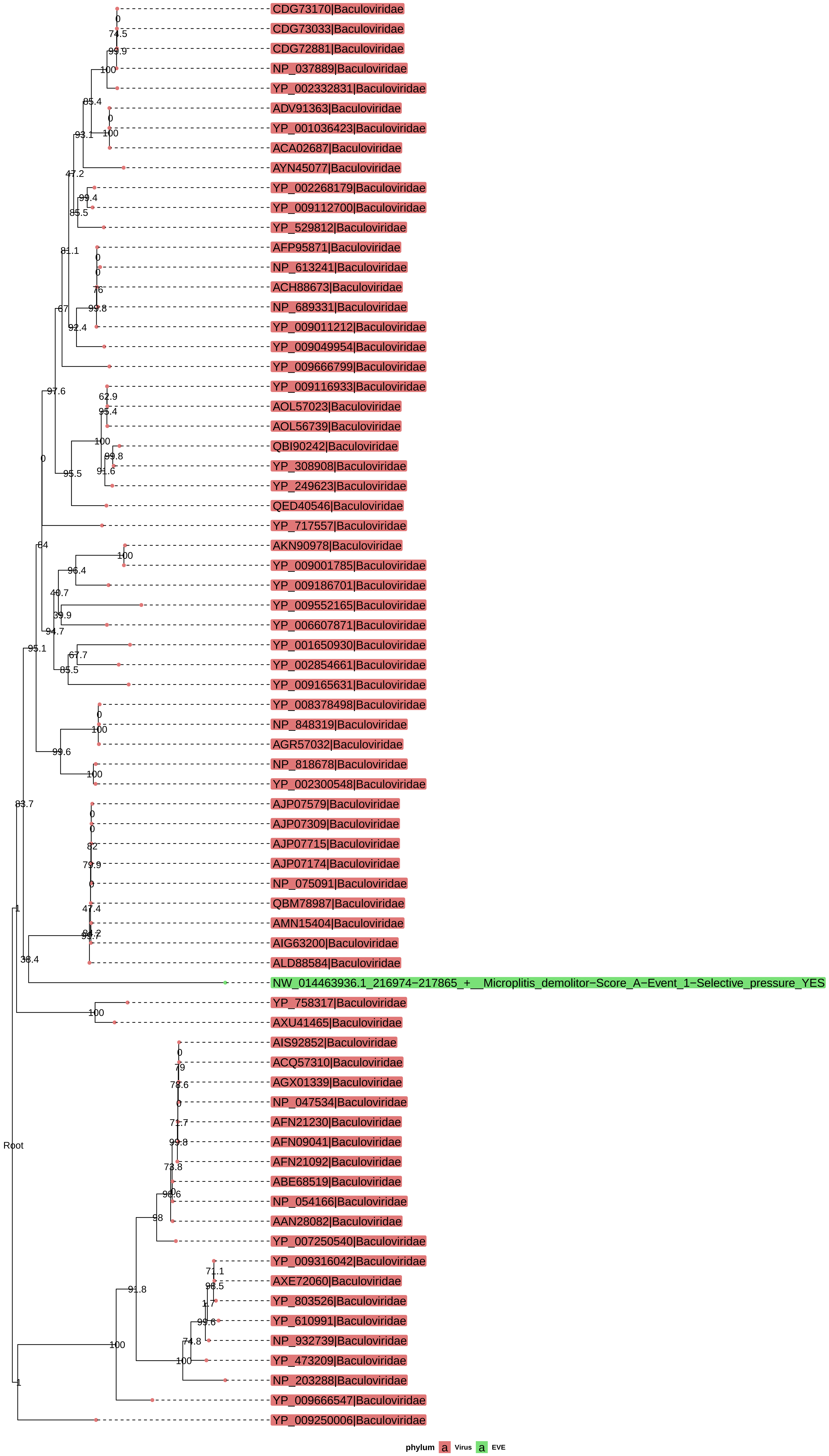

Cluster3538 − families involved : ['Apis_filamentous−like'] − putative protein : ['Borrelia outer surface protein E']

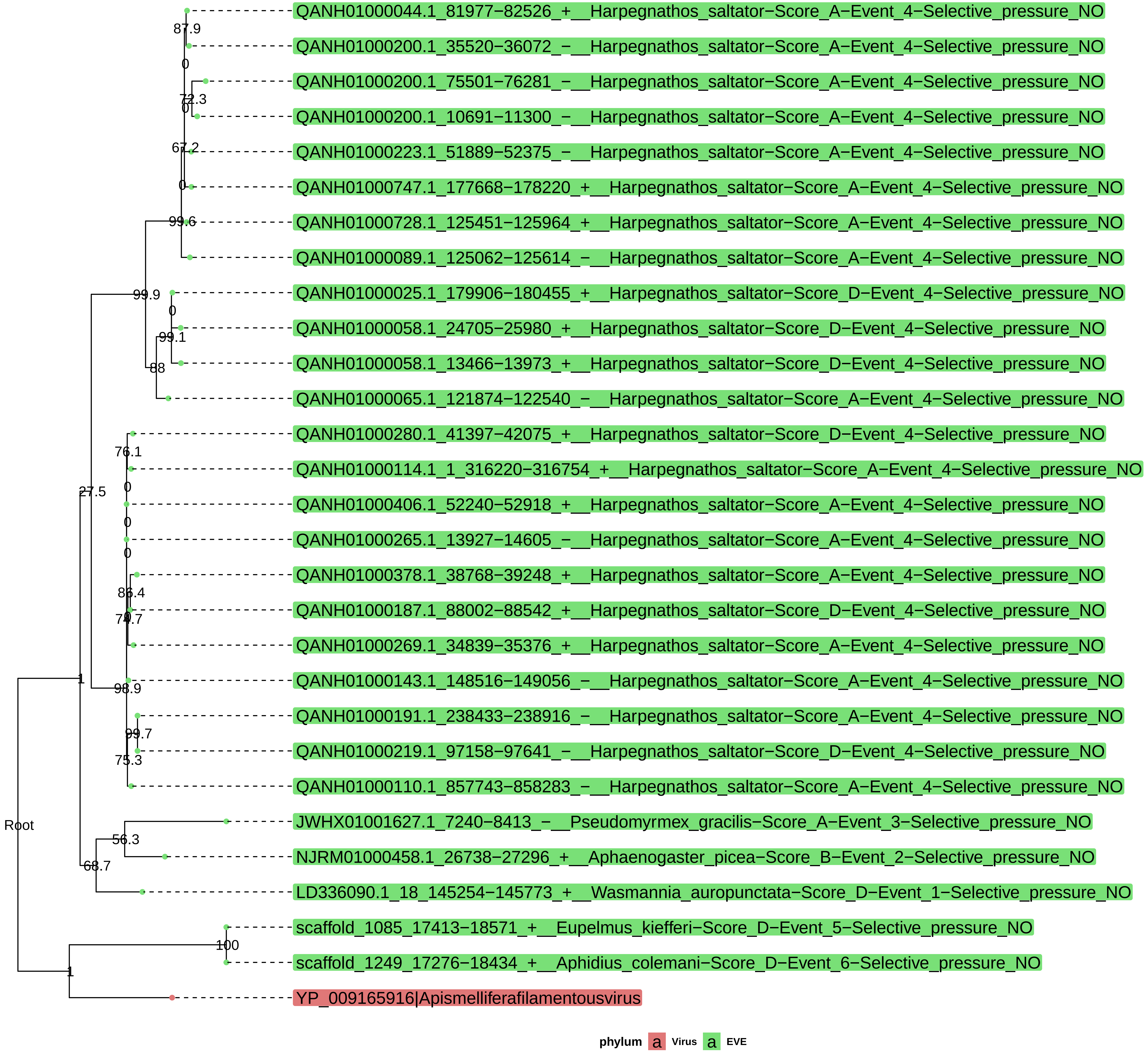

Cluster3481 − families involved : ['Apis_filamentous−like'] − putative protein : ['Virulence−associated protein E']

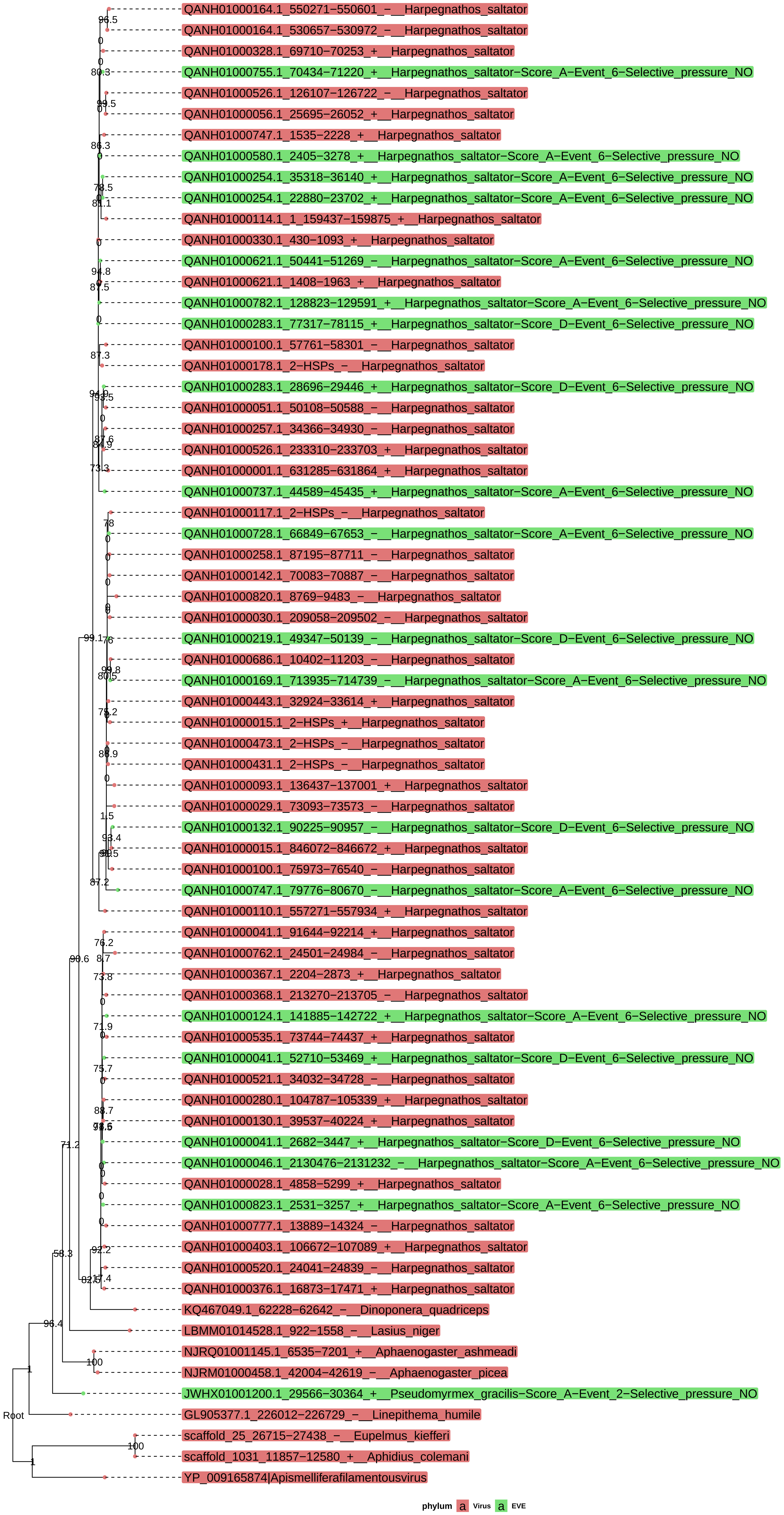

Cluster22359 − families involved : ['Apis_filamentous−like'] − putative protein : ['Protein of unknown function (DUF3847)']

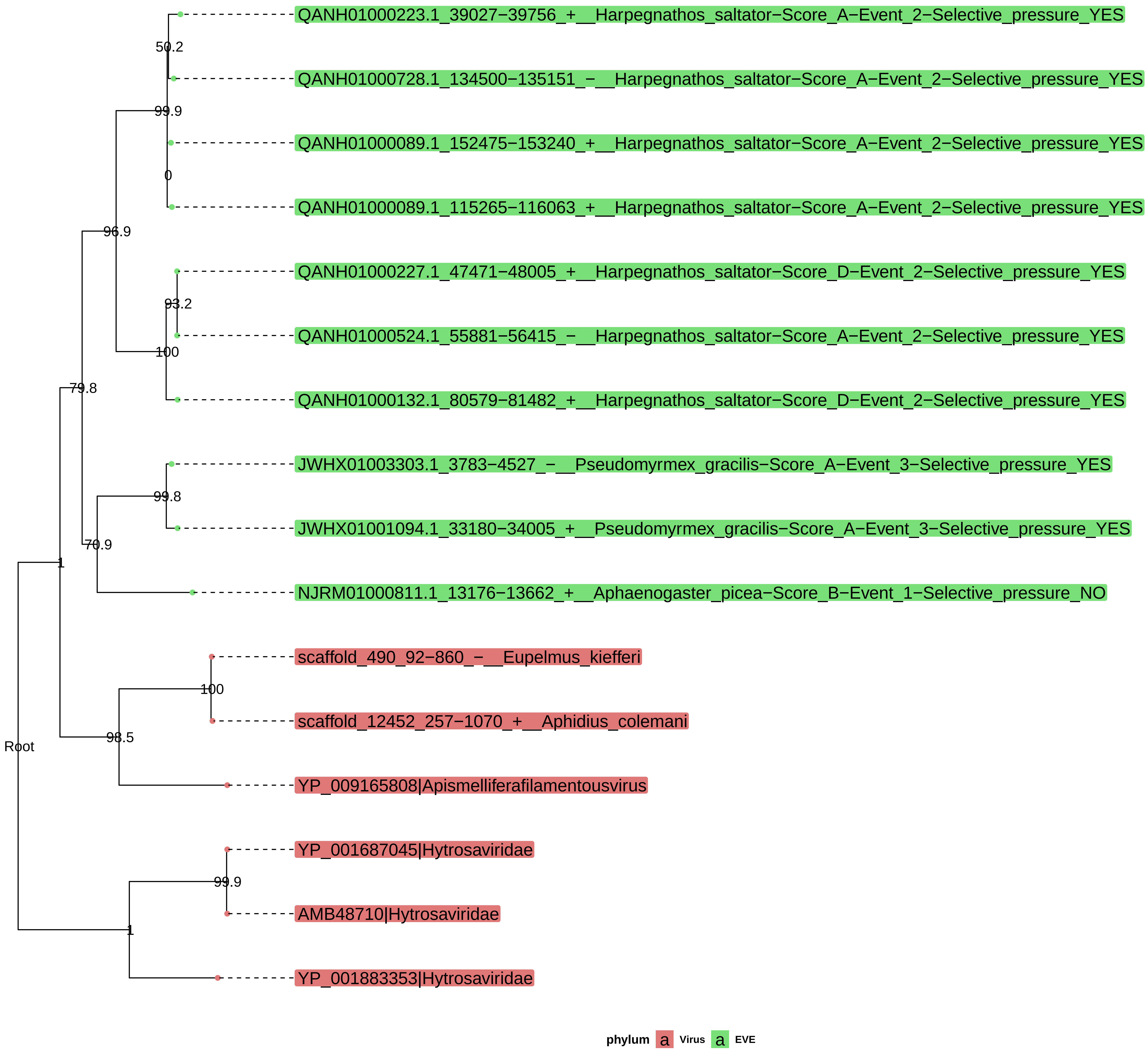

Cluster20941 − families involved : ['Apis_filamentous−like'] − putative protein : ['Methyltransferase domain']

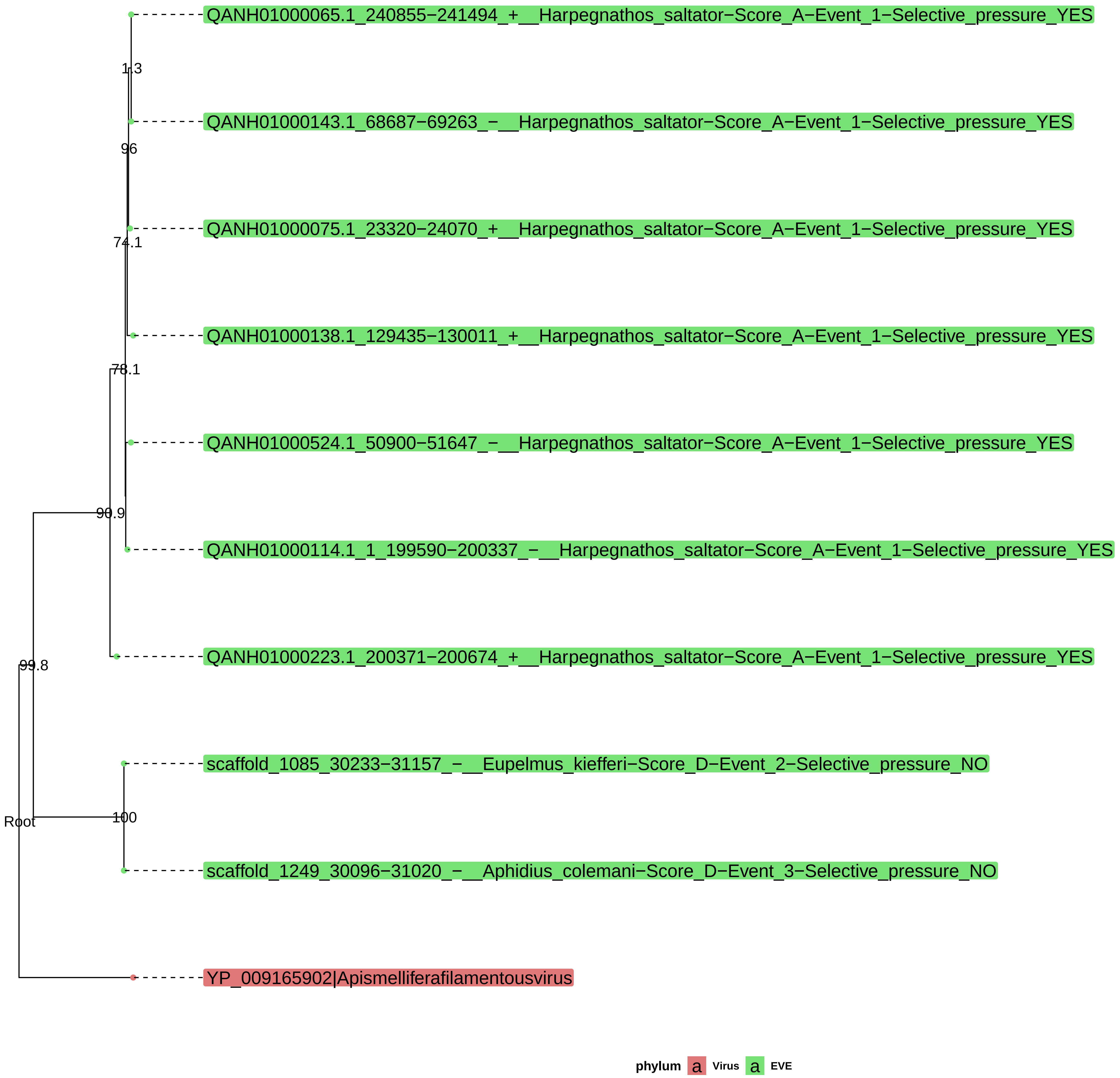

Cluster19349 − families involved : ['Apis_filamentous−like'] − putative protein : ['RNA polymerase Rpb1, domain 2']

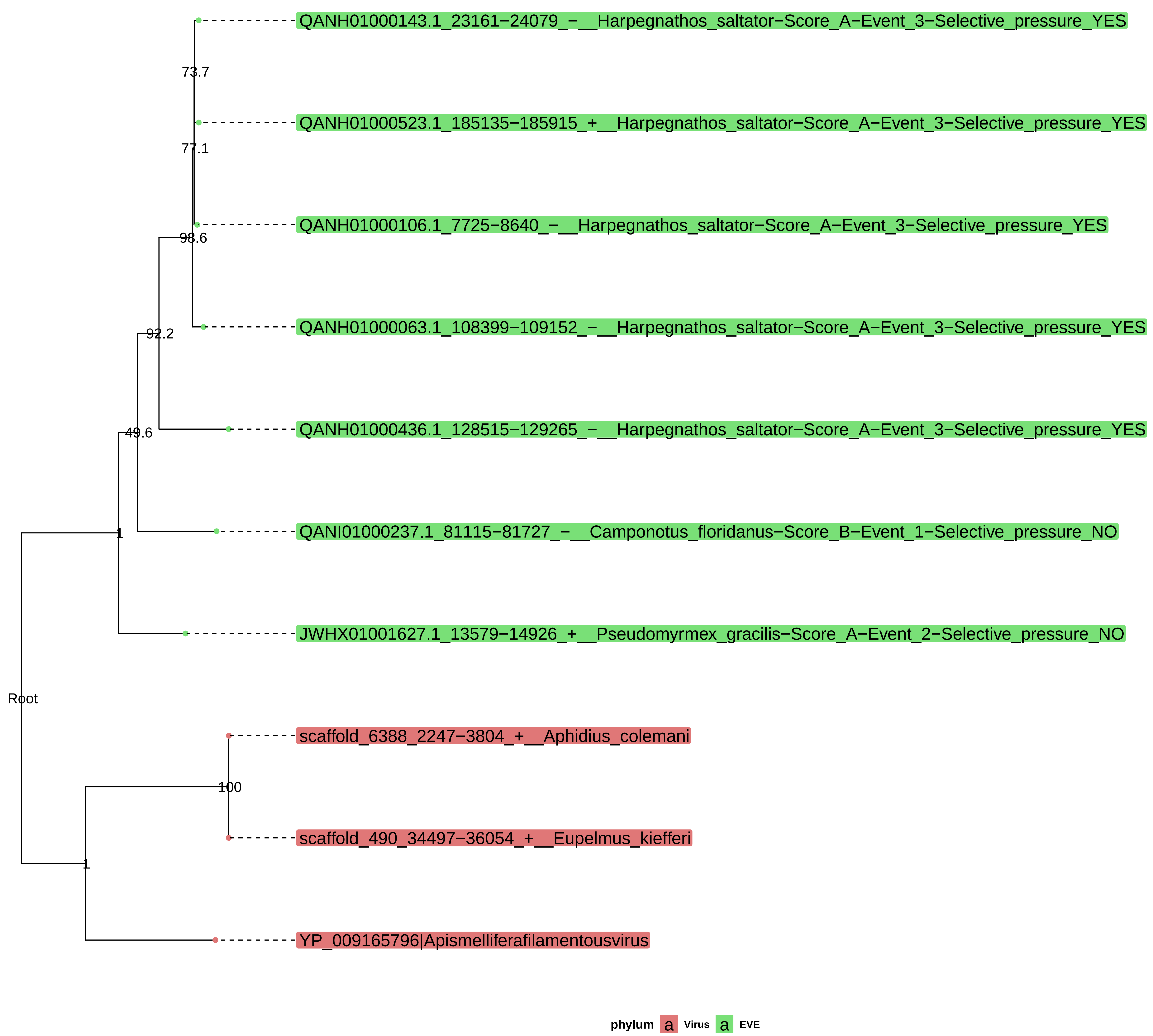

Cluster18348 − families involved : ['Apis_filamentous−like'] − putative protein : ['ATP−dependent zinc metalloprotease']

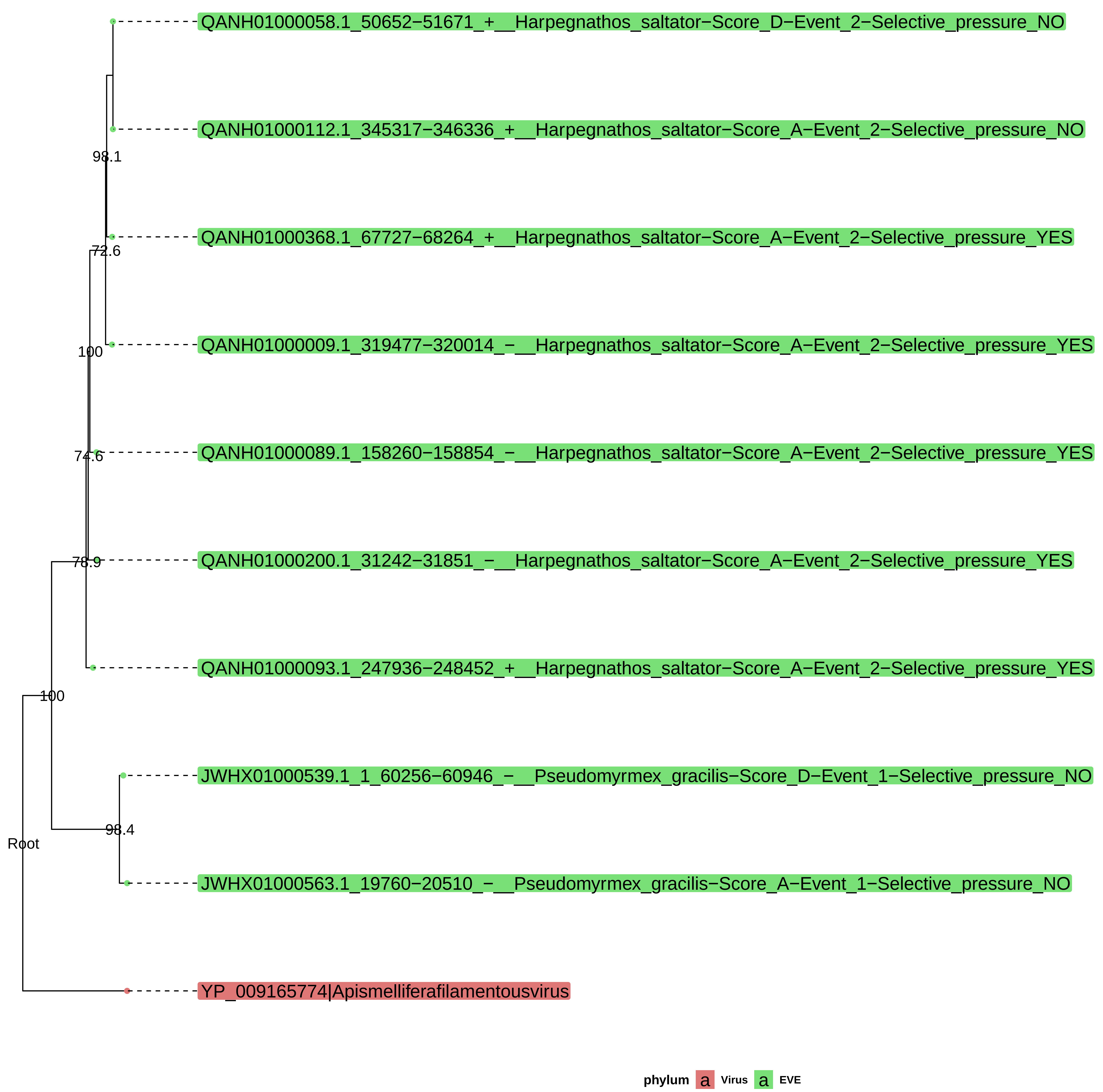

Cluster17658 − families involved : ['Orthomyxoviridae'] − putative protein : ['RNA−directed RNA polymerase catalytic subunit (EC 2.7.7.48)']

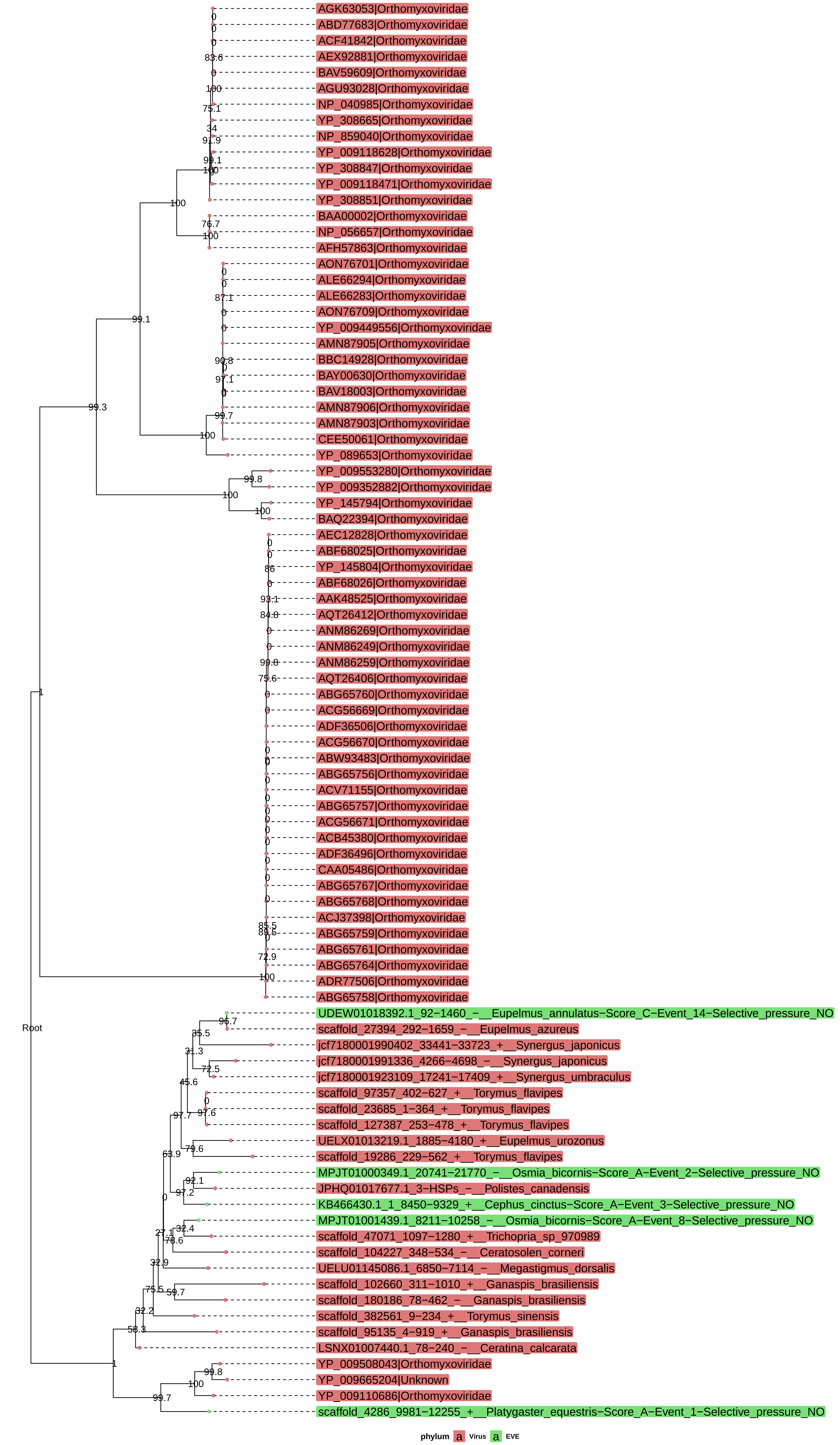

Cluster26257 − families involved : ['Poxviridae'] − putative protein : ['AAA domain']

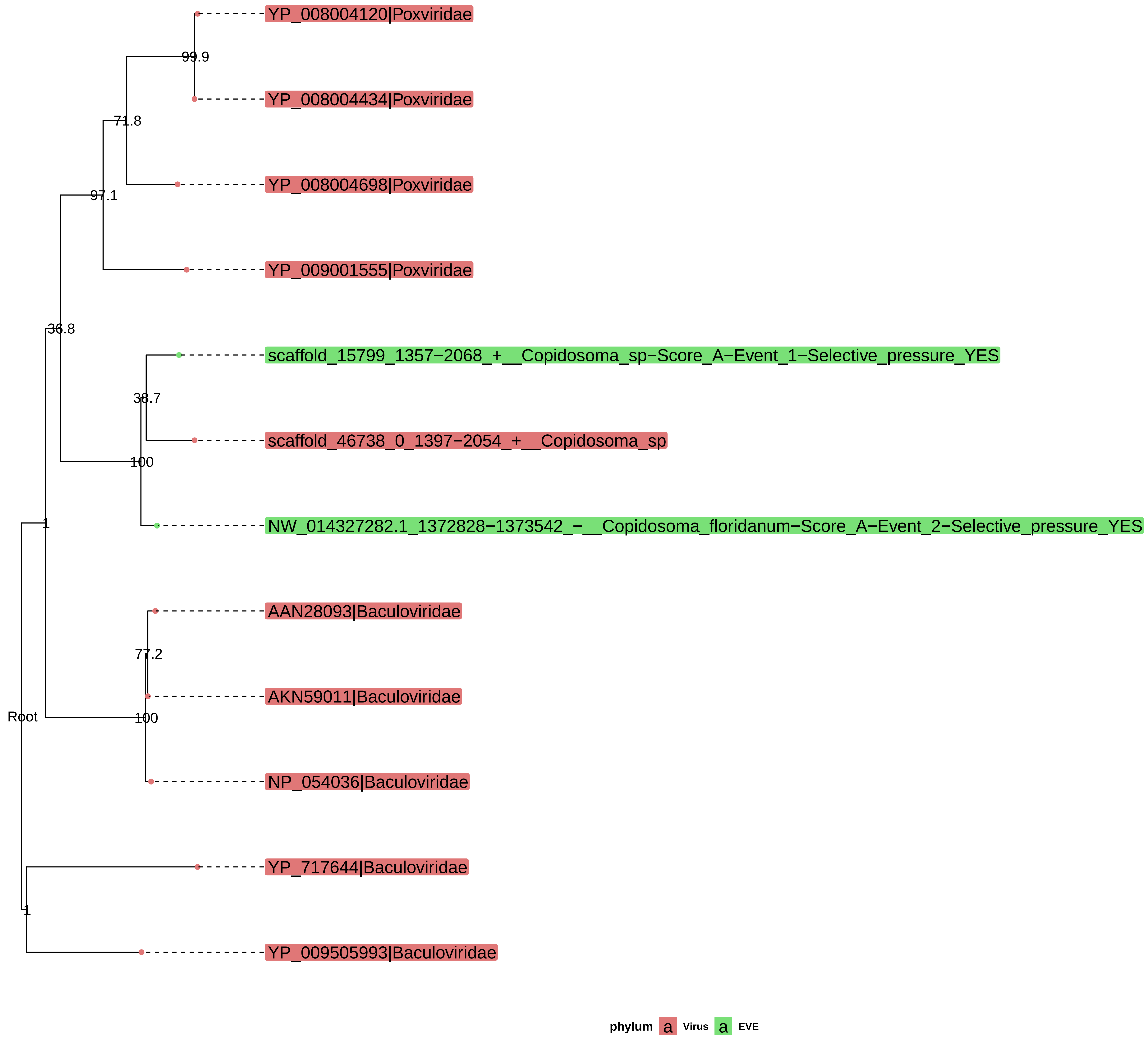

Cluster1758 − families involved : ['Poxviridae'] − putative protein : ['Calmodulin binding domain']

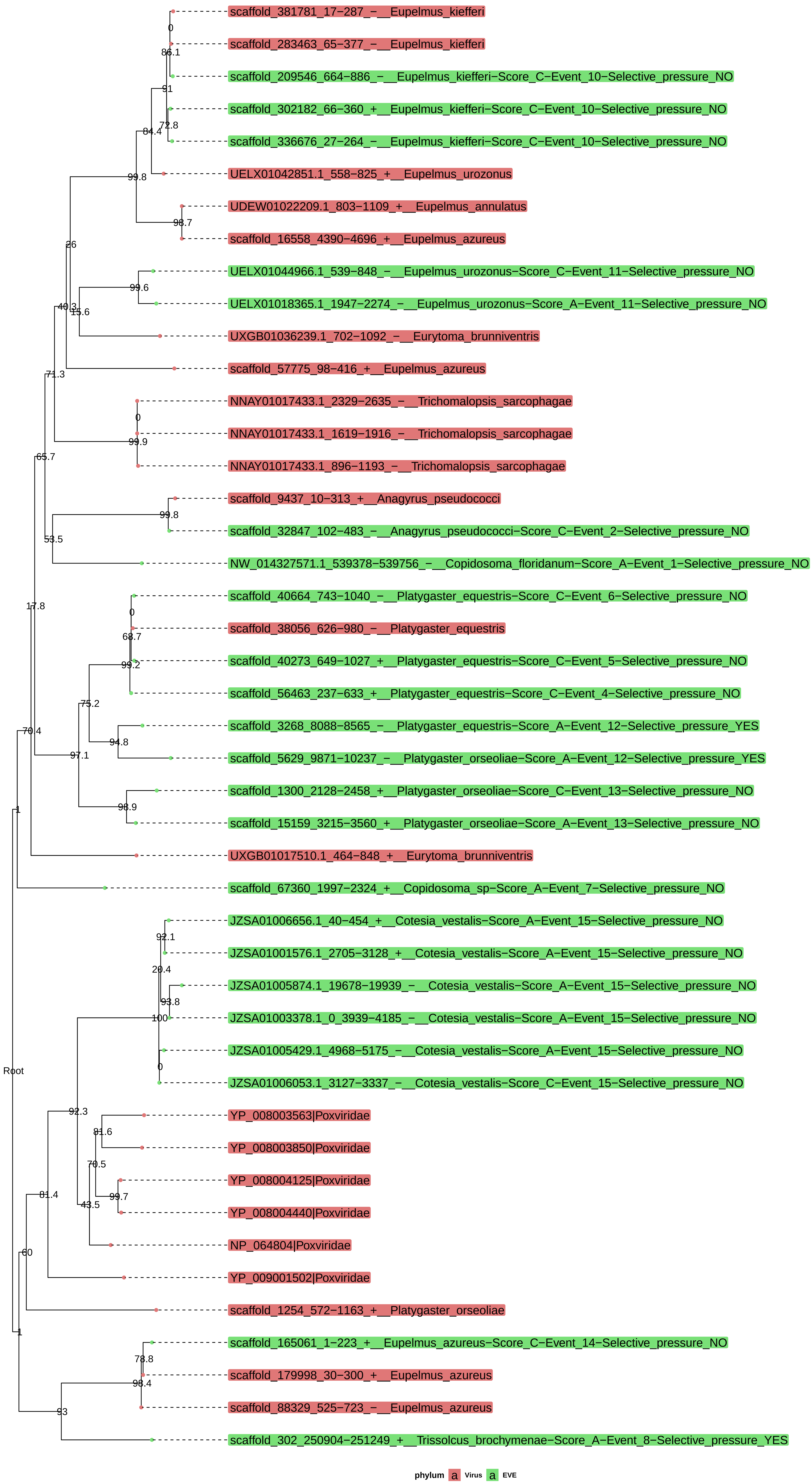

Cluster1757 − families involved : ['Unknown'] − putative protein : ['NS5−like protein']

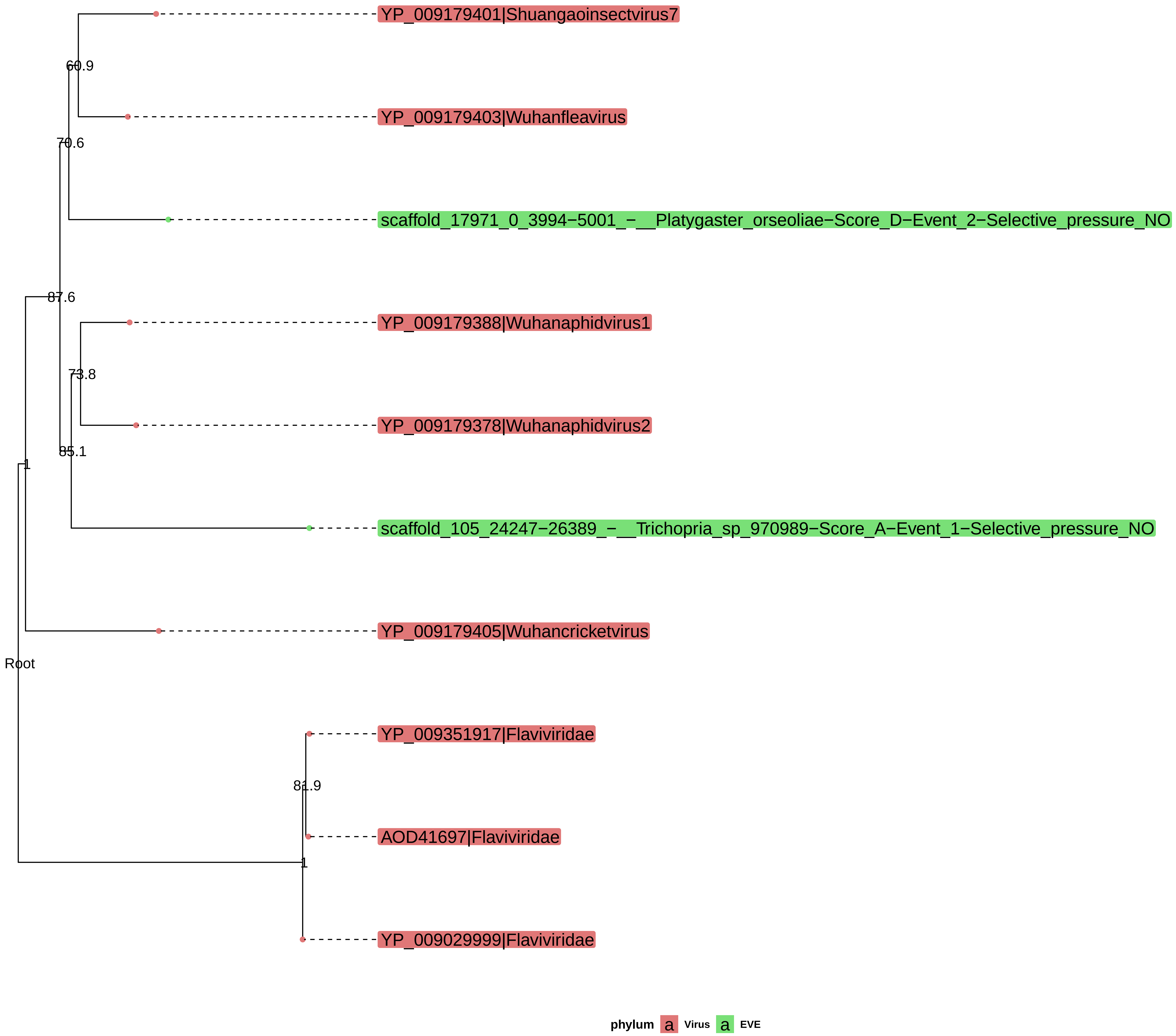

Cluster17557 − families involved : ['Iridoviridae'] − putative protein : ['Uncharacterized protein']

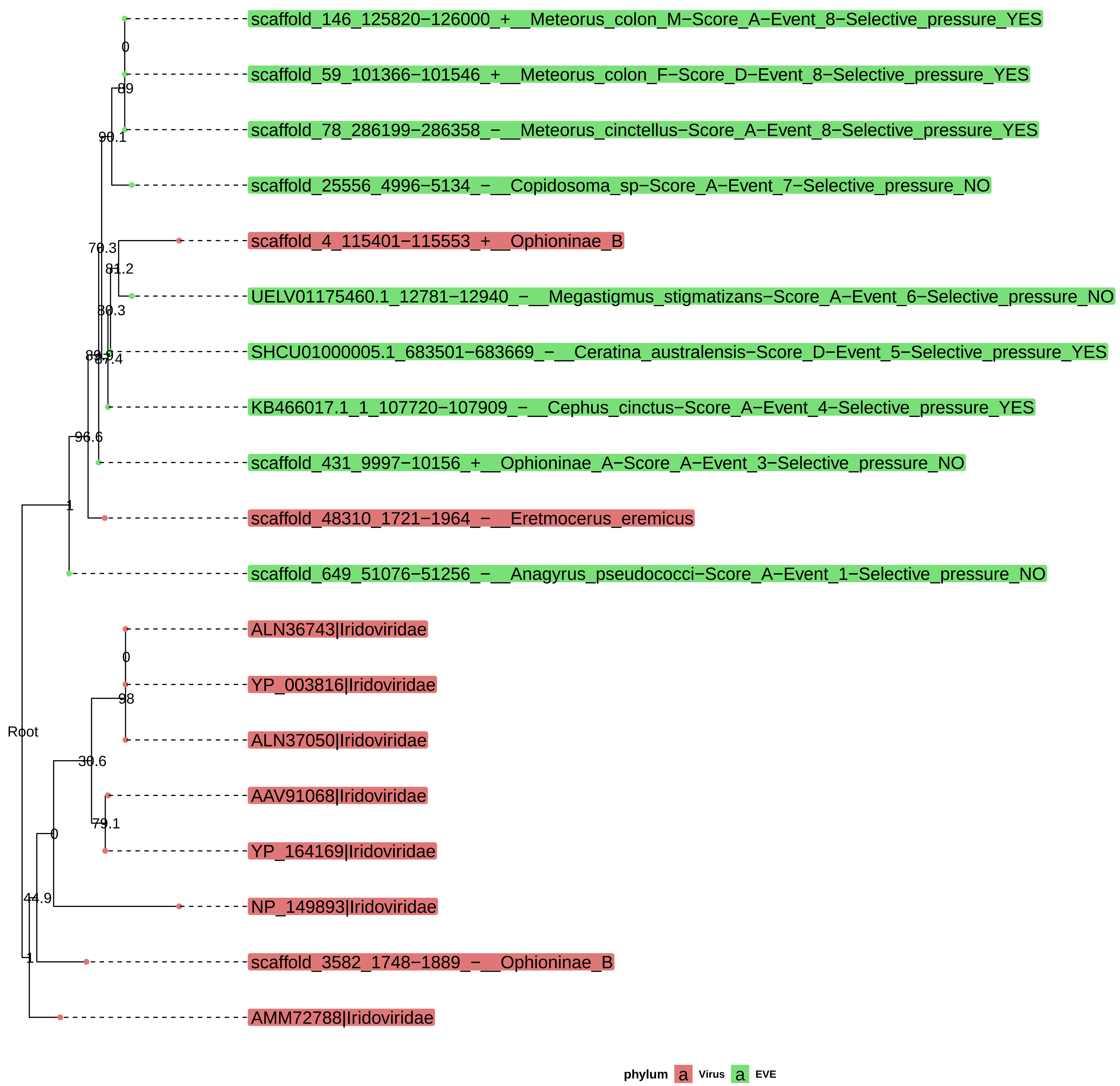

Cluster22895 − families involved : ['Phycodnaviridae'] − putative protein : ['Uncharacterized protein m550L']

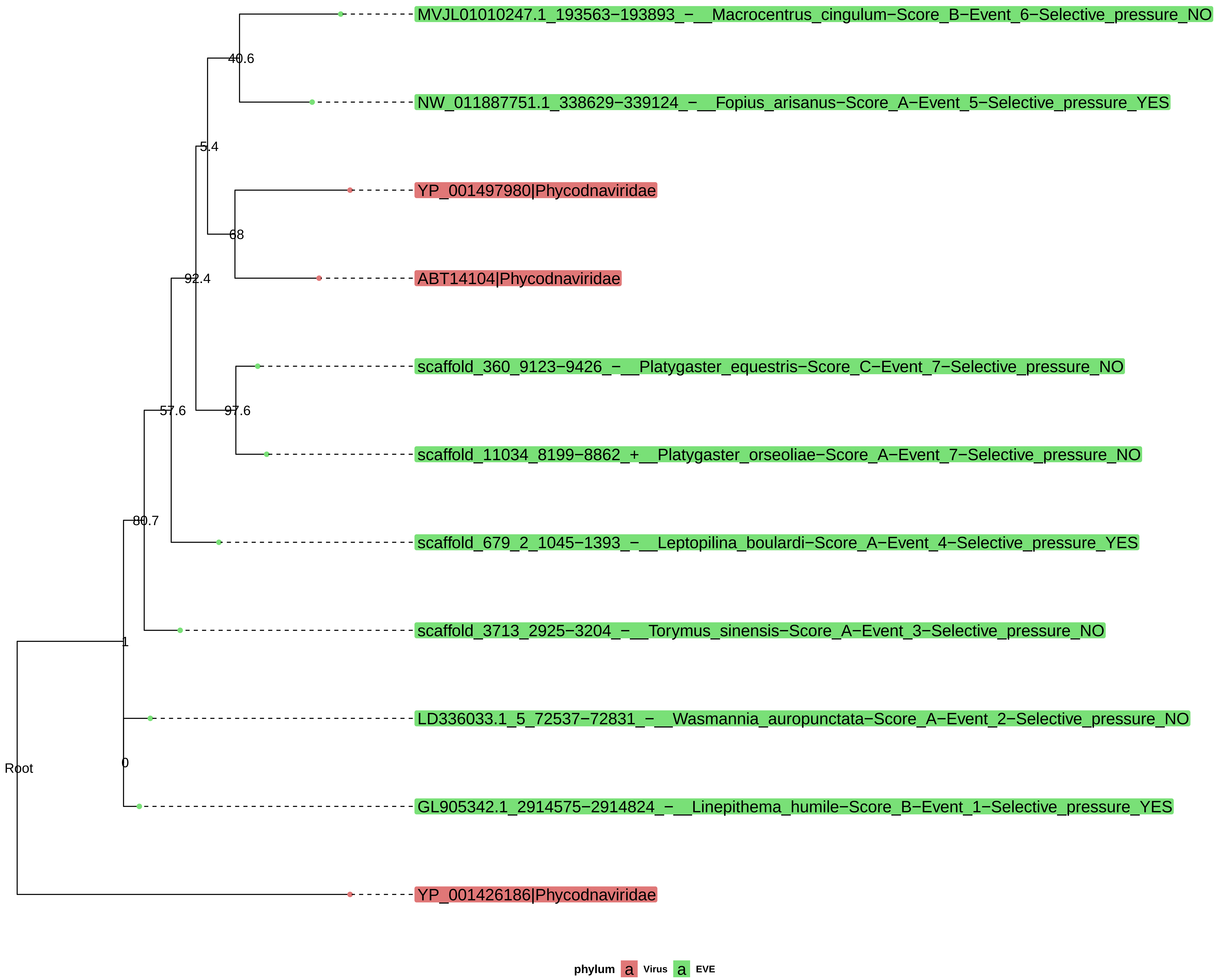

Cluster17518 − families involved : ['Phycodnaviridae'] − putative protein : ['Uncharacterized protein m778R']

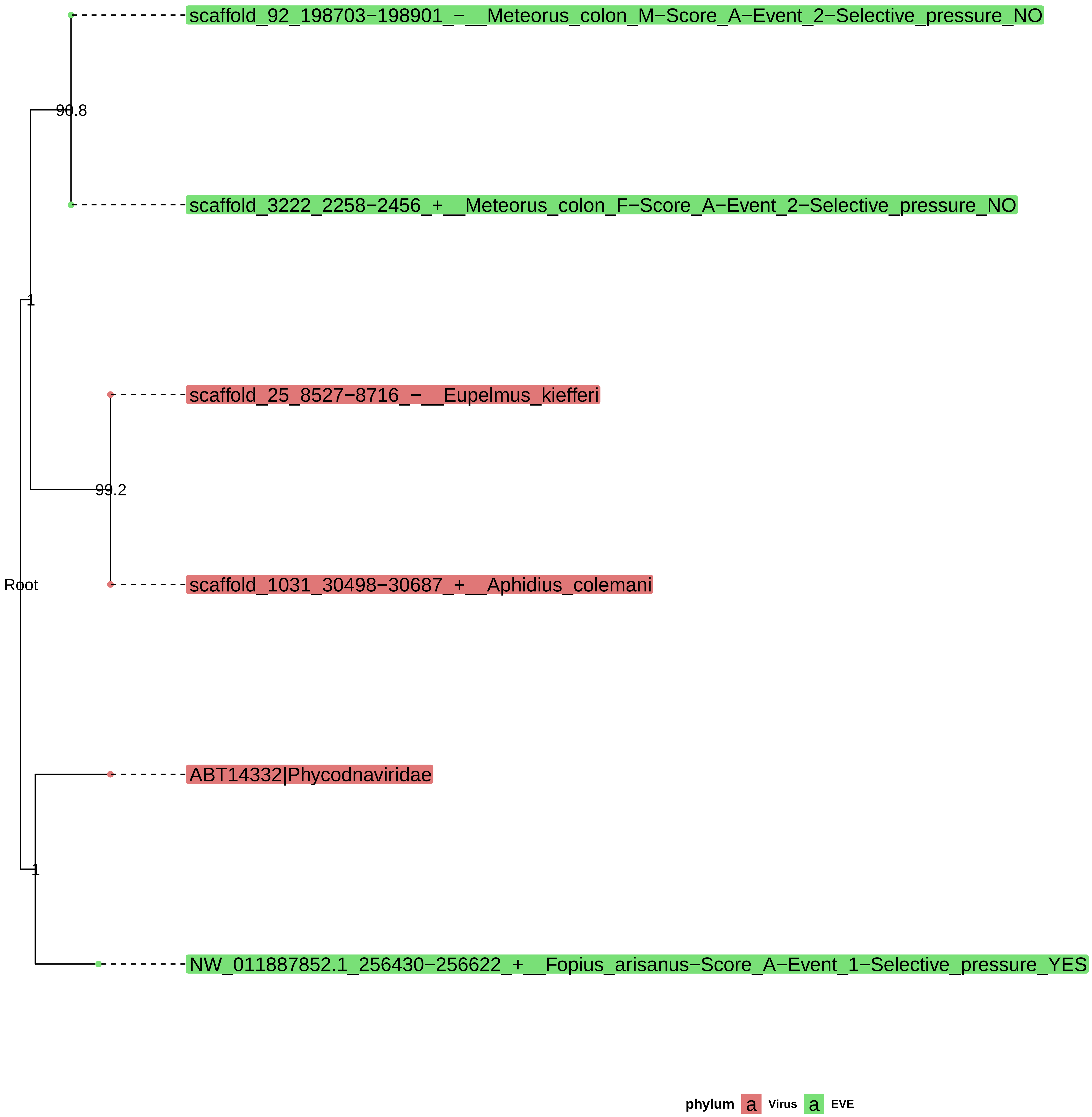

Cluster25892 − families involved : ['Orthomyxoviridae'] − putative protein : ['Polymerase P3']

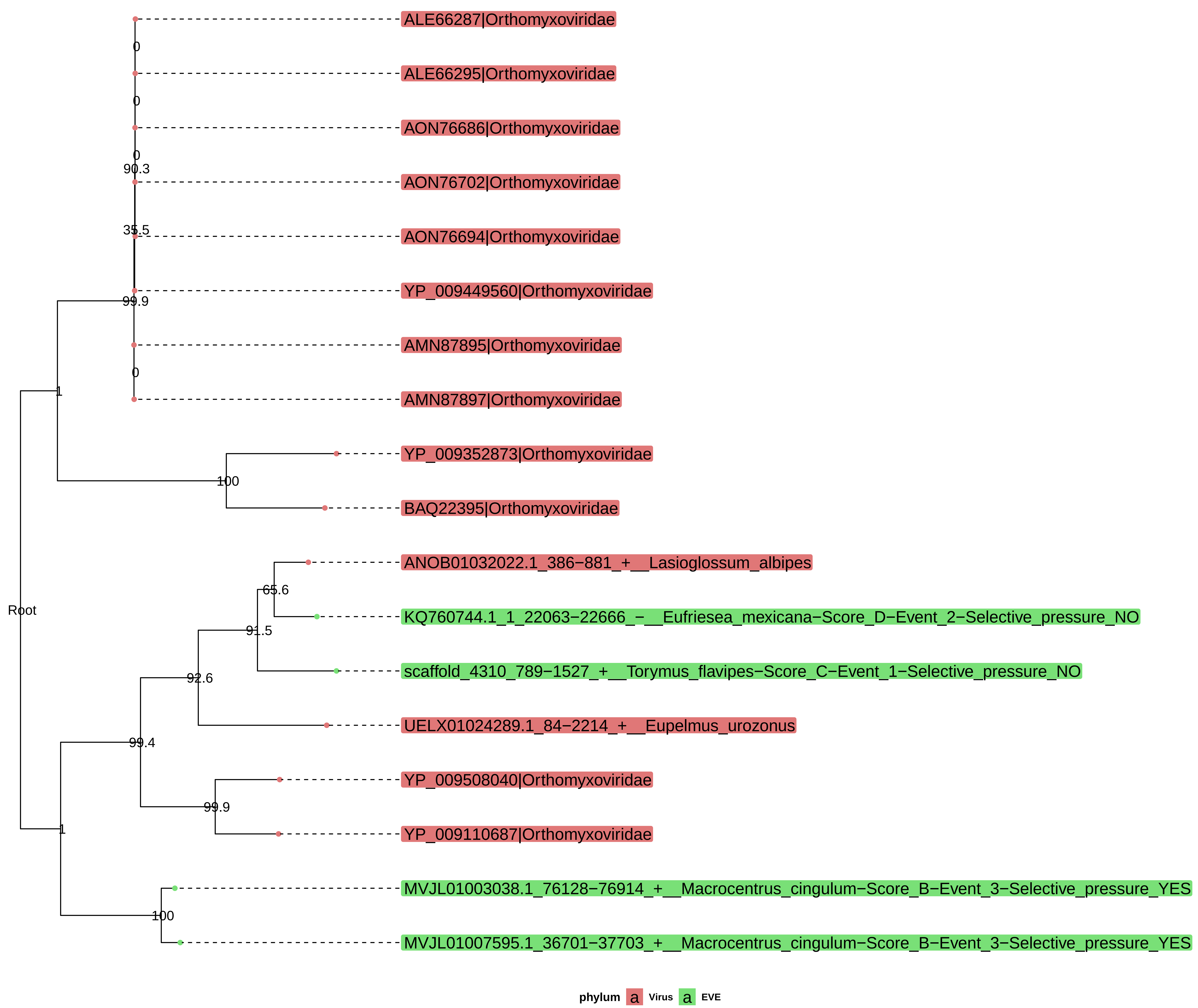

Cluster23103 − families involved : ['Negevirus−like'] − putative protein : ['Putative virion membrane protein of plant and insect virus']

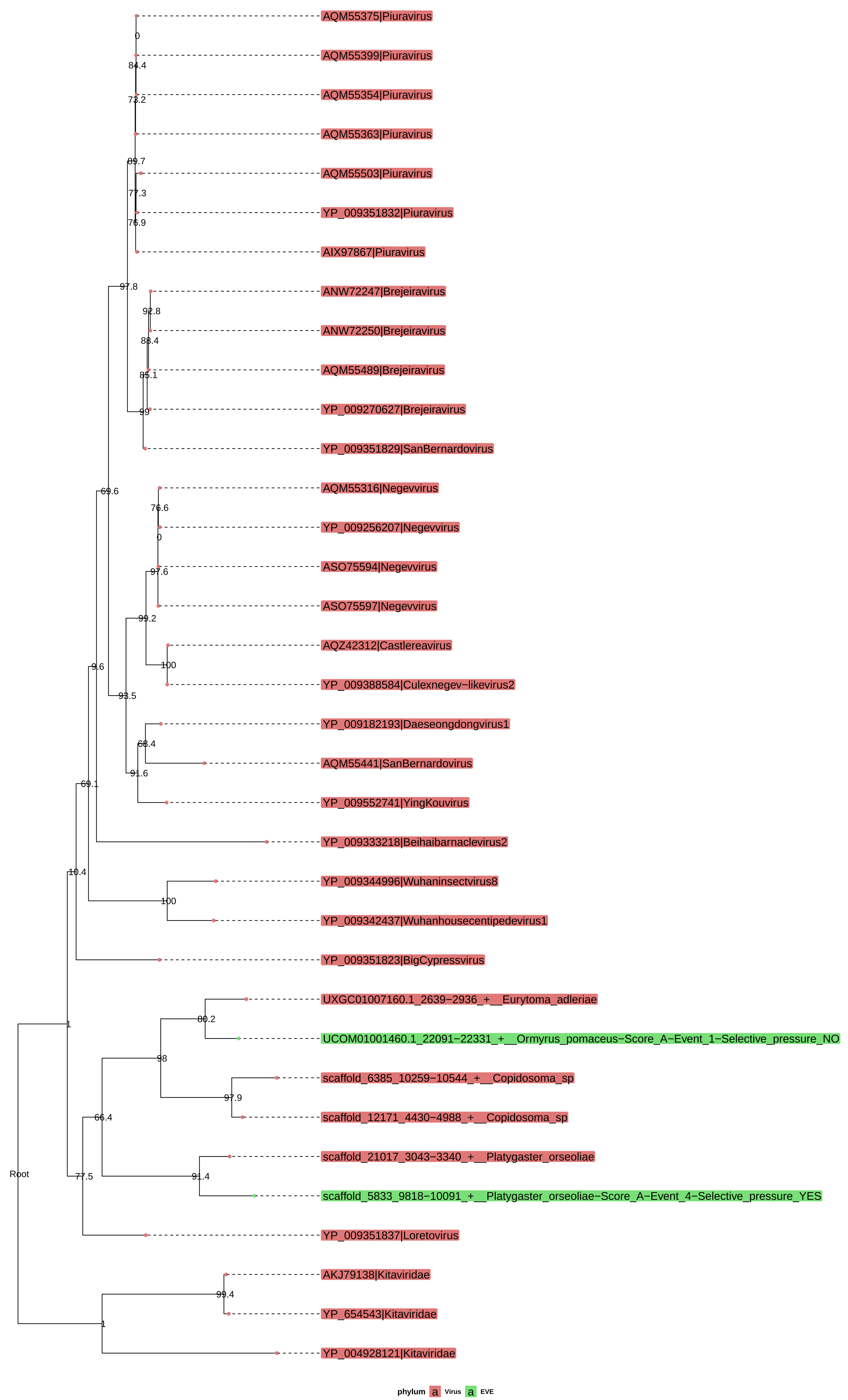

Cluster17514 − families involved : ['Orthomyxoviridae'] − putative protein : ['Nucleoprotein']

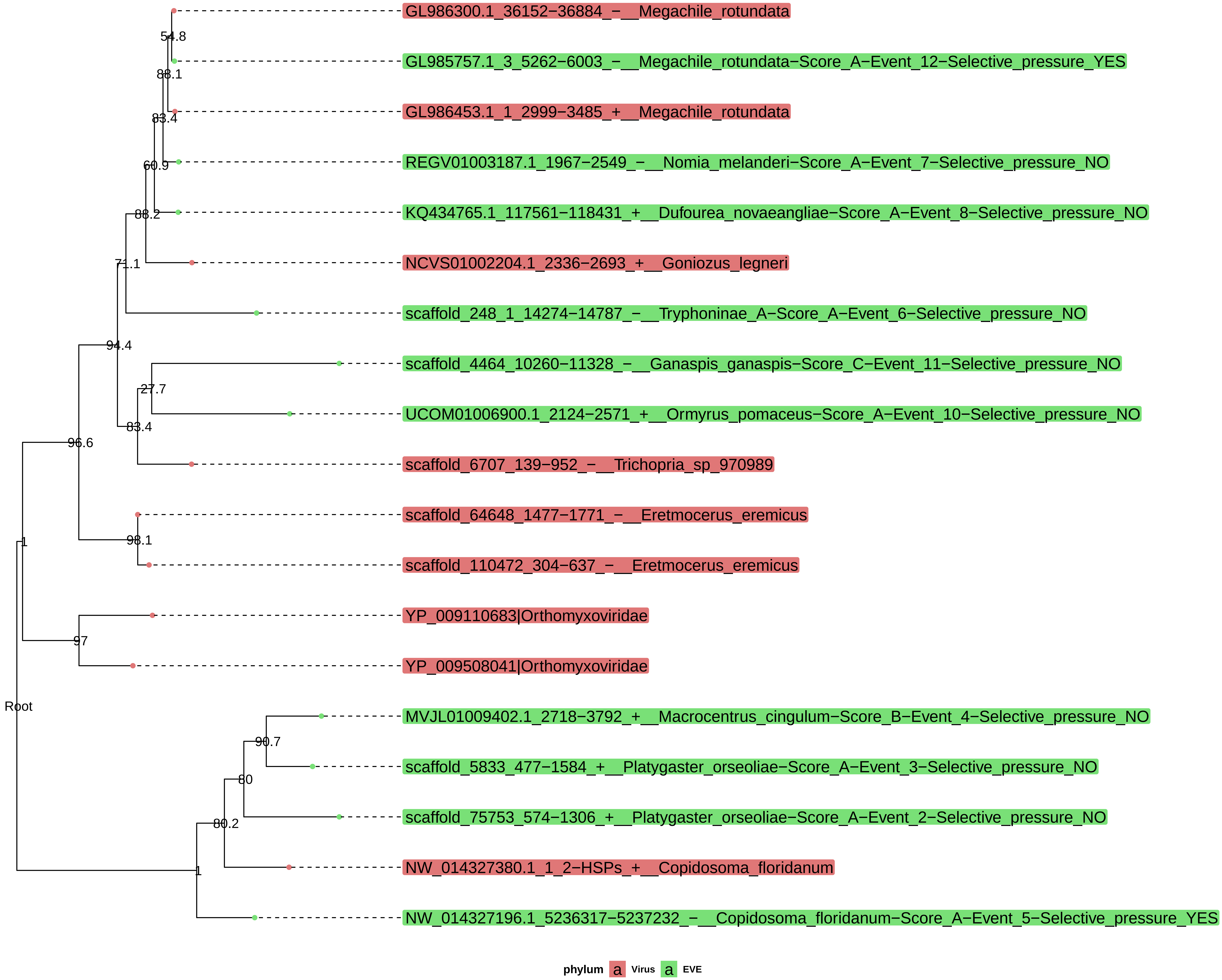

Cluster201 − families involved : ['Nudiviridae'] − putative protein : ['XPG I−region']

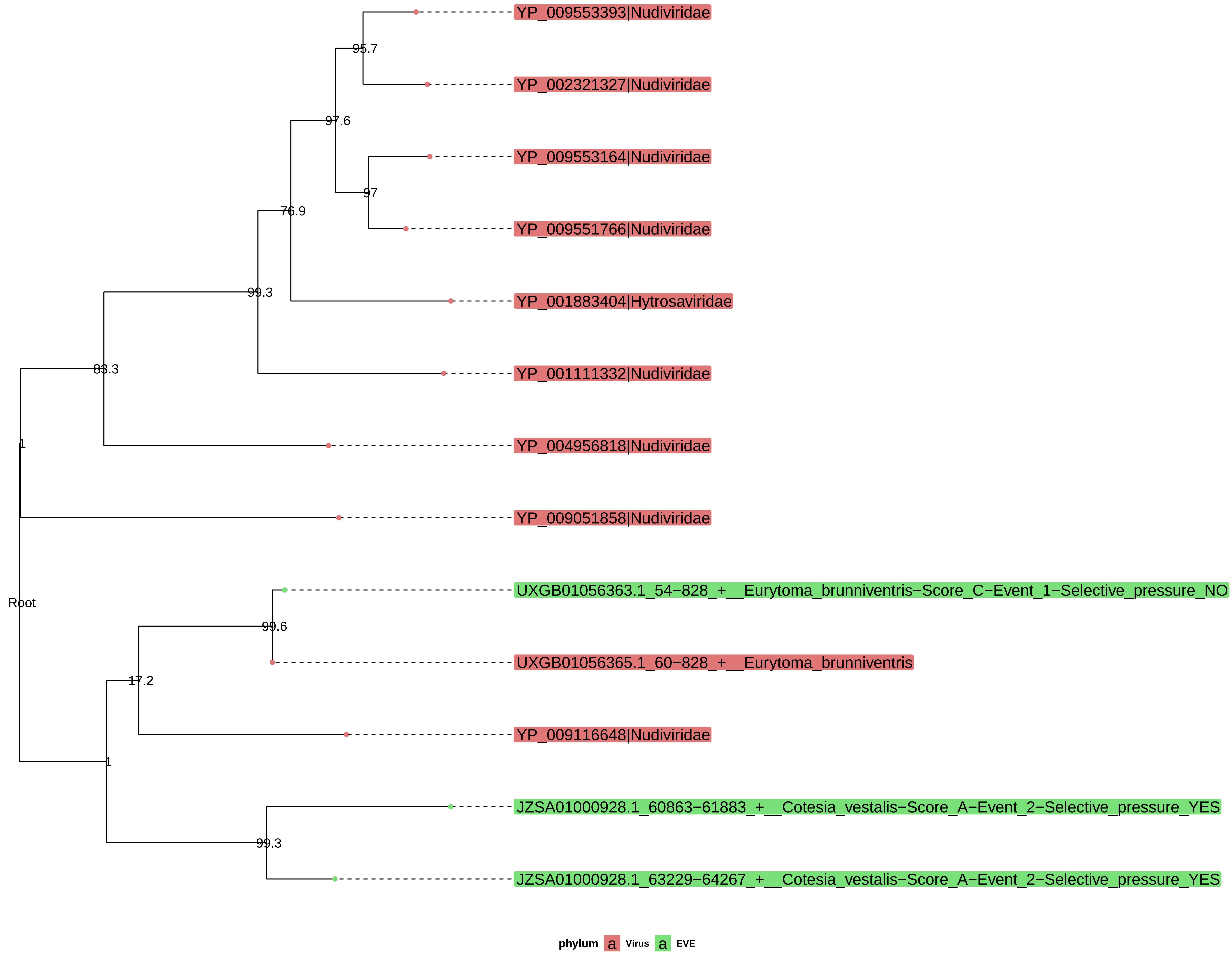

Cluster17266 − families involved : ['Iridoviridae'] − putative protein : ['Seminal vesicle protein repeat']

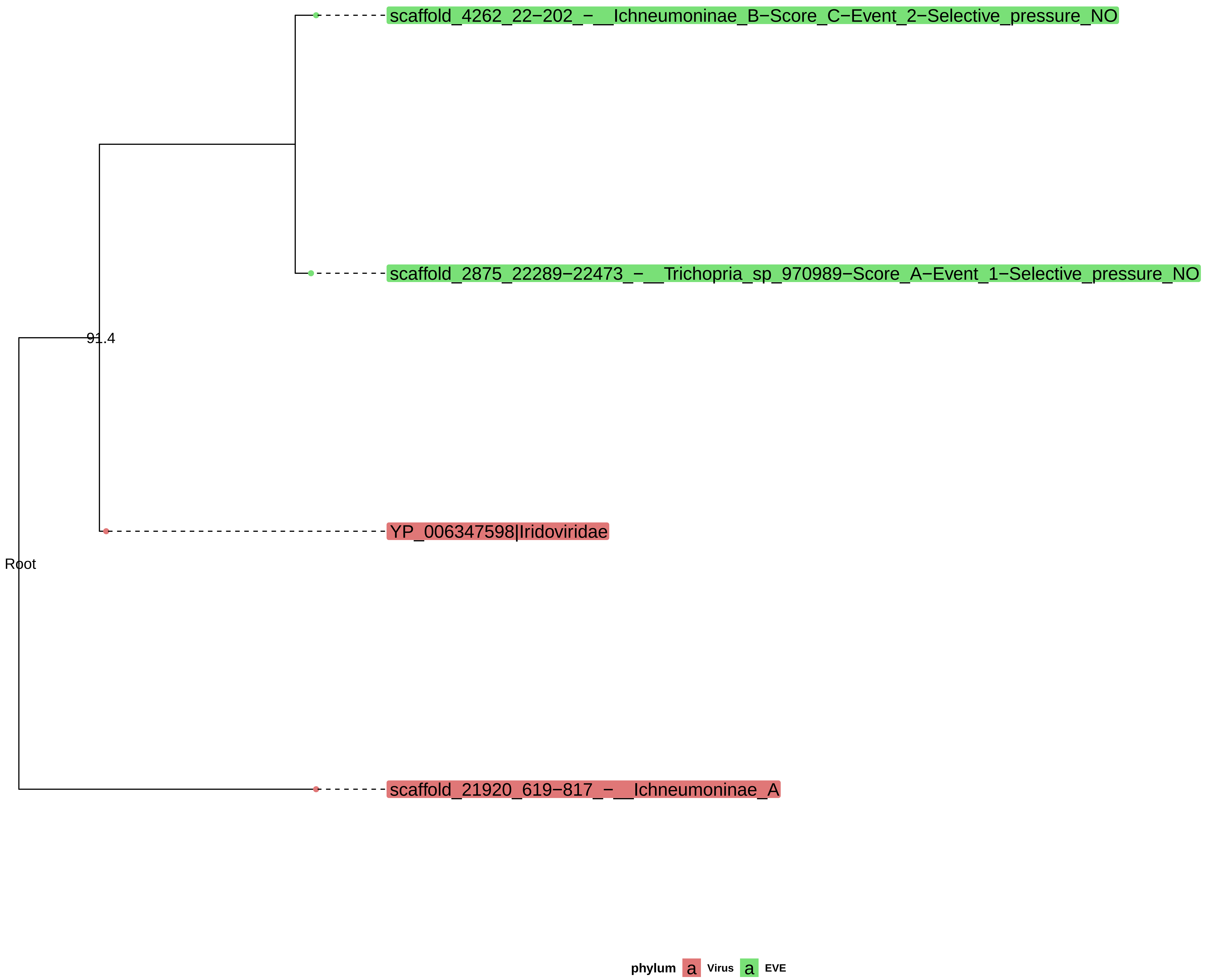

Cluster17265 − families involved : ['Partitiviridae'] − putative protein : ['Capsid protein']

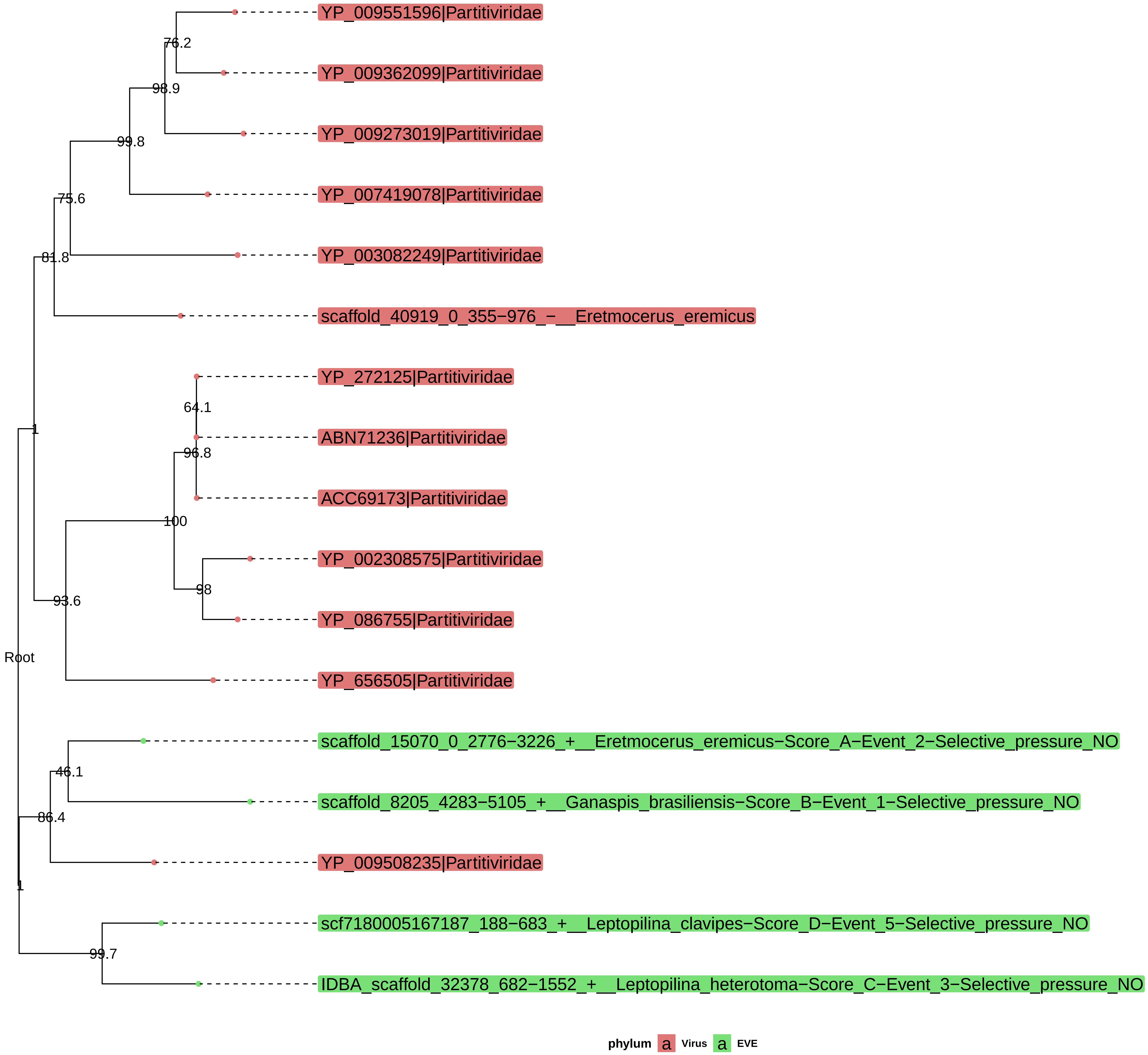

Cluster27049 − families involved : ['Parvoviridae'] − putative protein : ['Structural protein VP1']

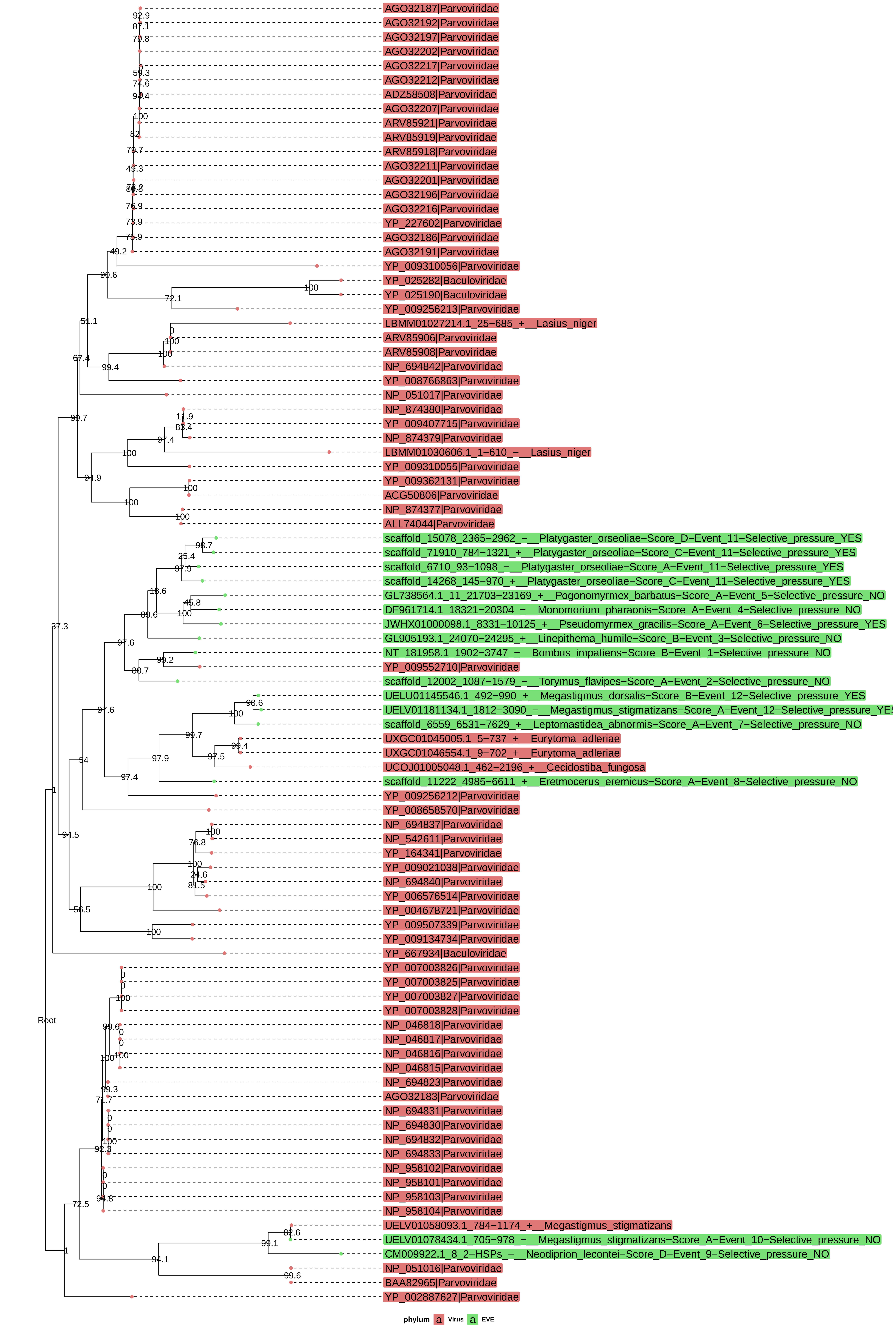

Cluster22122 − families involved : ['Parvoviridae'] − putative protein : ['Nonstructural protein 1']

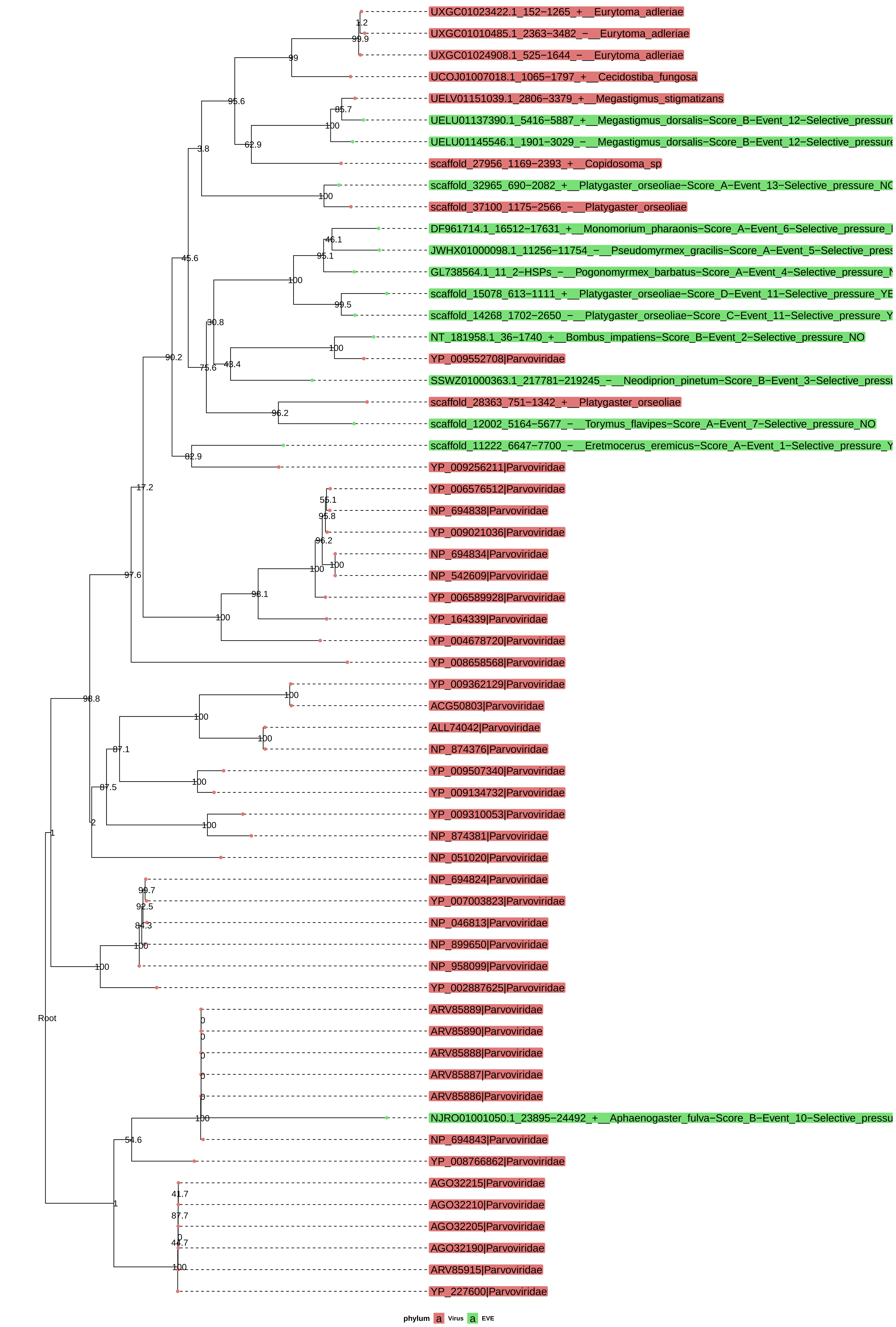

Cluster16868 − families involved : ['Chuviridae', nan] − putative protein : ['Predicted integral membrane zinc−ribbon metal−binding protein', nan]

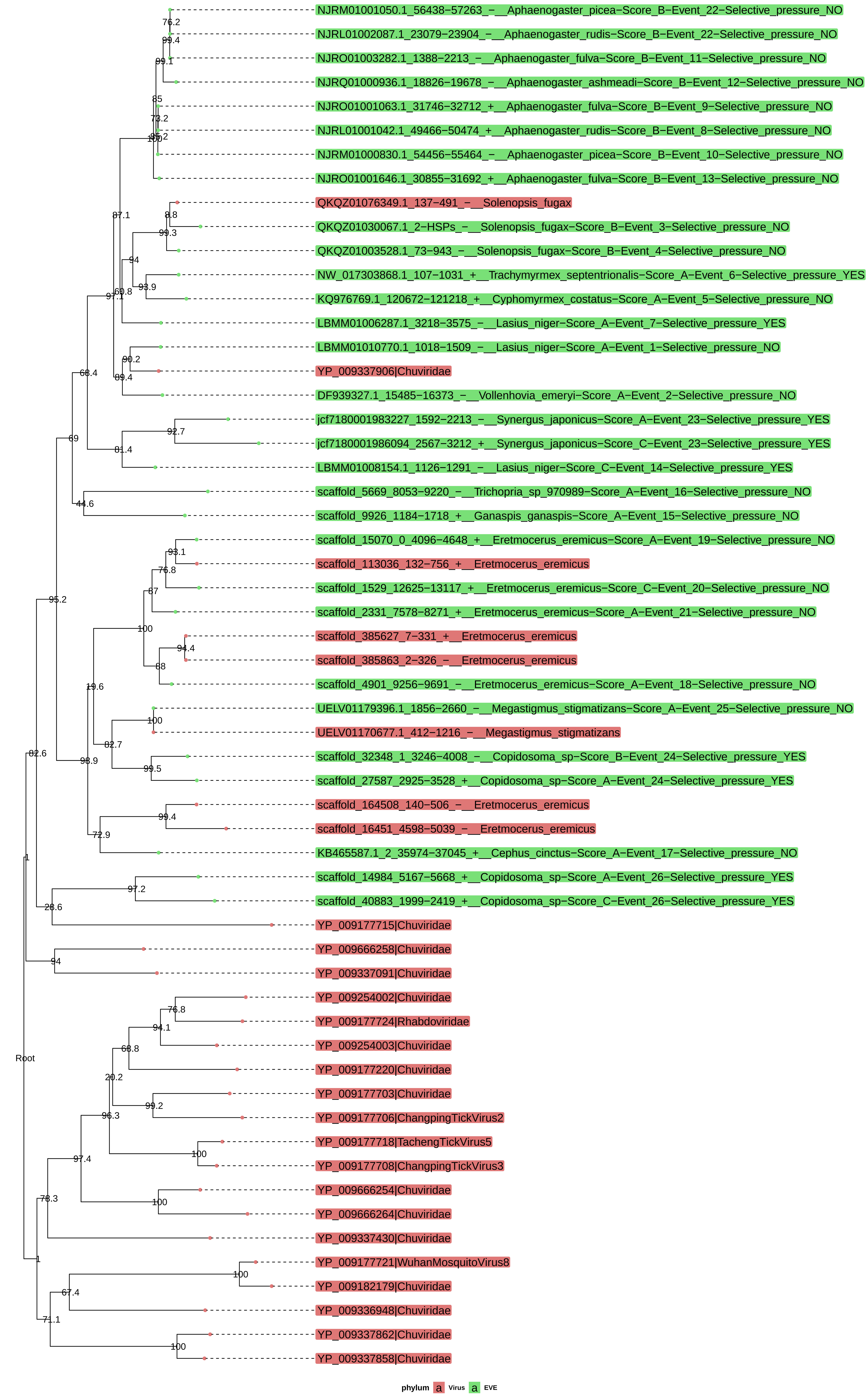

Cluster16096 − families involved : ['Poxviridae'] − putative protein : ['Viral late gene transcription factor 3']

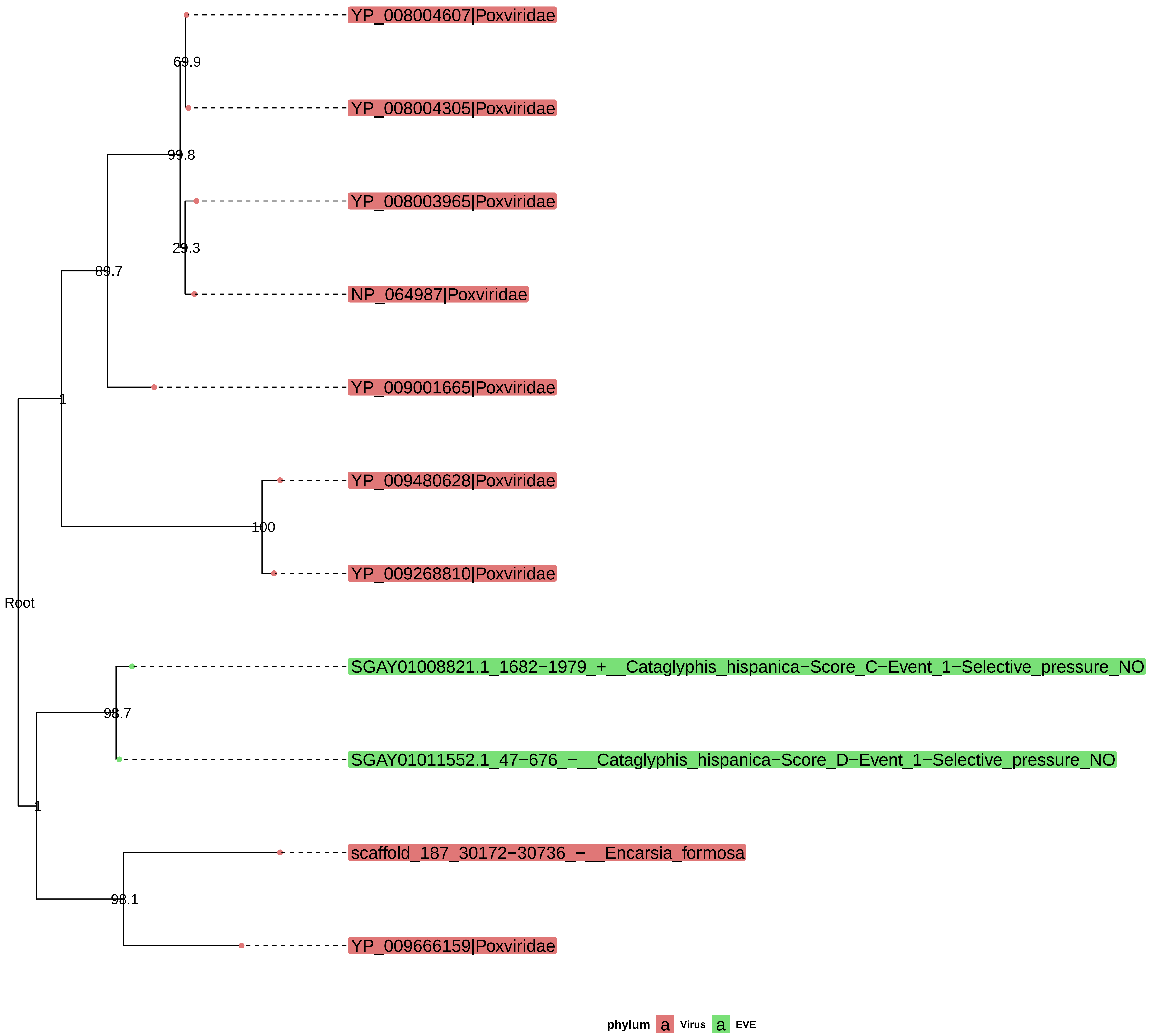

Cluster15833 − families involved : ['Nimaviridae'] − putative protein : ['ORF1211']

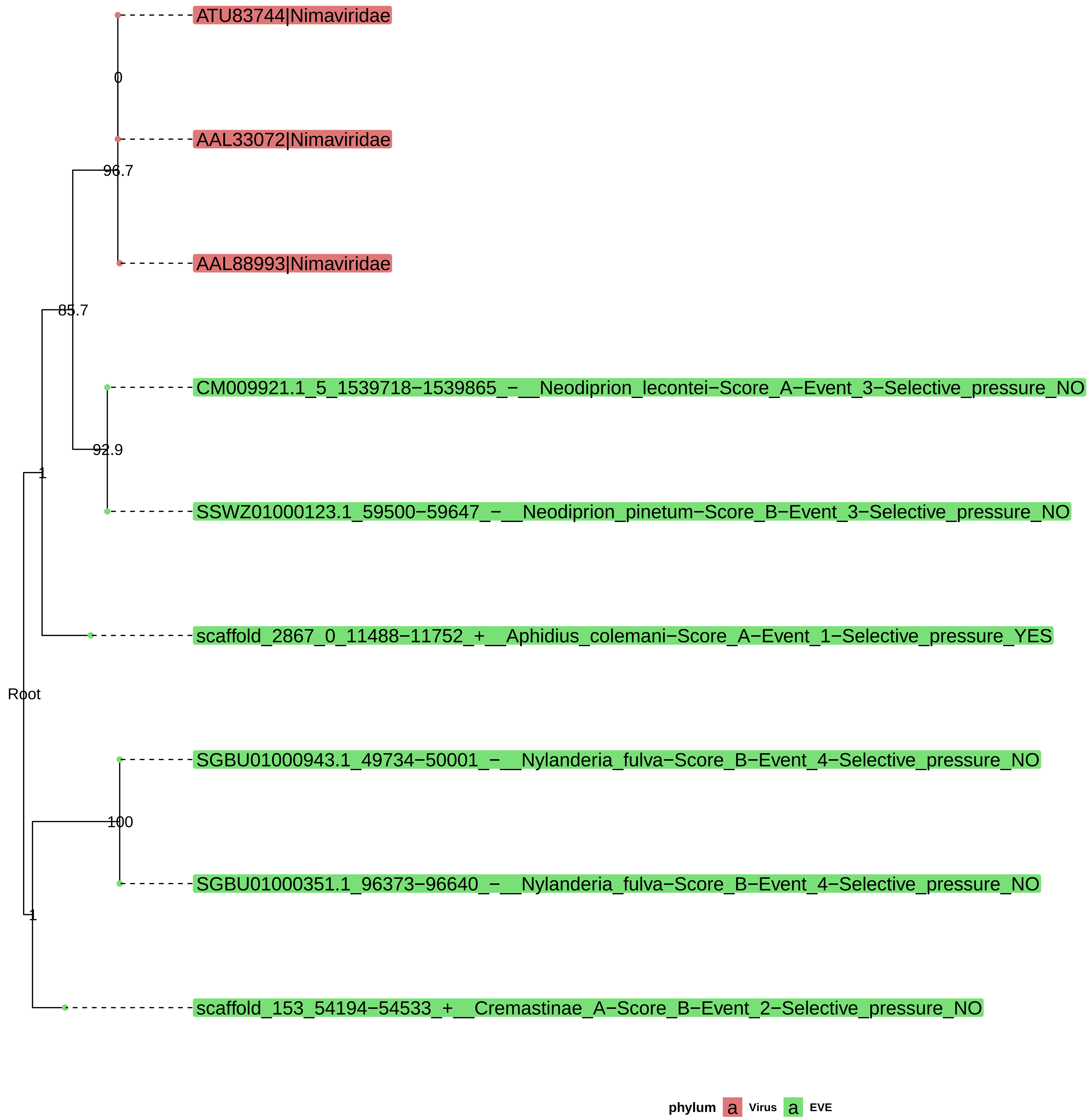

Cluster2905 − families involved : ['Poxviridae', 'Baculoviridae'] − putative protein : ['Leucine rich repeat gene family']

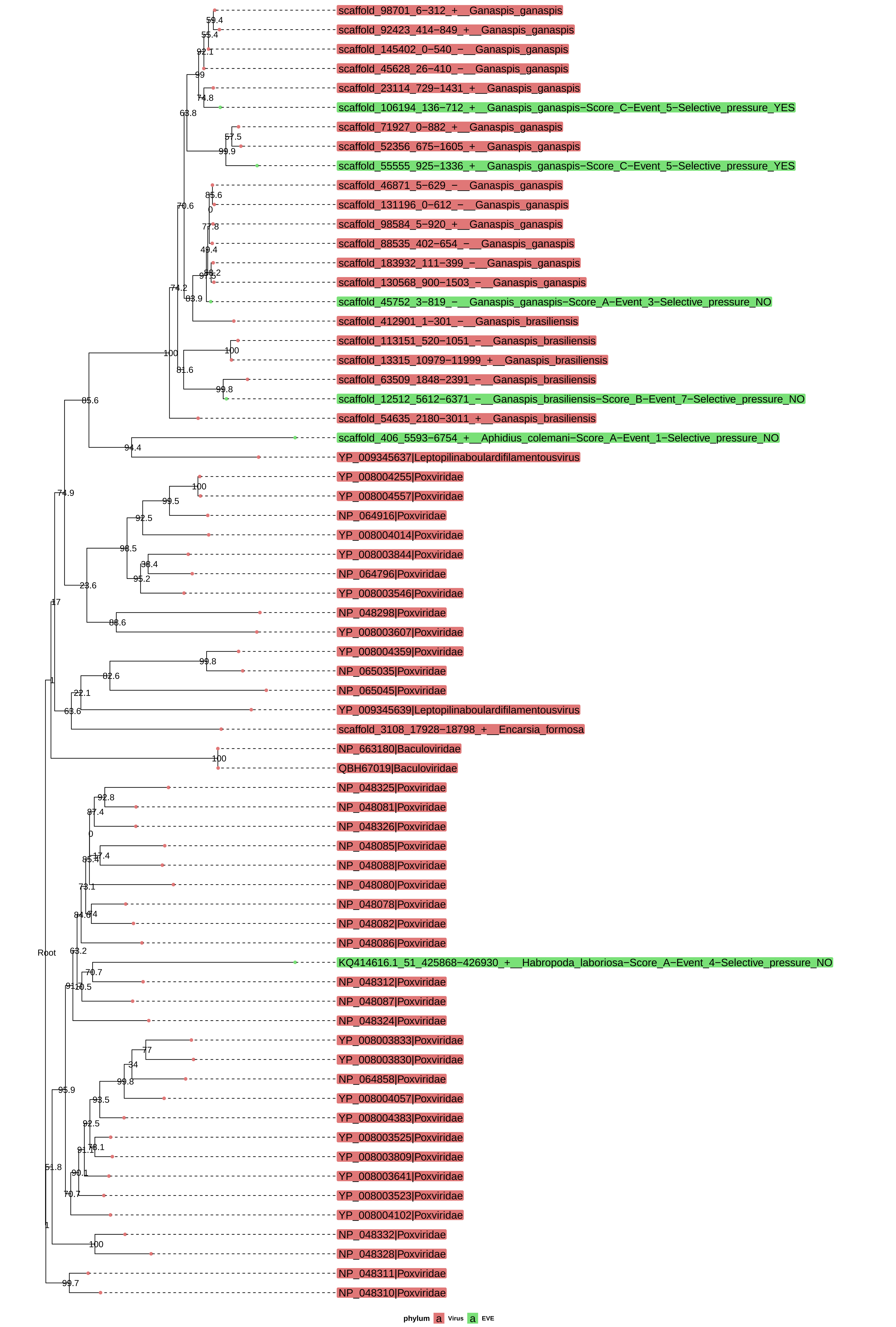

Cluster15309 − families involved : ['Phycodnaviridae'] − putative protein : ['Uncharacterized protein']

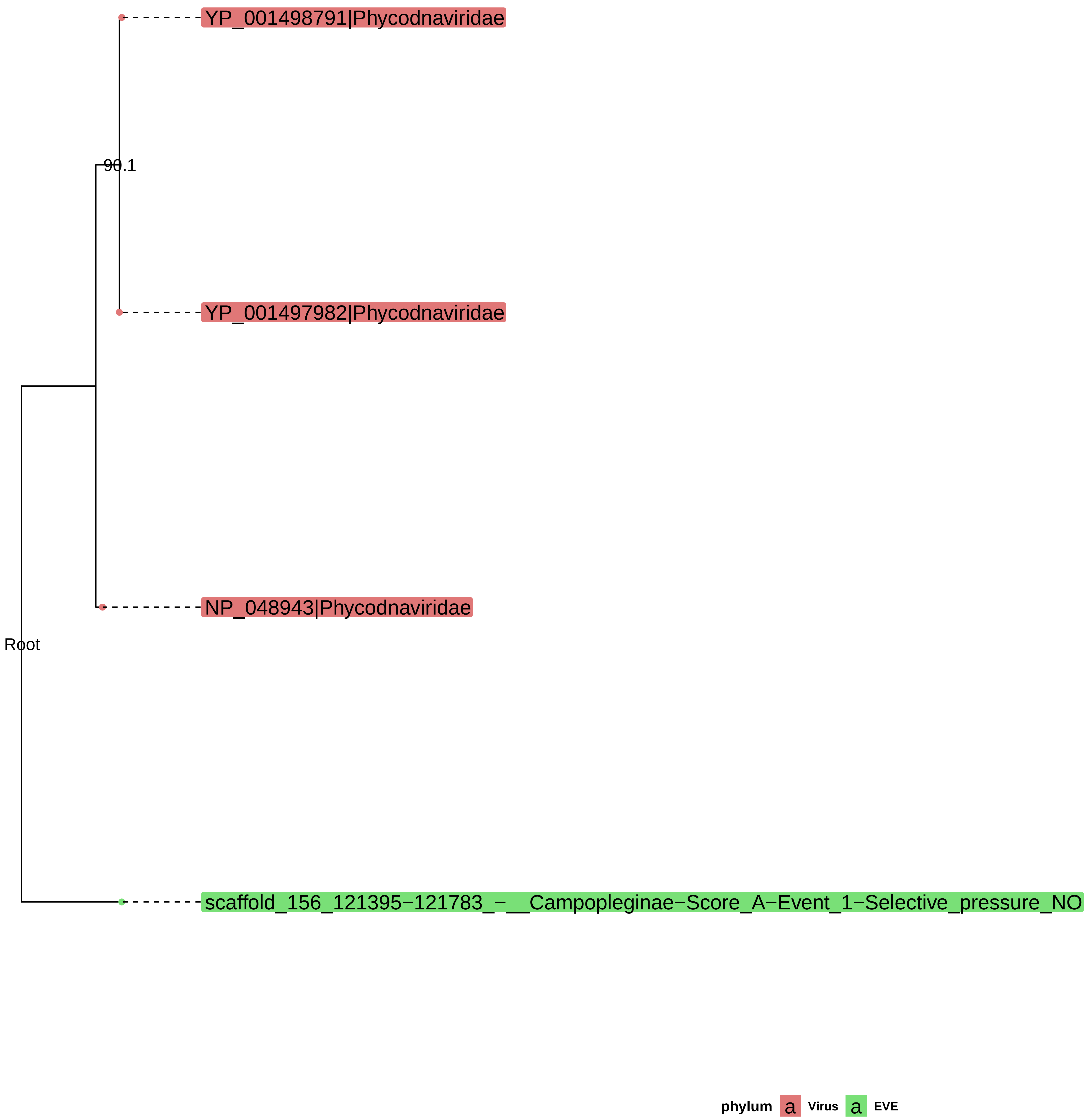

Cluster15201 − families involved : ['Parvoviridae'] − putative protein : ['Non−structural protein 1']

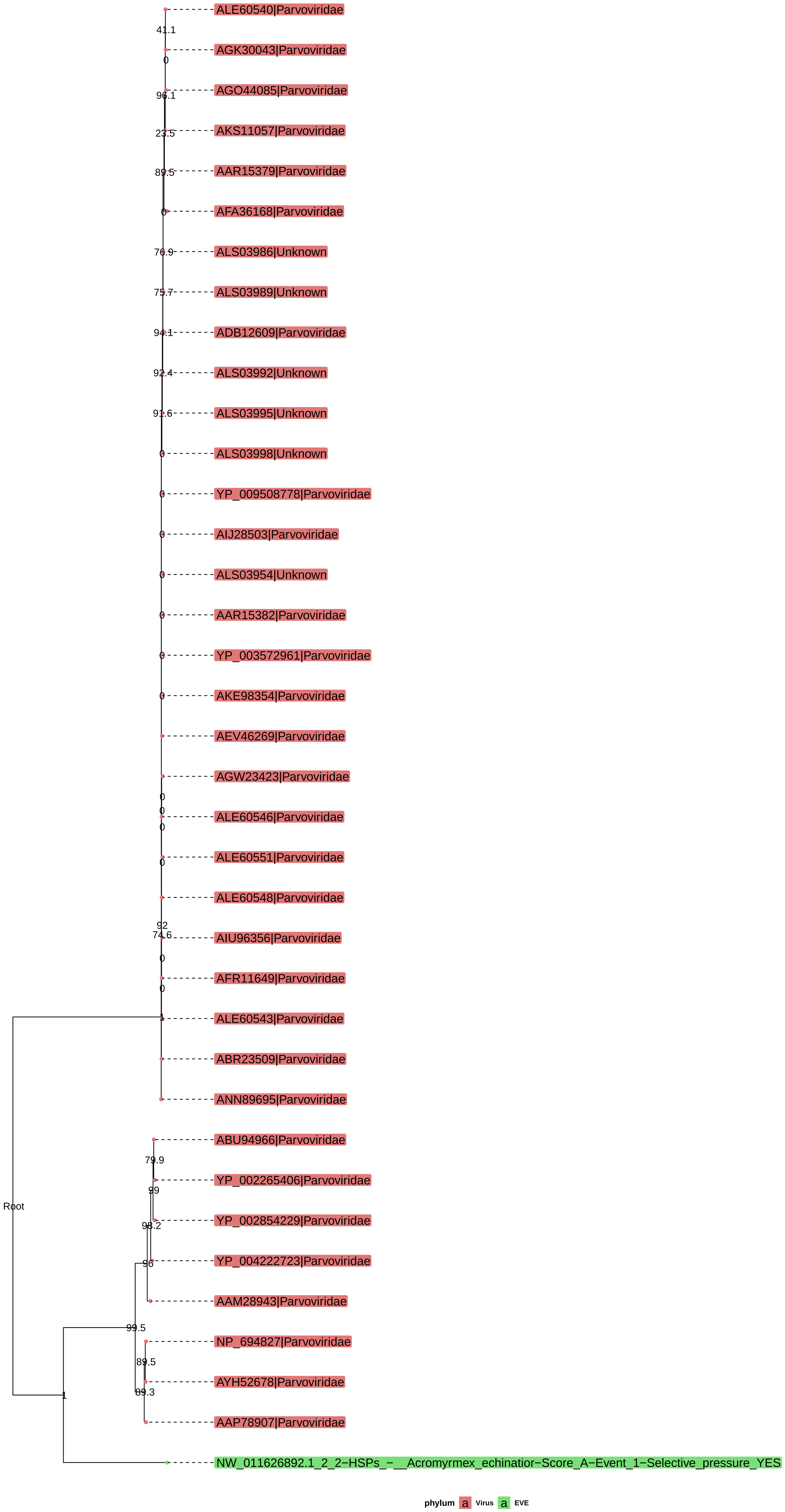

Cluster5853 − families involved : ['Nudiviridae'] − putative protein : ['GrBNV_gp72−like protein']

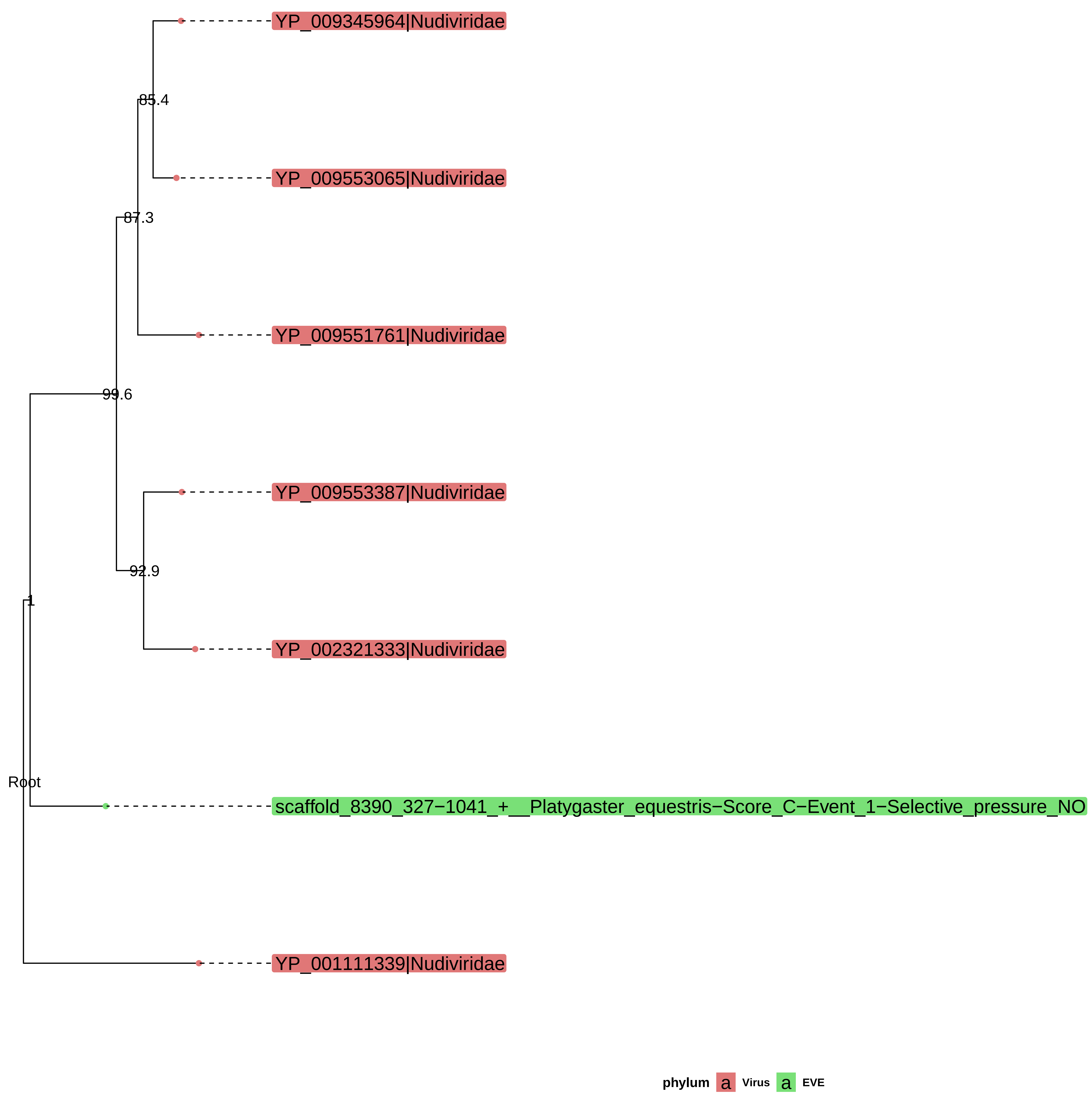

Cluster14552 − families involved : ['Poxviridae'] − putative protein : ['ATPase']

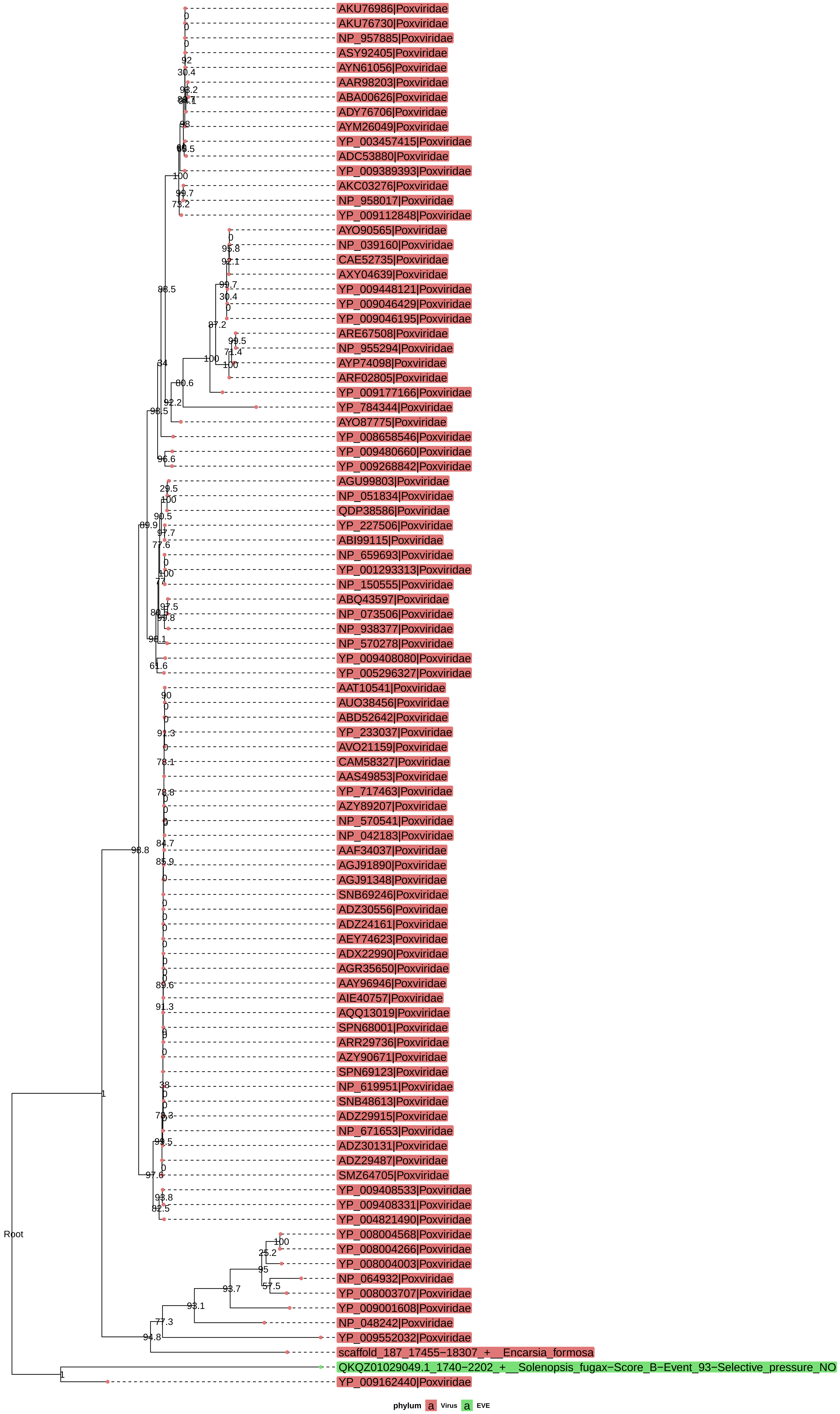

Cluster14344 − families involved : ['Partiti−Picobirna'] − putative protein : ['DNA photolyase']

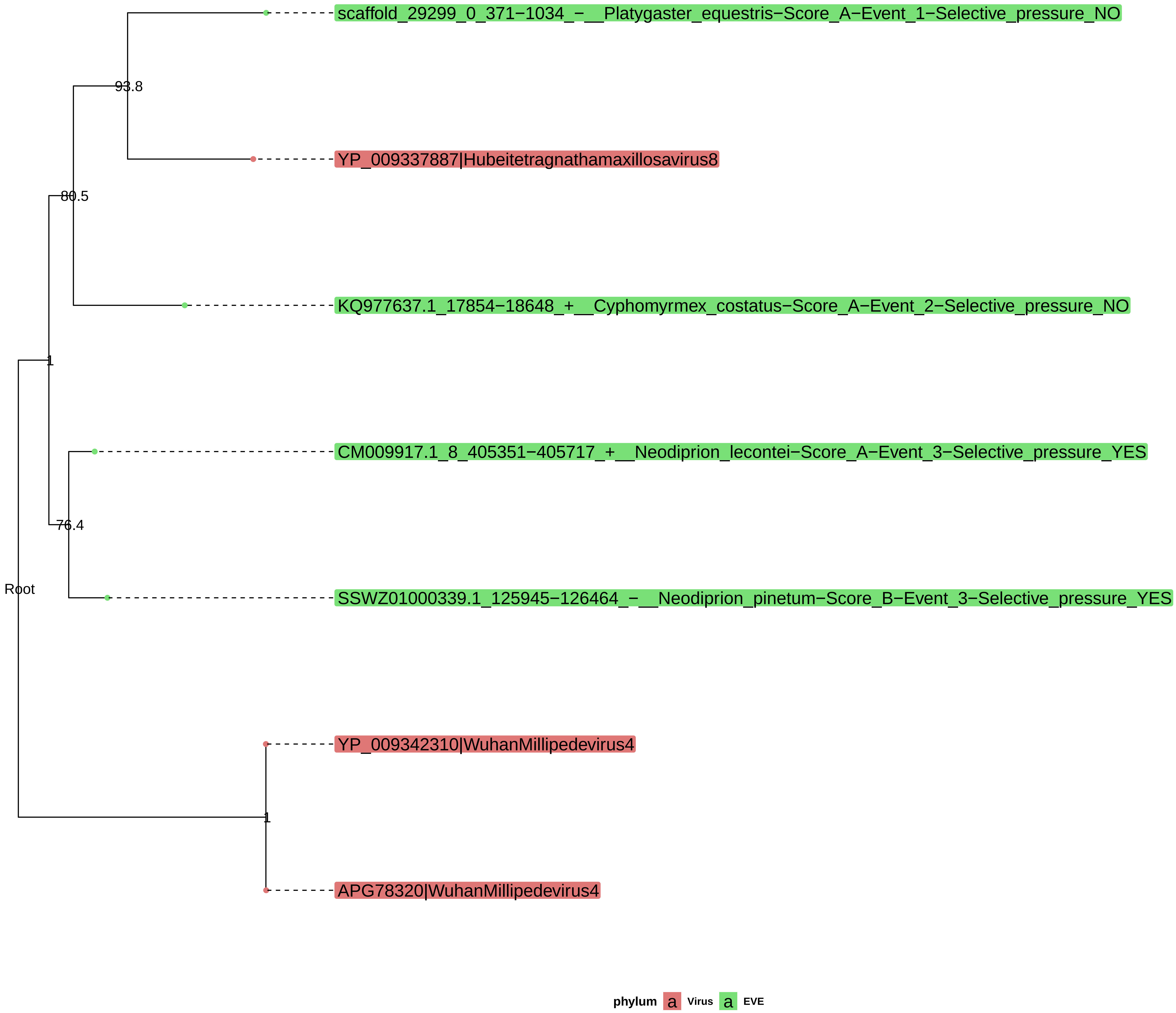

Cluster8607 − families involved : ['Unknown'] − putative protein : ['integral component of membrane [GO:0016021]']

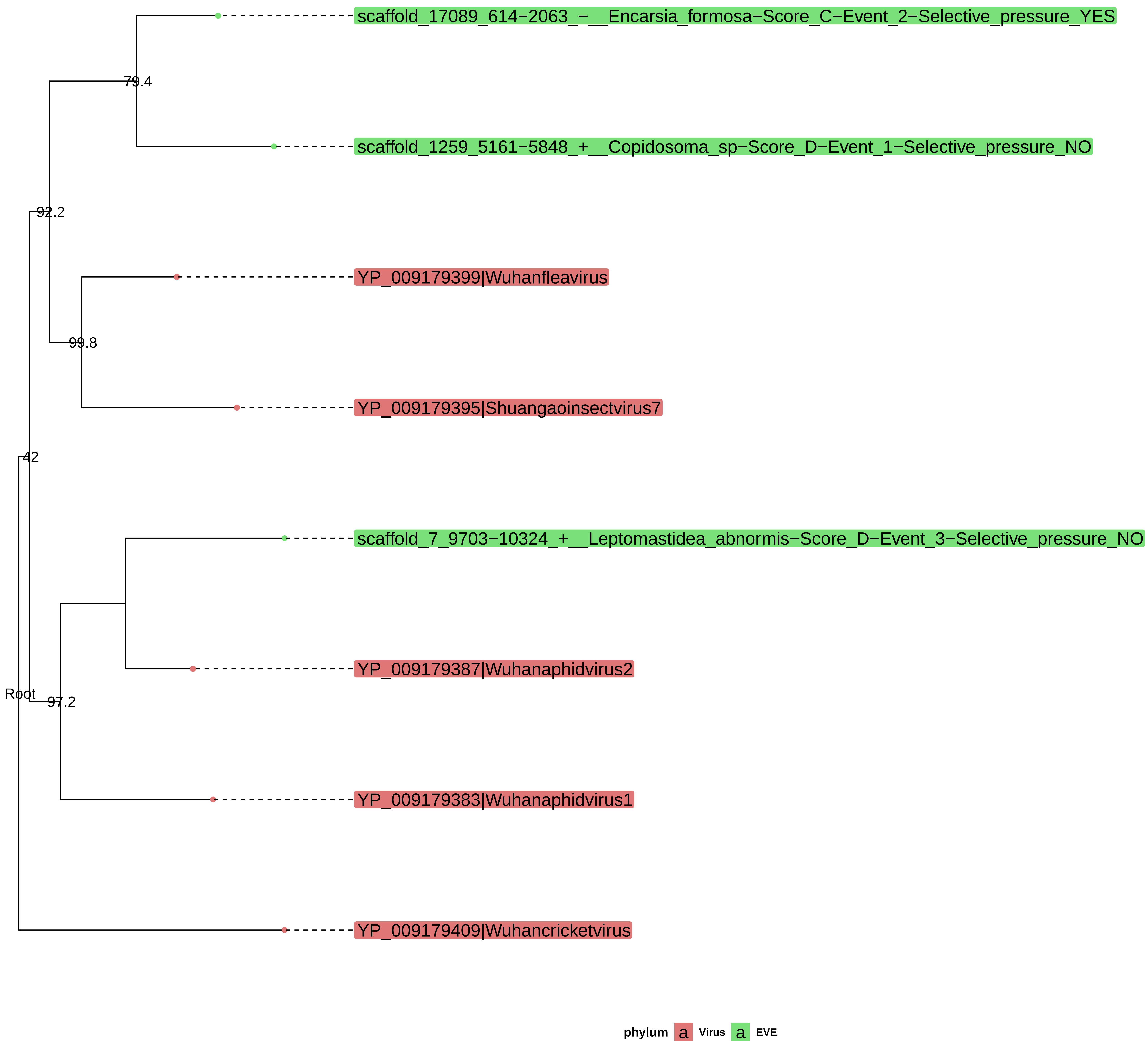

Cluster13782 − families involved : ['Unknown'] − putative protein : ['Putative capsid protein']

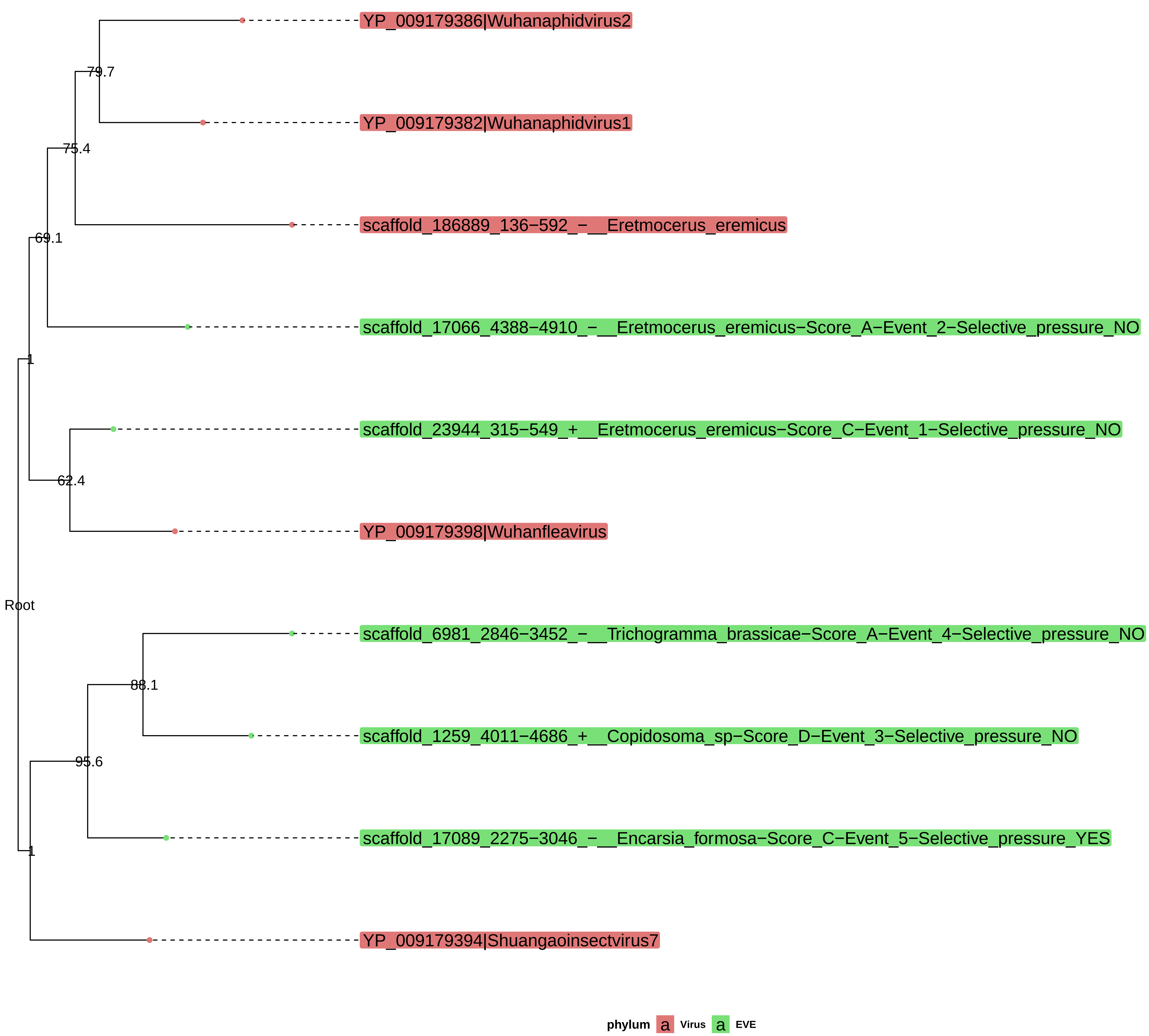

Cluster13533 − families involved : ['Reoviridae'] − putative protein : ['VP11']

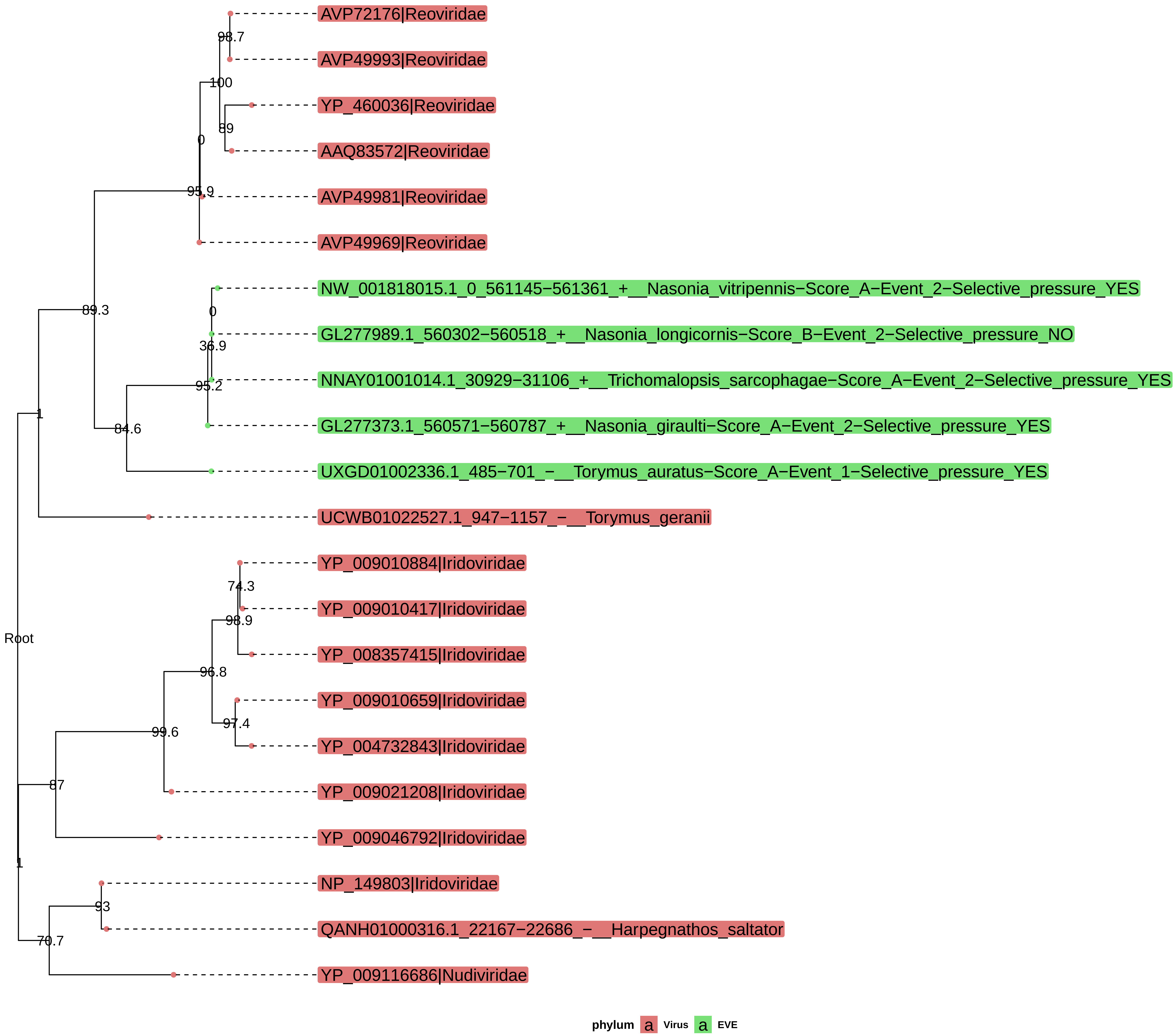

Cluster13442 − families involved : ['Peribunyaviridae'] − putative protein : ['Nucleocapsid protein (Nucleoprotein)']

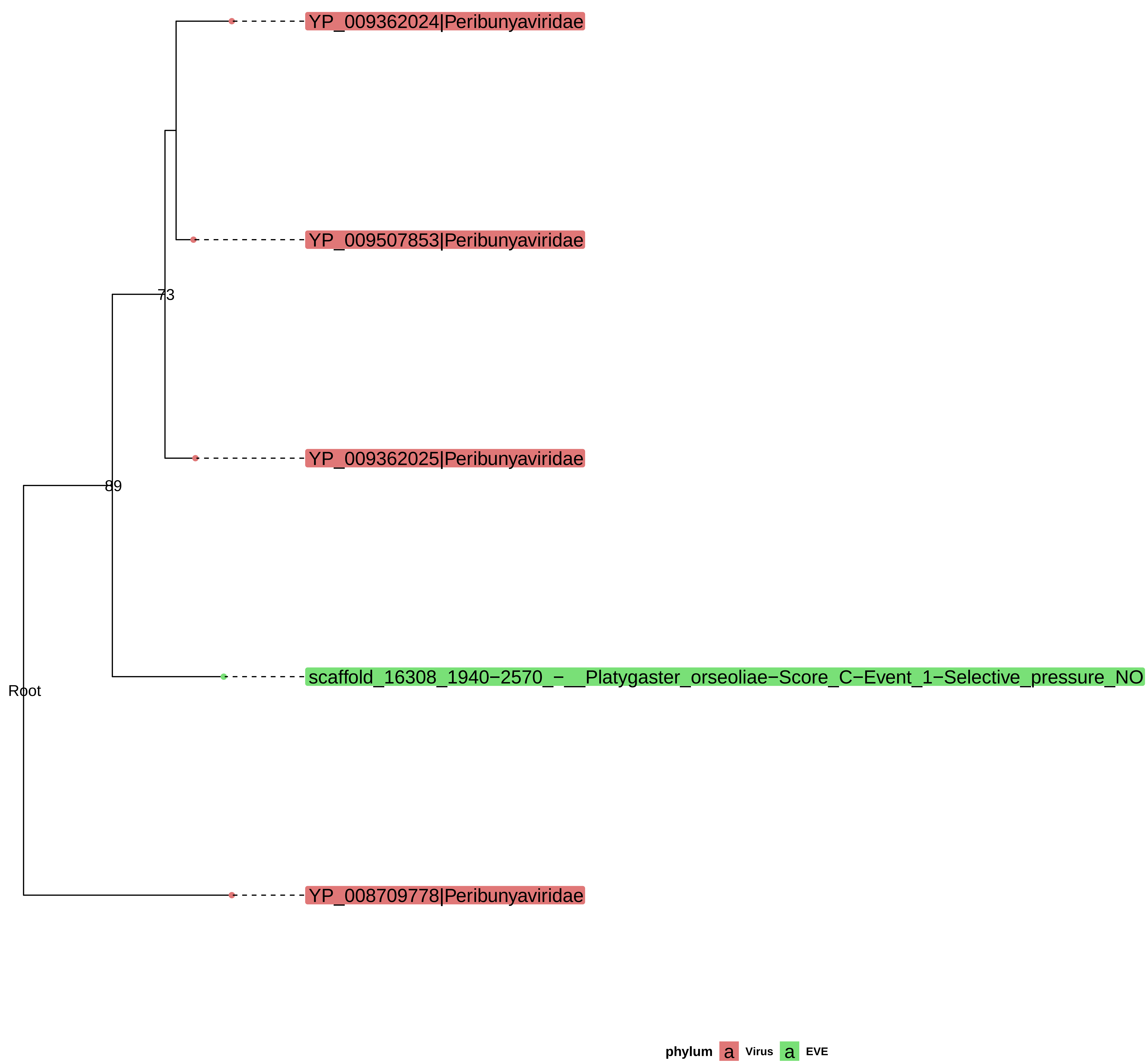

Cluster13438 − families involved : ['Phenuiviridae', 'Bunya−Arena'] − putative protein : ['Replicase (EC 2.7.7.48) (Transcriptase)']

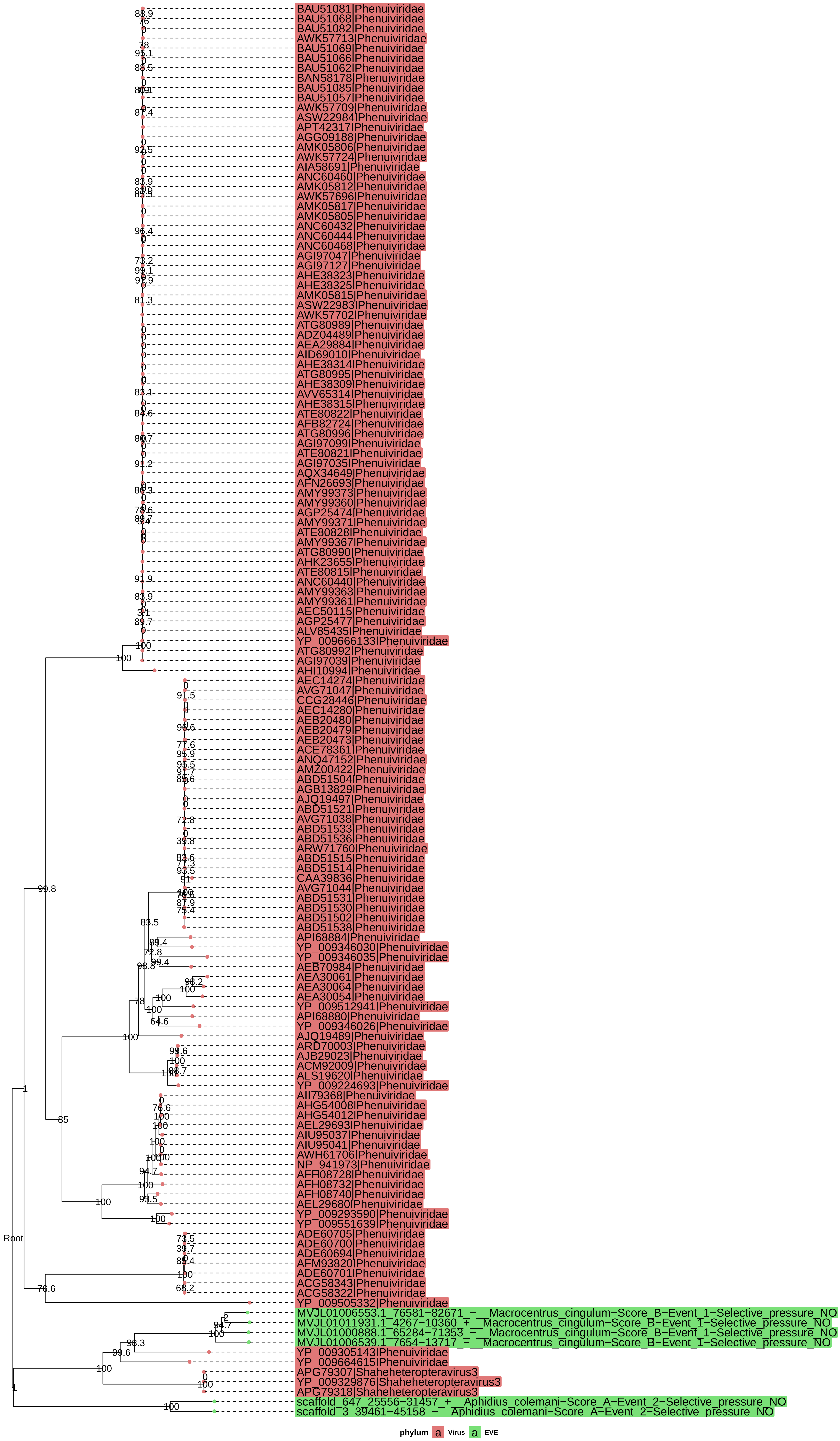

Cluster6003 − families involved : ['Artoviridae', 'Nyamiviridae', 'Chuviridae', 'Xinmoviridae', 'Rhabdoviridae', 'Bornaviridae', 'Mono−Chu', nan, 'Lispiviridae'] − putative protein : ["GDP polyribonucleotidyl

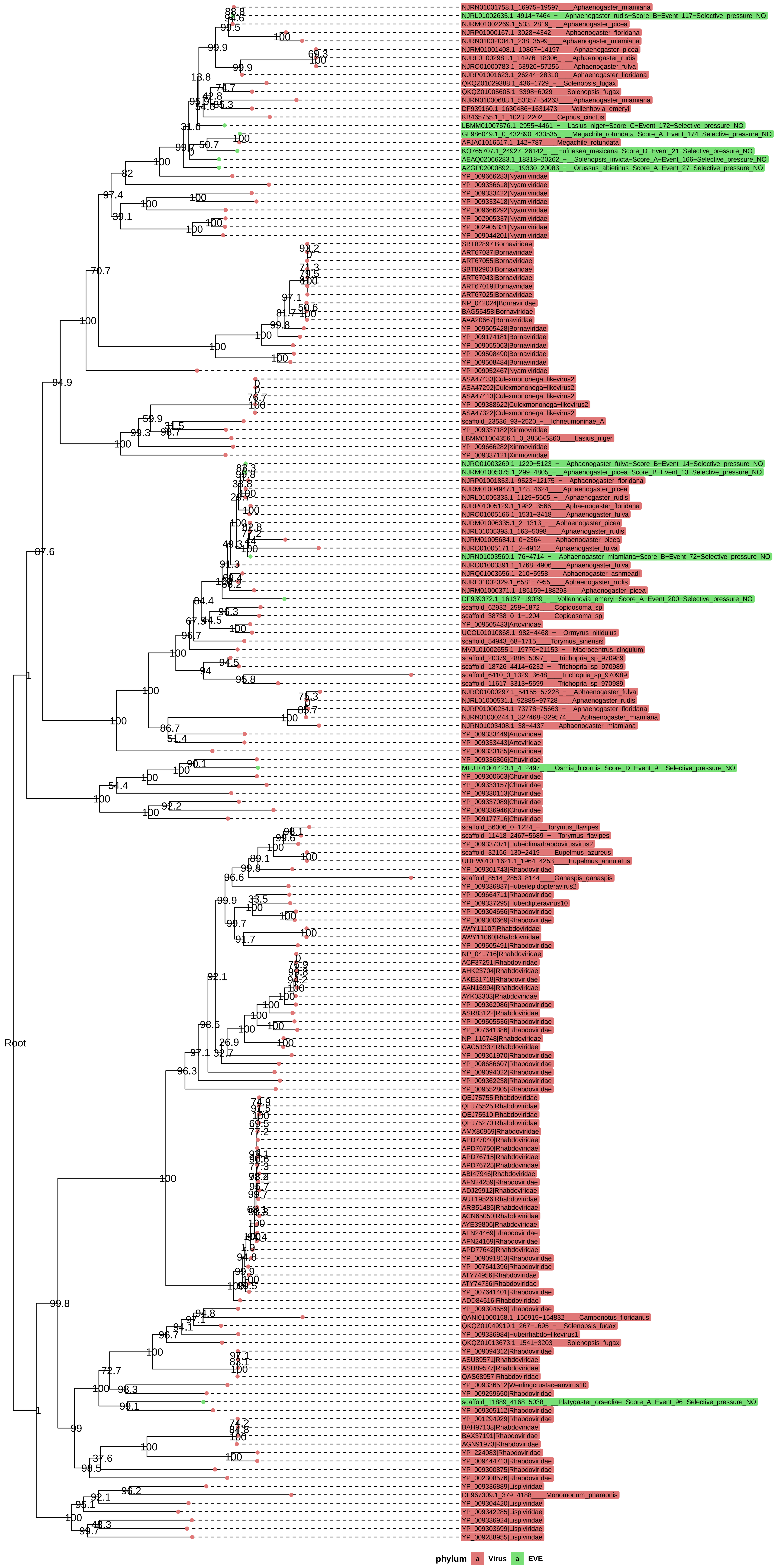

Cluster13237 − families involved : ['Artoviridae'] − putative protein : ['Membrane fusion protein Use1']

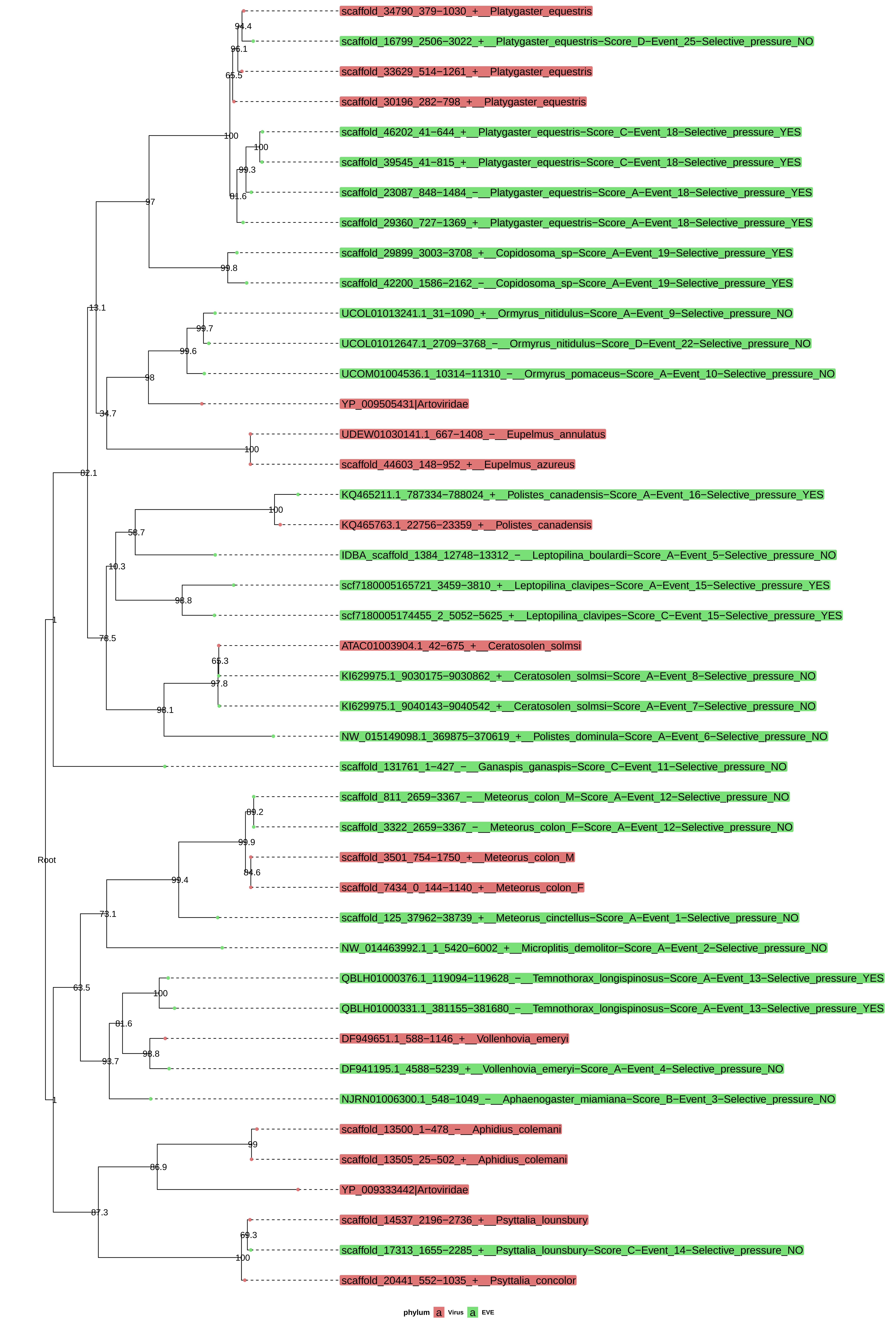

Cluster13229 − families involved : ['Phenuiviridae'] − putative protein : ['Nucleoprotein']

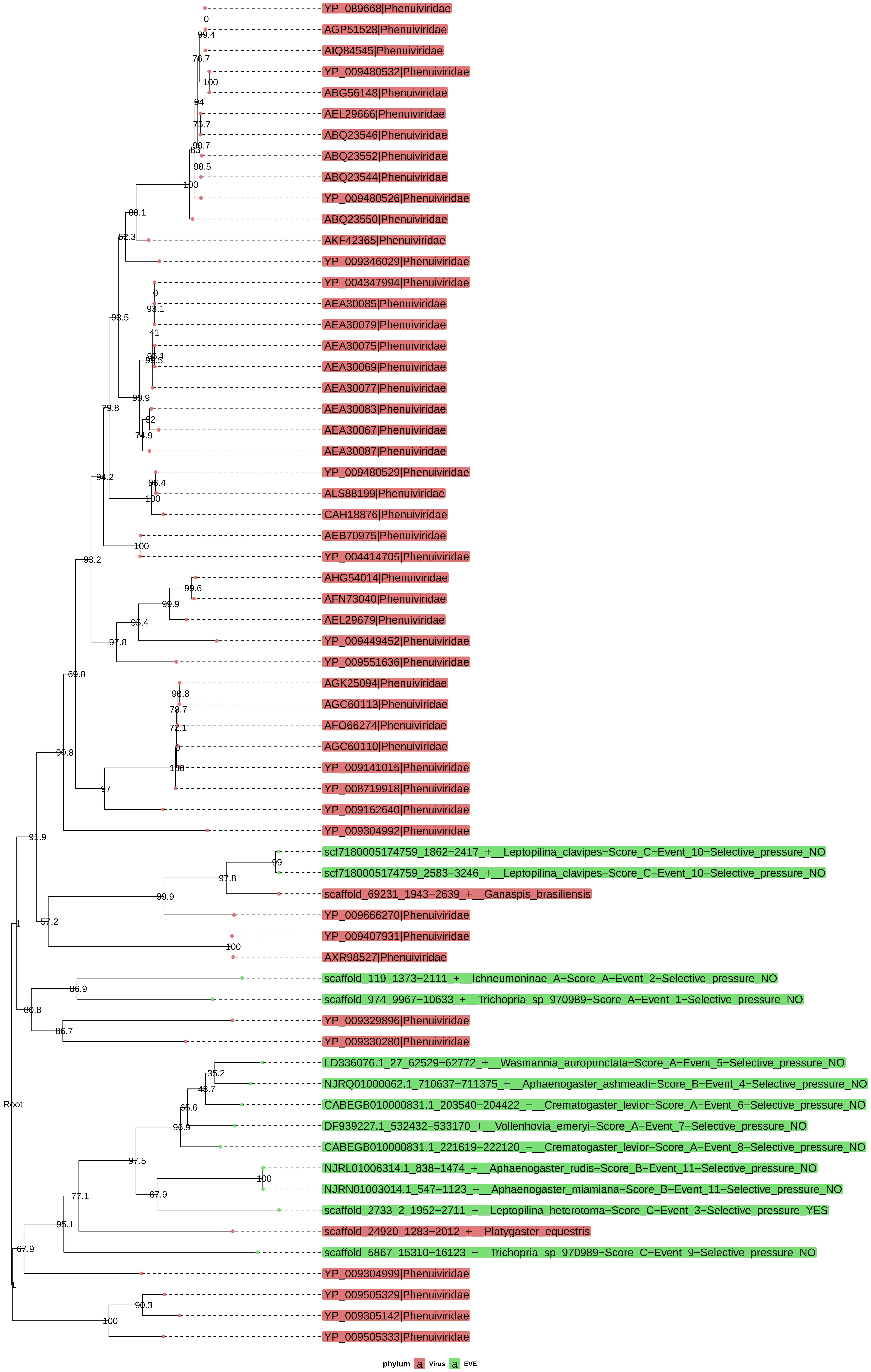

Cluster13047 − families involved : ['Mono−Chu'] − putative protein : ['Putative glycoprotein']

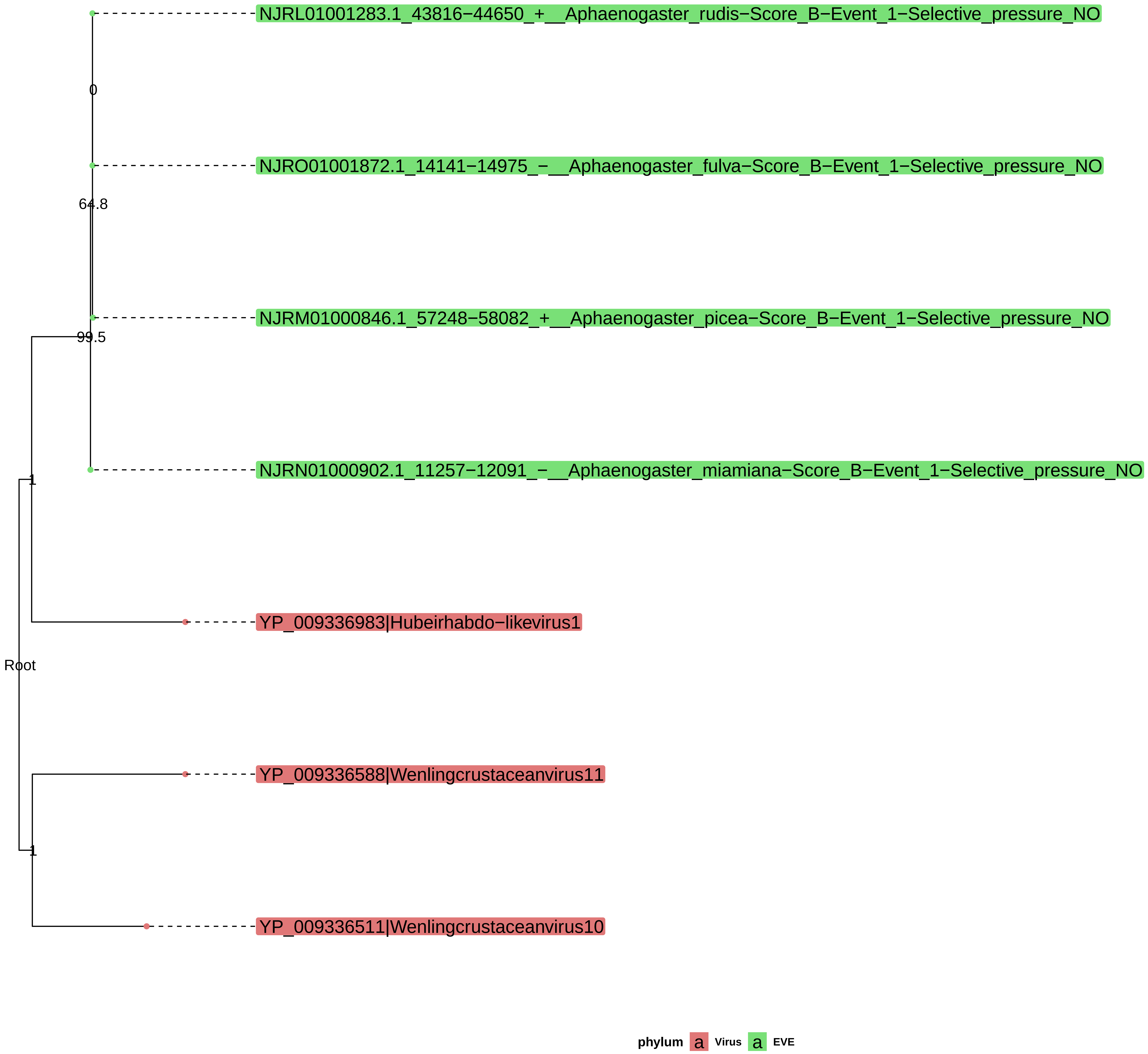

Cluster5646 − families involved : ['Hepe−Virga', 'Tymoviridae'] − putative protein : ['Uncharacterized protein']

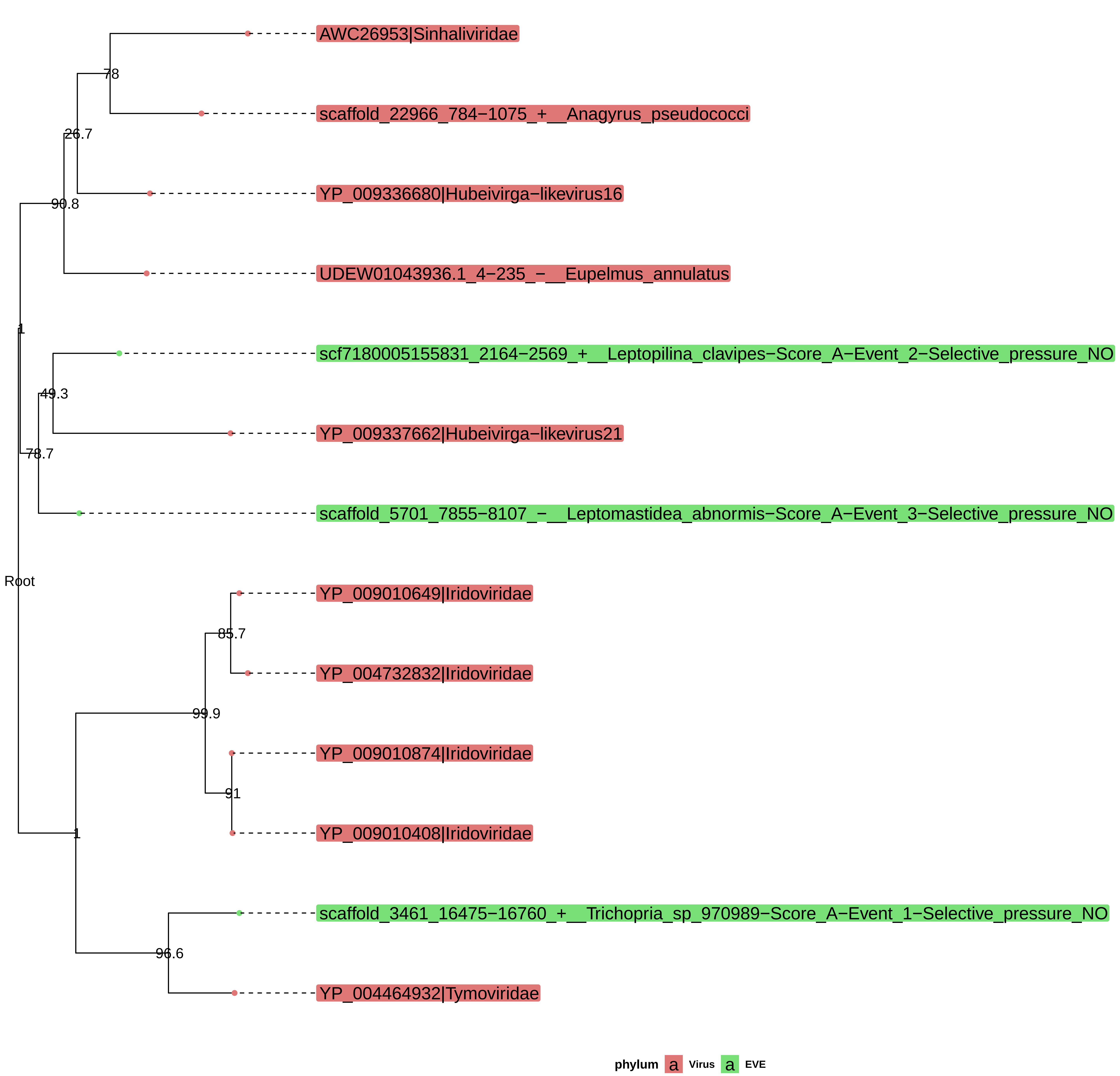

Cluster12625 − families involved : ['Lispiviridae', nan] − putative protein : ['Gluconate 2−dehydrogenase subunit 3', nan]

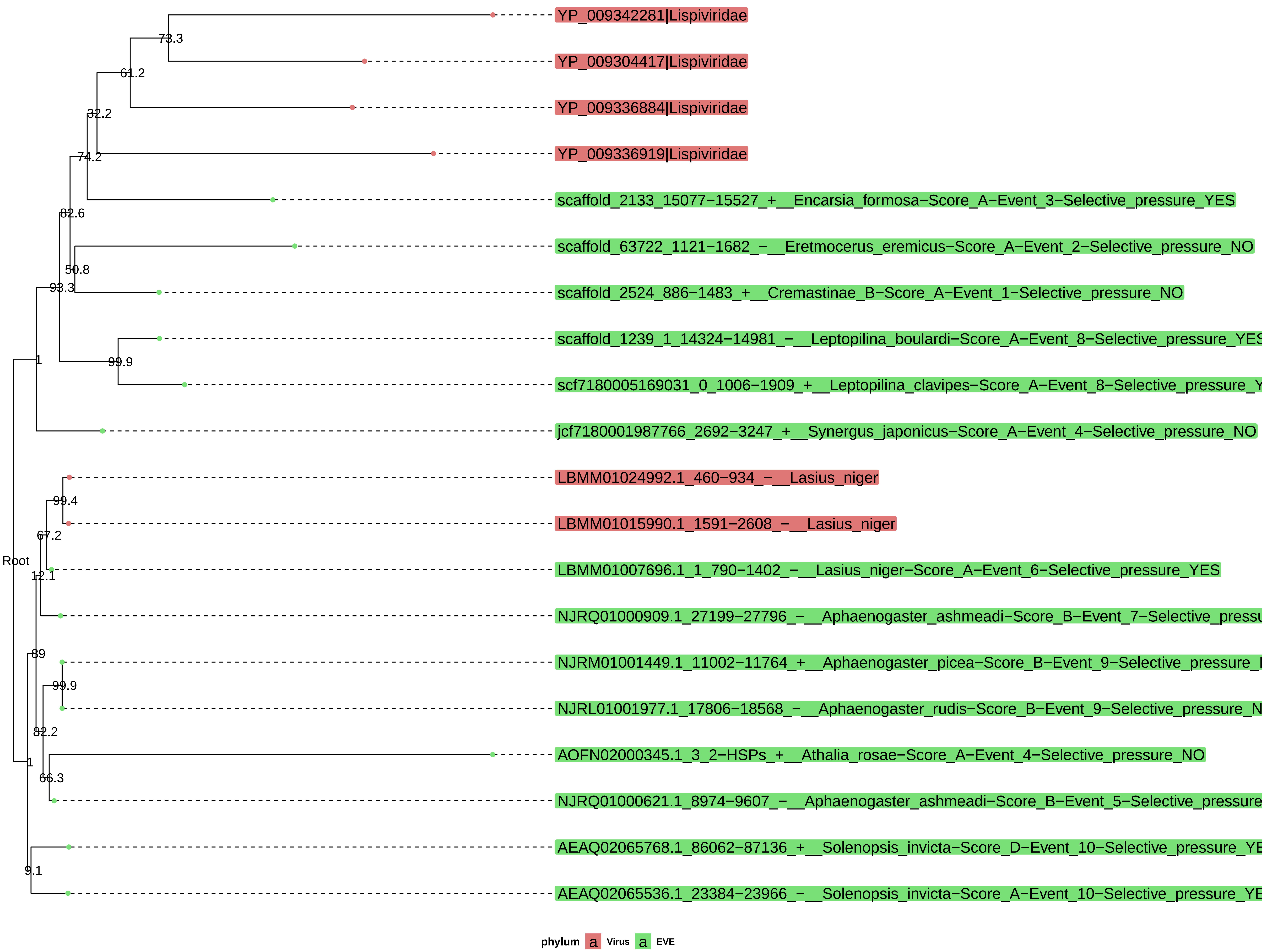

Cluster11728 − families involved : ['Phycodnaviridae'] − putative protein : ['Ribonuclease H (EC 3.1.26.4)']

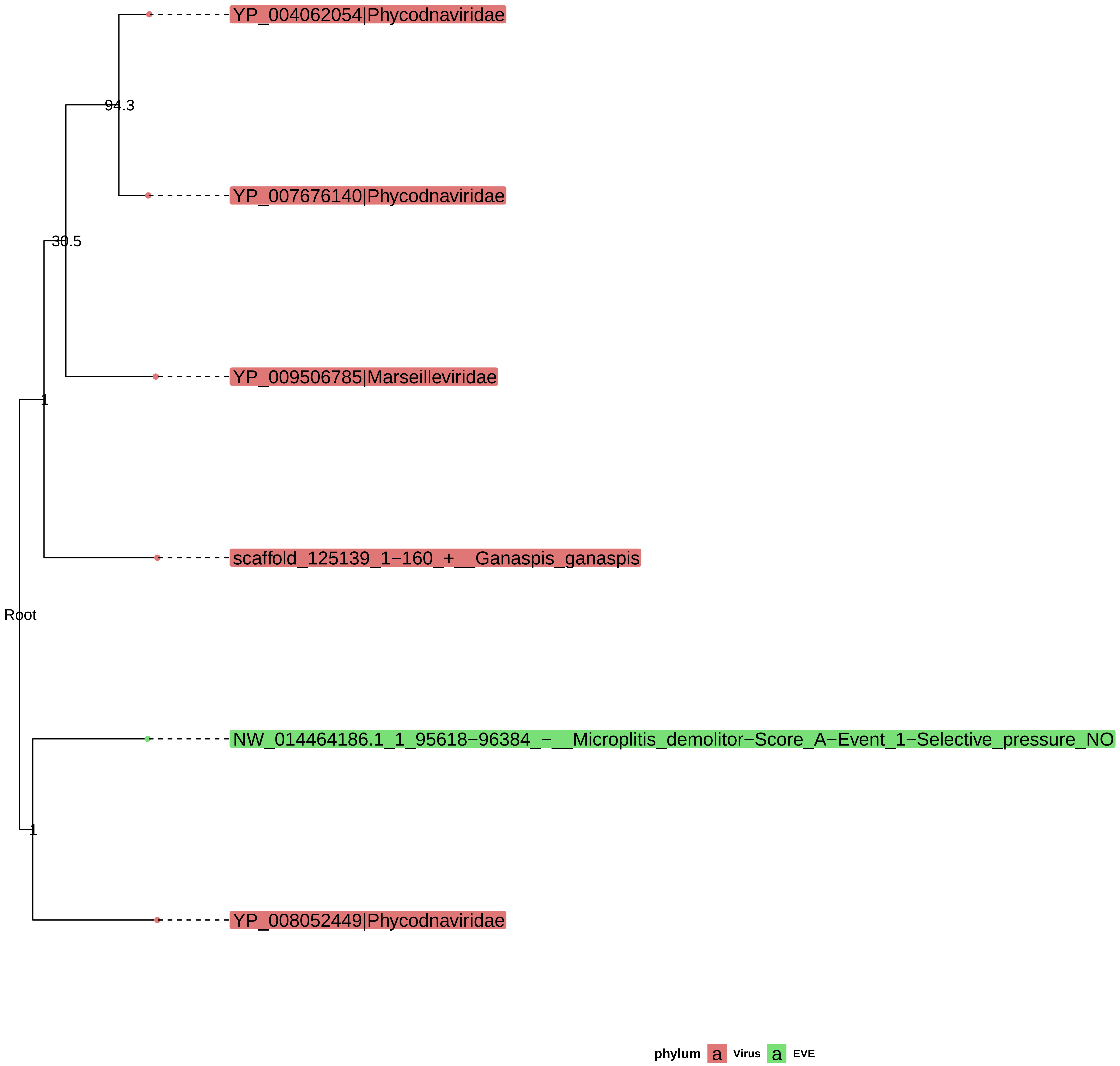

Cluster15744 − families involved : ['Reoviridae'] − putative protein : ['RNA−directed RNA polymerase (EC 2.7.7.48)']

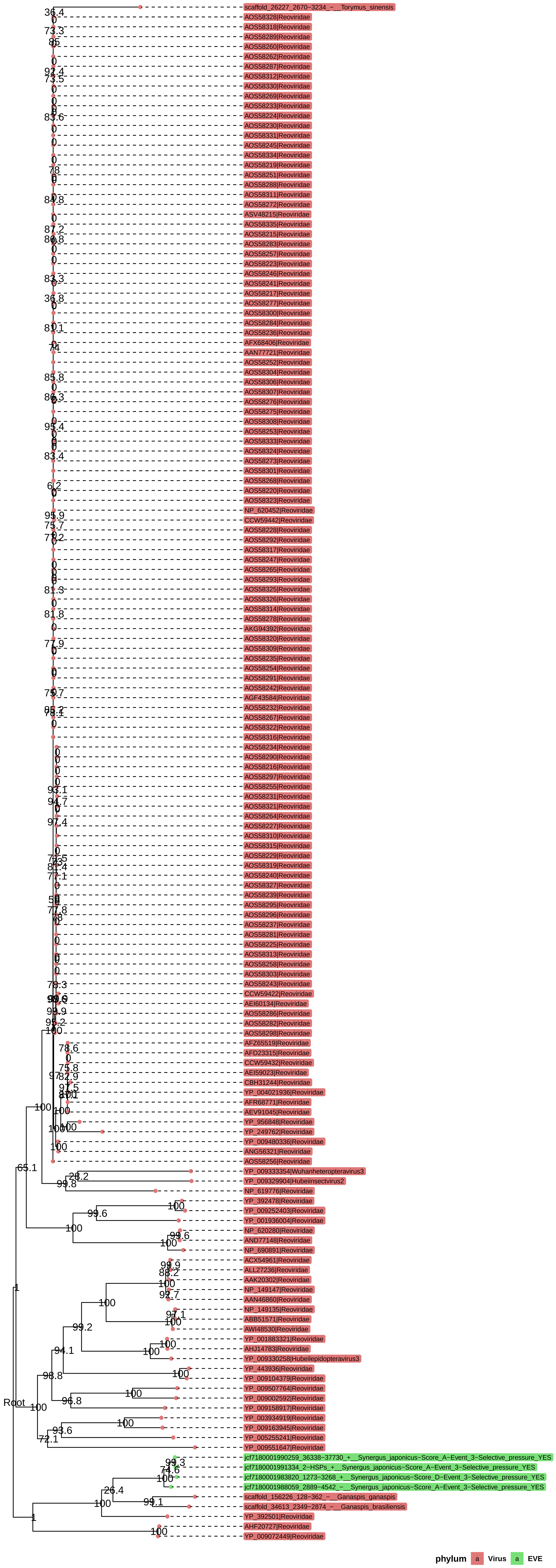

Cluster11392 − families involved : ['Reoviridae'] − putative protein : ['Uncharacterized protein']

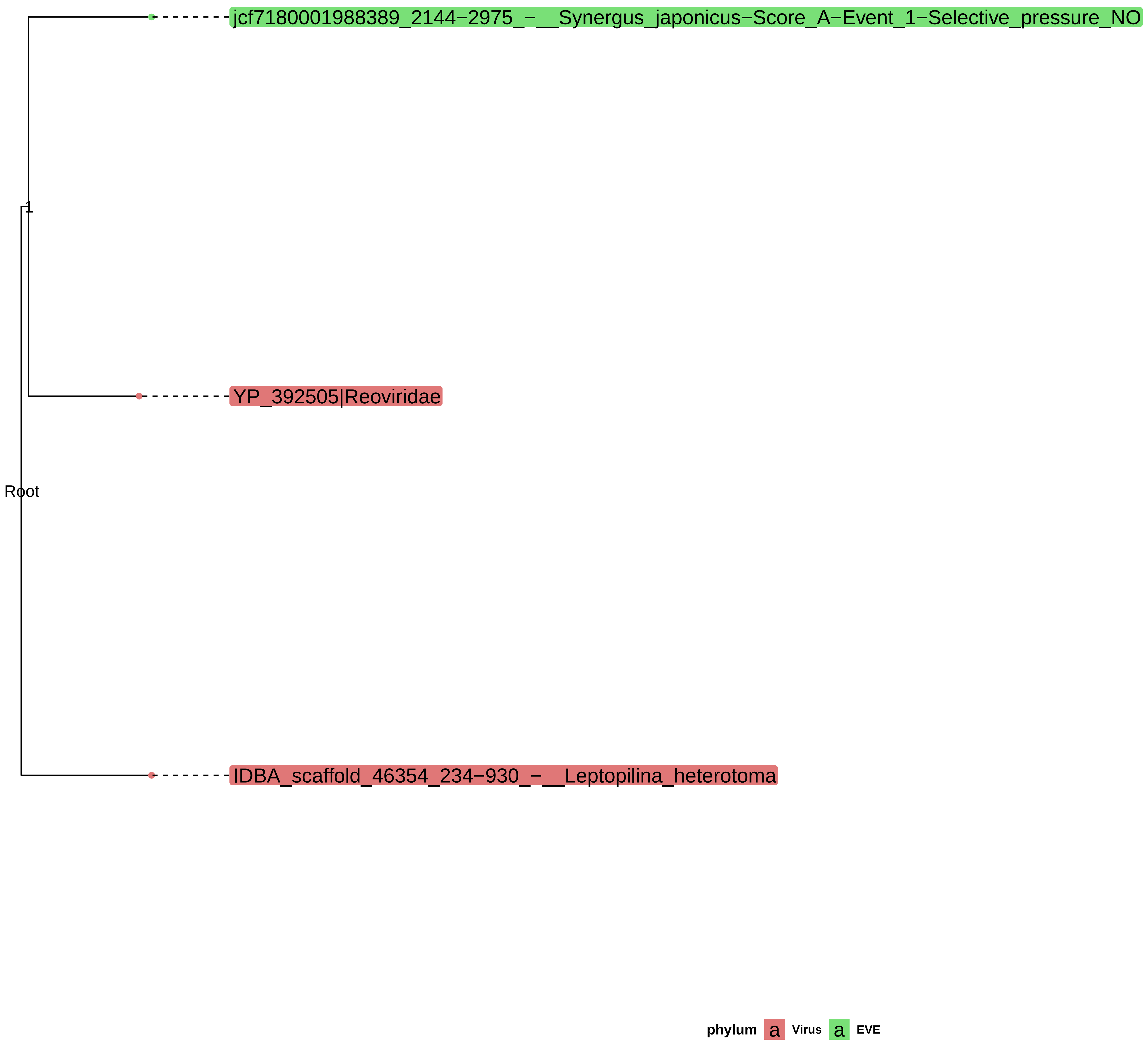

Cluster11354 − families involved : ['Baculoviridae'] − putative protein : ['Uncharacterized protein']

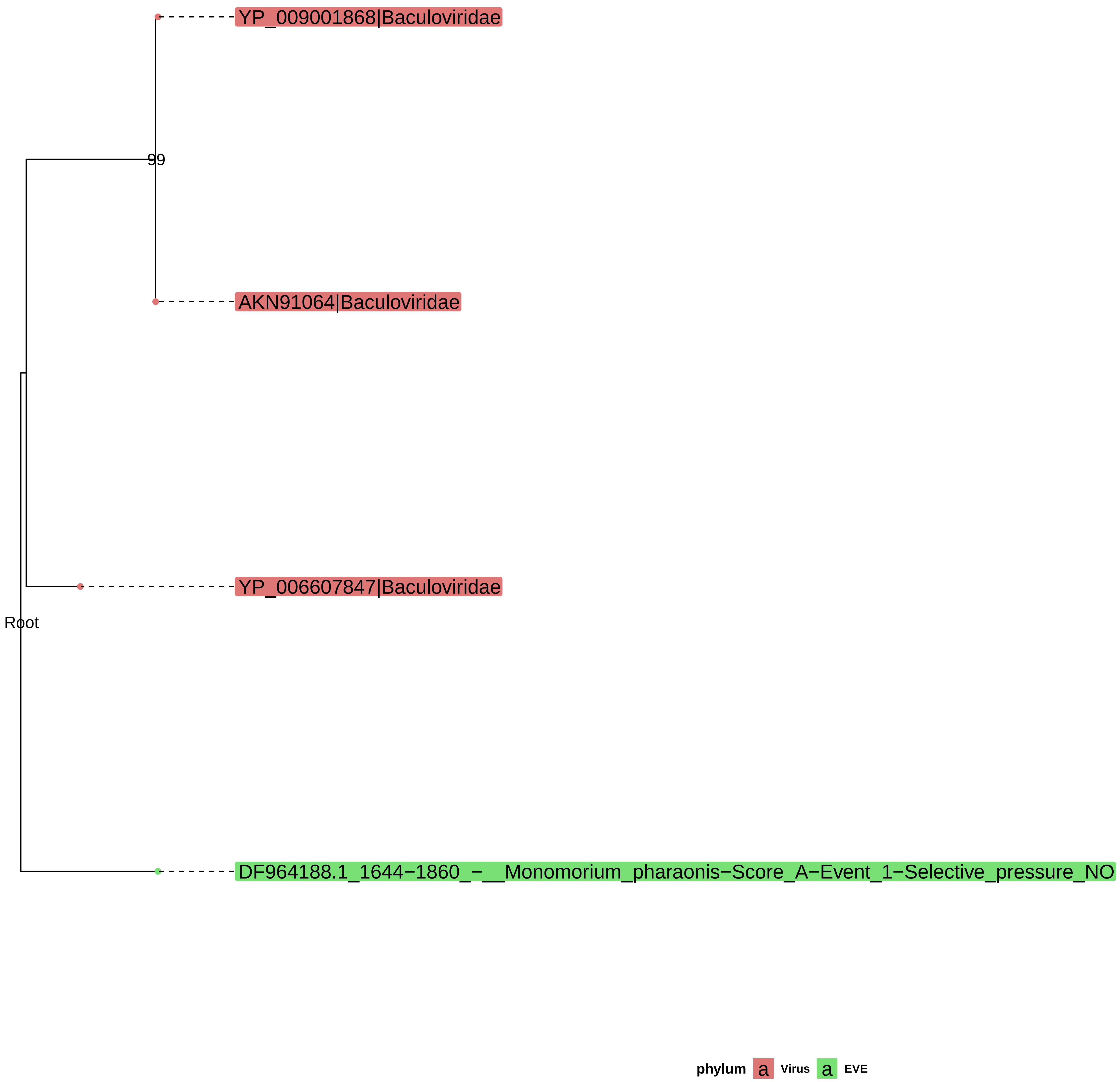

Cluster11297 − families involved : ['Unknown'] − putative protein : ['Non−structural protein 3 (EC 3.6.1.15) (EC 3.6.4.13)']

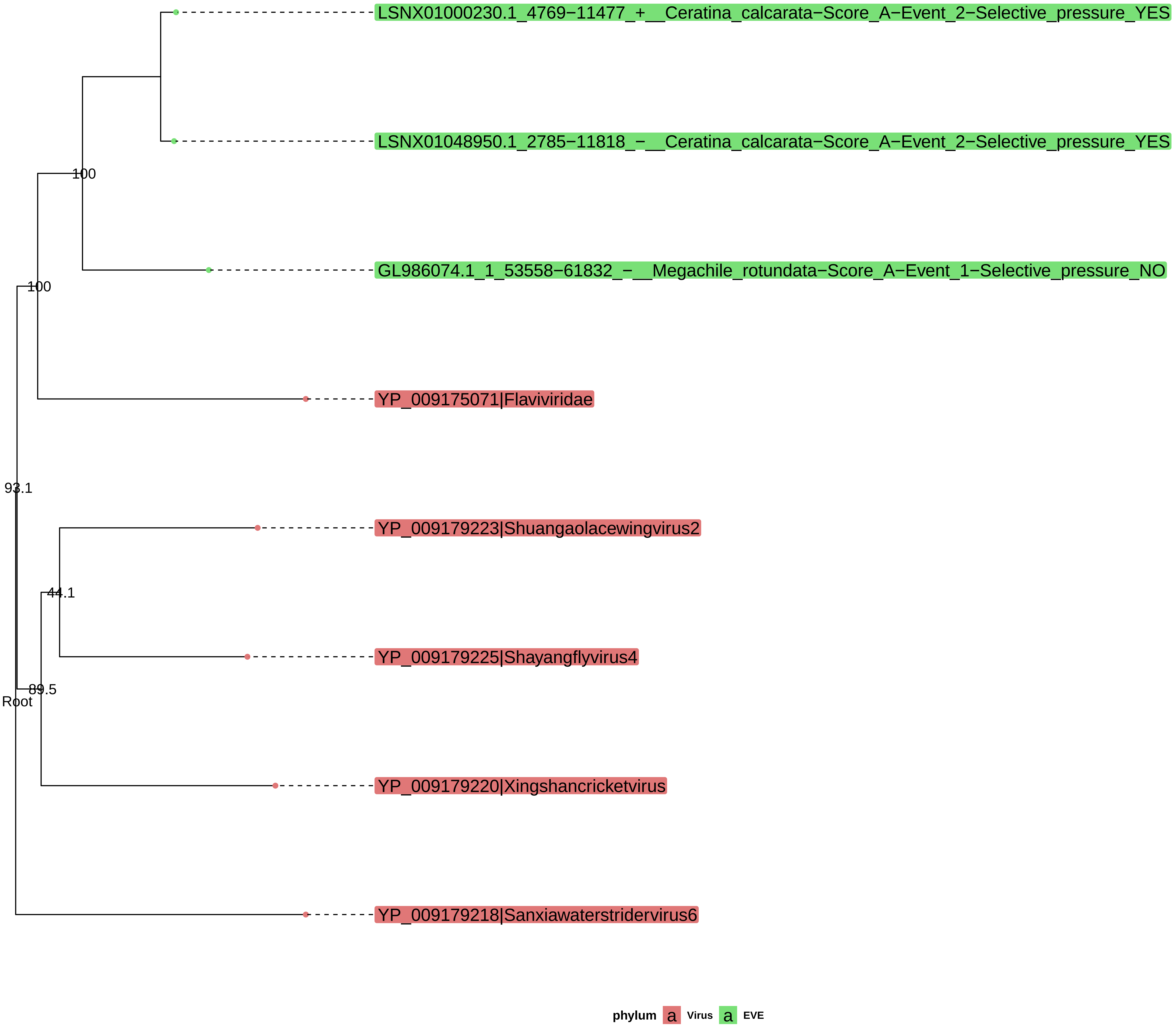

Cluster22220 − families involved : ['LbFV_like'] − putative protein : ['Family of unknown function (DUF5422)']

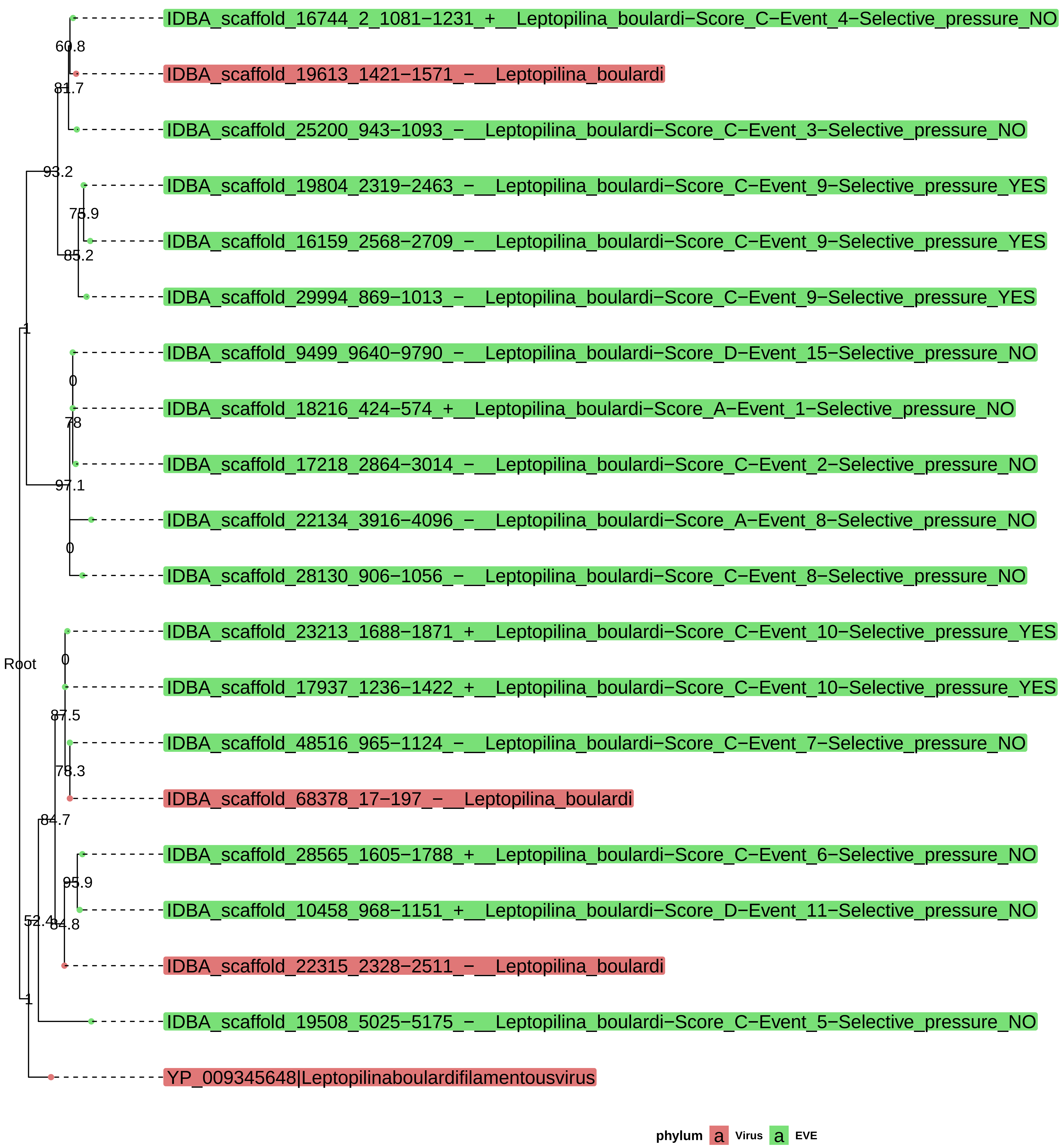

Cluster11296 − families involved : ['LbFV_like'] − putative protein : ['Uncharacterized protein']

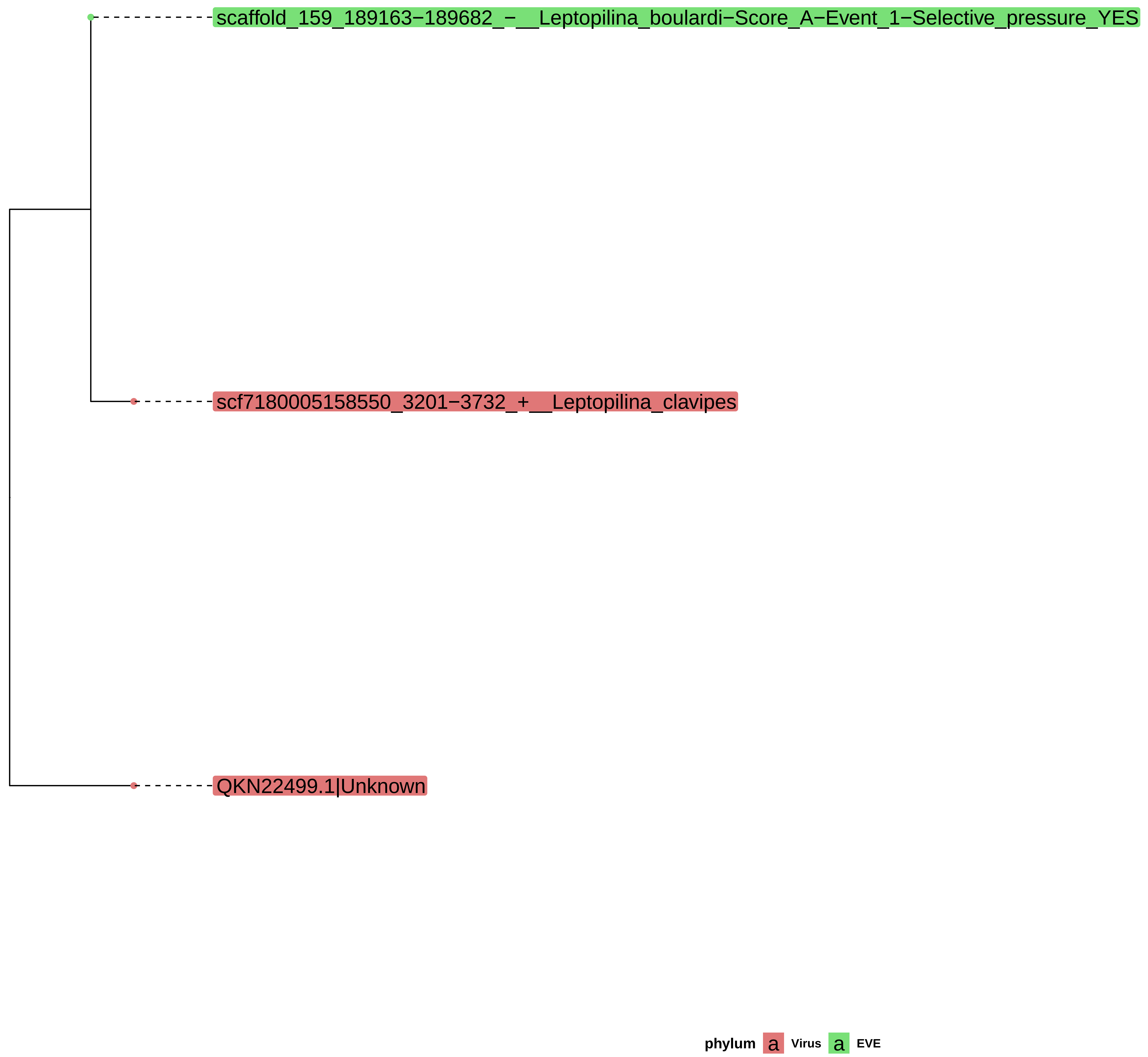

Cluster3075 − families involved : ['Reoviridae'] − putative protein : ['Dcp1−like decapping family']

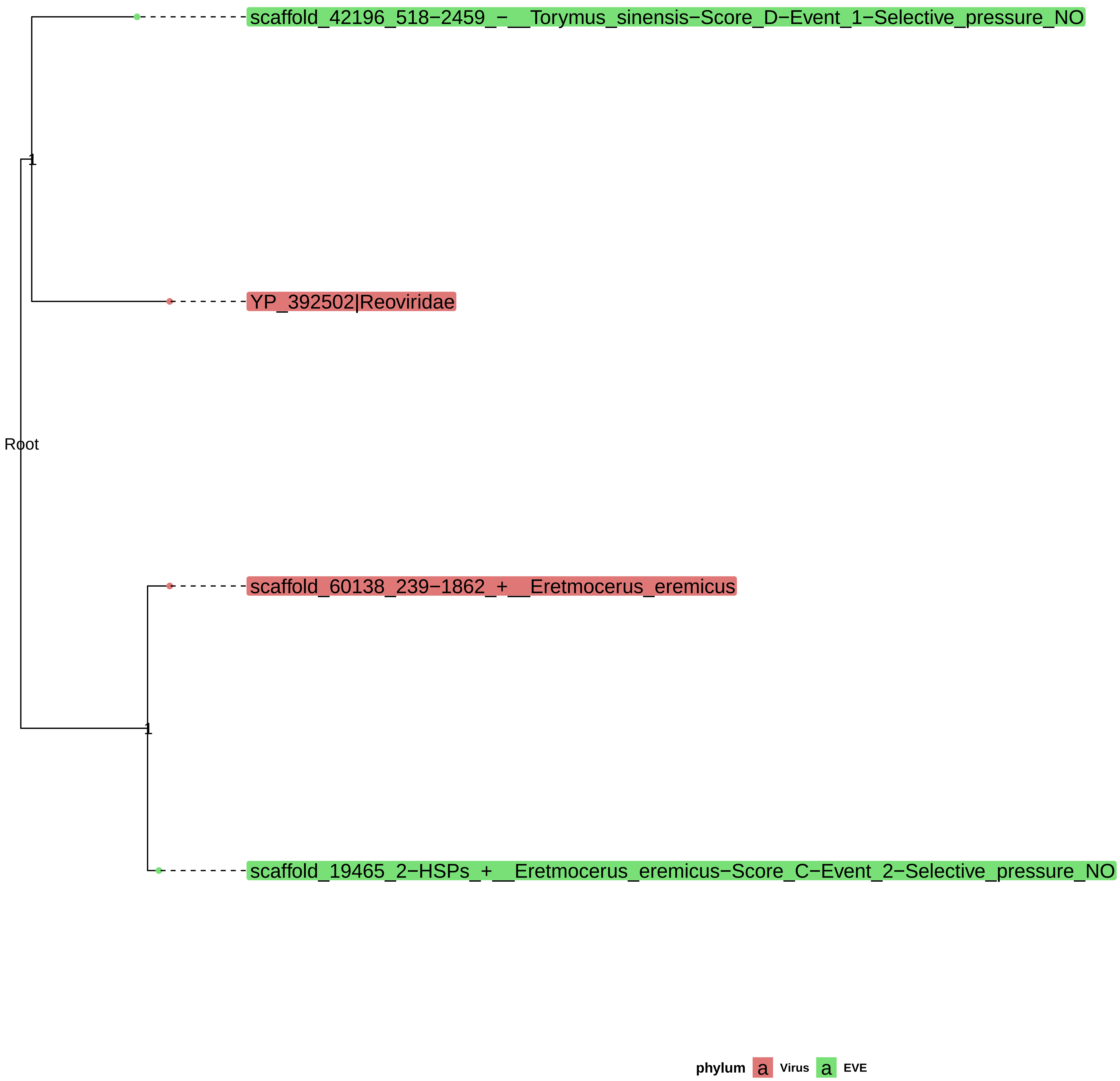

Cluster11235 − families involved : ['Reoviridae'] − putative protein : ['RNA−directed RNA polymerase (EC 2.7.7.48)']

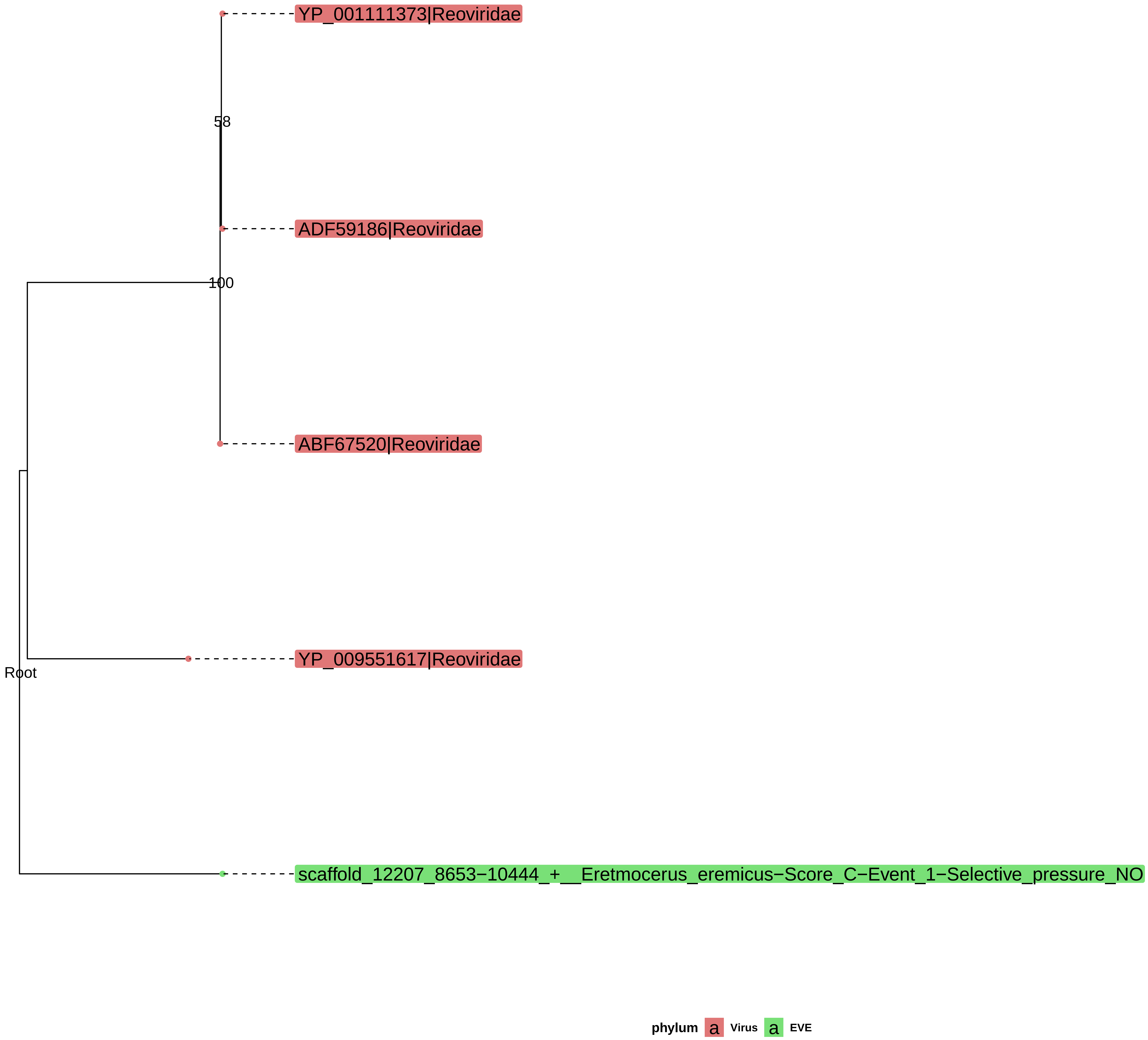

Cluster4724 − families involved : ['Iridoviridae'] − putative protein : ['SCP−2 sterol transfer family']

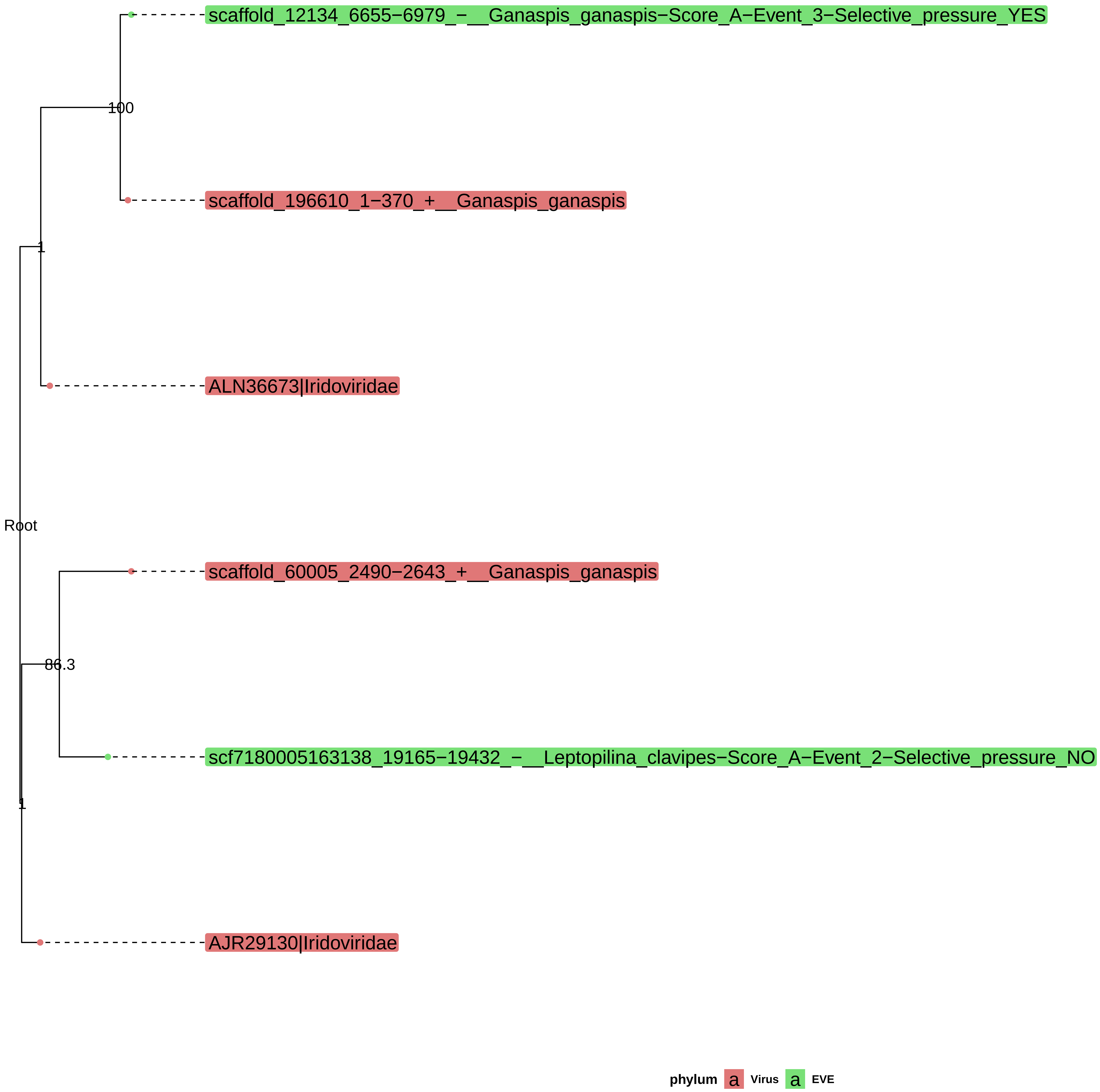

Cluster11270 − families involved : ['Iridoviridae'] − putative protein : ['Domain of unknown function (DUF1965)']

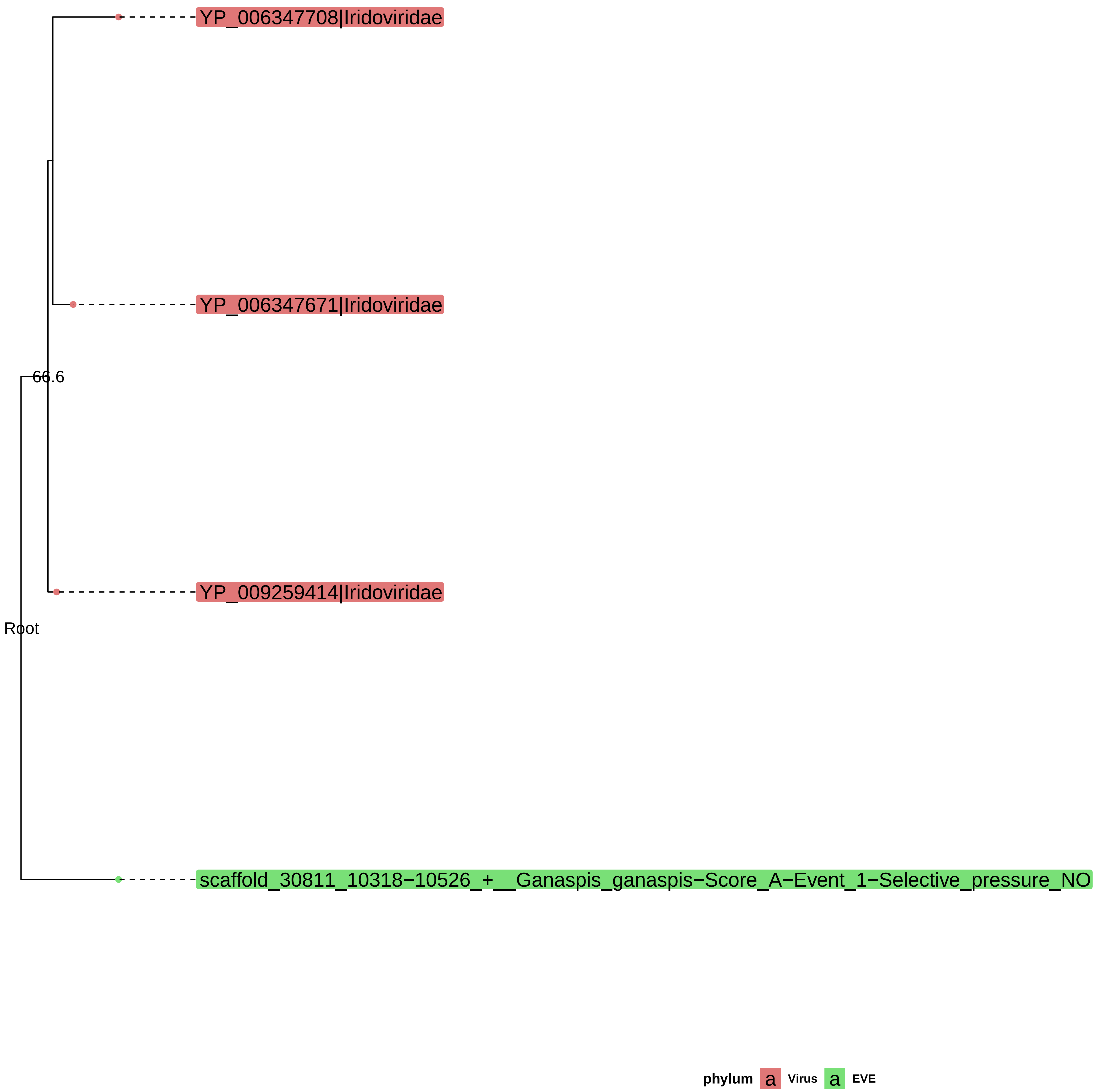

Cluster11161 − families involved : ['Iridoviridae', 'Phycodnaviridae'] − putative protein : ['YLP motif']

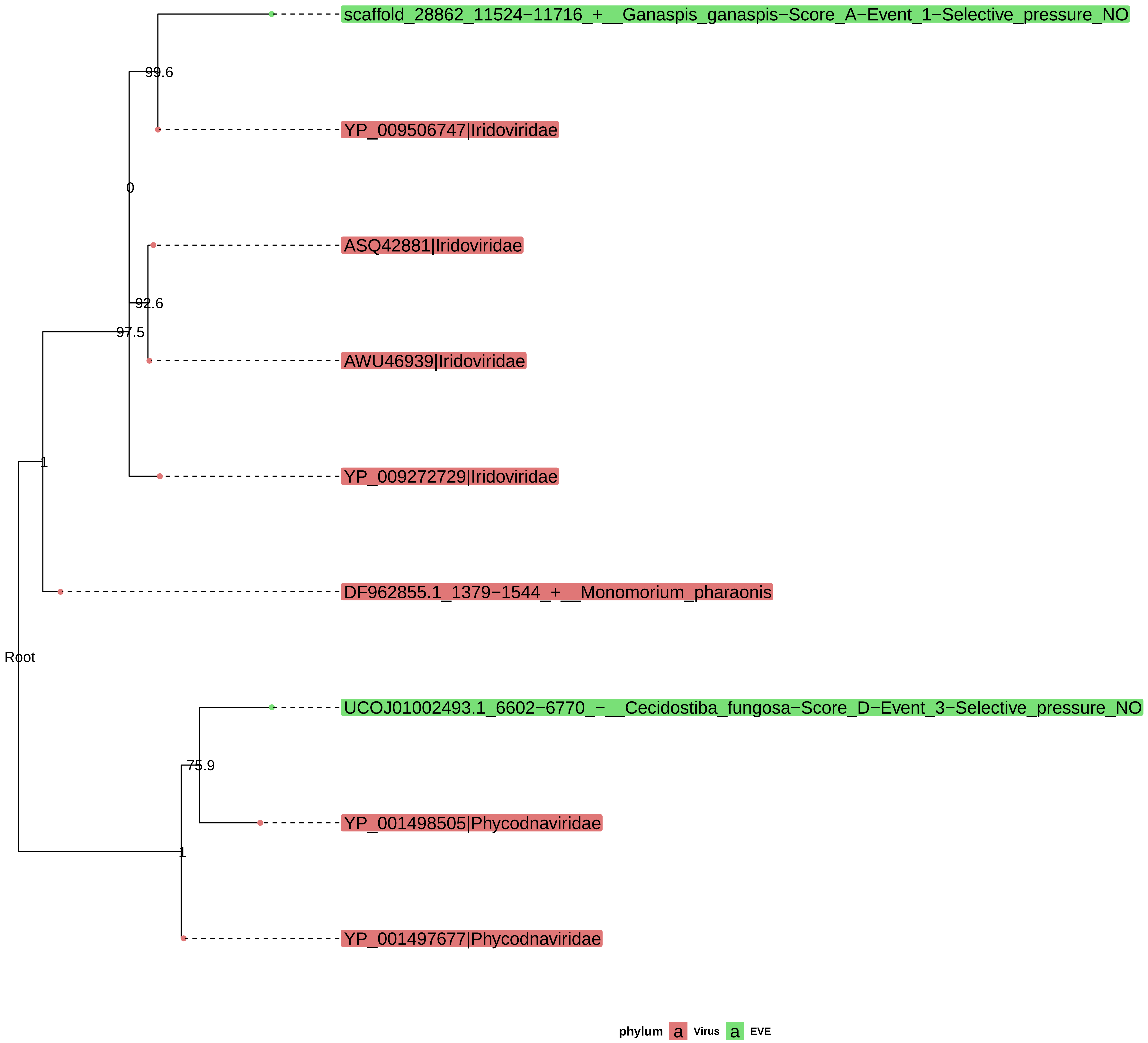

Cluster8206 − families involved : ['Nudiviridae'] − putative protein : ['ACH96209.1 GrBNV gp59−like protein']

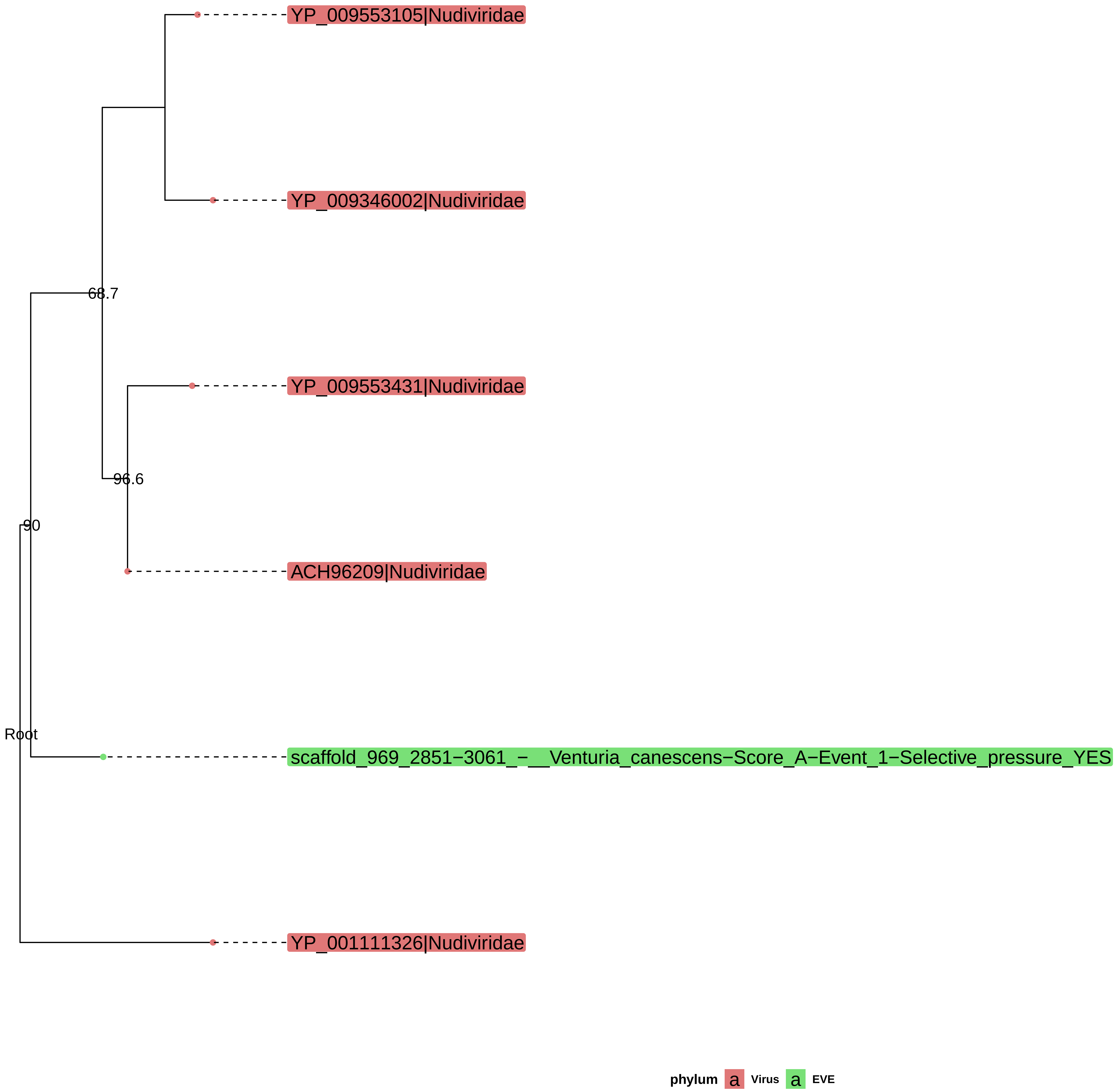

Cluster27243 − families involved : ['Nudiviridae'] − putative protein : ['ACH96171.1 GrBNV gp95−like protein (Putative gp95−like protein)']

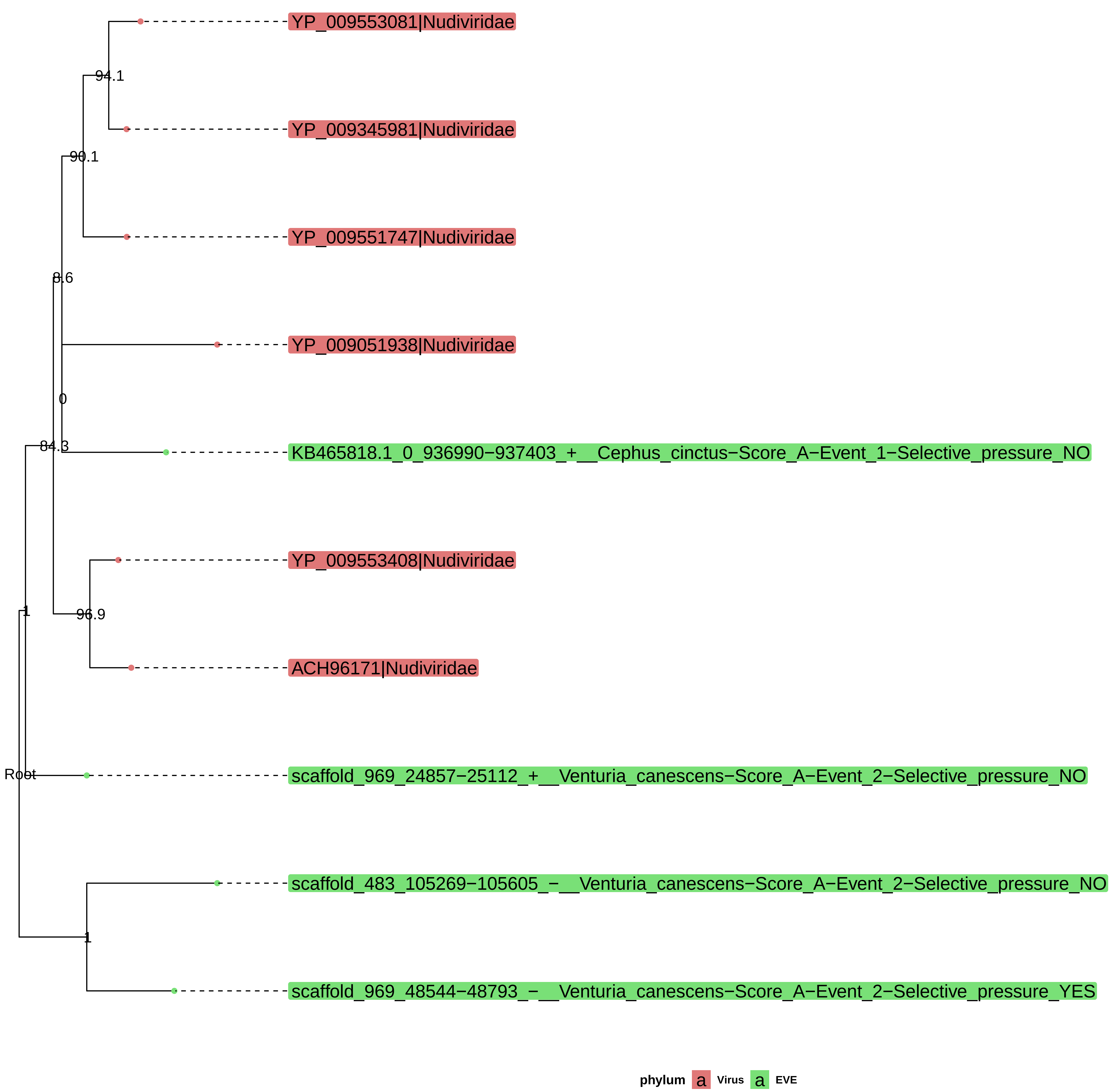

Cluster21249 − families involved : ['Nudiviridae'] − putative protein : ['ACH96176.1 GrBNV gp22 and 23−like protein']

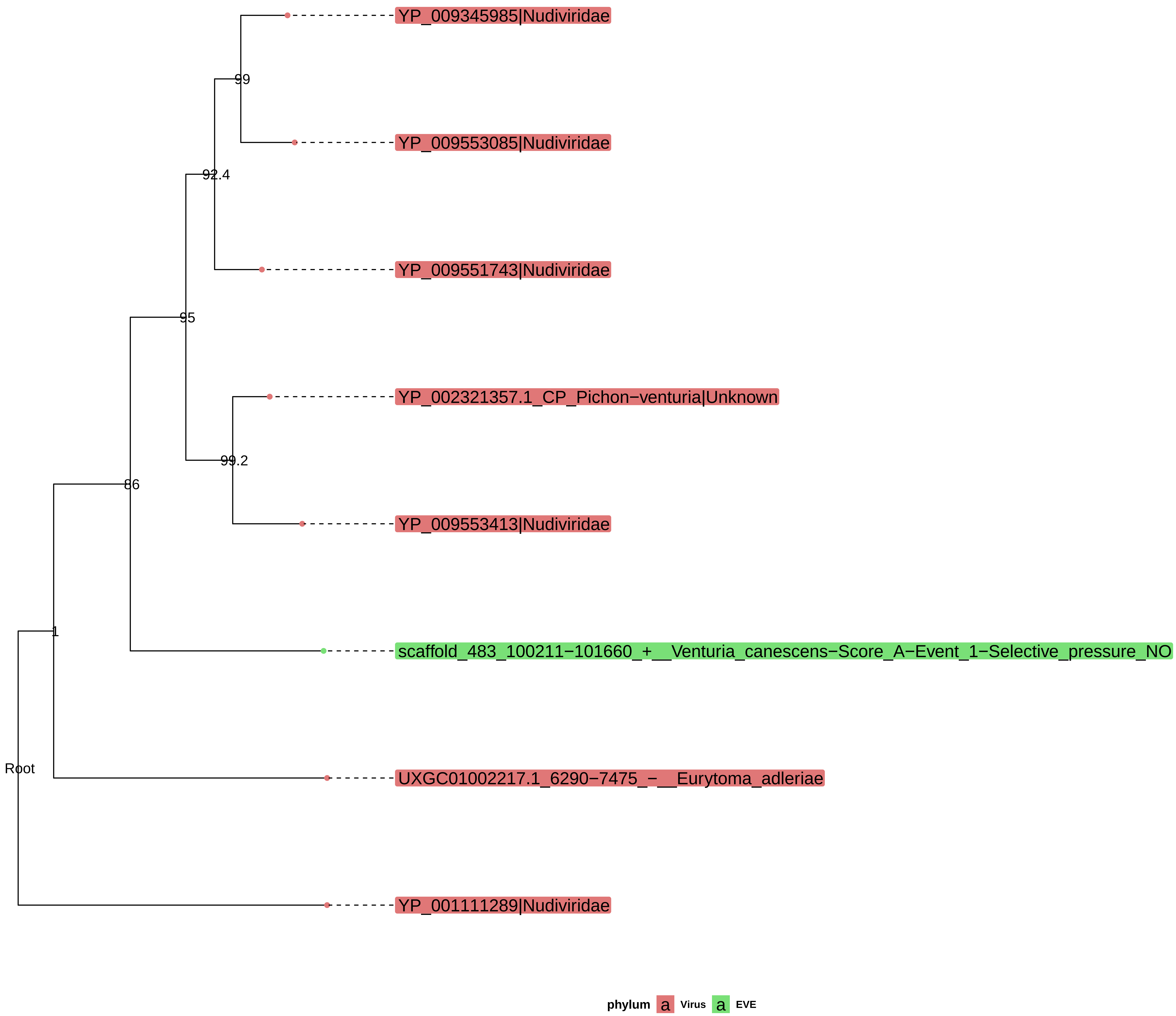

Cluster18176 − families involved : ['Nudiviridae'] − putative protein : ['GrBNV_gp83−like protein']

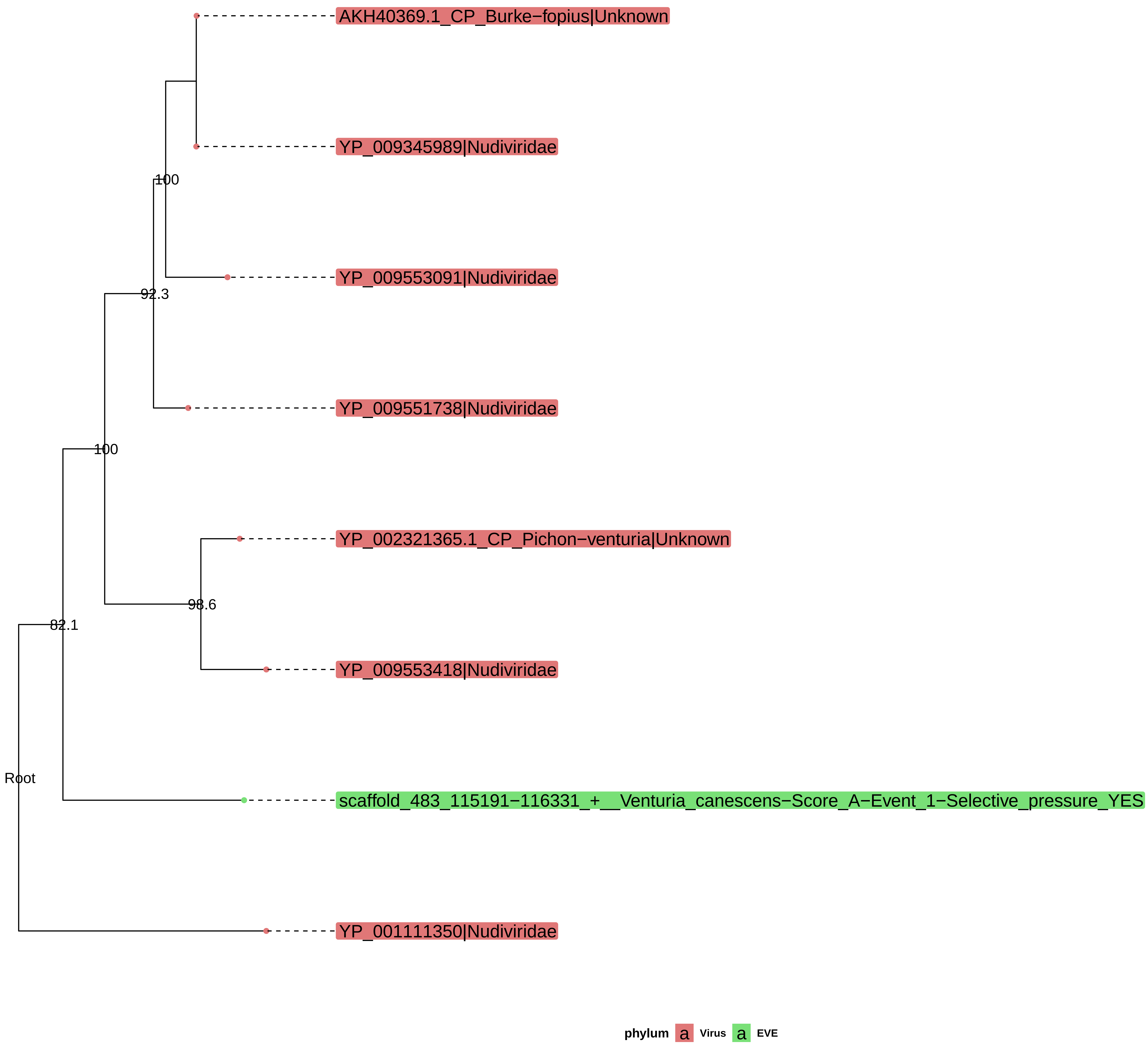

Cluster14604 − families involved : ['Nudiviridae'] − putative protein : ['Putative guanylate kinase']

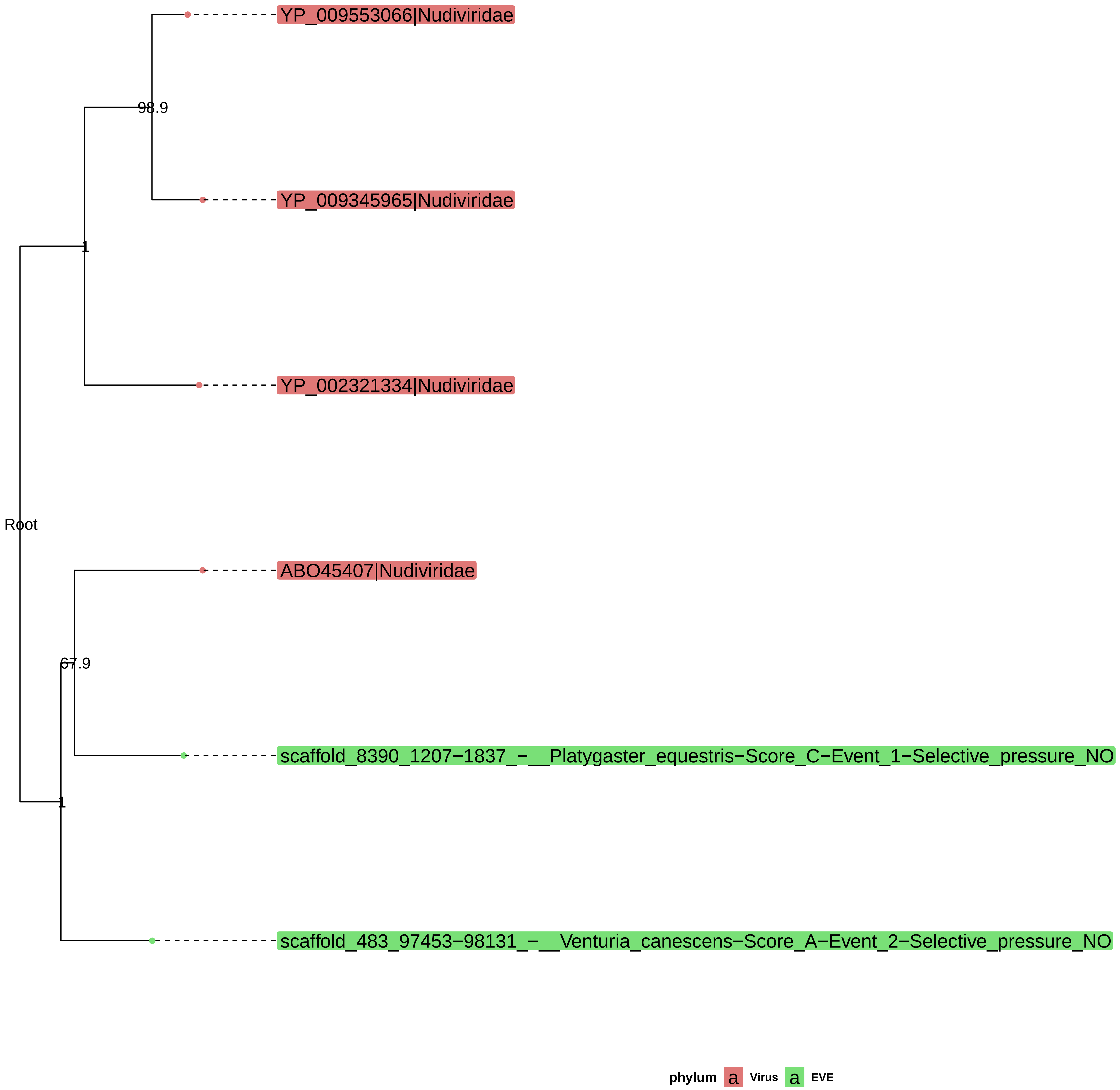

Cluster13641 − families involved : ['Nudiviridae'] − putative protein : ['Uncharacterized protein']

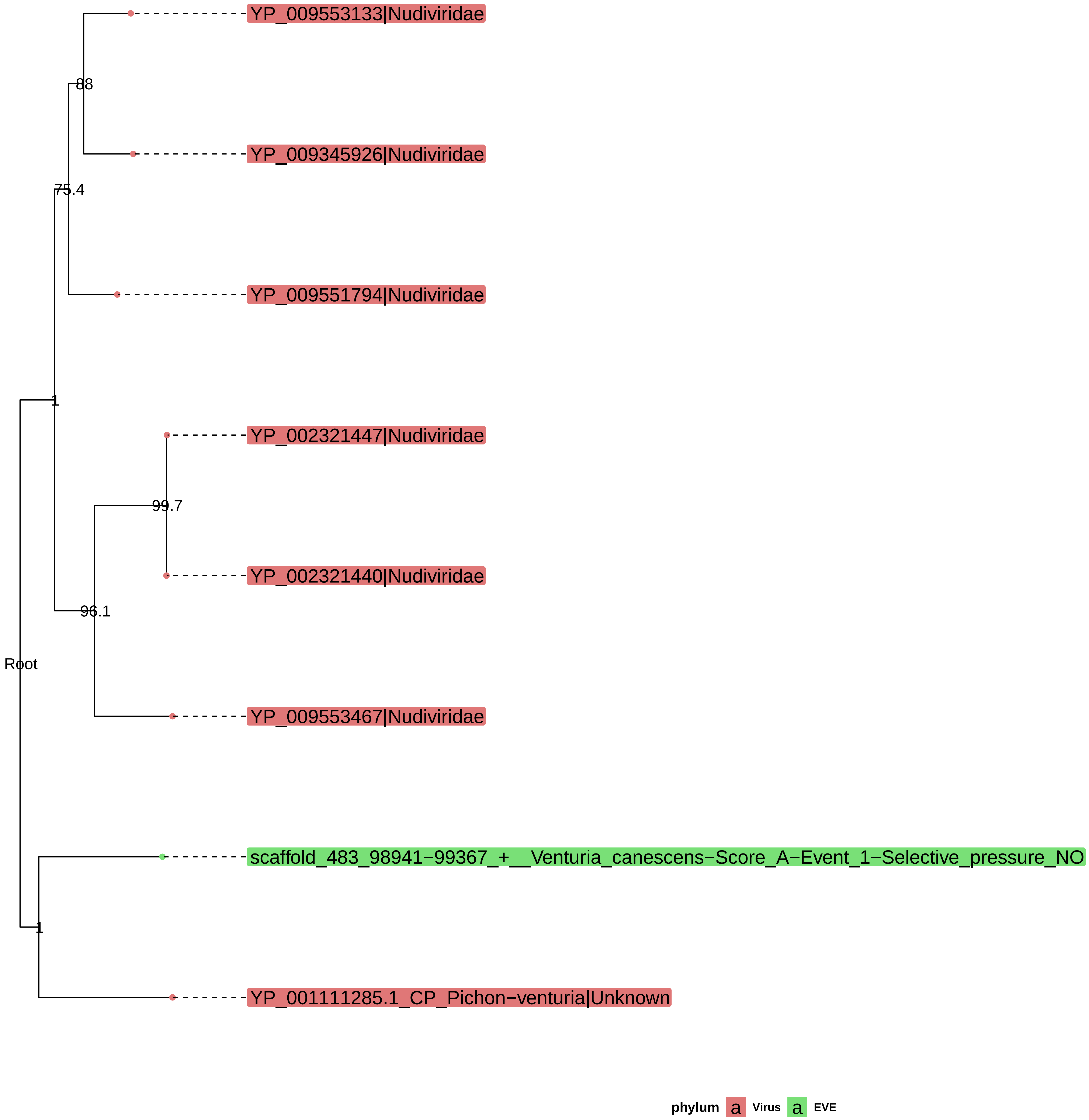

Cluster10827 − families involved : ['Lispiviridae', nan] − putative protein : ['Putative glycoprotein', nan]

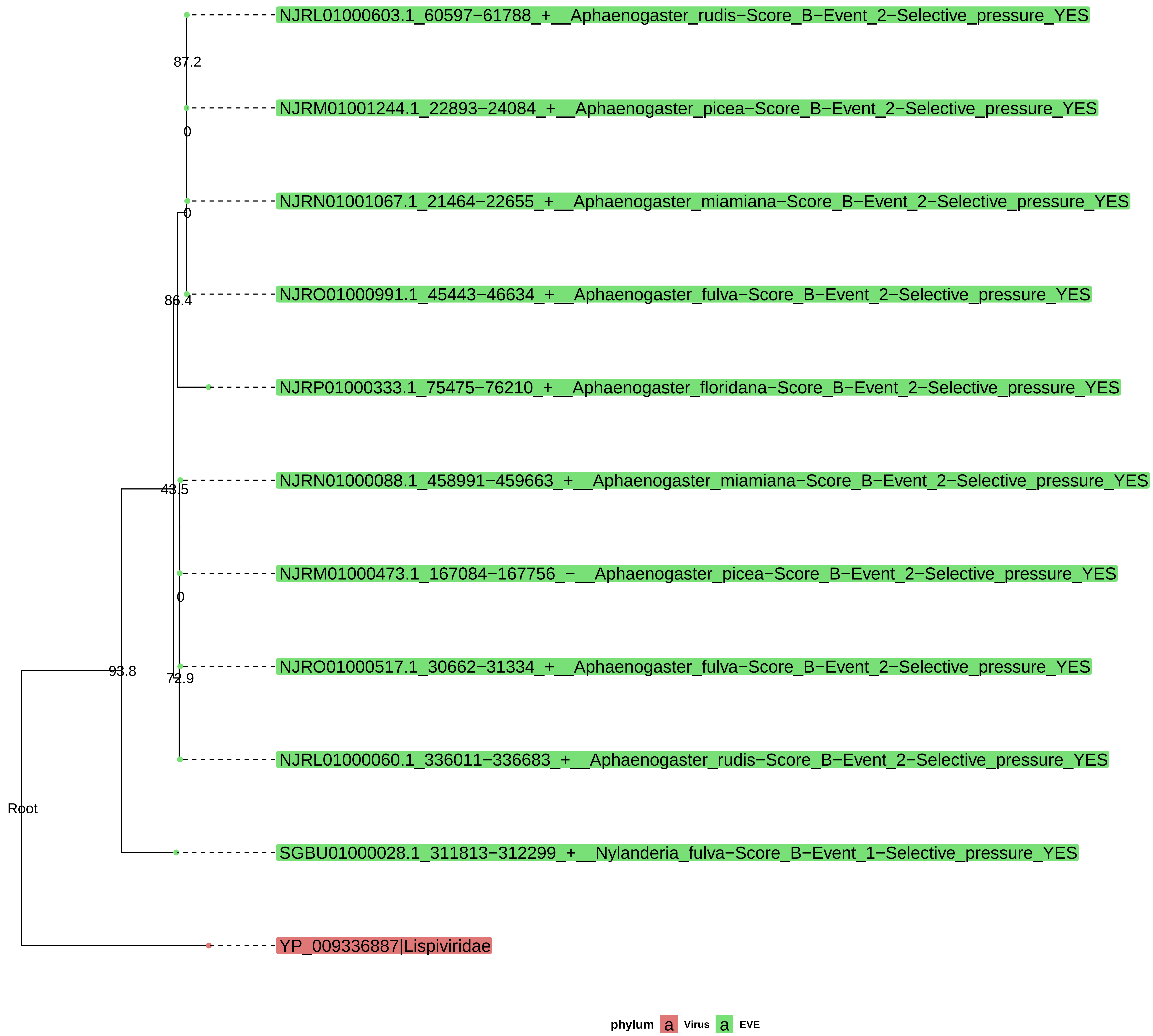

Cluster5910 − families involved : ['IVSPERs'] − putative protein : [nan]

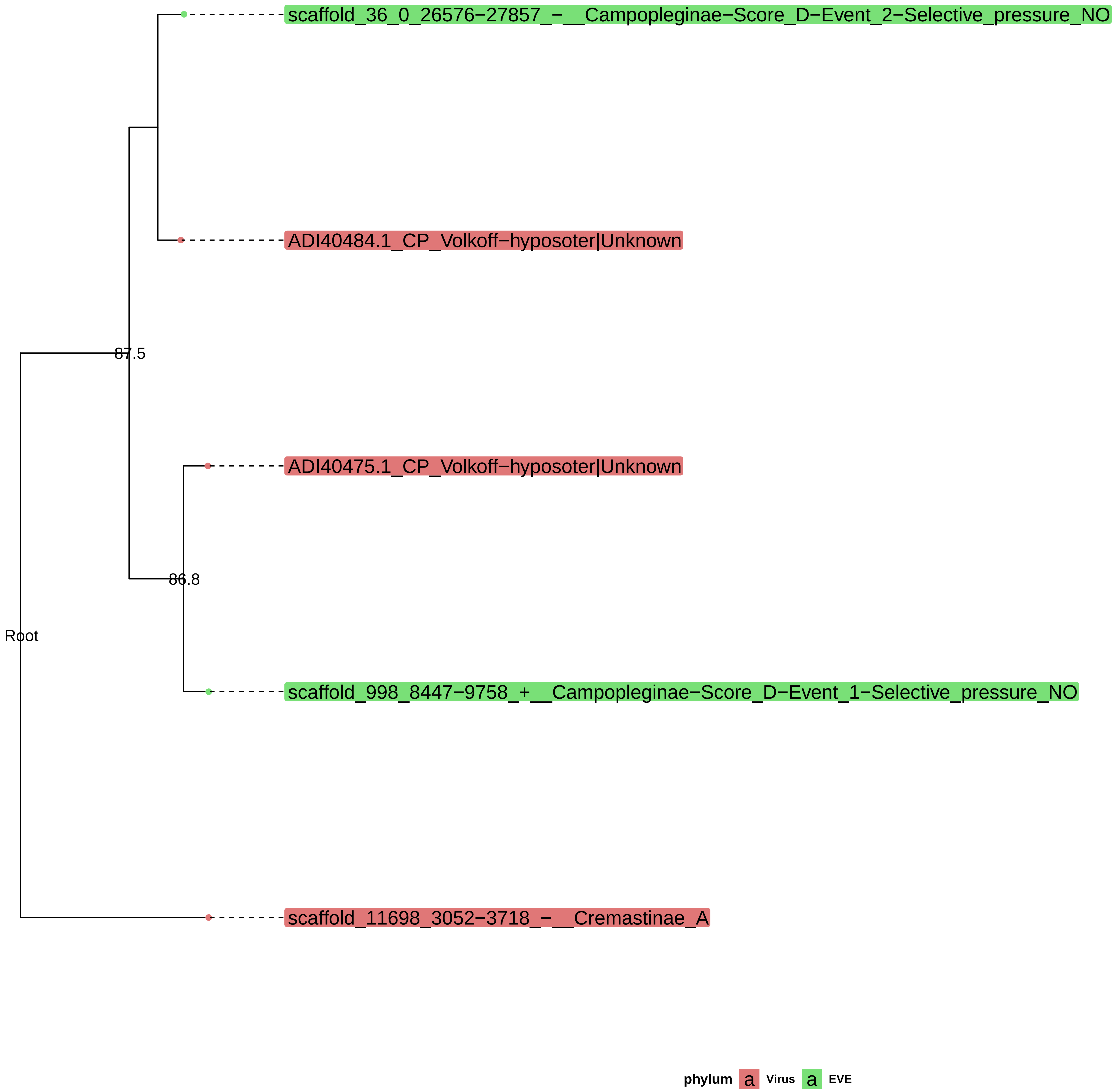

Cluster3513 − families involved : ['IVSPERs', 'Nudiviridae'] − putative protein : ['Rep−like protein']

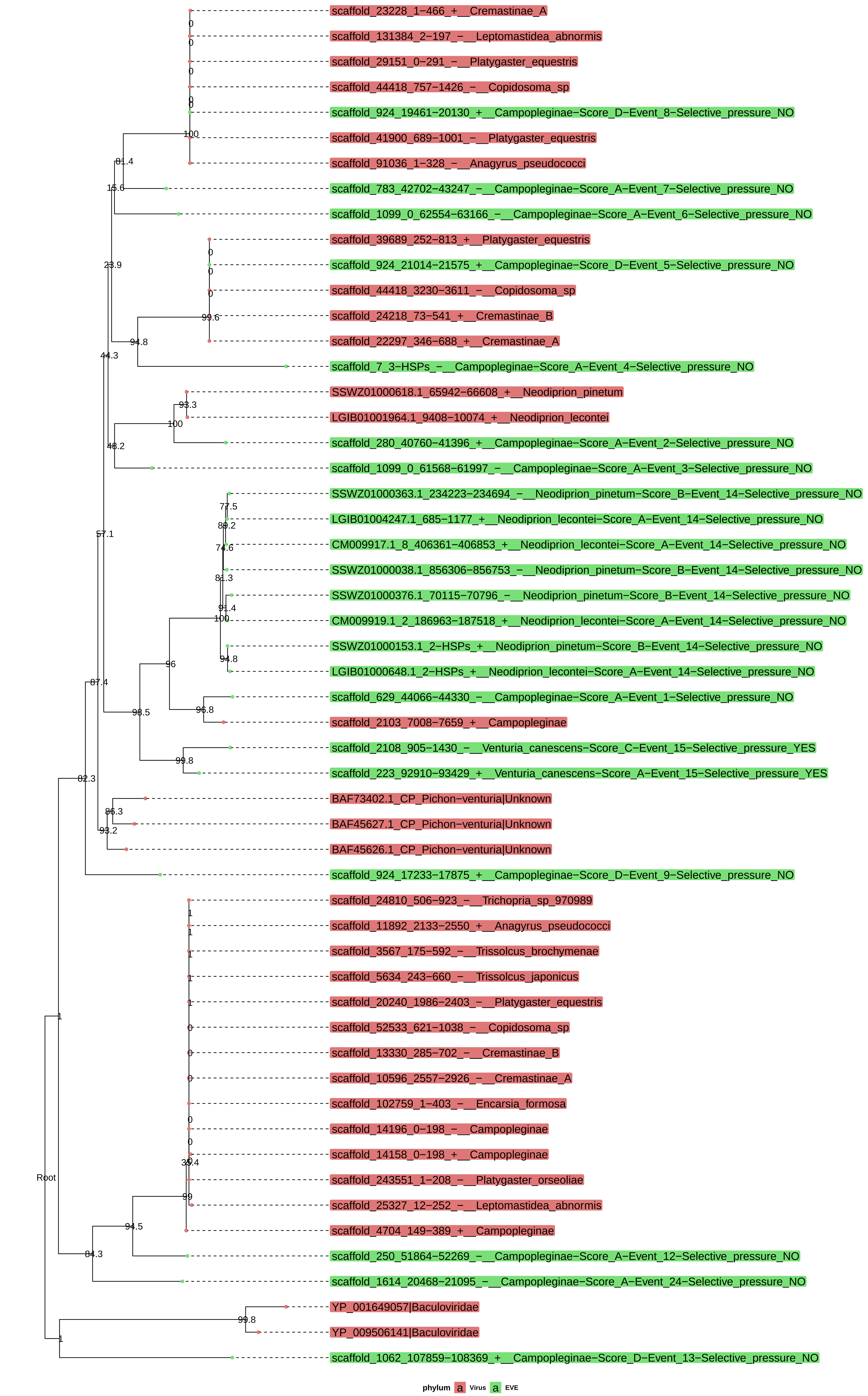

Cluster24594 − families involved : ['IVSPERs'] − putative protein : [nan]

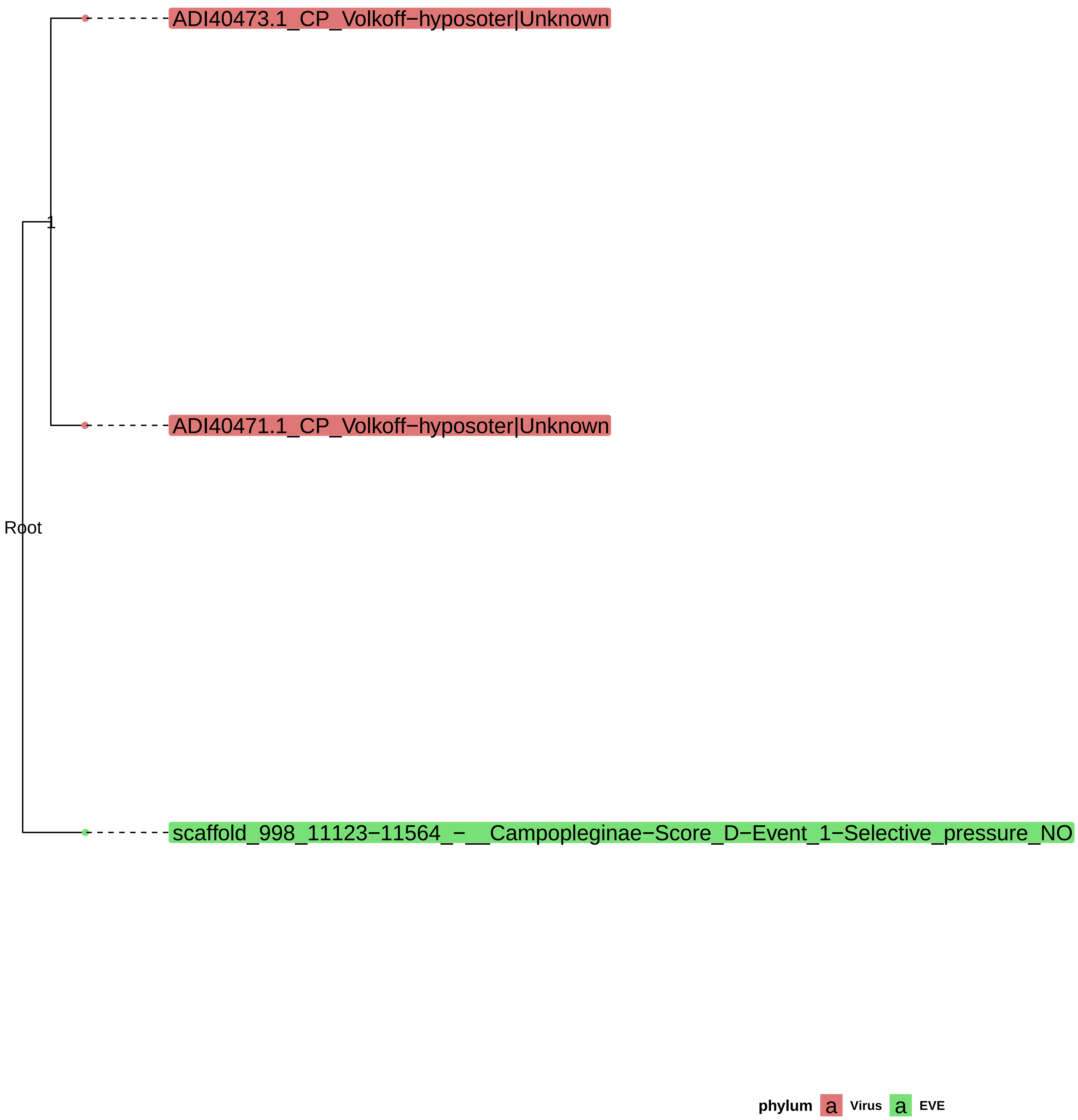

Cluster24593 − families involved : ['IVSPERs'] − putative protein : ["Cytochrome C'"]

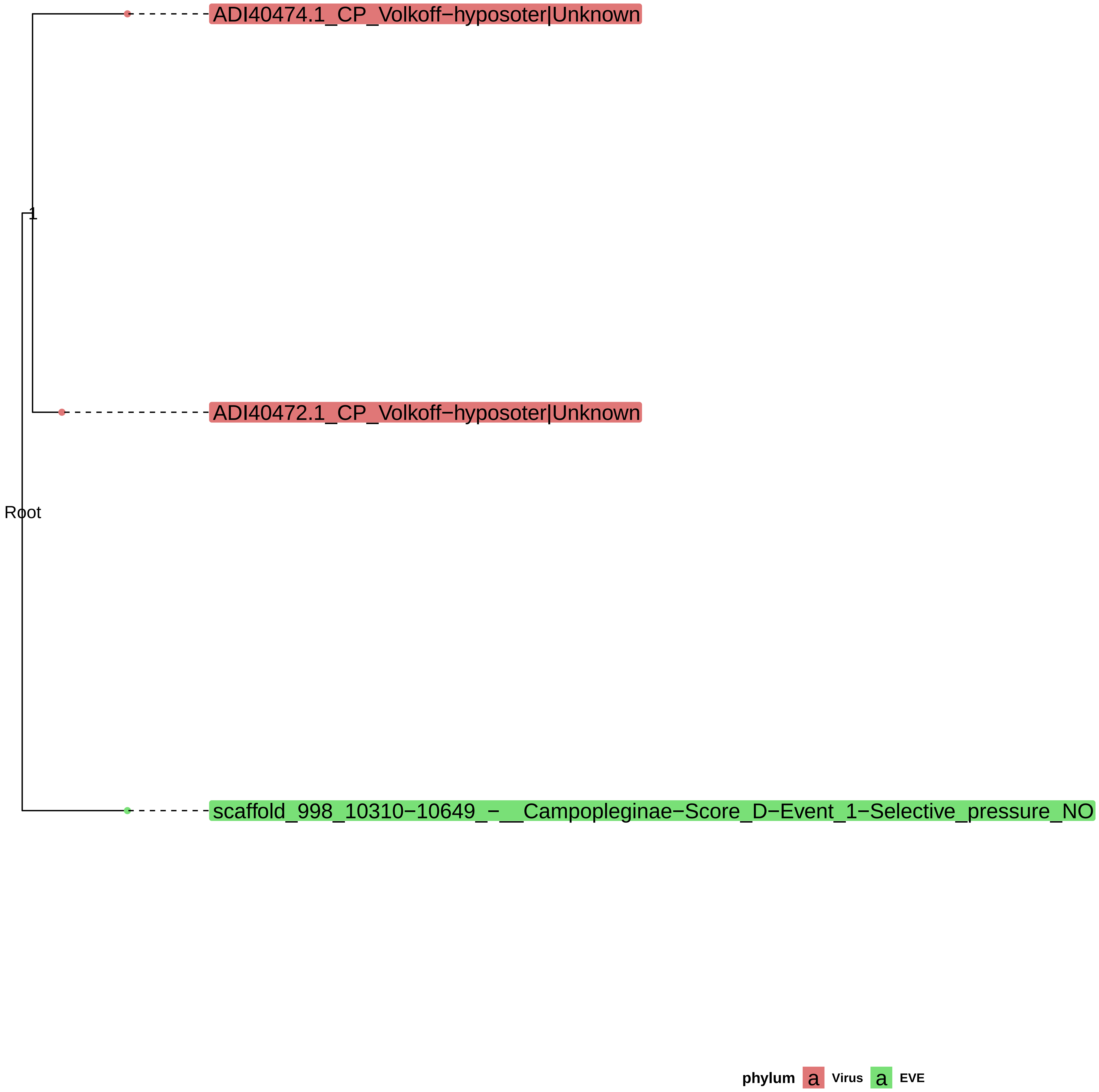

Cluster22765 − families involved : ['IVSPERs'] − putative protein : ['Baculoviridae P74 N−terminal']

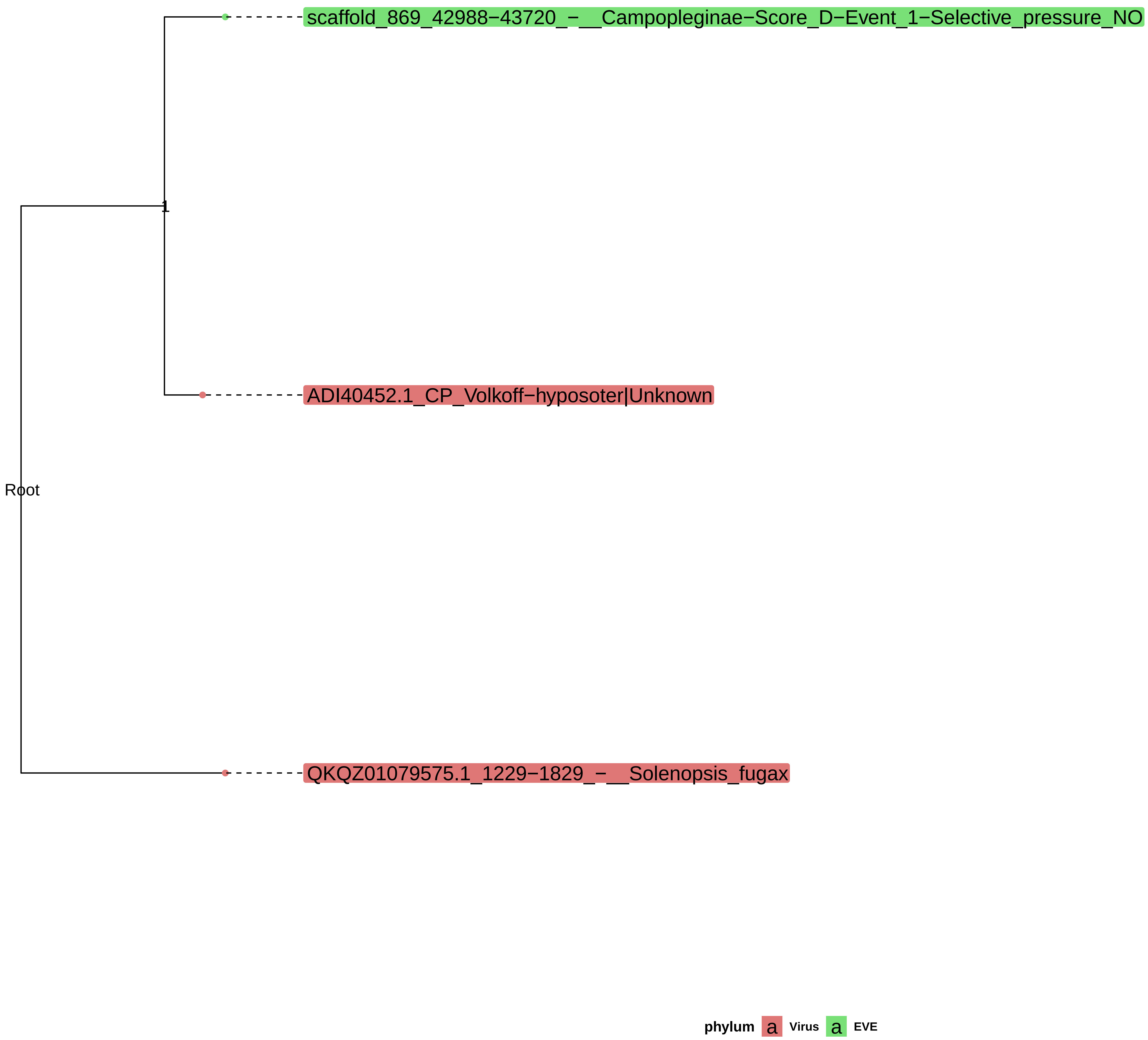

Cluster20806 − families involved : ['IVSPERs'] − putative protein : ['Cholesterol oxidase, substrate−binding']

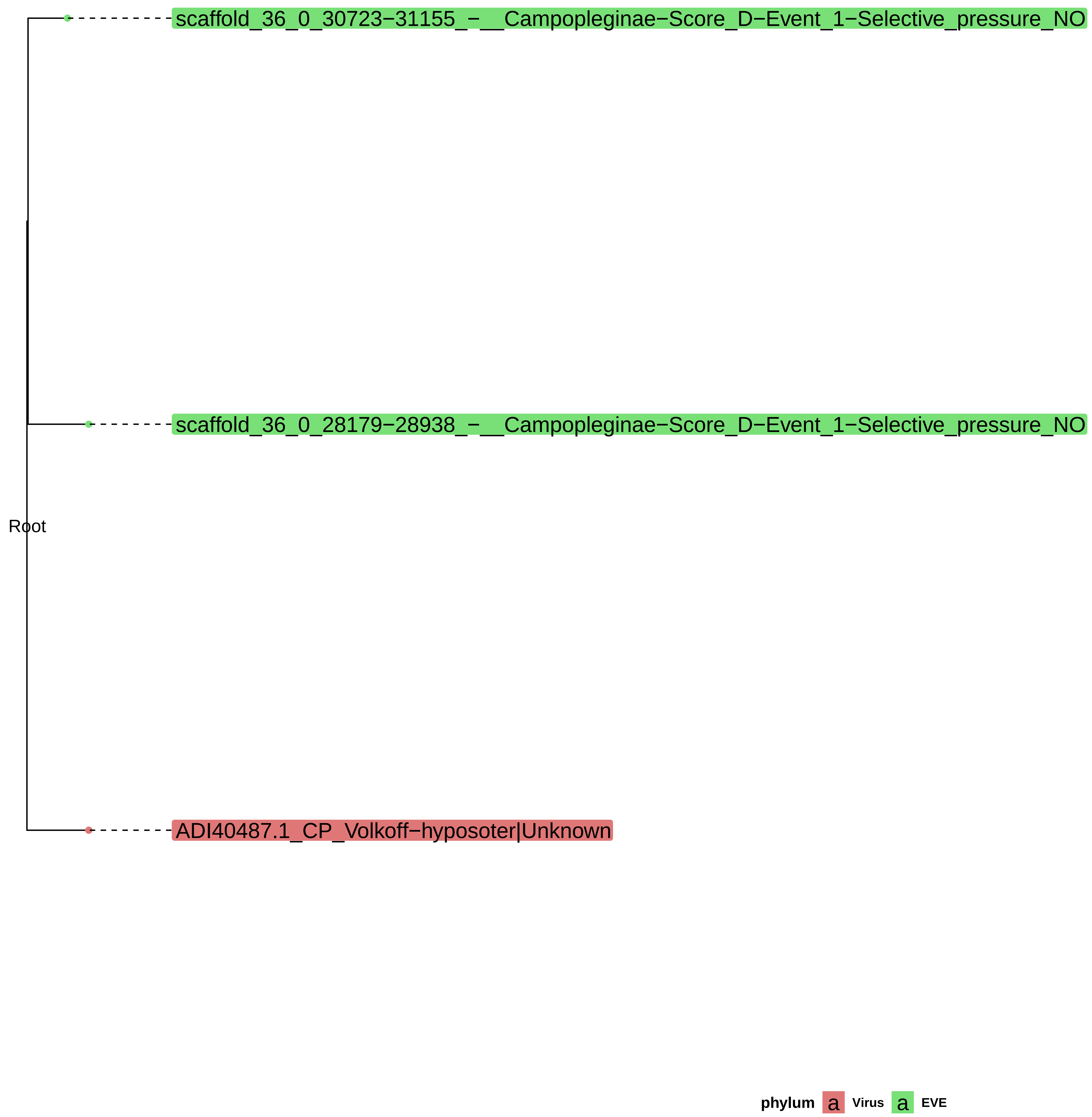

Cluster1828 − families involved : ['IVSPERs'] − putative protein : [nan]

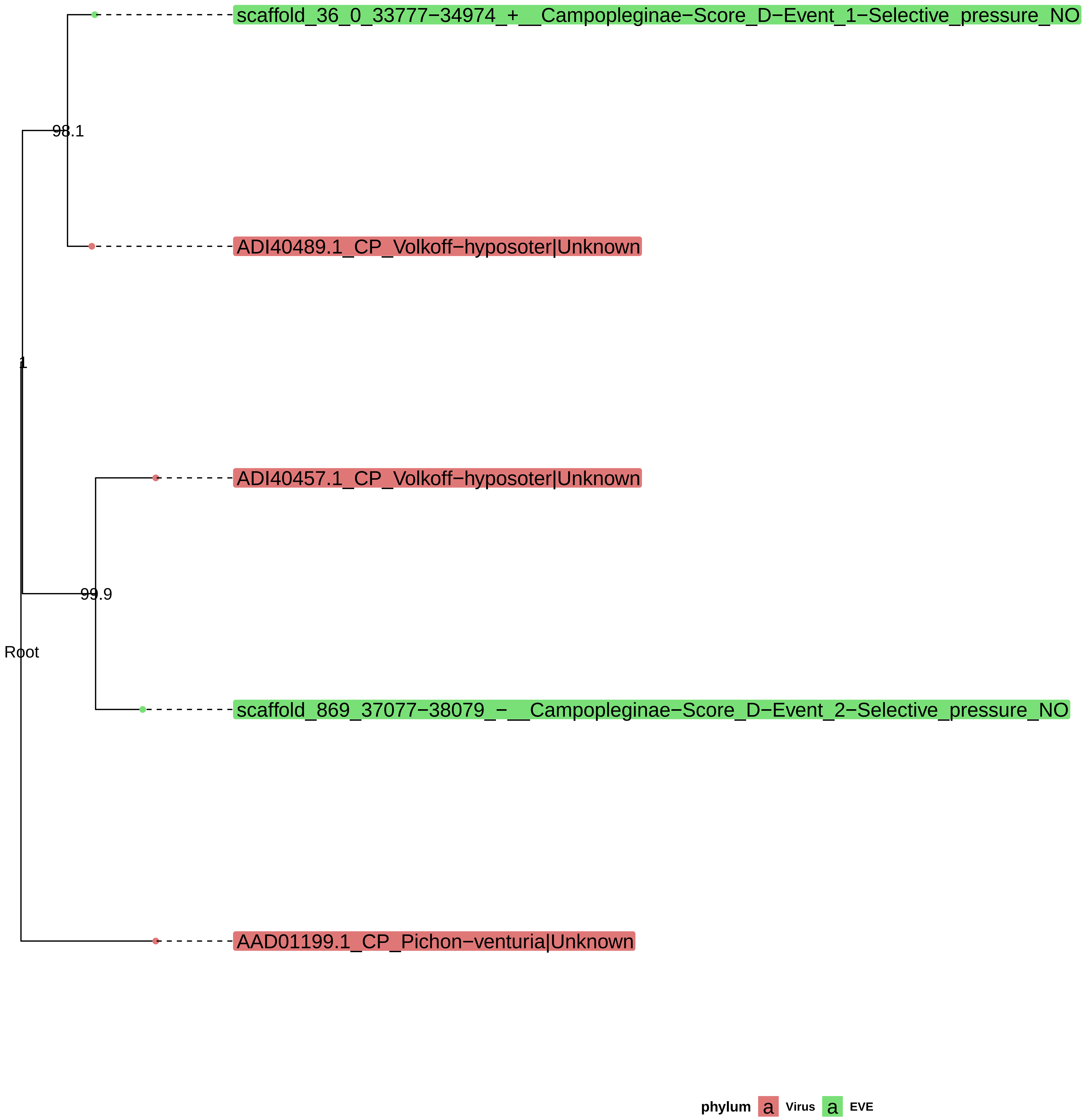

Cluster1826 − families involved : ['IVSPERs'] − putative protein : [nan]

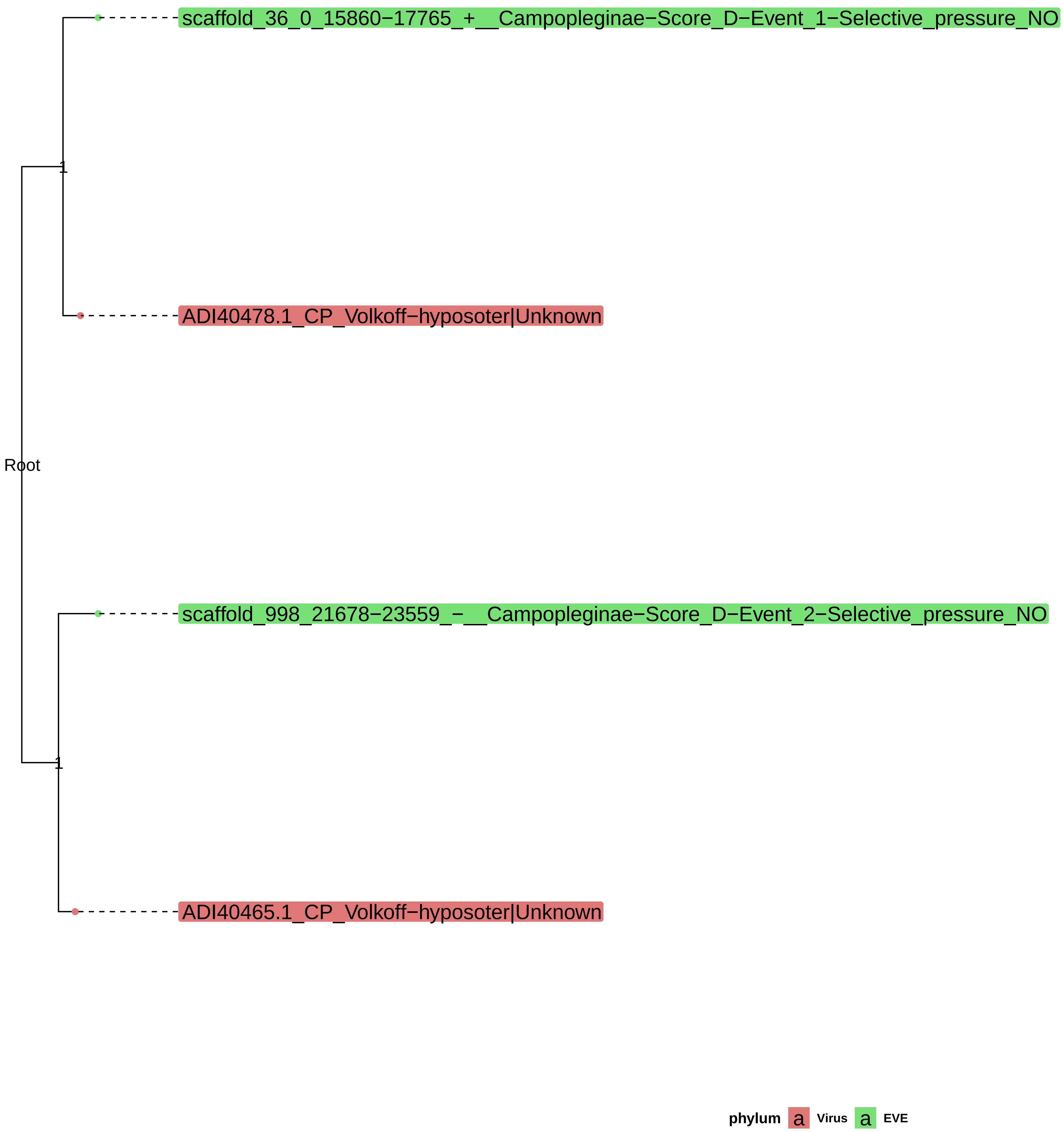

Cluster17553 − families involved : ['IVSPERs'] − putative protein : ['Protein of unknown function (DUF2524)']

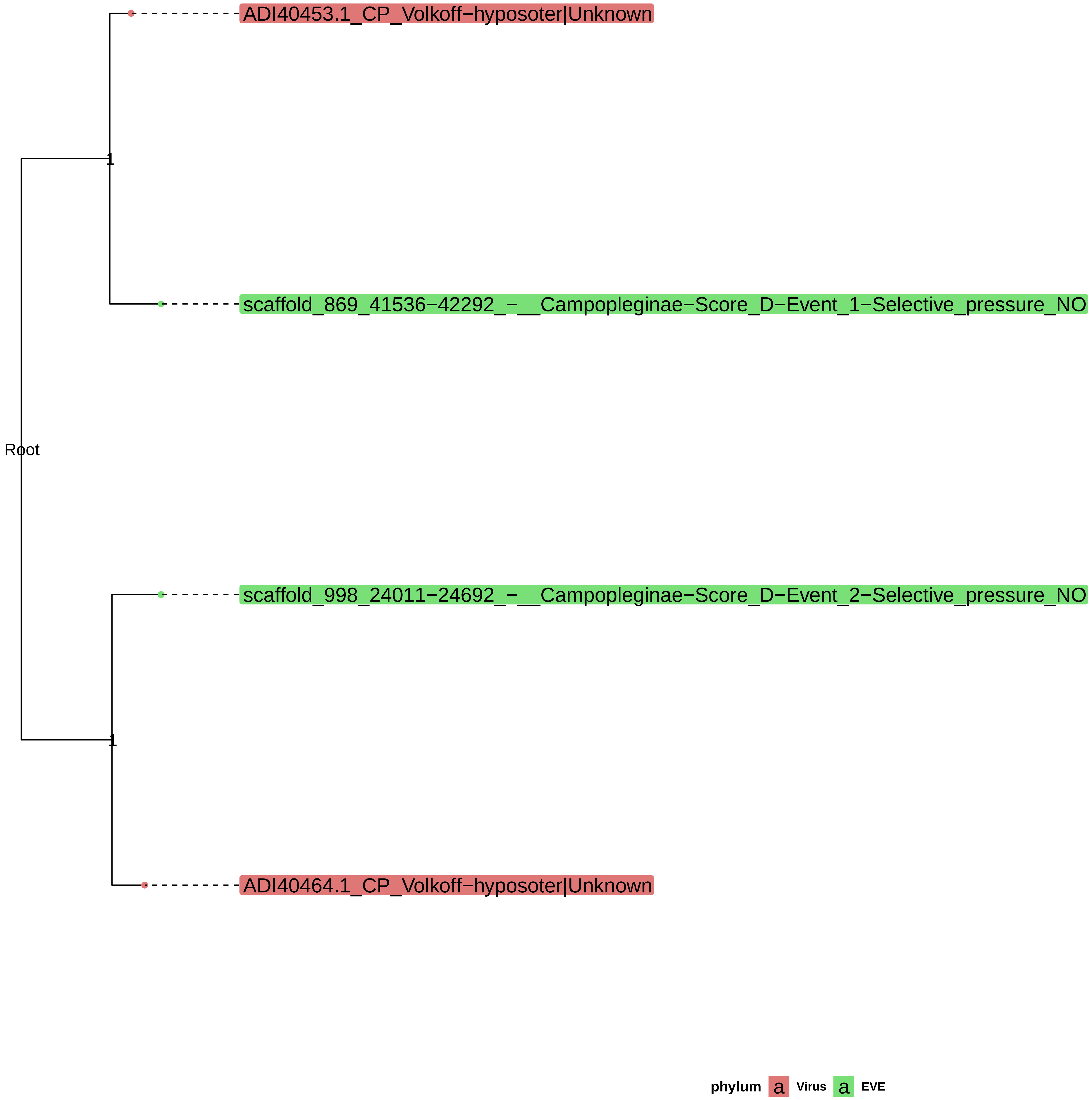

Cluster16867 − families involved : ['IVSPERs', 'Nudiviridae'] − putative protein : ['Glycosyl hydrolase family 67 C−terminus']

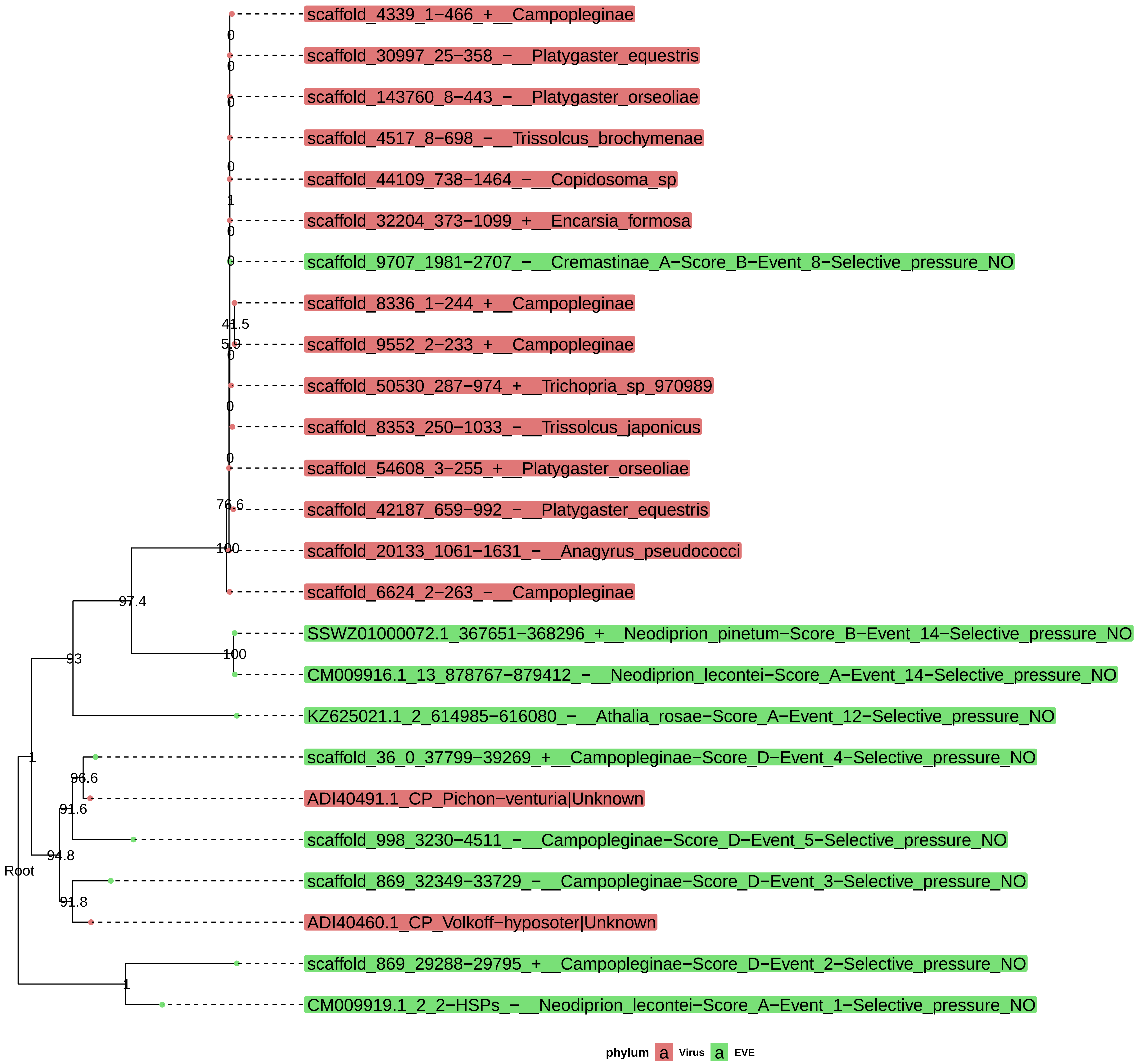

Cluster11128 − families involved : ['IVSPERs'] − putative protein : [nan]

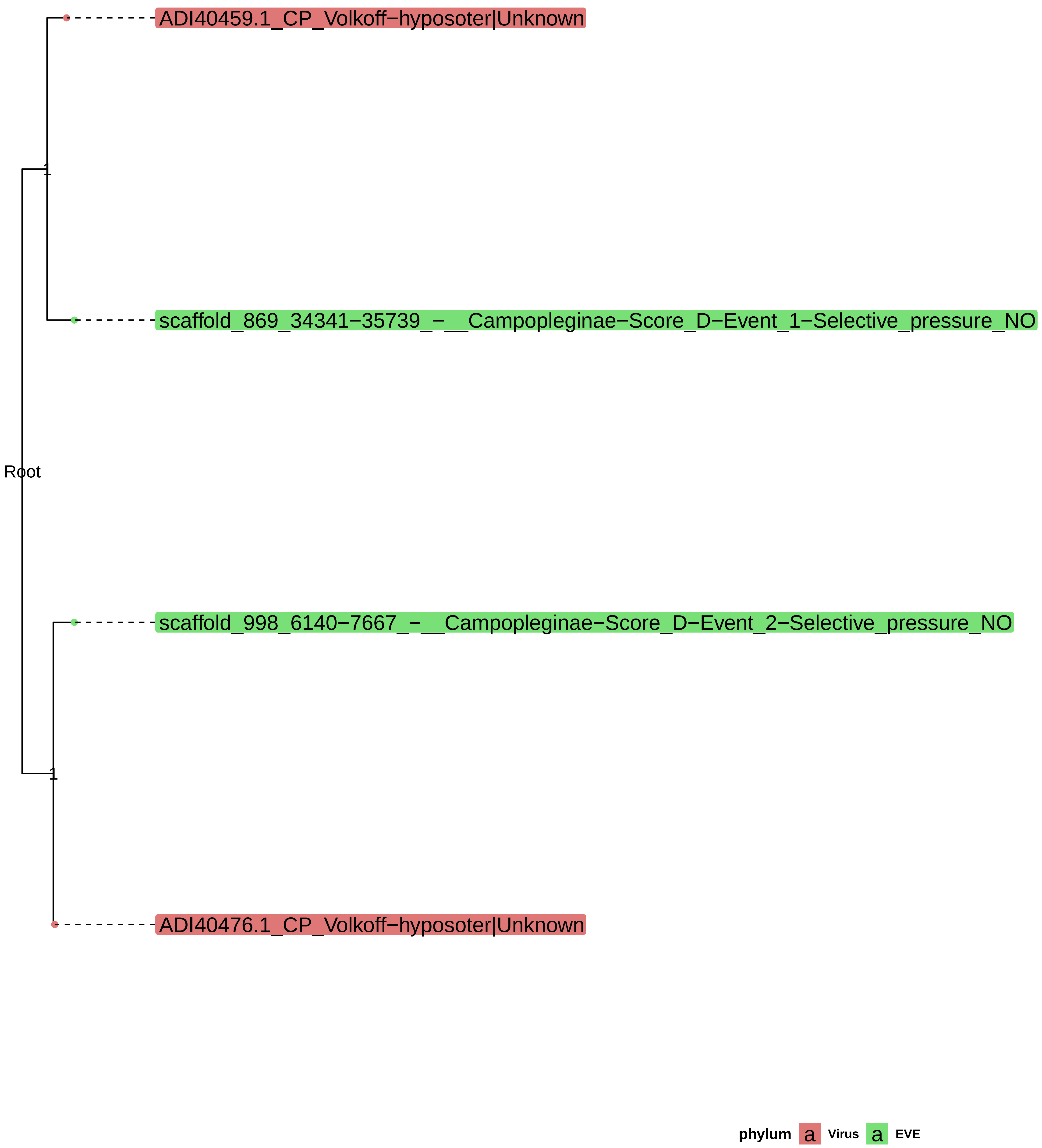

Cluster5559 − families involved : ['LbFV_like'] − putative protein : ['Uncharacterized protein']

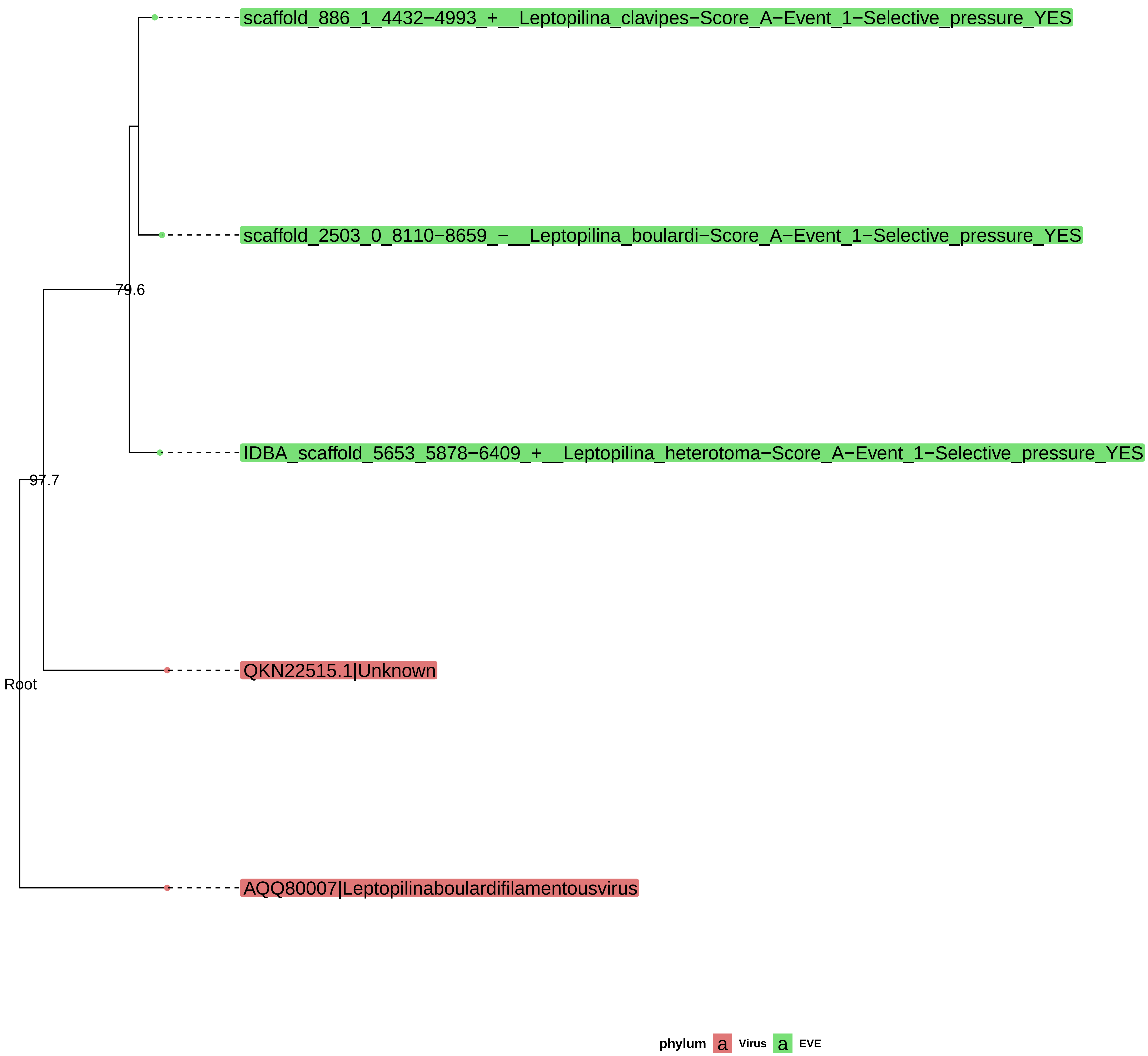

Cluster27528 − families involved : ['LbFV_like'] − putative protein : ['Uncharacterized protein']

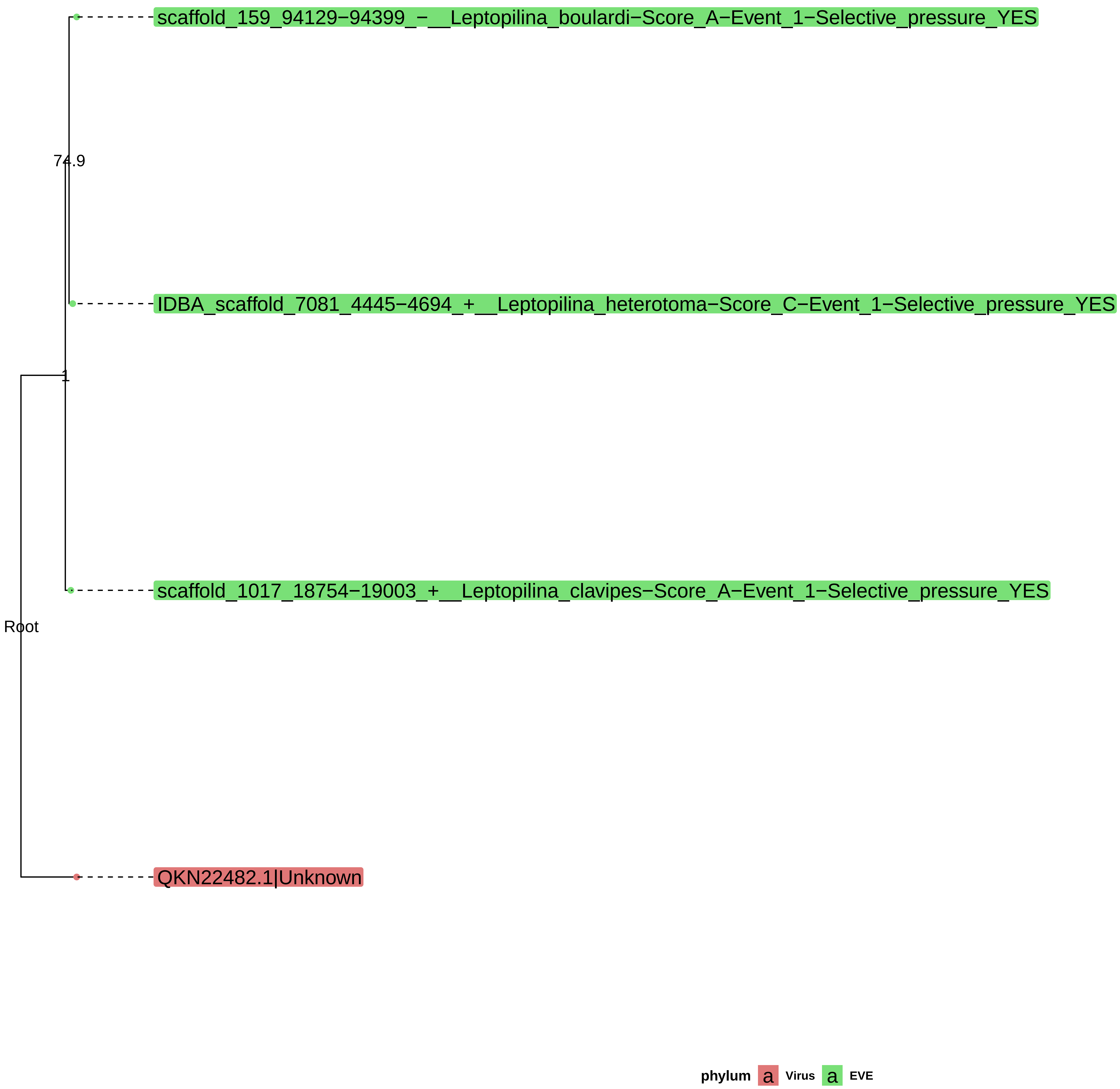

Cluster25244 − families involved : ['LbFV_like'] − putative protein : ['Bromodomain']

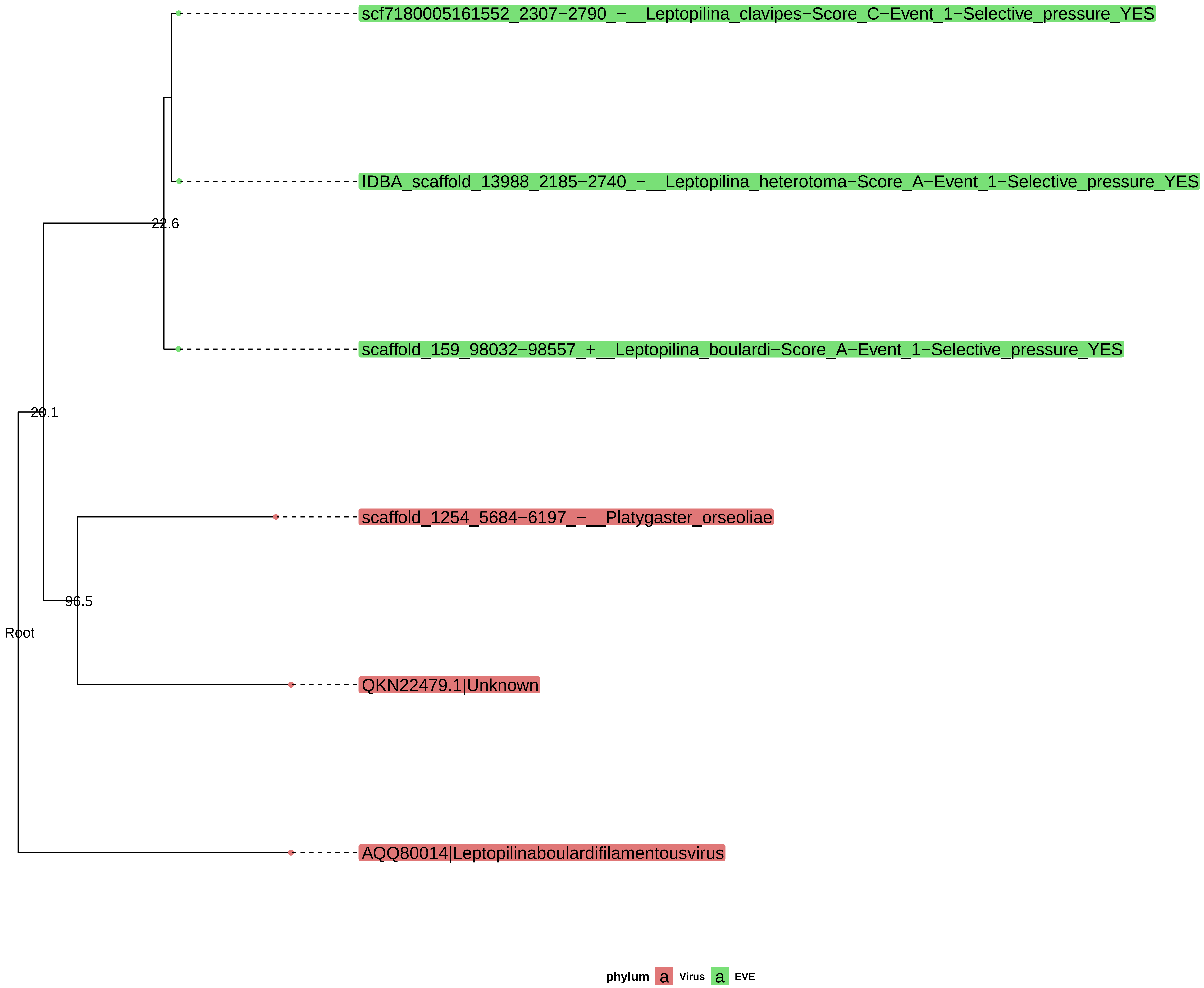

Cluster22585 − families involved : ['LbFV_like'] − putative protein : ['Putative ORF5']

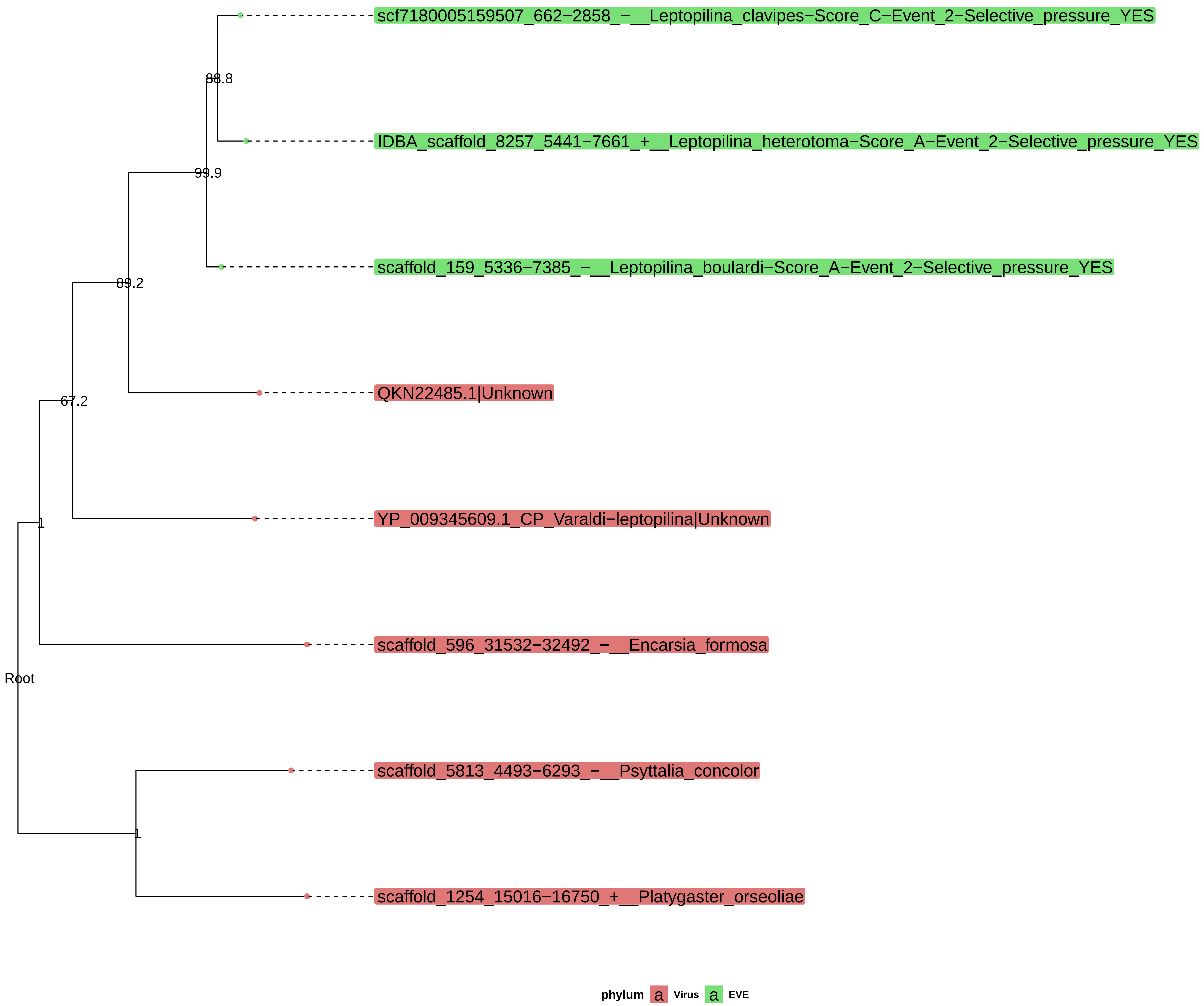

Cluster12272 − families involved : ['LbFV_like'] − putative protein : ['ATP cone domain']

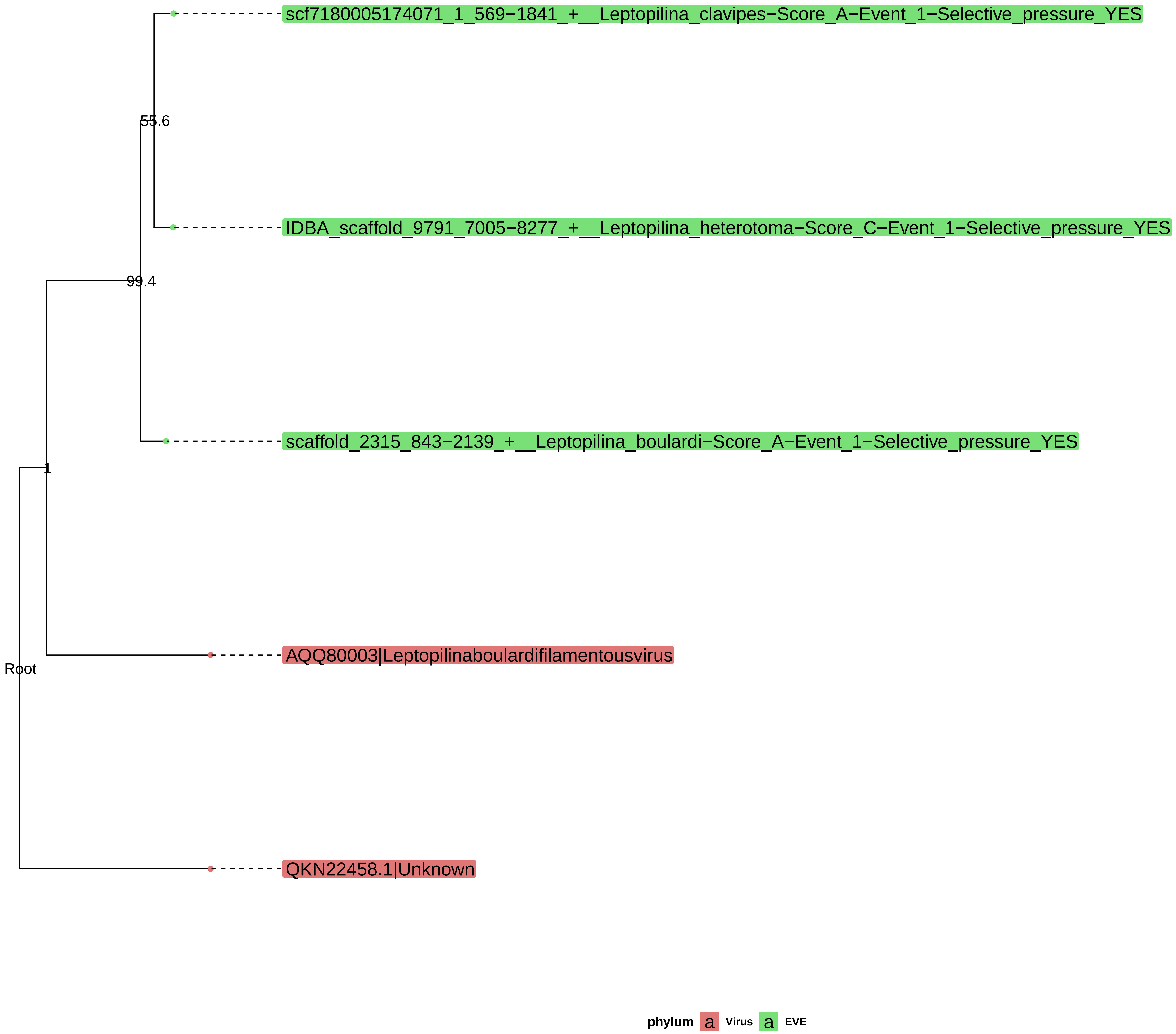

Cluster5563 − families involved : ['LbFV_like'] − putative protein : ['Erv1 / Alr family']

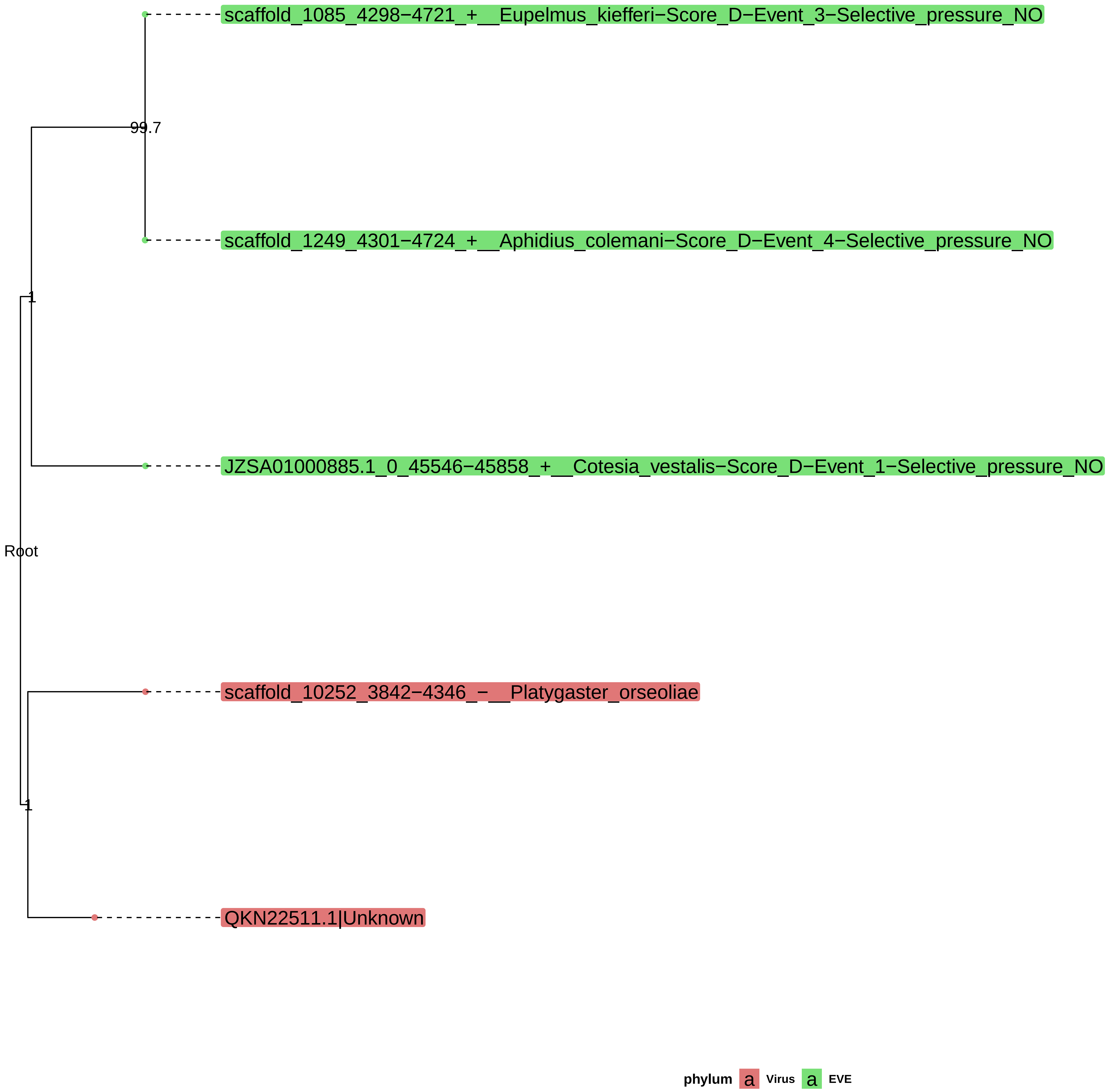

Cluster26024 − families involved : ['LbFV_like'] − putative protein : ['Hepatocyte growth factor−regulated tyrosine kinase substrate']

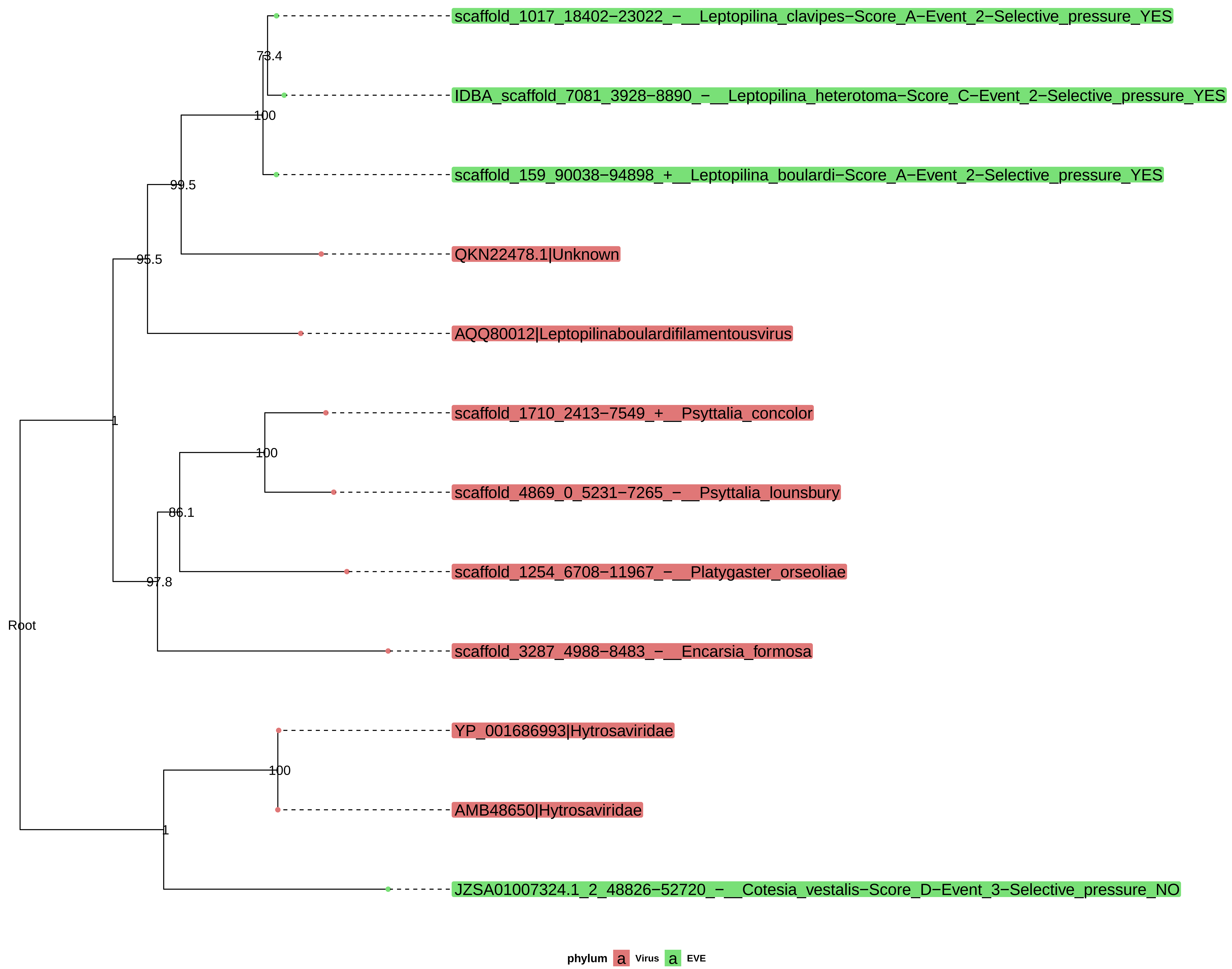

Cluster22219 − families involved : ['LbFV_like', 'Nudiviridae'] − putative protein : ['Helicase 2']

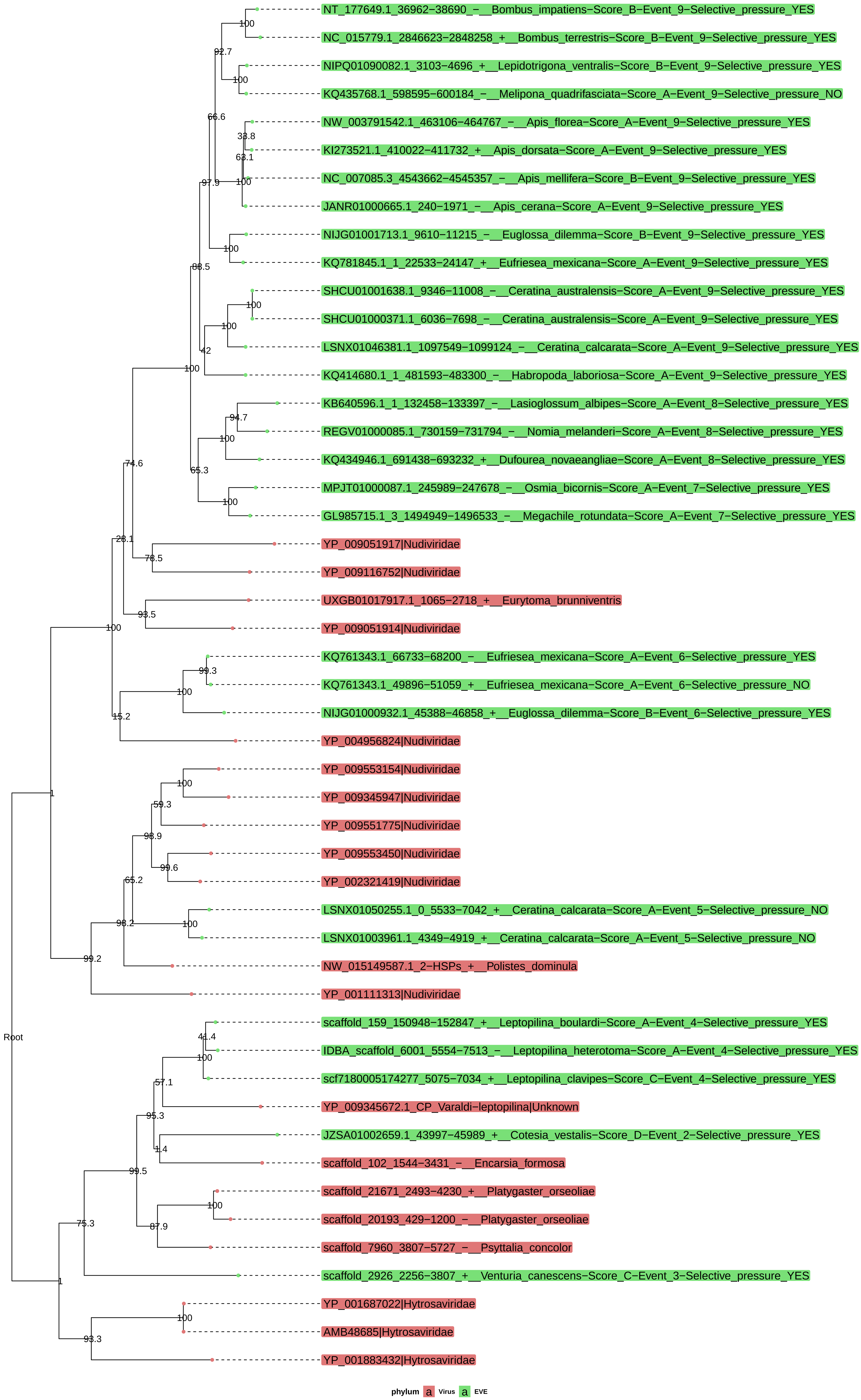

Cluster27041 − families involved : ['Nudiviridae'] − putative protein : ['VP39']

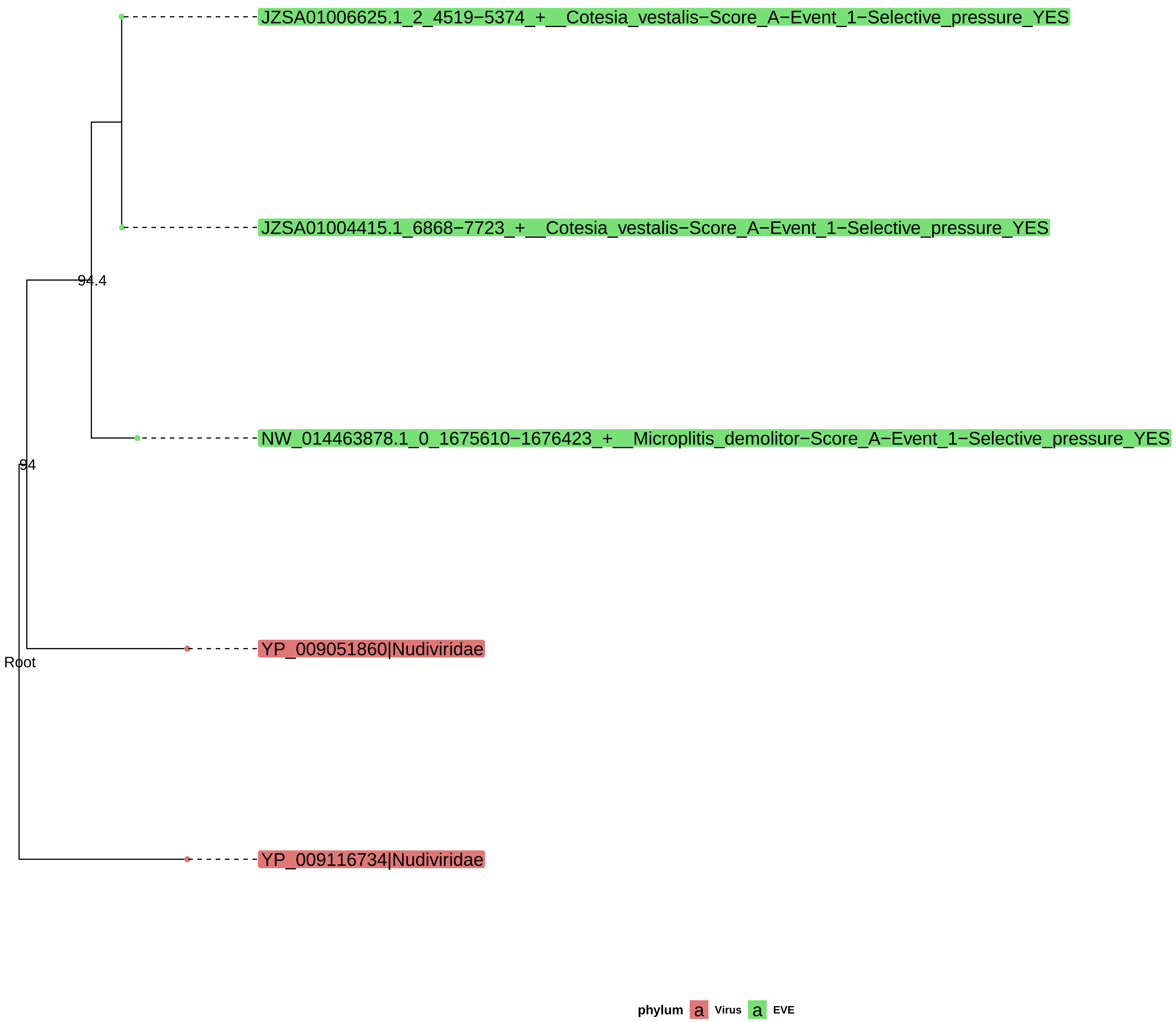

Cluster24469 − families involved : ['Nudiviridae'] − putative protein : ['VLF−1']

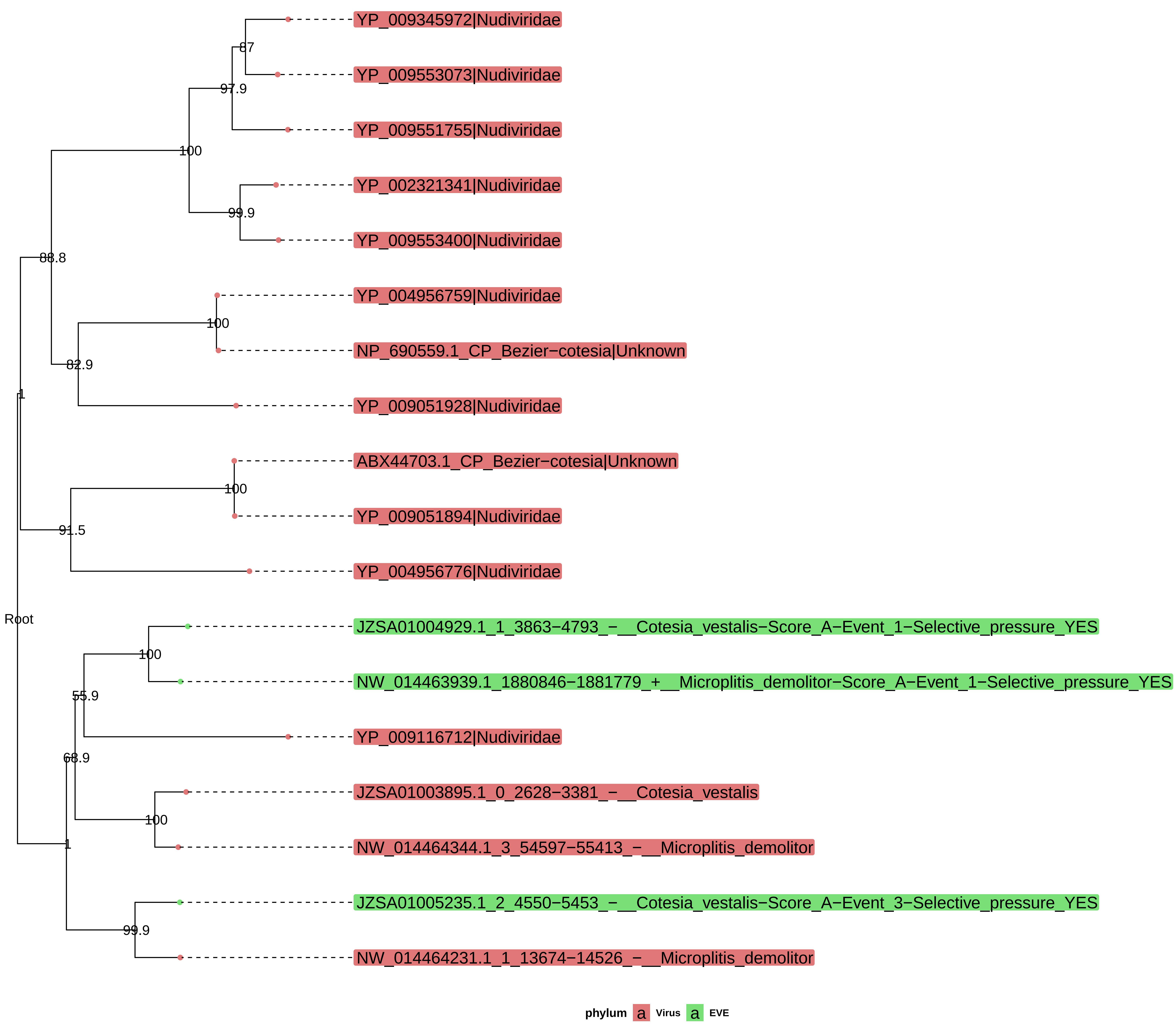

Cluster22358 − families involved : ['Nudiviridae', 'Apis_filamentous−like'] − putative protein : ['Tyr recombinase domain−containing protein']

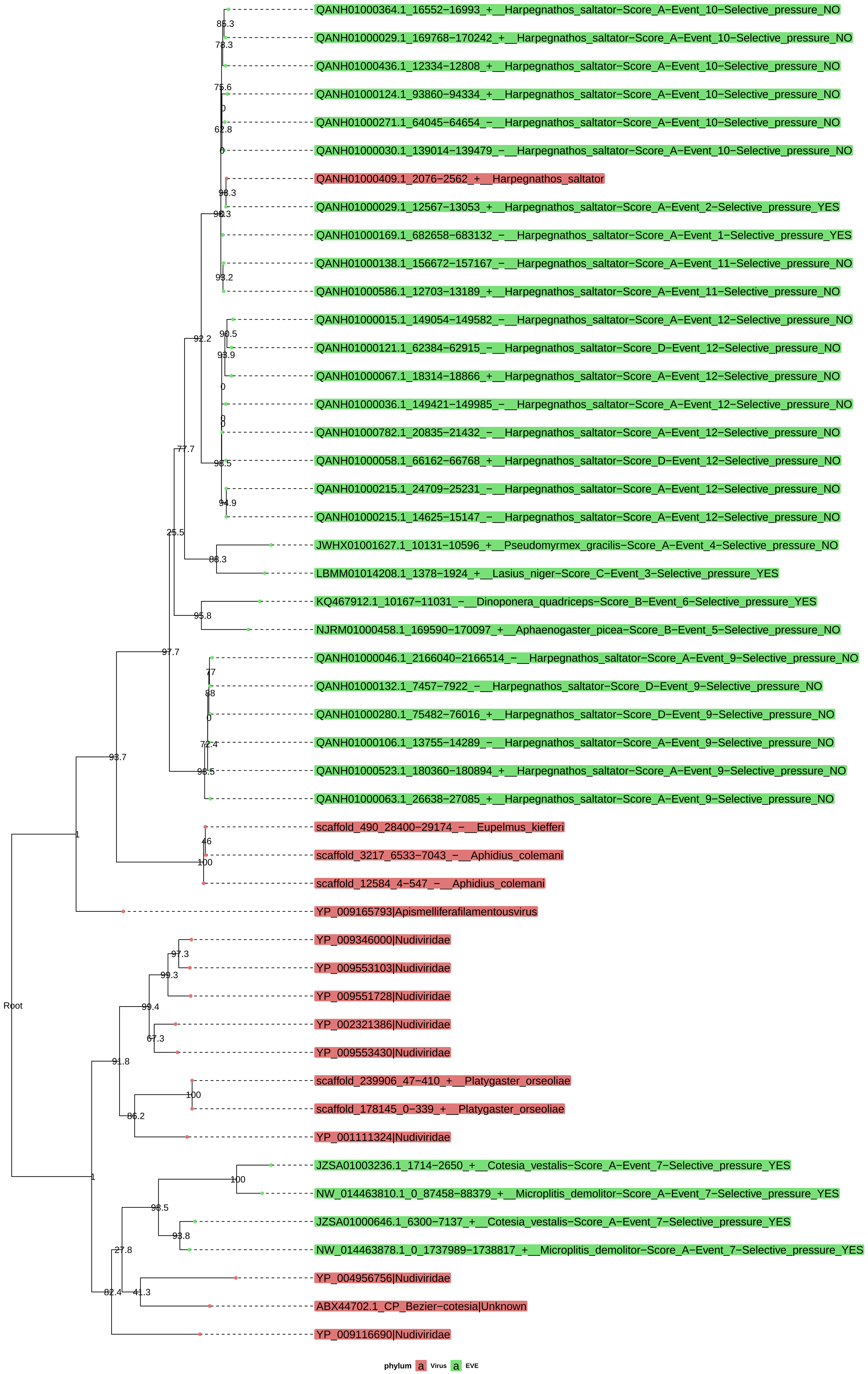

Cluster17433 − families involved : ['Nudiviridae'] − putative protein : ['PIF−5a']

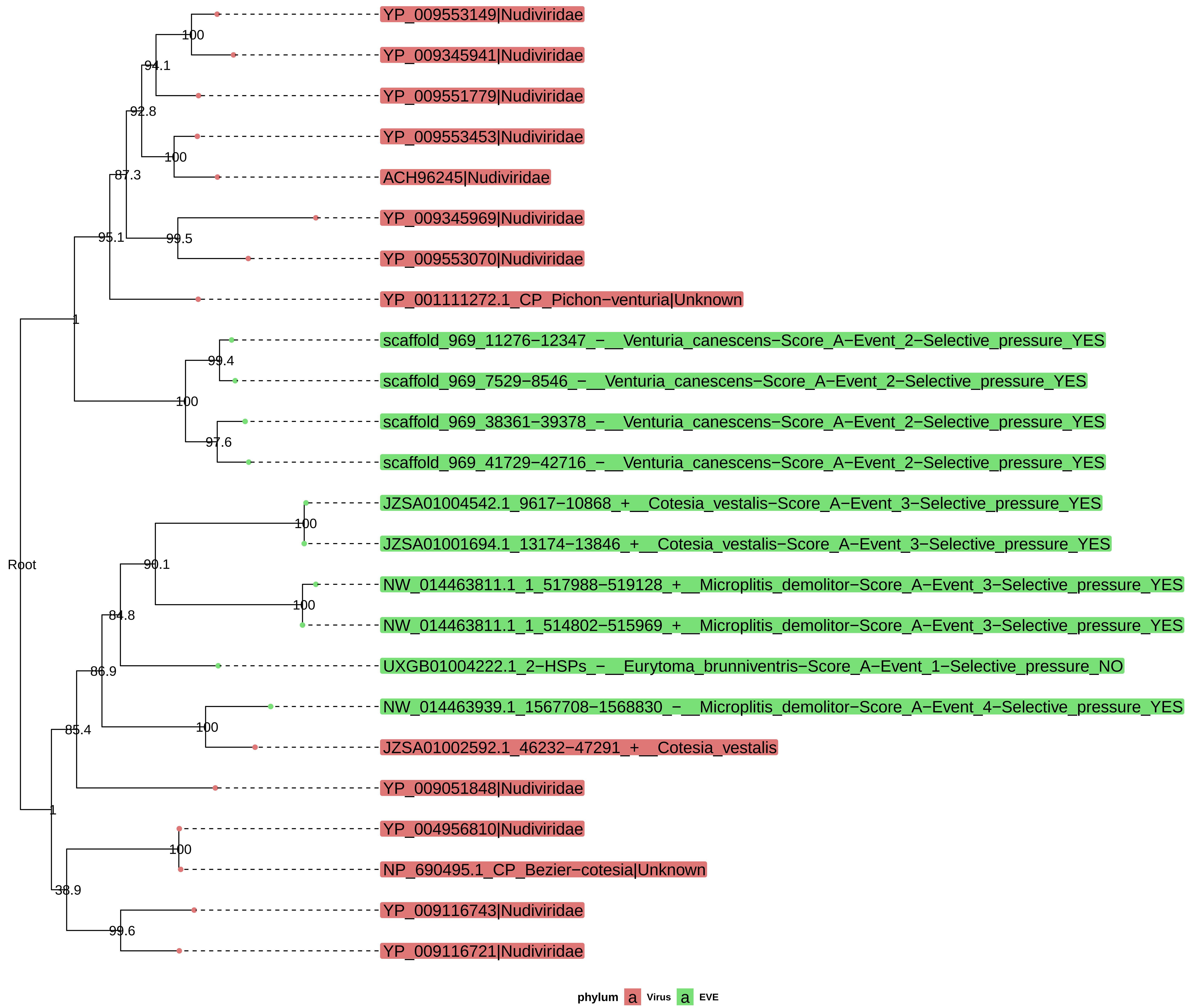

Cluster13904 − families involved : ['Nudiviridae'] − putative protein : ['tRNA−(MS[2]IO[6]A)−hydroxylase (MiaE)']

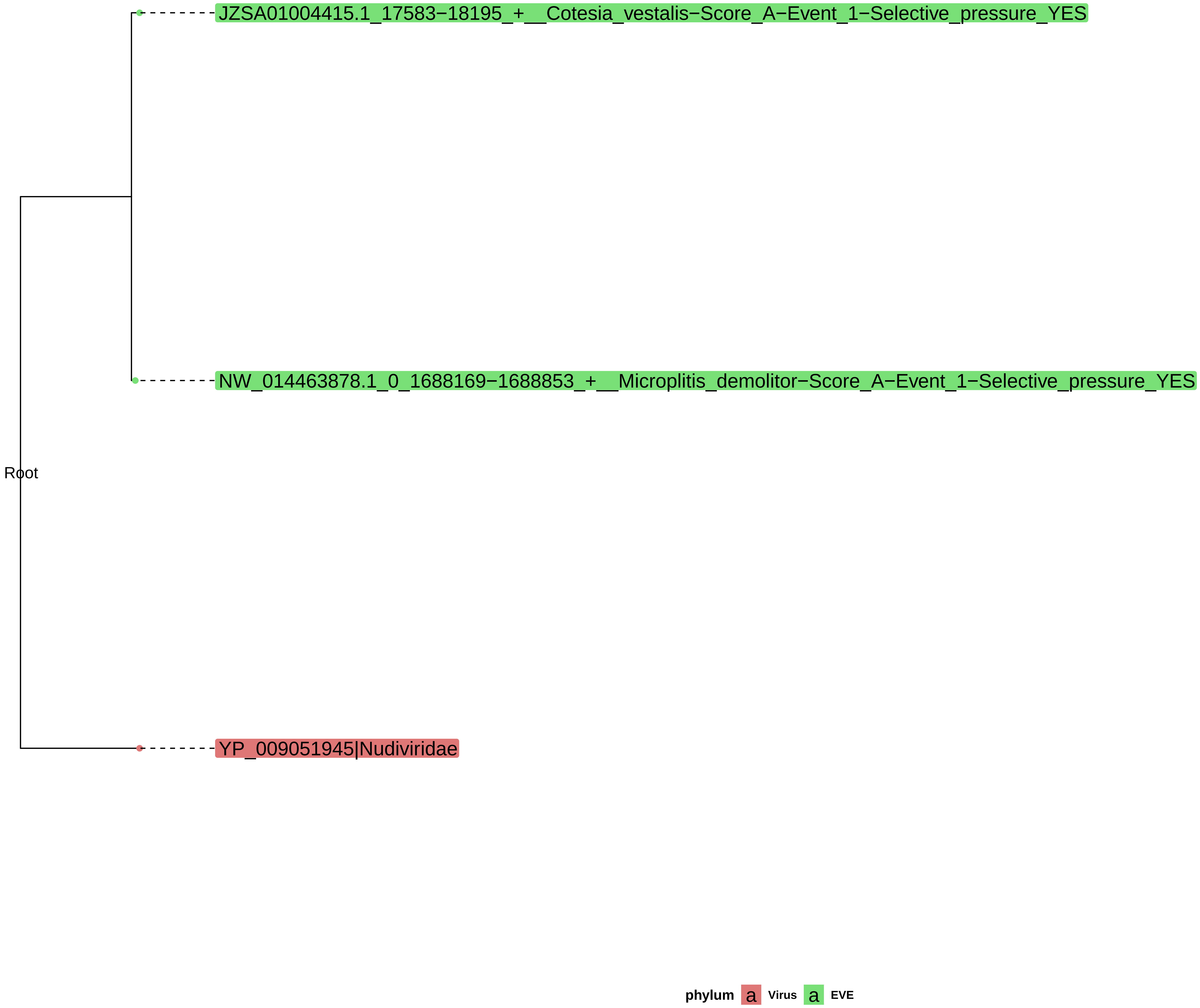

Cluster9642 − families involved : ['Nudiviridae'] − putative protein : ['GrBNV_gp75−like protein']

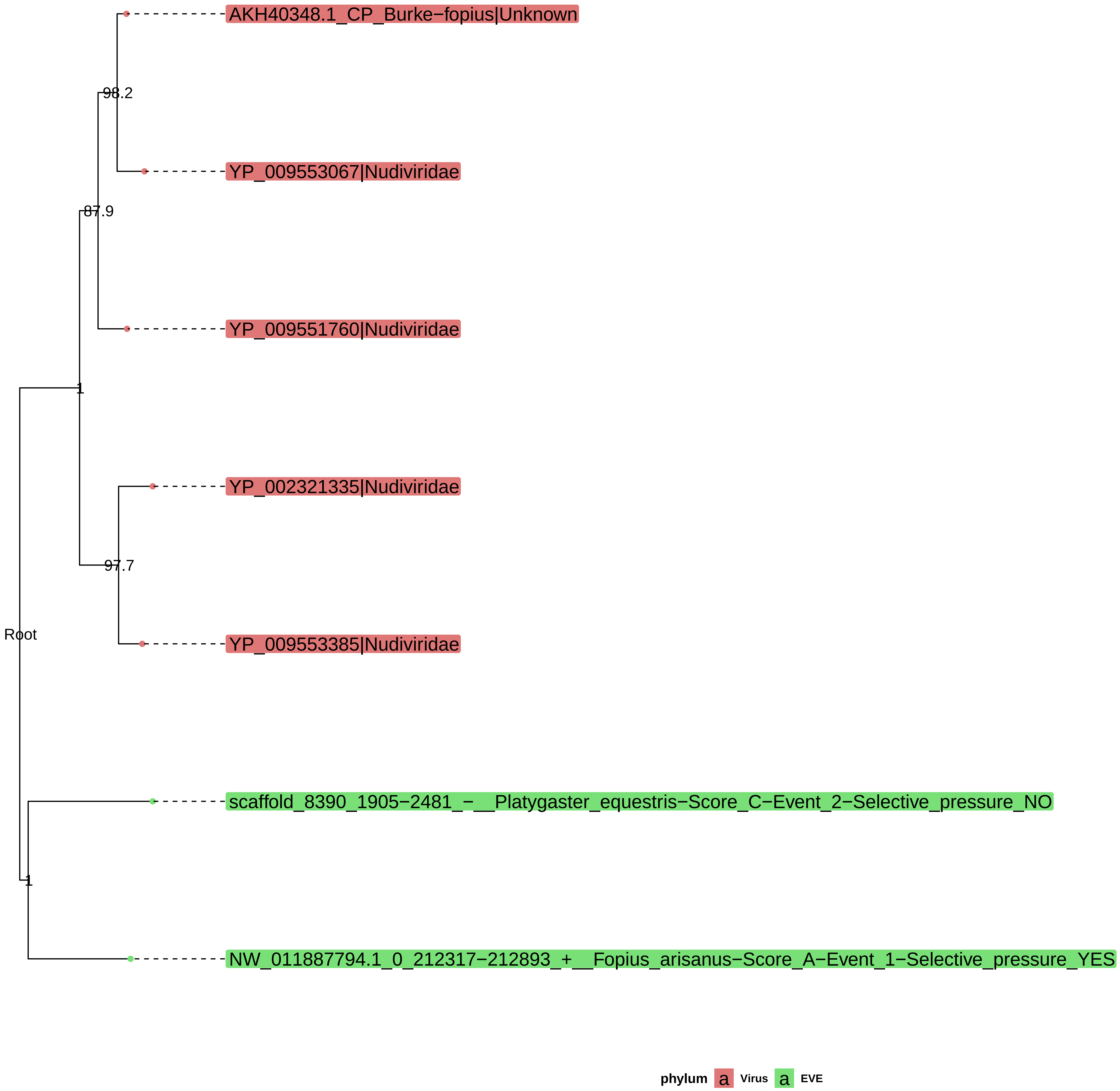

Cluster9535 − families involved : ['Nudiviridae'] − putative protein : ['Late expression factor 4']

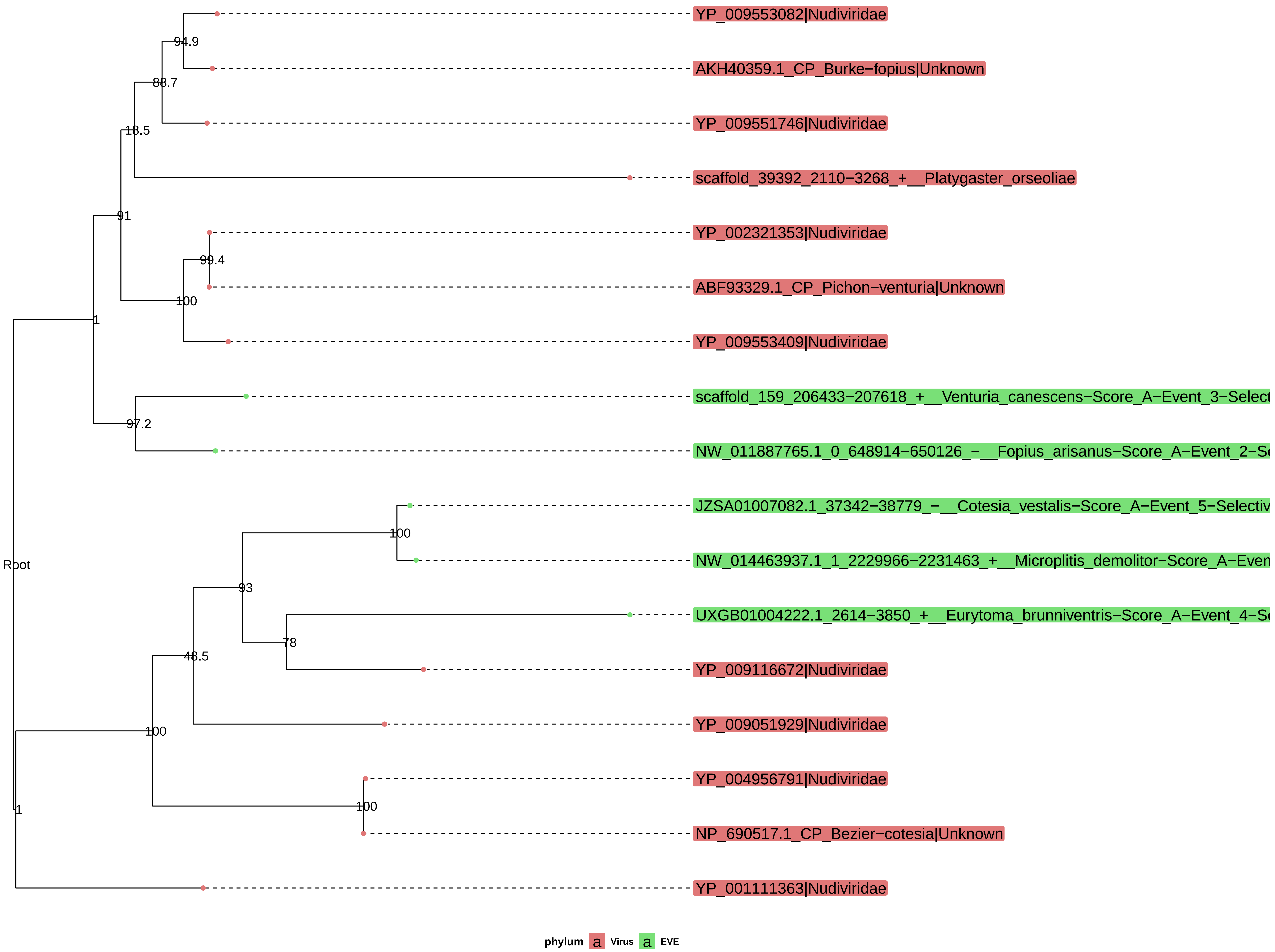

Cluster8604 − families involved : ['Nudiviridae'] − putative protein : ['GrBNV_gp60−like protein']

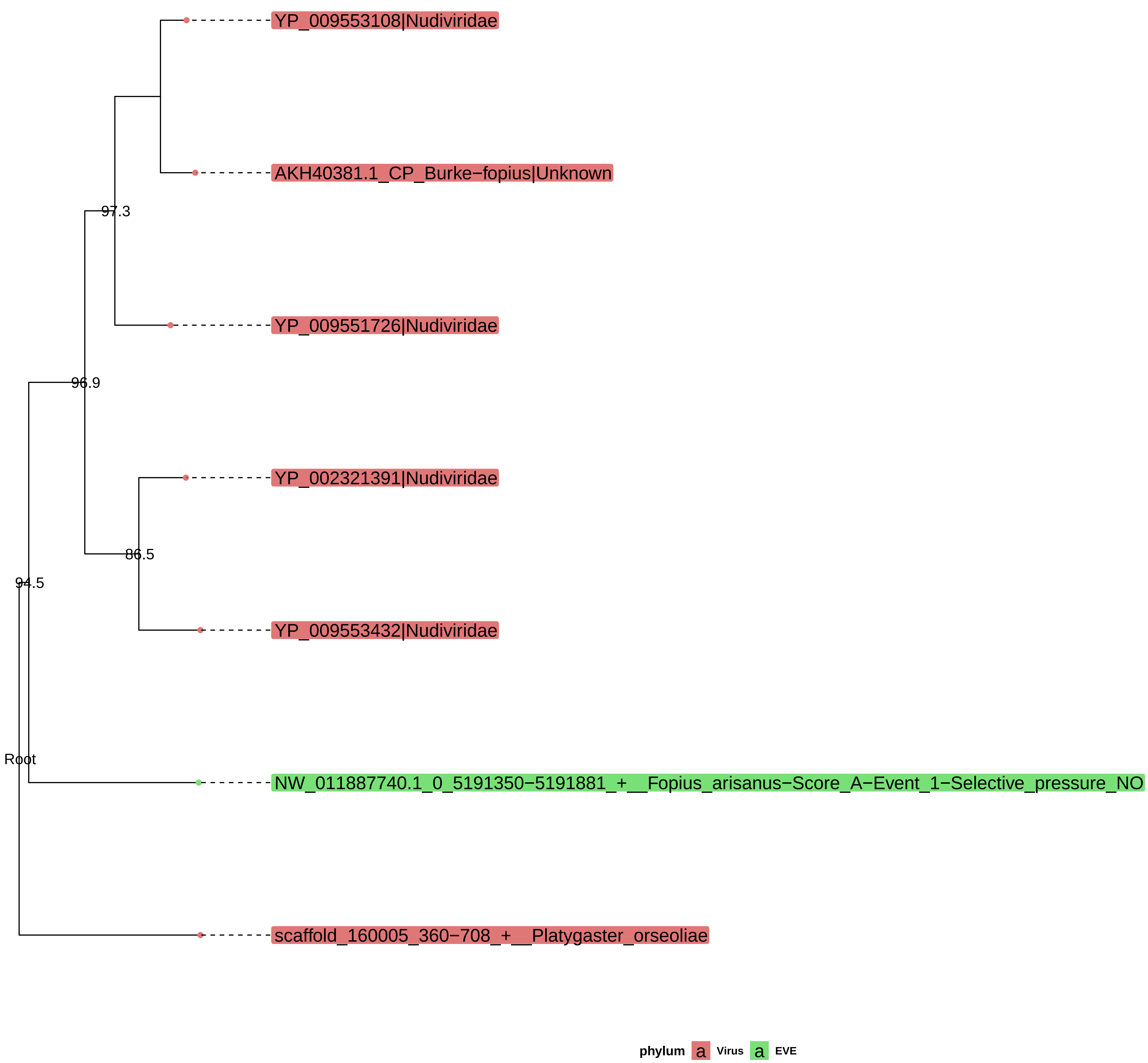

Cluster8496 − families involved : ['Nudiviridae'] − putative protein : ['Sulfhydryl oxidase (EC 1.8.3.2)']

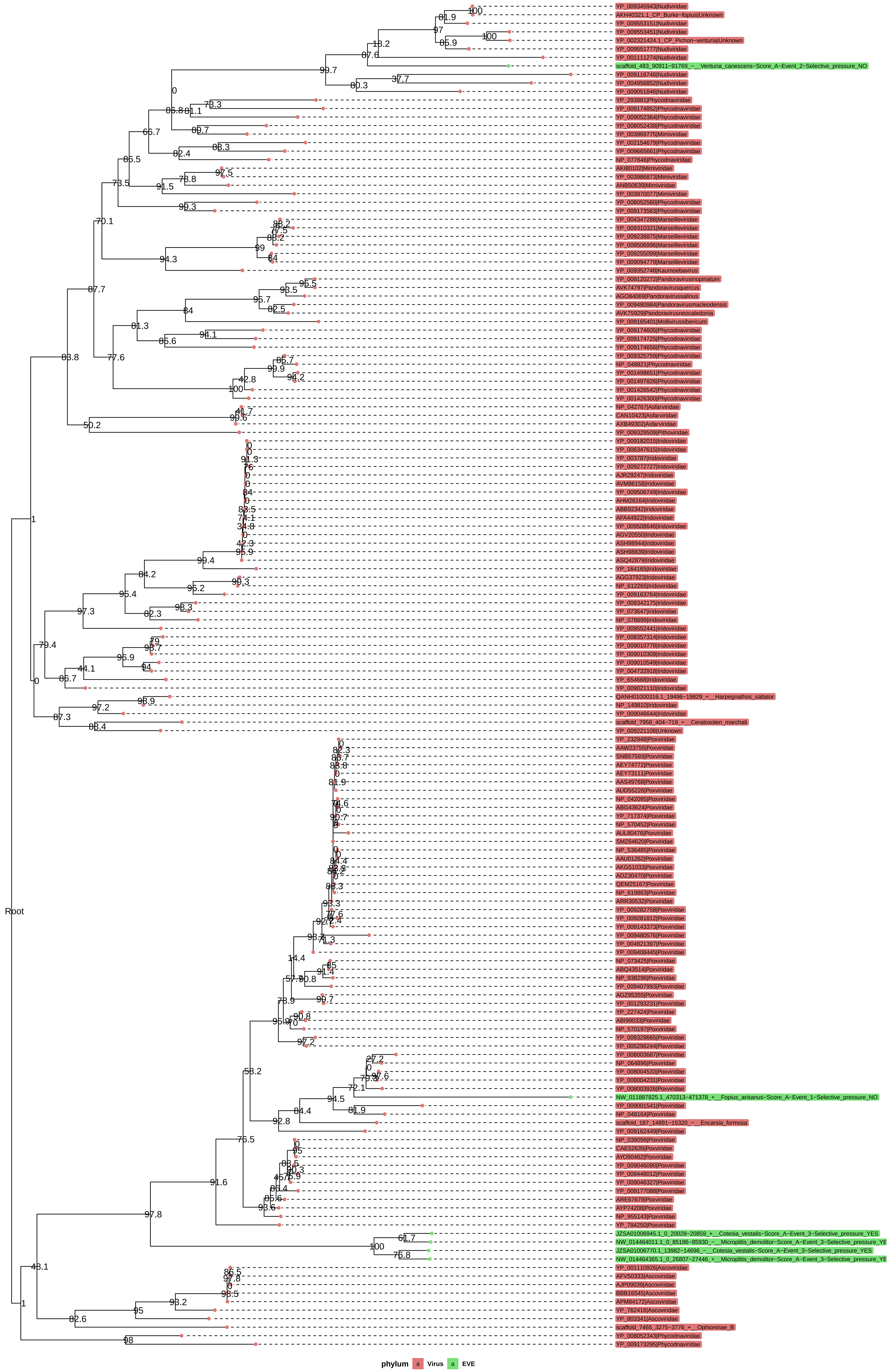

Cluster5096 − families involved : ['Nudiviridae', 'Apis_filamentous−like'] − putative protein : ['Baculovirus 19 kDa protein conserved region']

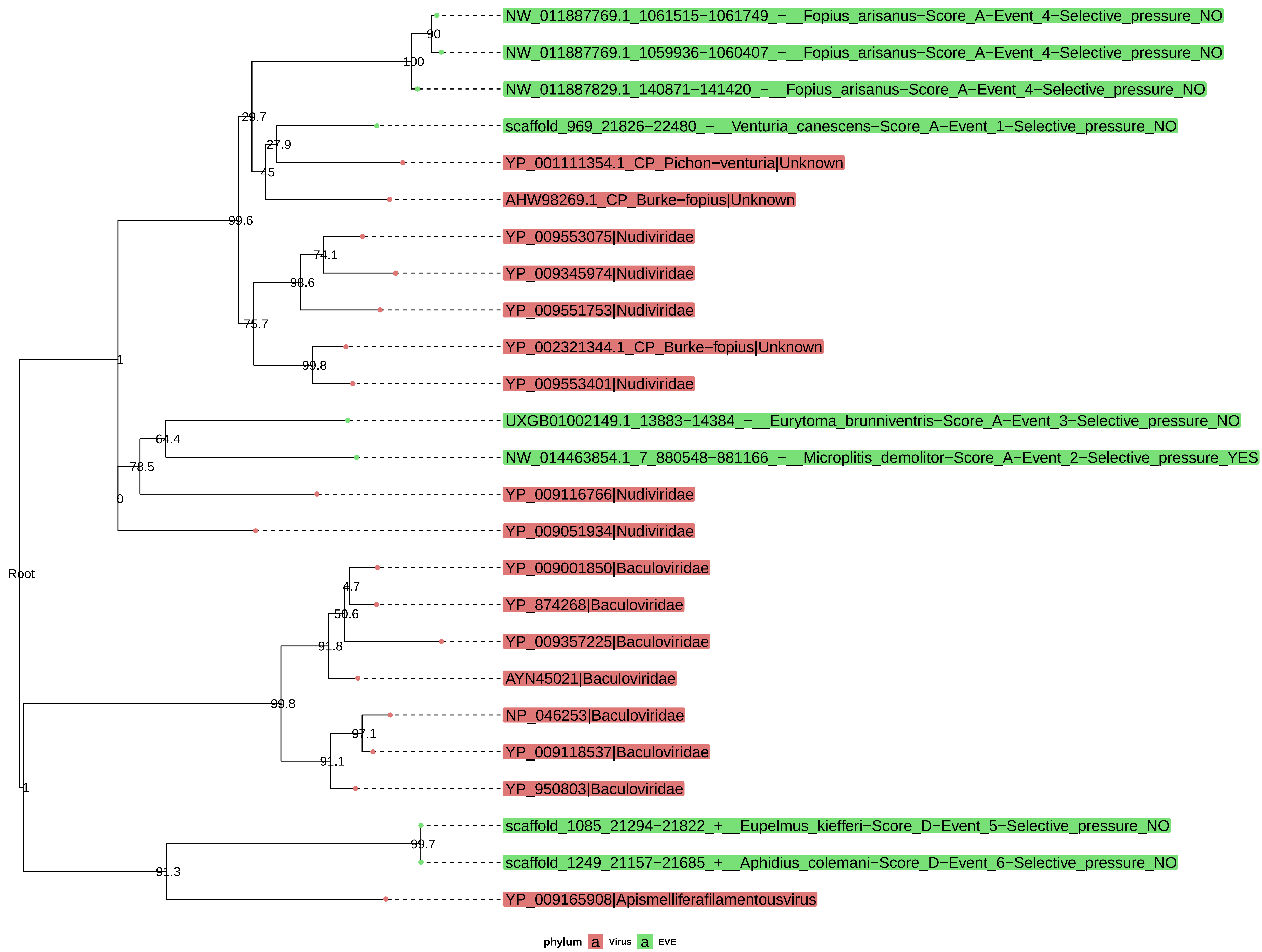

Cluster469 − families involved : ['Nudiviridae'] − putative protein : ['Ac146−like protein']

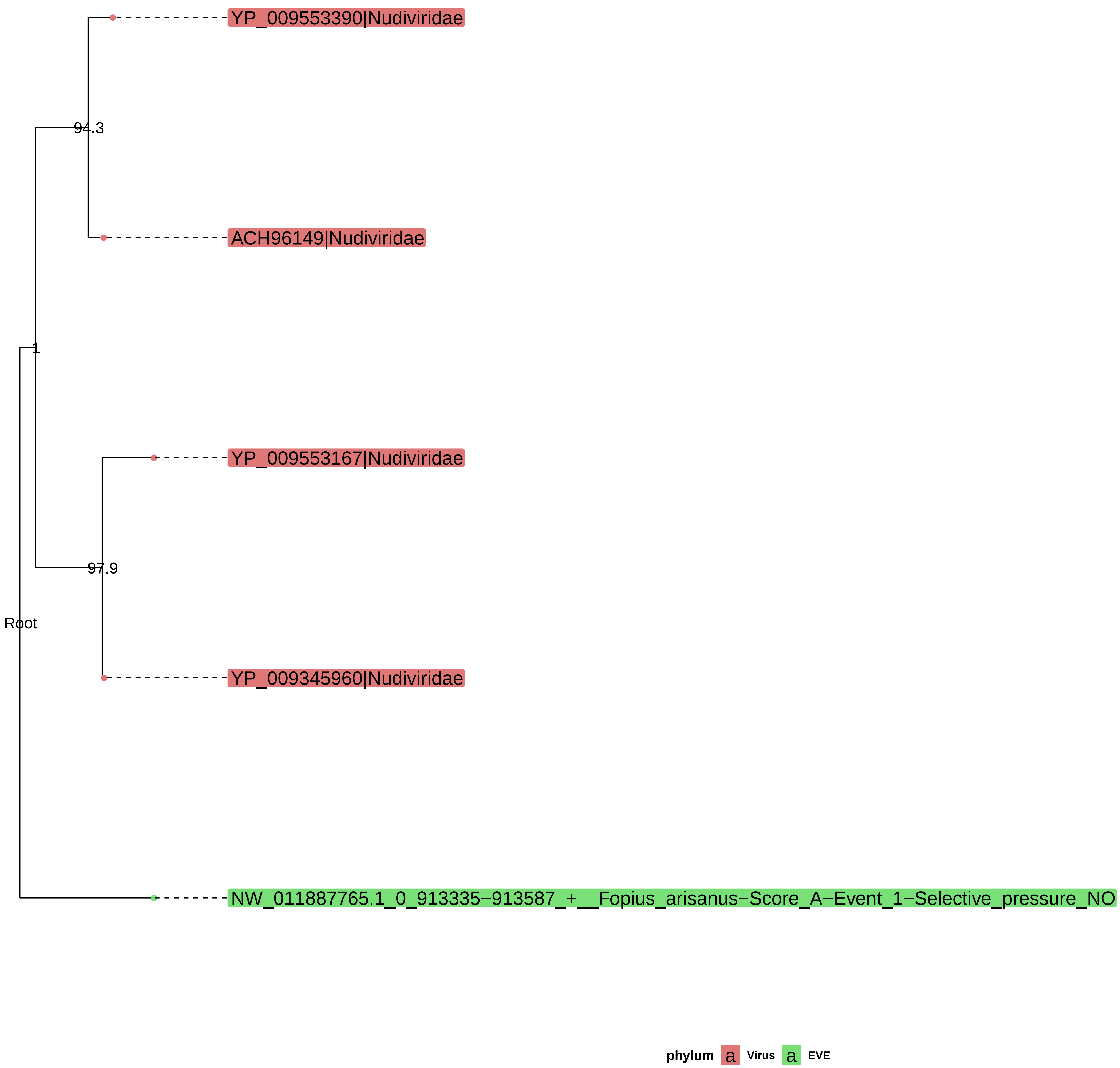

Cluster4454 − families involved : ['Nudiviridae'] − putative protein : ['GrBNV_gp36−like protein']

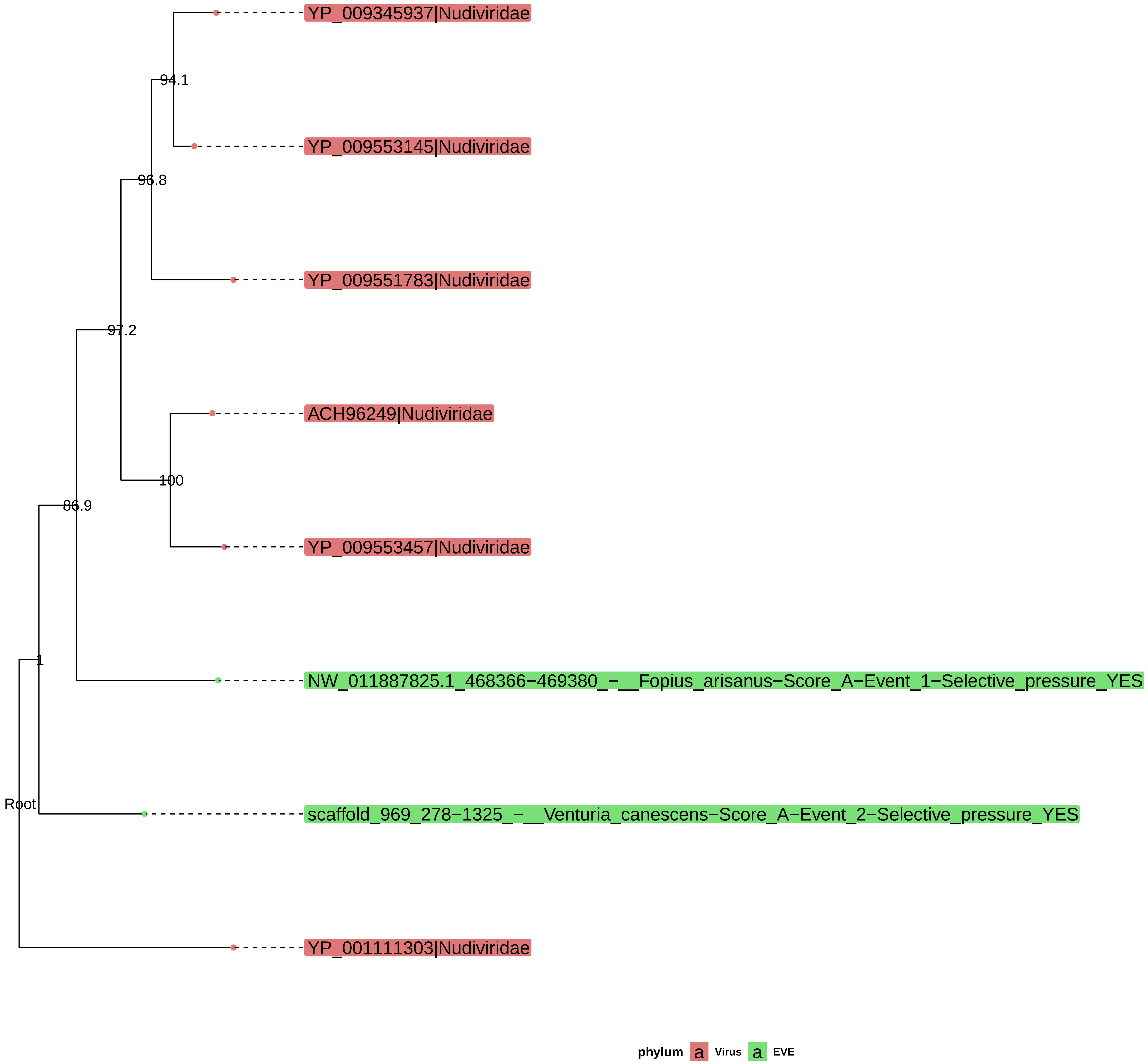

Cluster3912 − families involved : ['Nudiviridae'] − putative protein : ['ACH96220.1 GrBNV gp28−like protein']

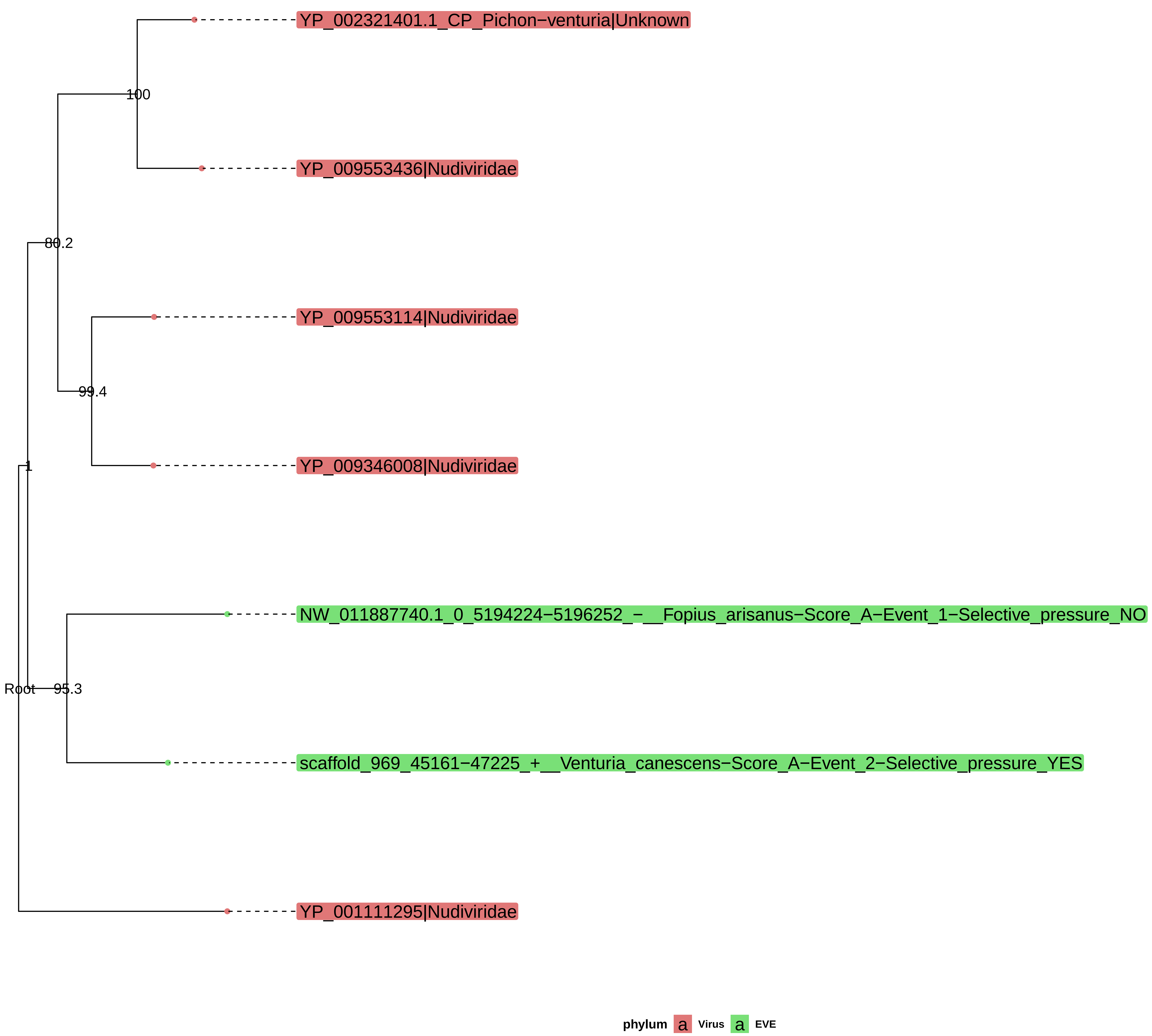

Cluster335 − families involved : ['Nudiviridae'] − putative protein : ['Transmembrane domain of protein tyrosine phosphatase, receptor type J']

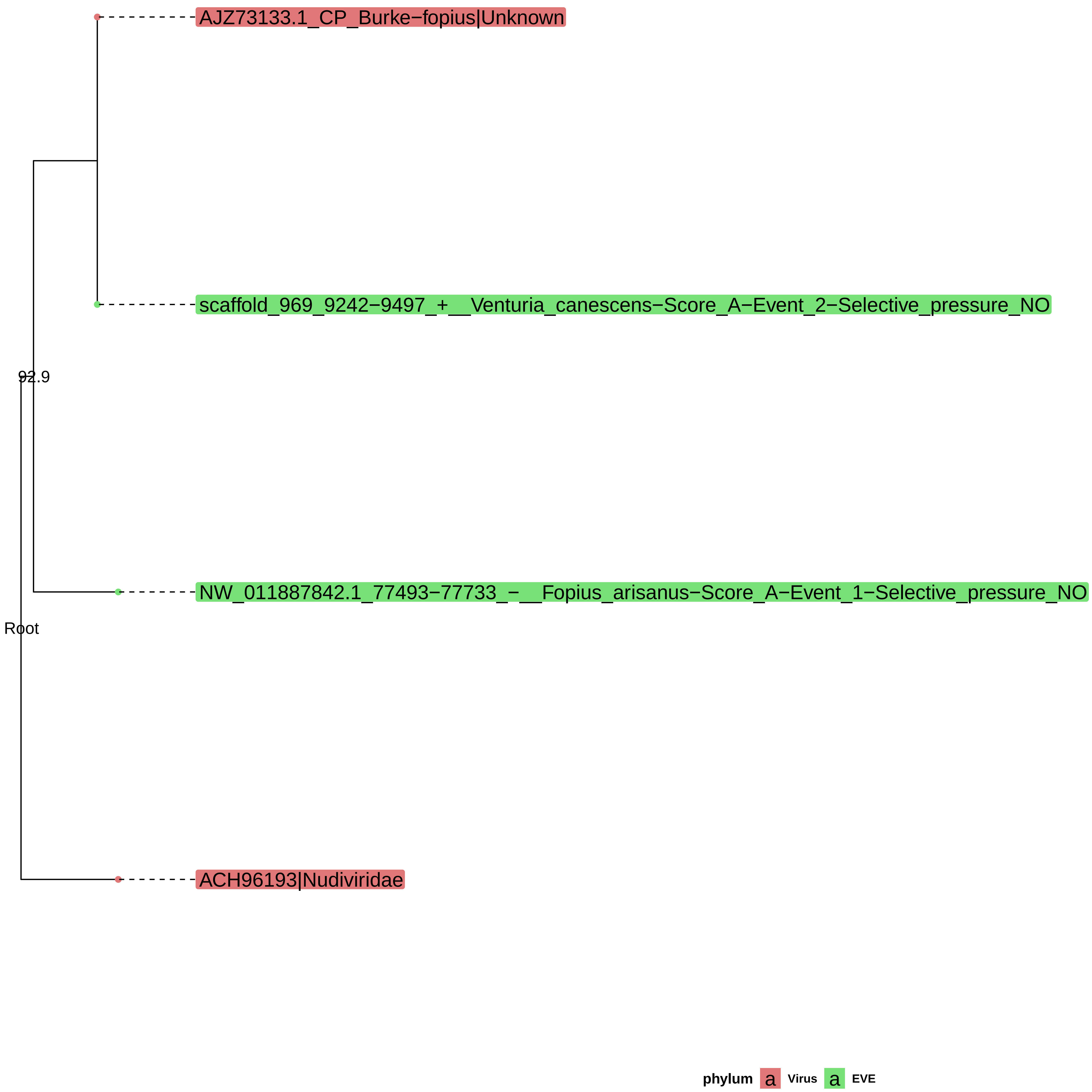

Cluster3232 − families involved : ['Nudiviridae'] − putative protein : ['ACH96148.1 GrBNV gp67−like protein']

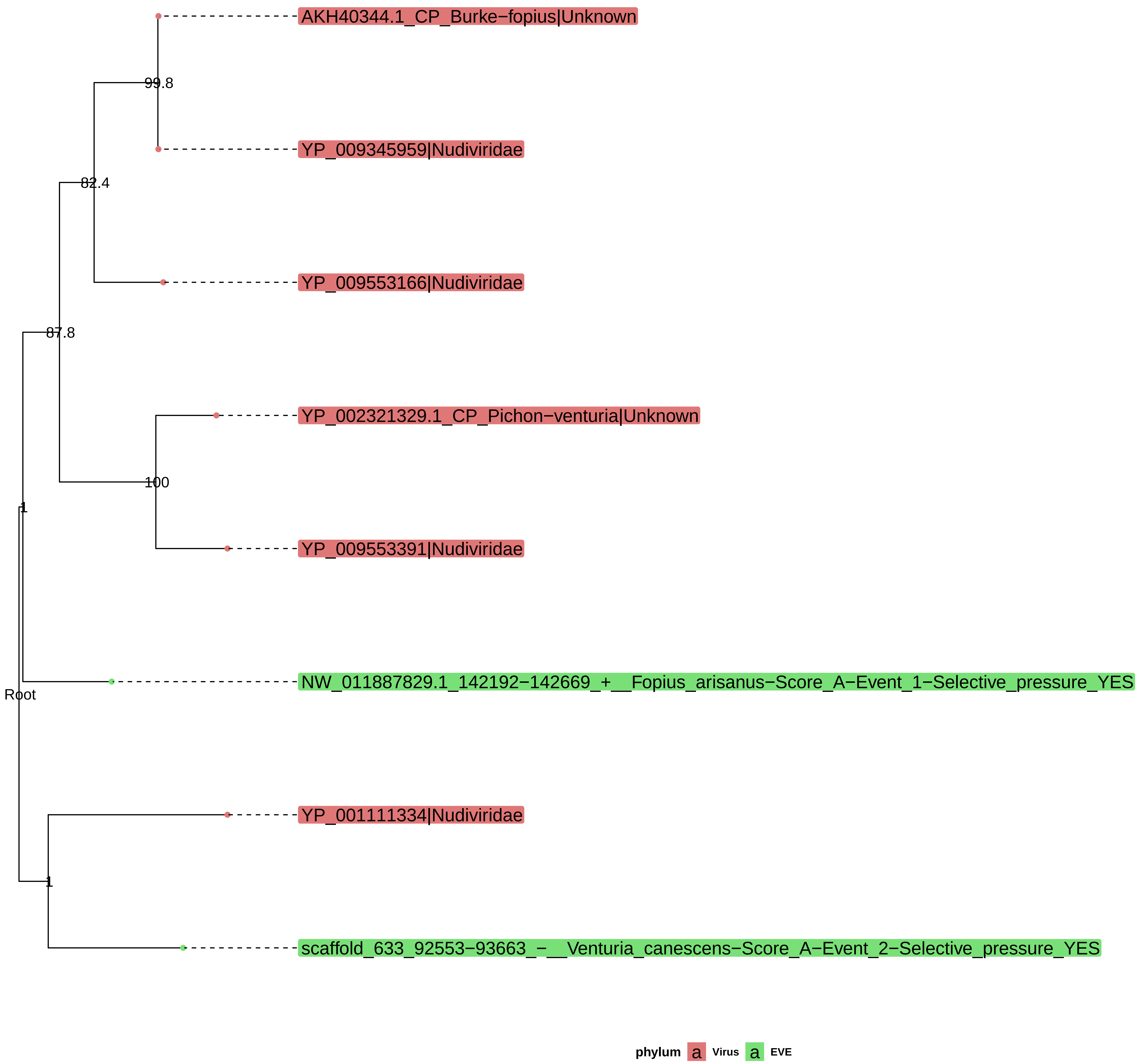

Cluster312 − families involved : ['Nudiviridae', 'Baculoviridae'] − putative protein : ['Vp91']

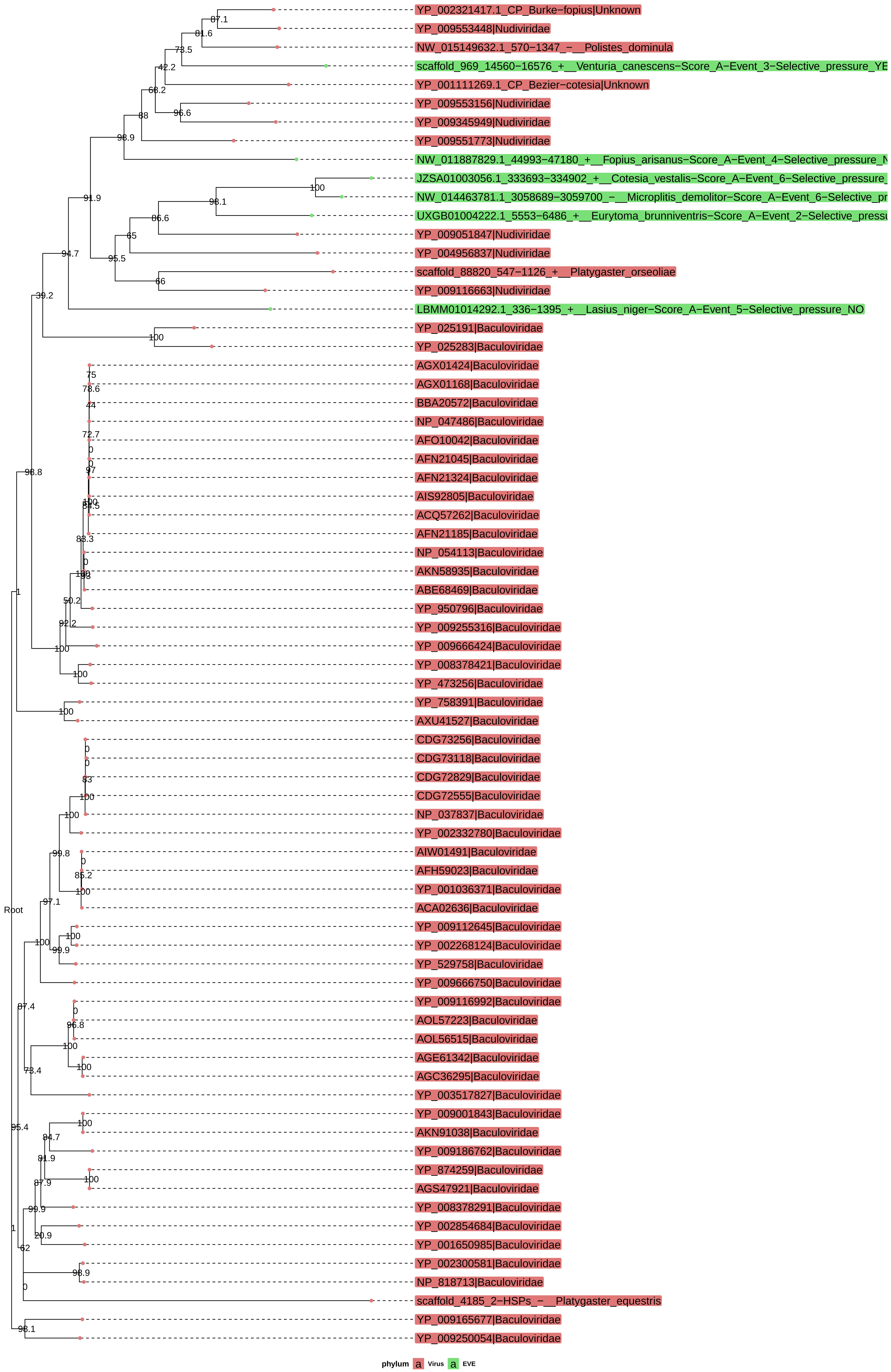

Cluster3072 − families involved : ['Nudiviridae'] − putative protein : ['p47']

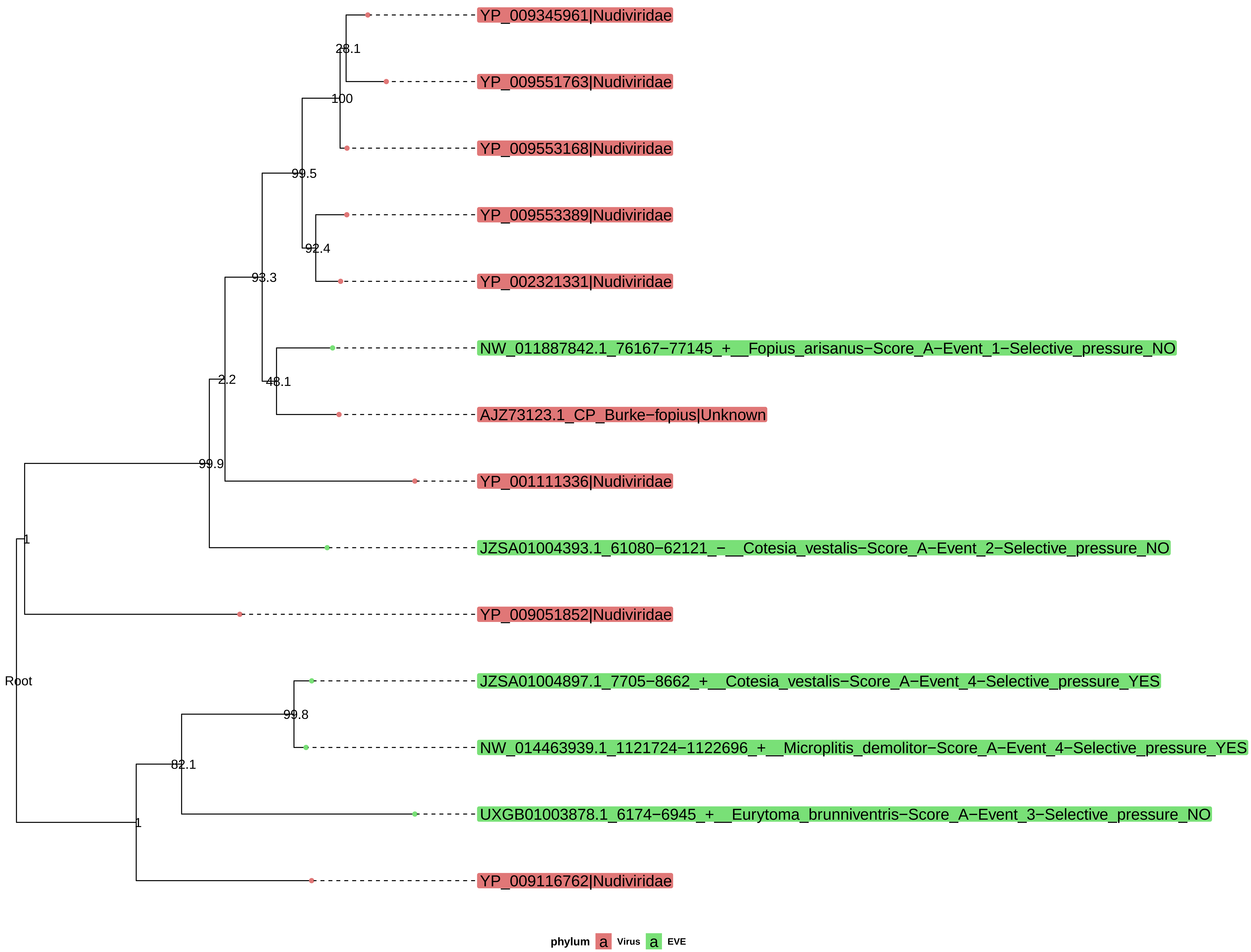

Cluster27187 − families involved : ['Nudiviridae'] − putative protein : ['GrBNV_gp84−like protein']

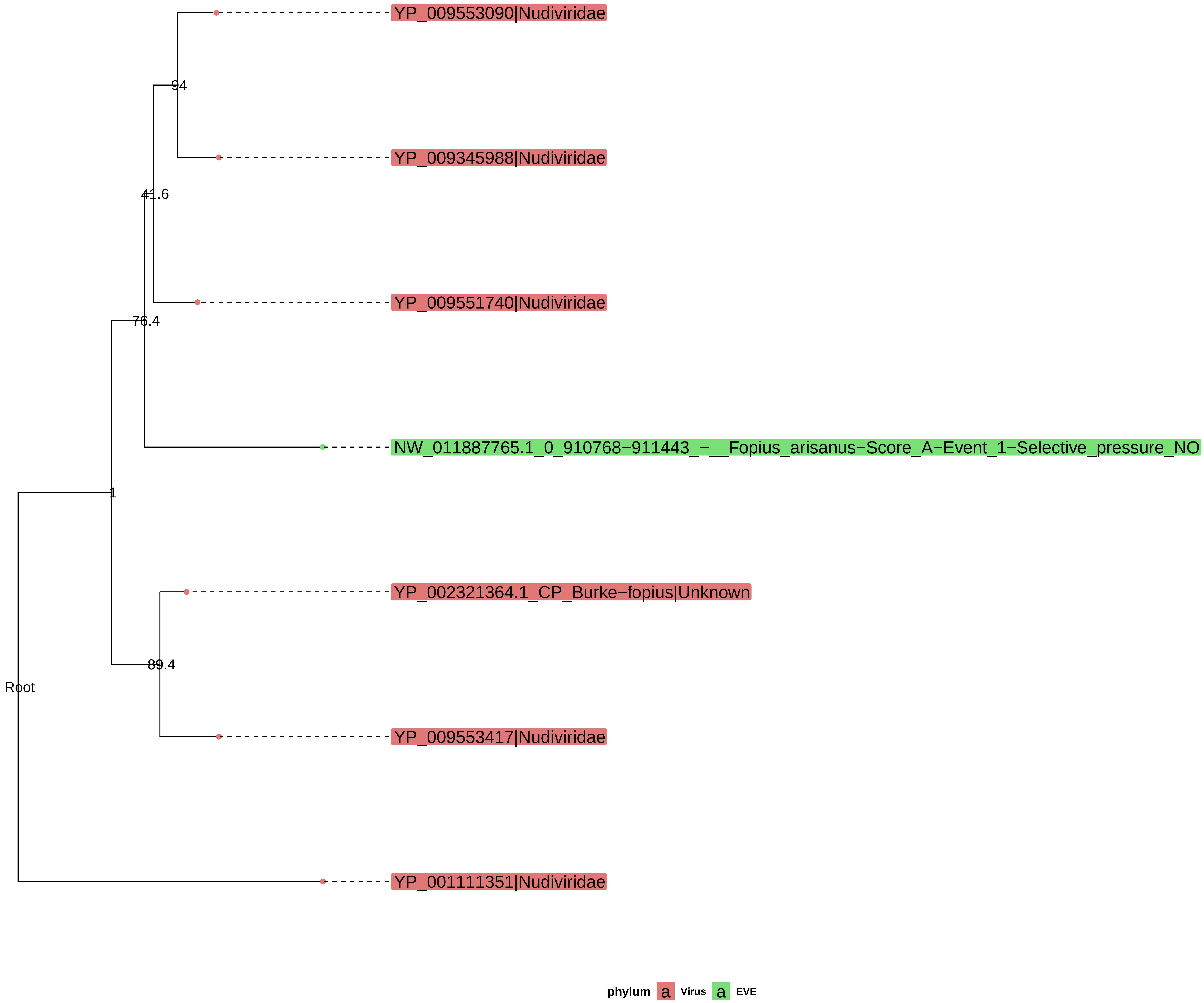

Cluster26944 − families involved : ['Nudiviridae'] − putative protein : [nan]

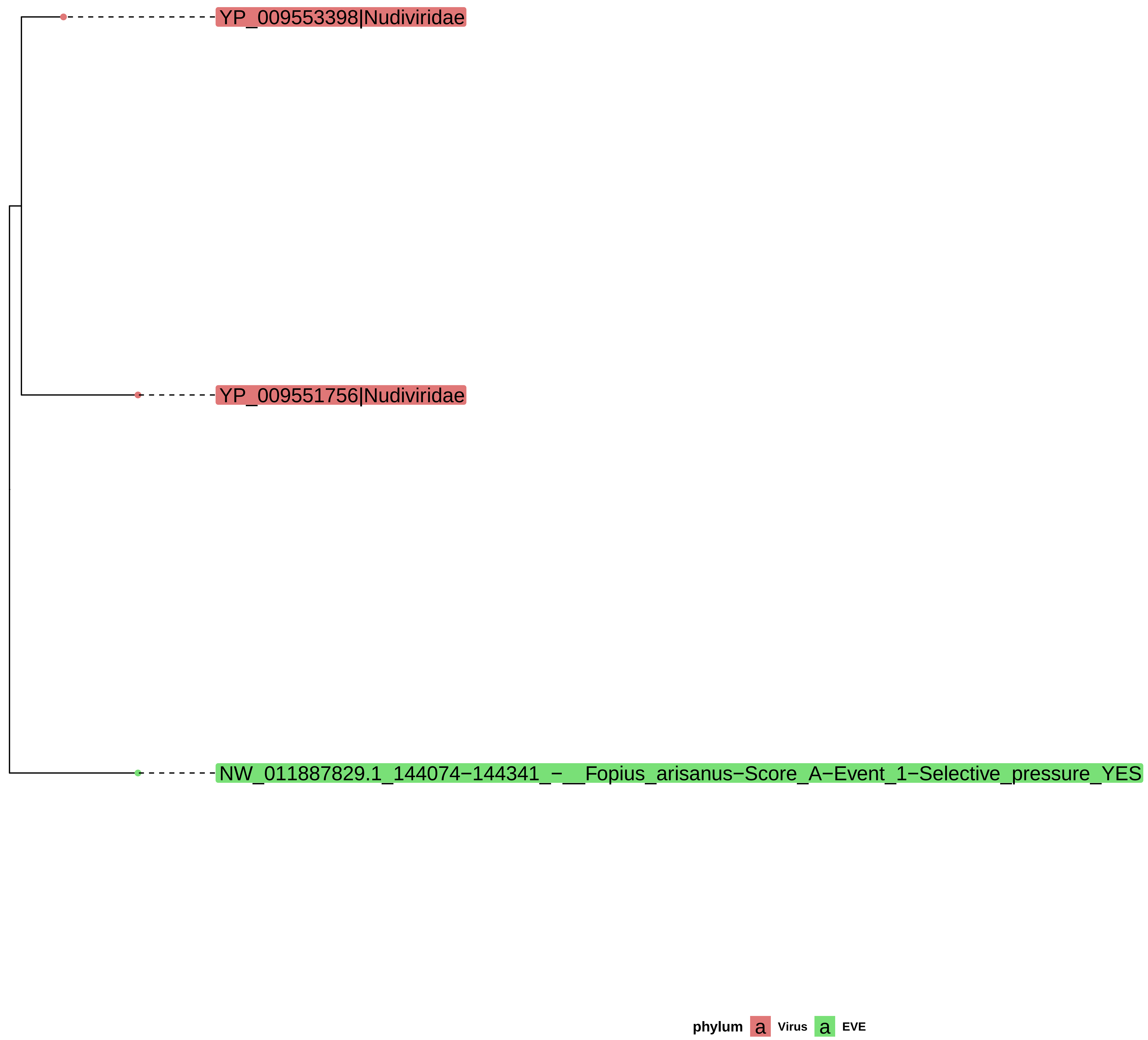

Cluster26 − families involved : ['Nudiviridae'] − putative protein : ['Ac81−like protein']

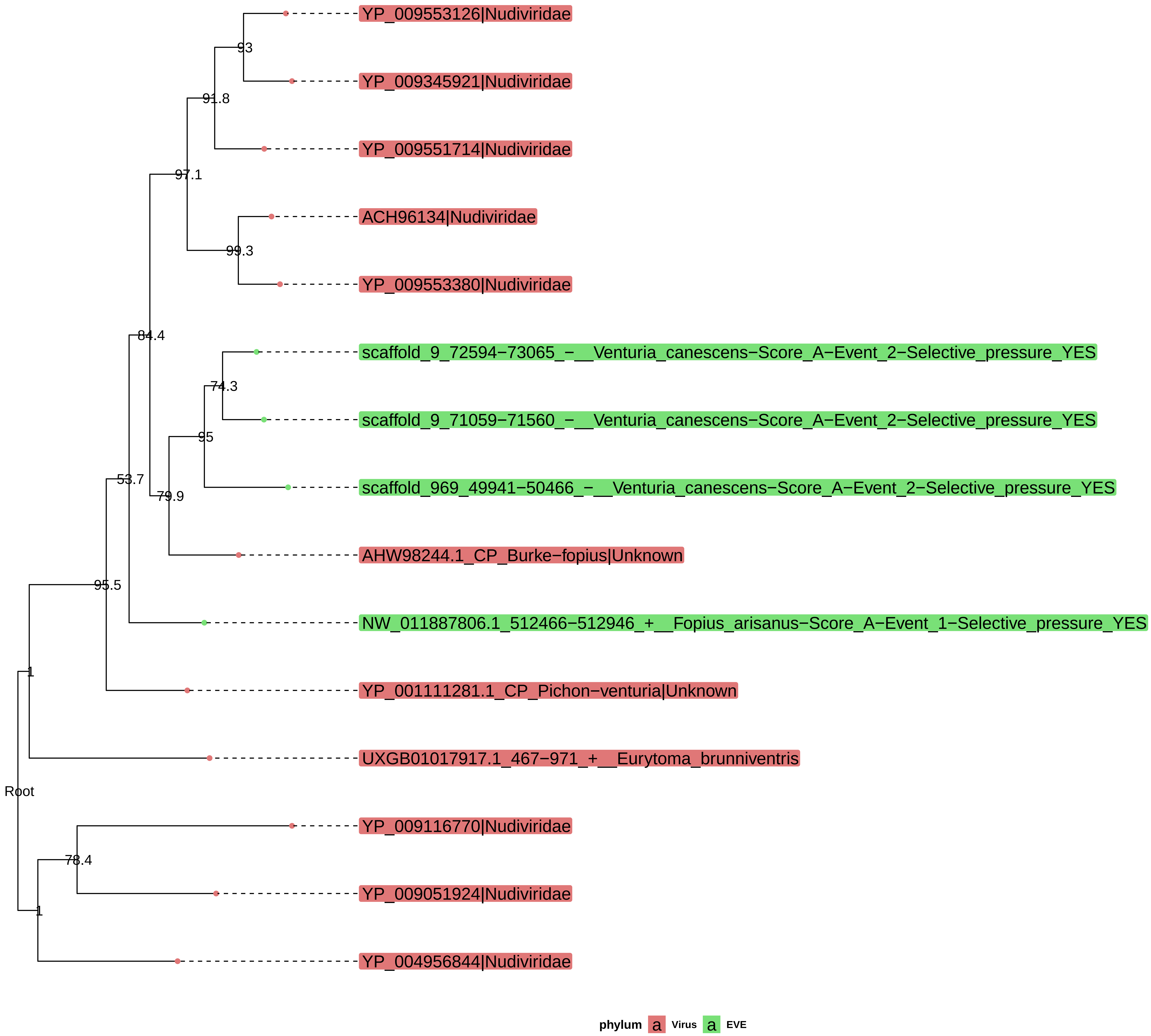

Cluster25942 − families involved : ['Nudiviridae'] − putative protein : ['ACH96225.1 OrNV gp095−like protein']

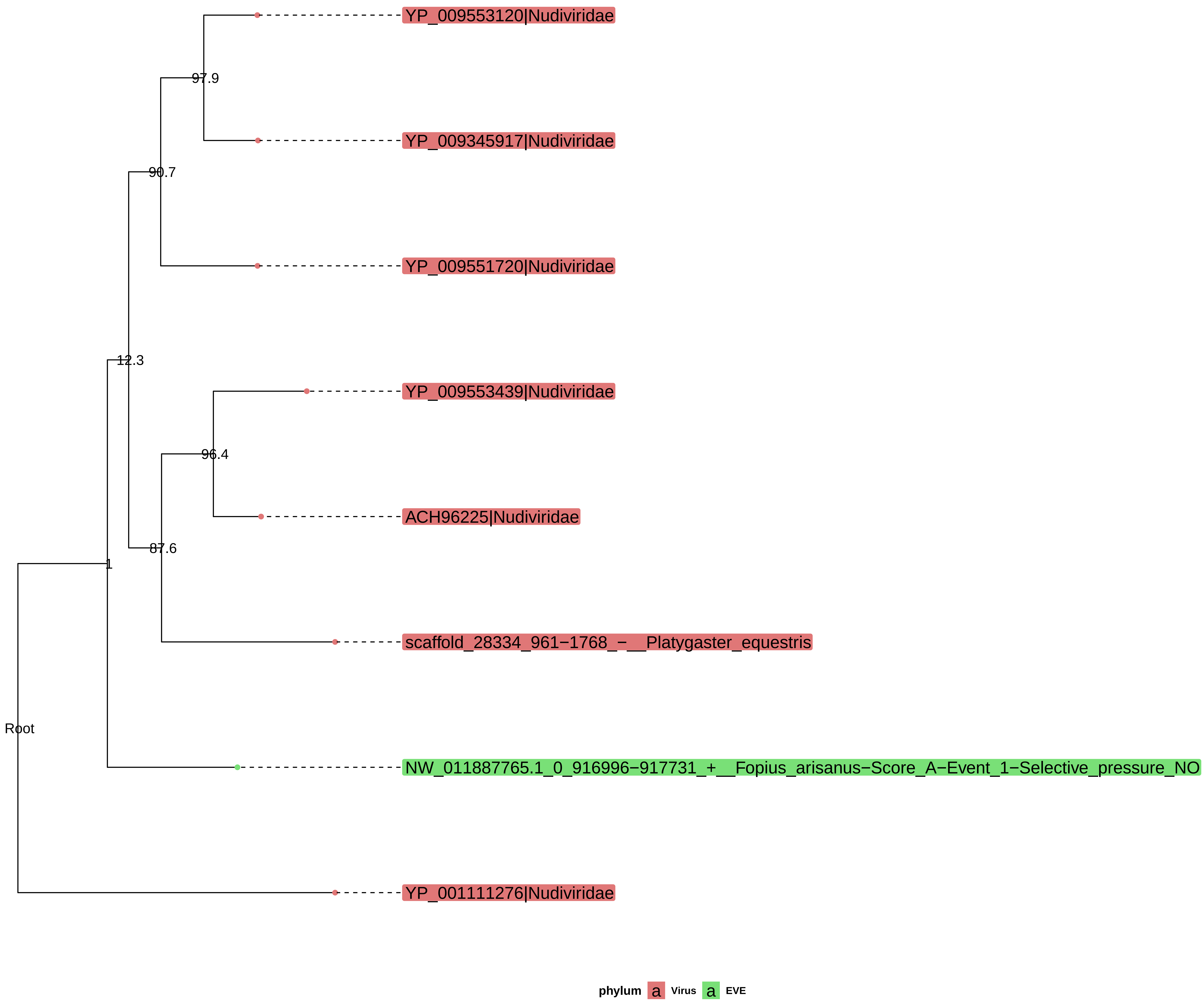

Cluster23903 − families involved : ['Nudiviridae'] − putative protein : ['Uncharacterized protein']

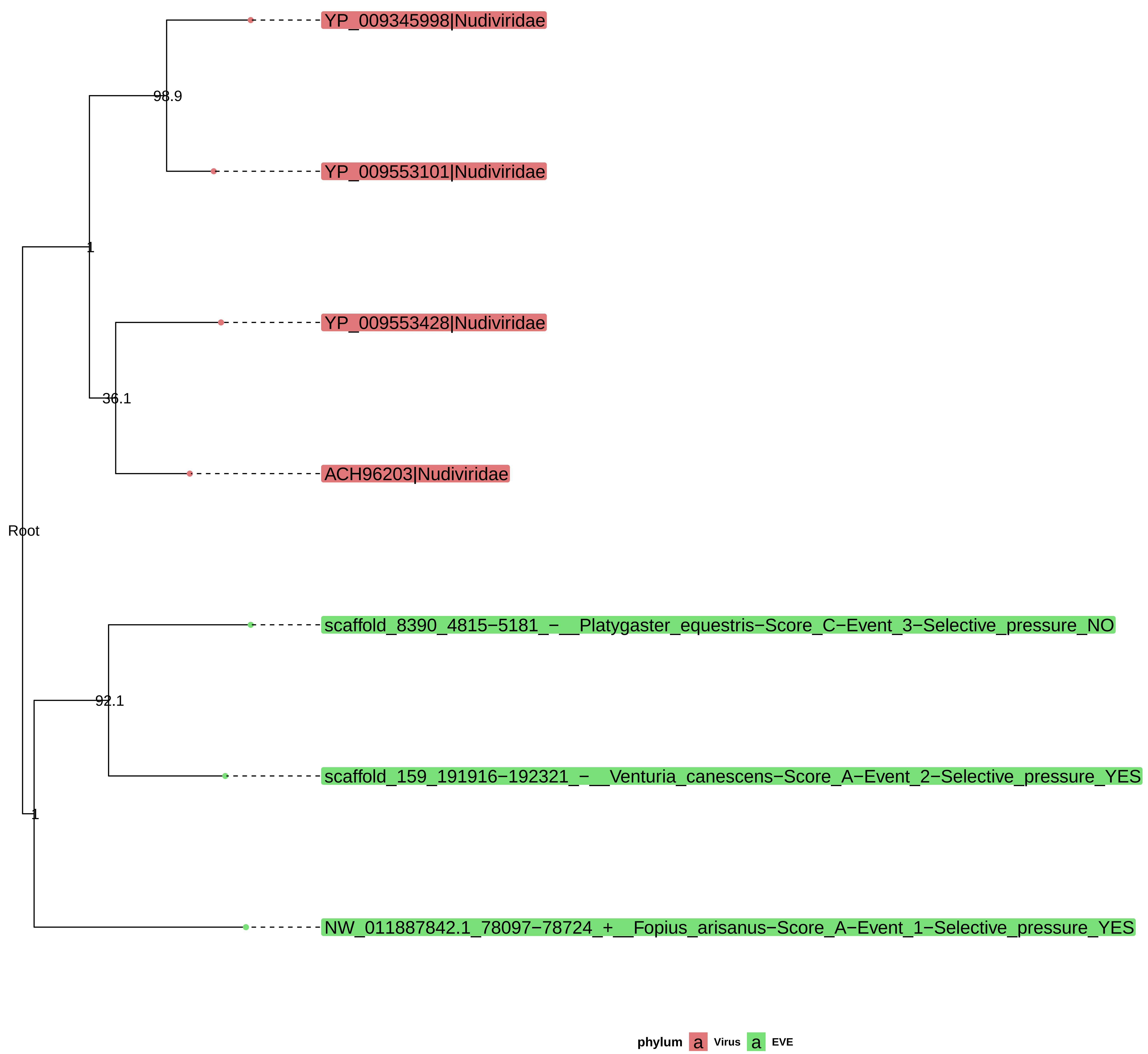

Cluster23691 − families involved : ['Nudiviridae'] − putative protein : ['ACH96133.1 GrBNV gp13−like protein']

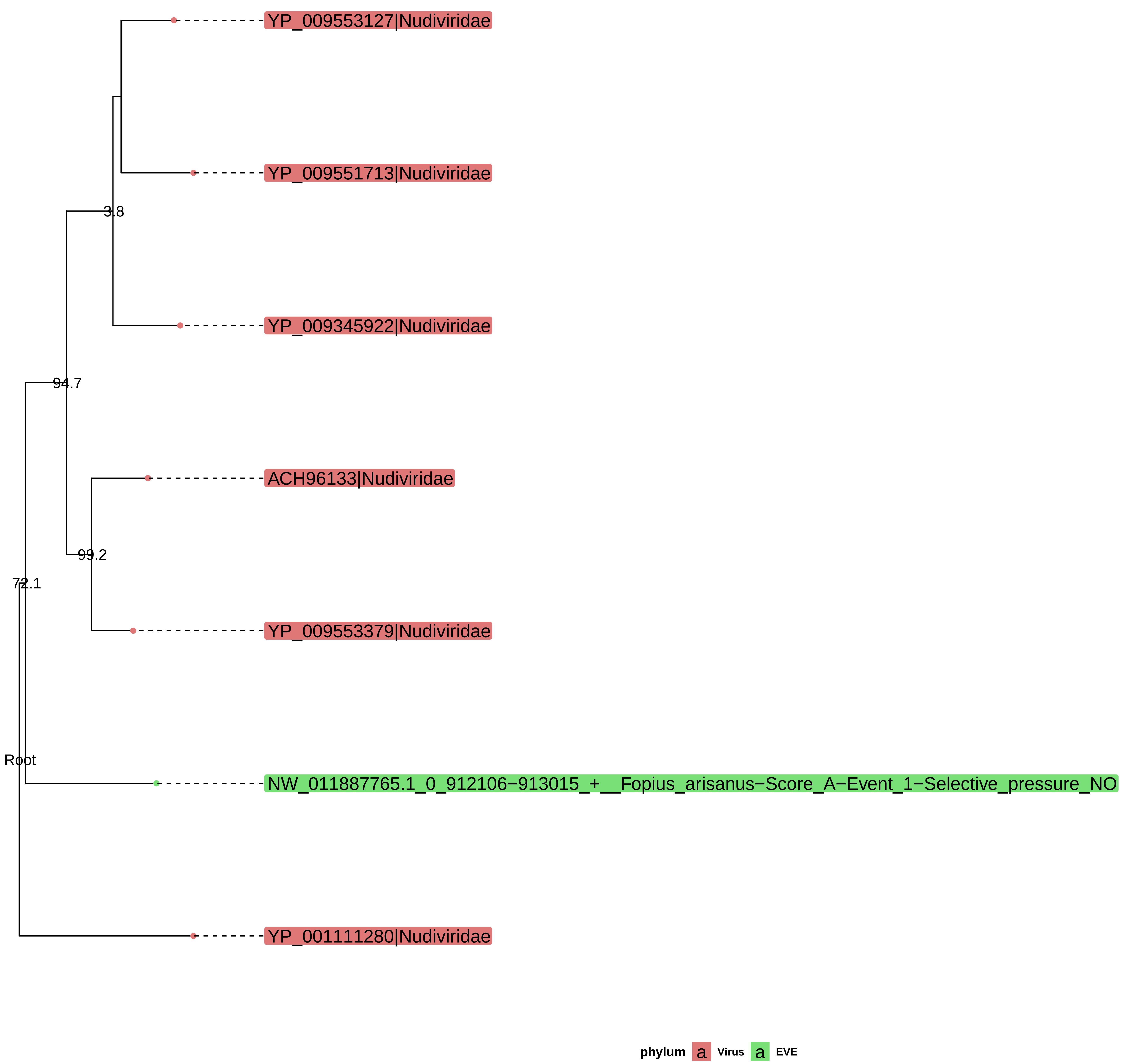

Cluster23368 − families involved : ['Nudiviridae'] − putative protein : ['GrBNV_gp39−like protein']

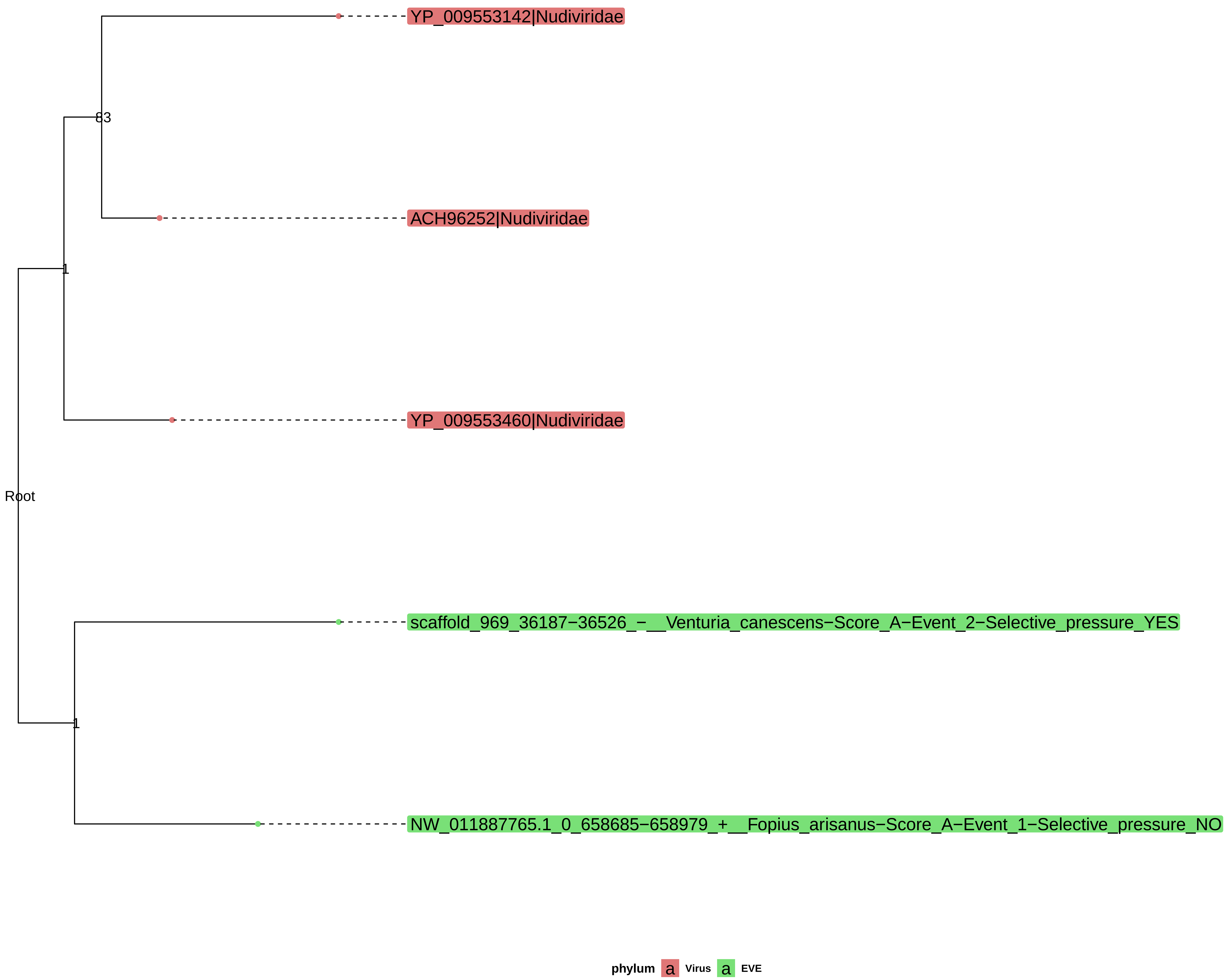

Cluster2197 − families involved : ['Nudiviridae'] − putative protein : ['ACH96250.1 GrBNV gp37−like protein']

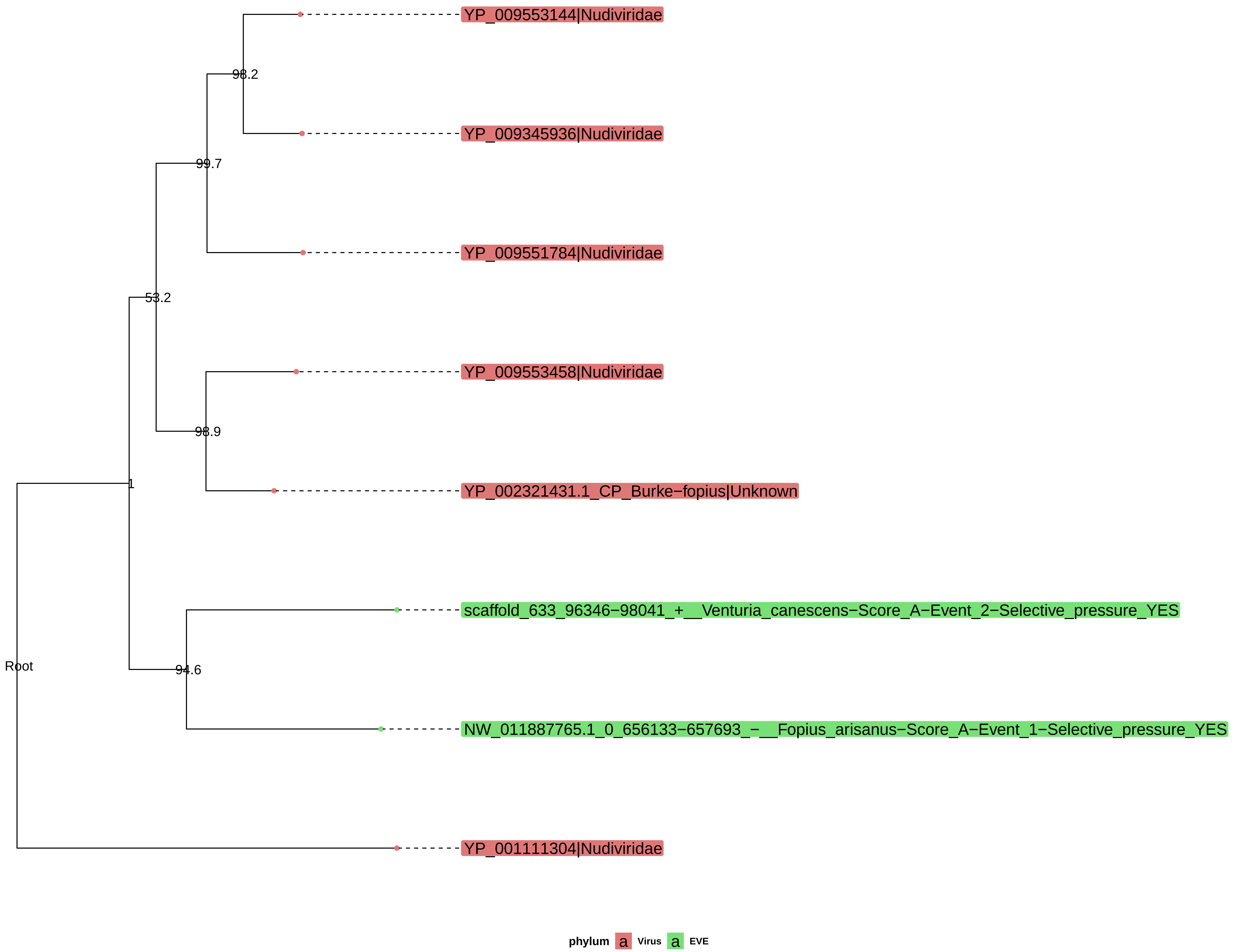

Cluster21733 − families involved : ['Nudiviridae', 'LbFV_like', 'Apis_filamentous−like'] − putative protein : ['PIF−2']

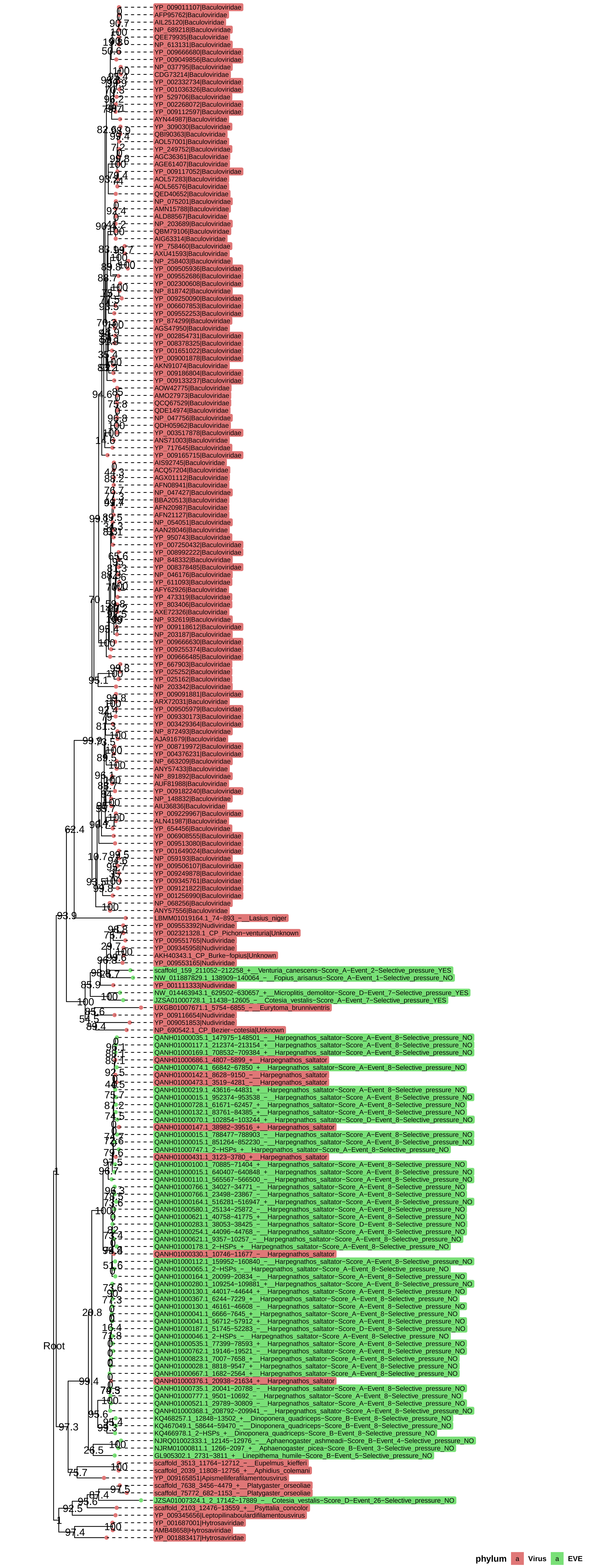

Cluster21204 − families involved : ['Nudiviridae'] − putative protein : ['ACH96189.1 lef−3−like protein']

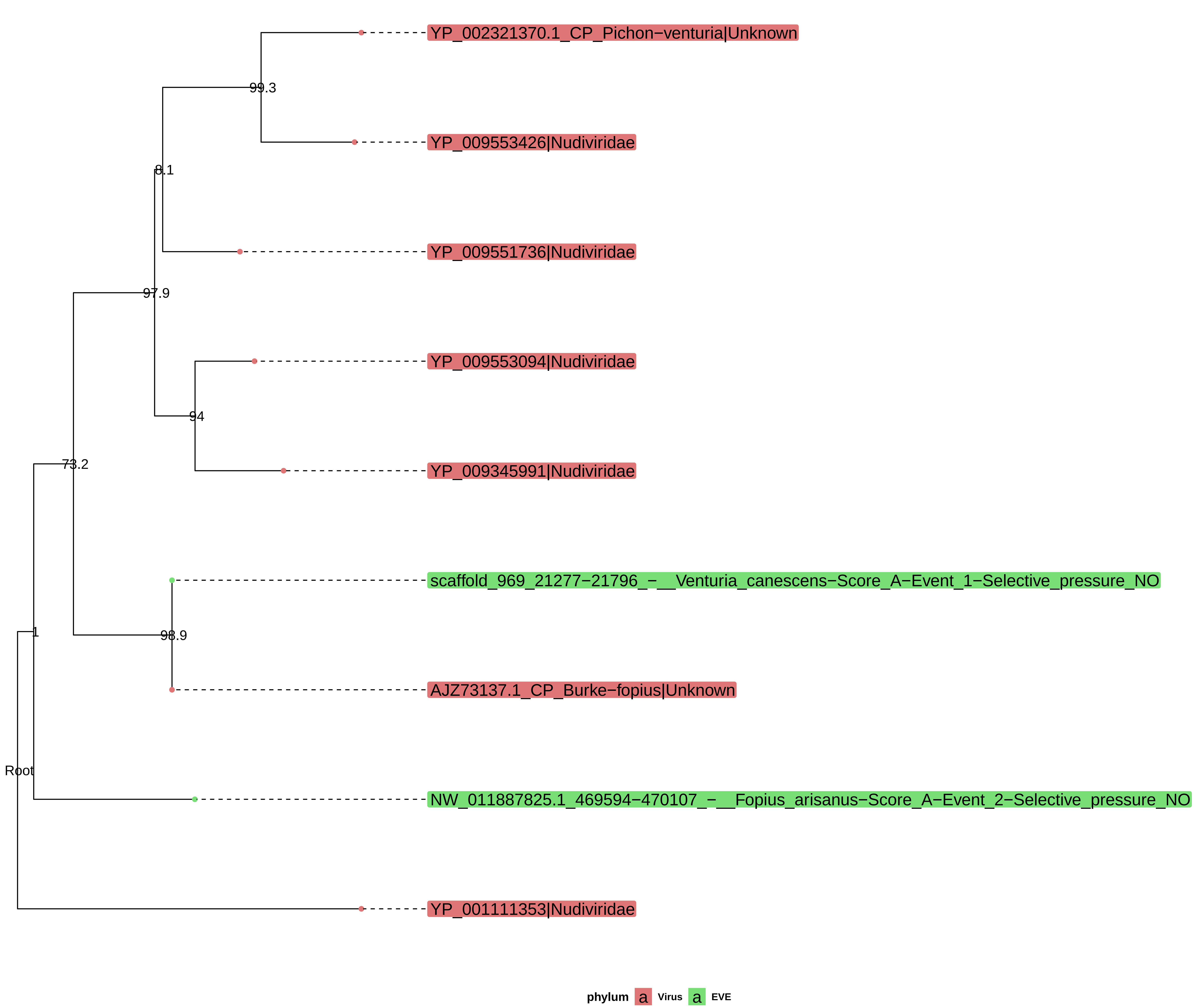

Cluster1971 − families involved : ['Nudiviridae'] − putative protein : ['ACH96157.1 GrBNV gp78−like protein']

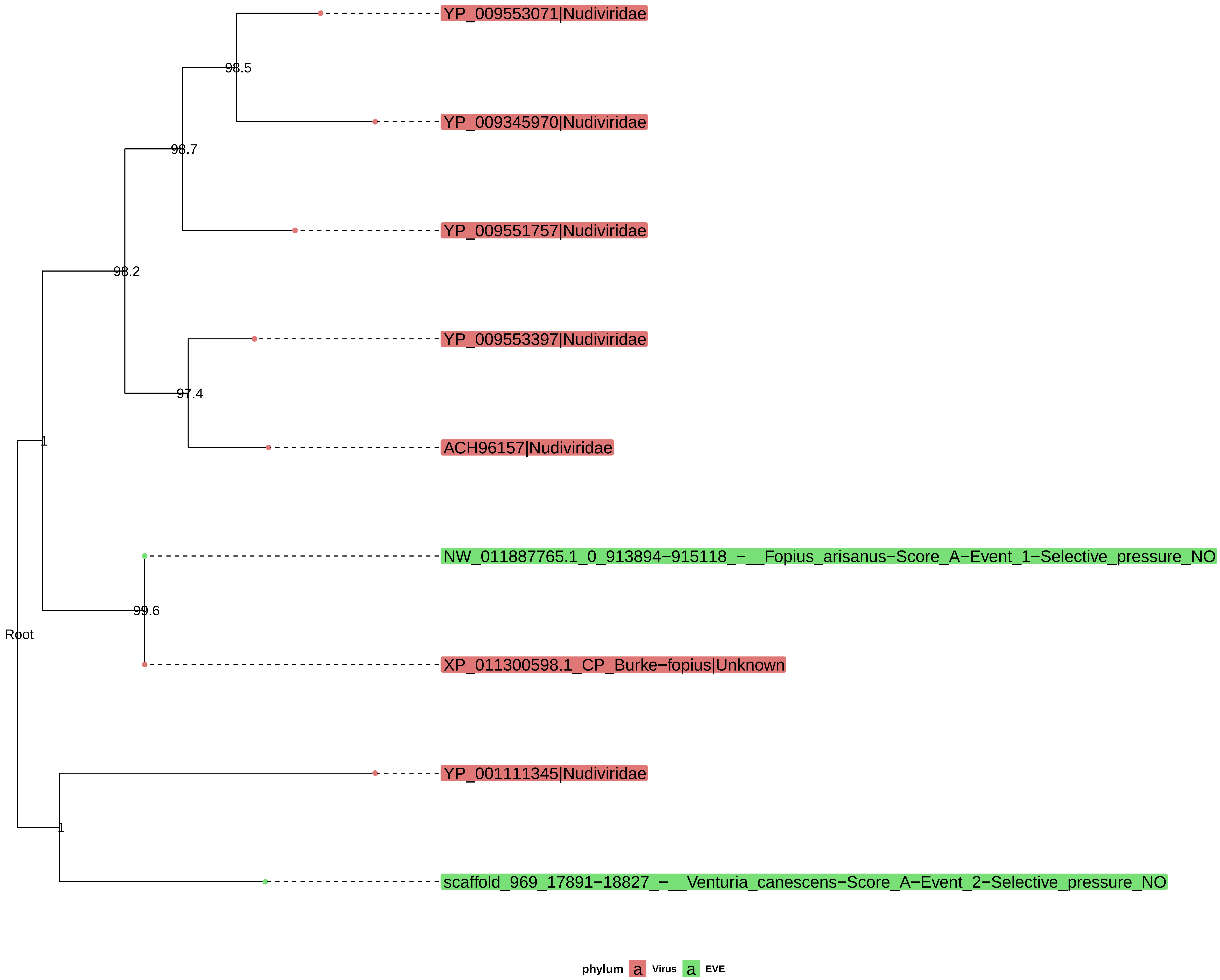

Cluster1964 − families involved : ['Nudiviridae', 'LbFV_like', 'Hytrosaviridae'] − putative protein : ['ODV−E66']

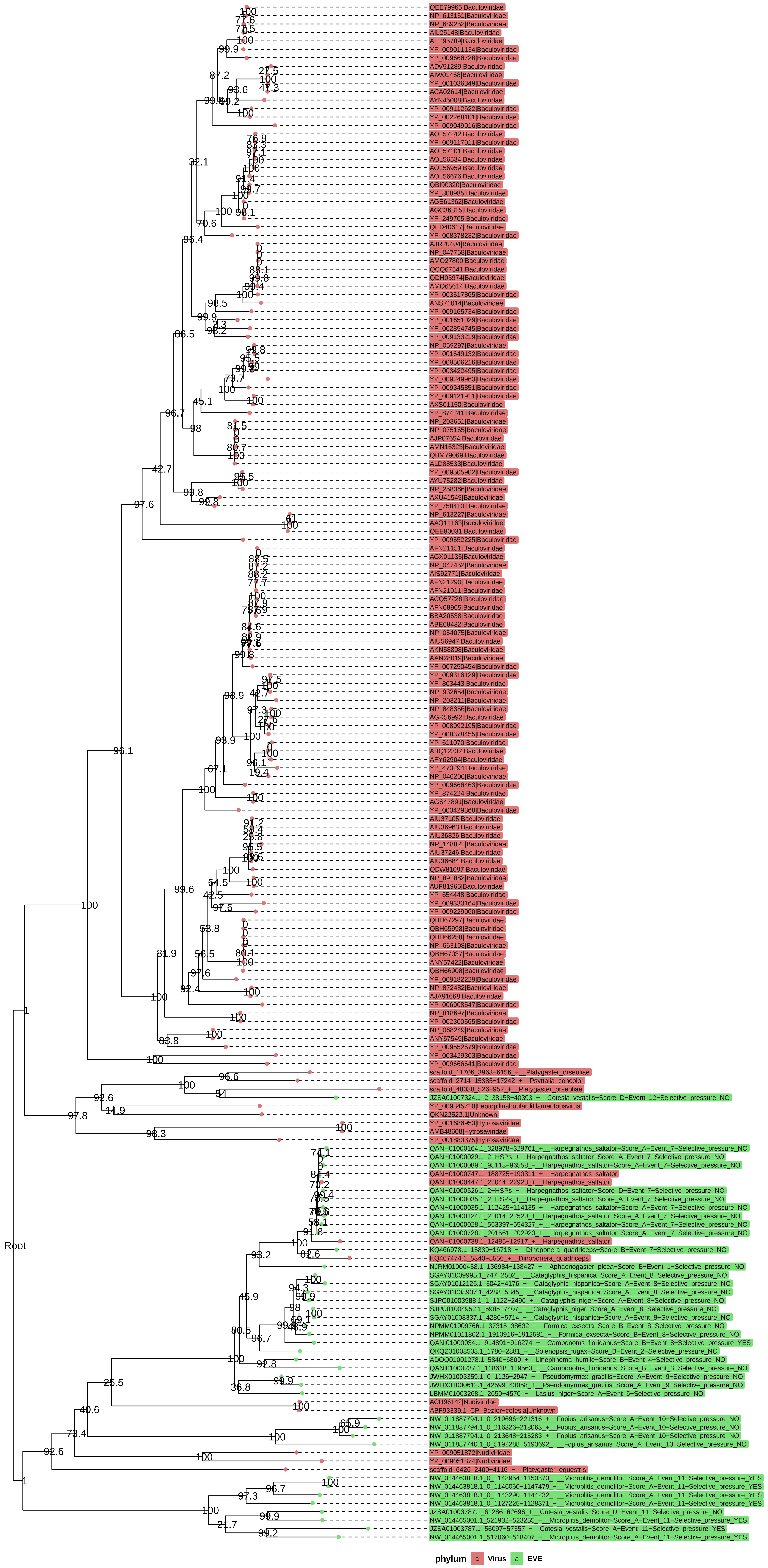

Cluster19564 − families involved : ['Nudiviridae'] − putative protein : ['ACH96202.1 Ac68−like protein (Putative Ac68−like protein)']

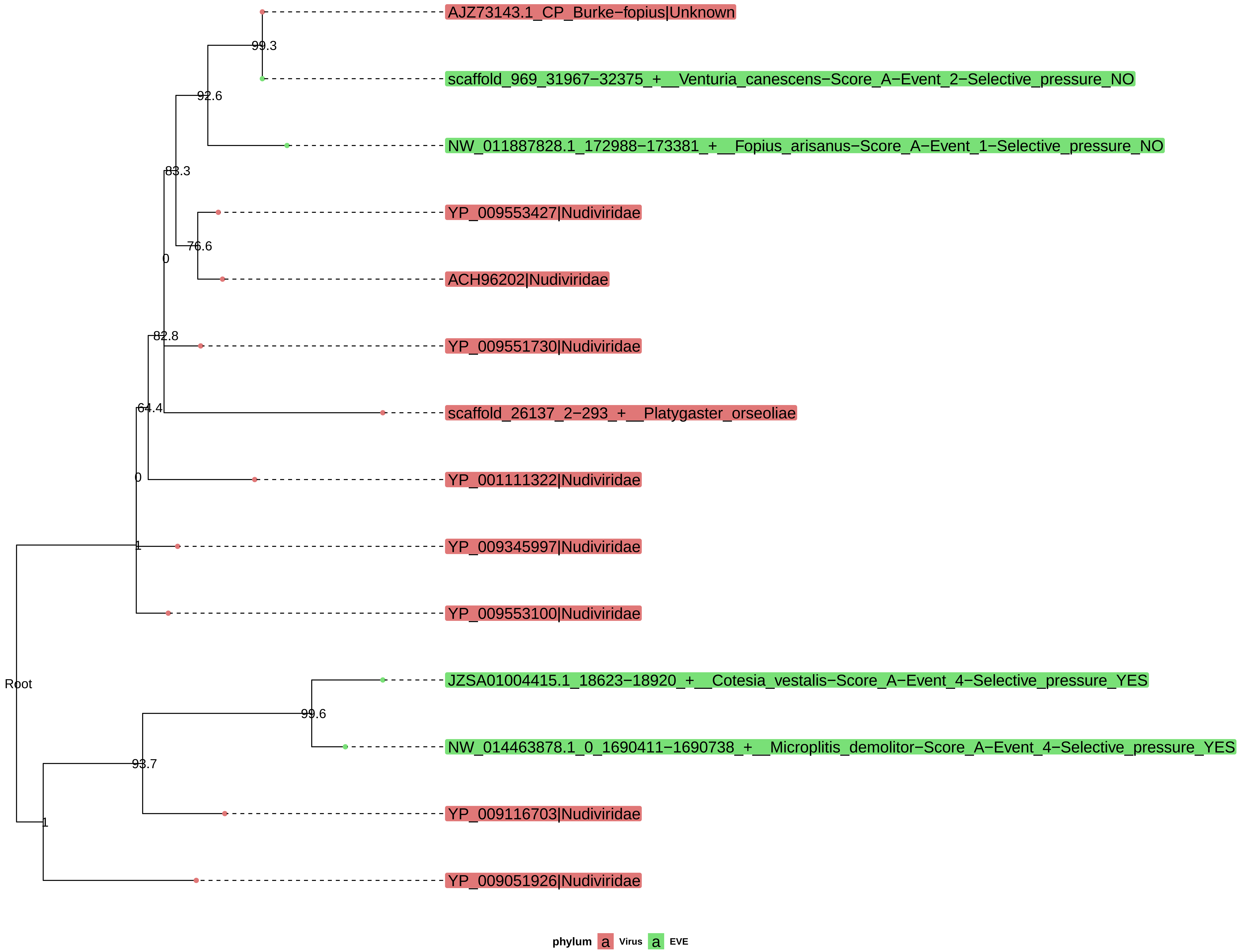

Cluster19347 − families involved : ['Nudiviridae'] − putative protein : ['Vp39']

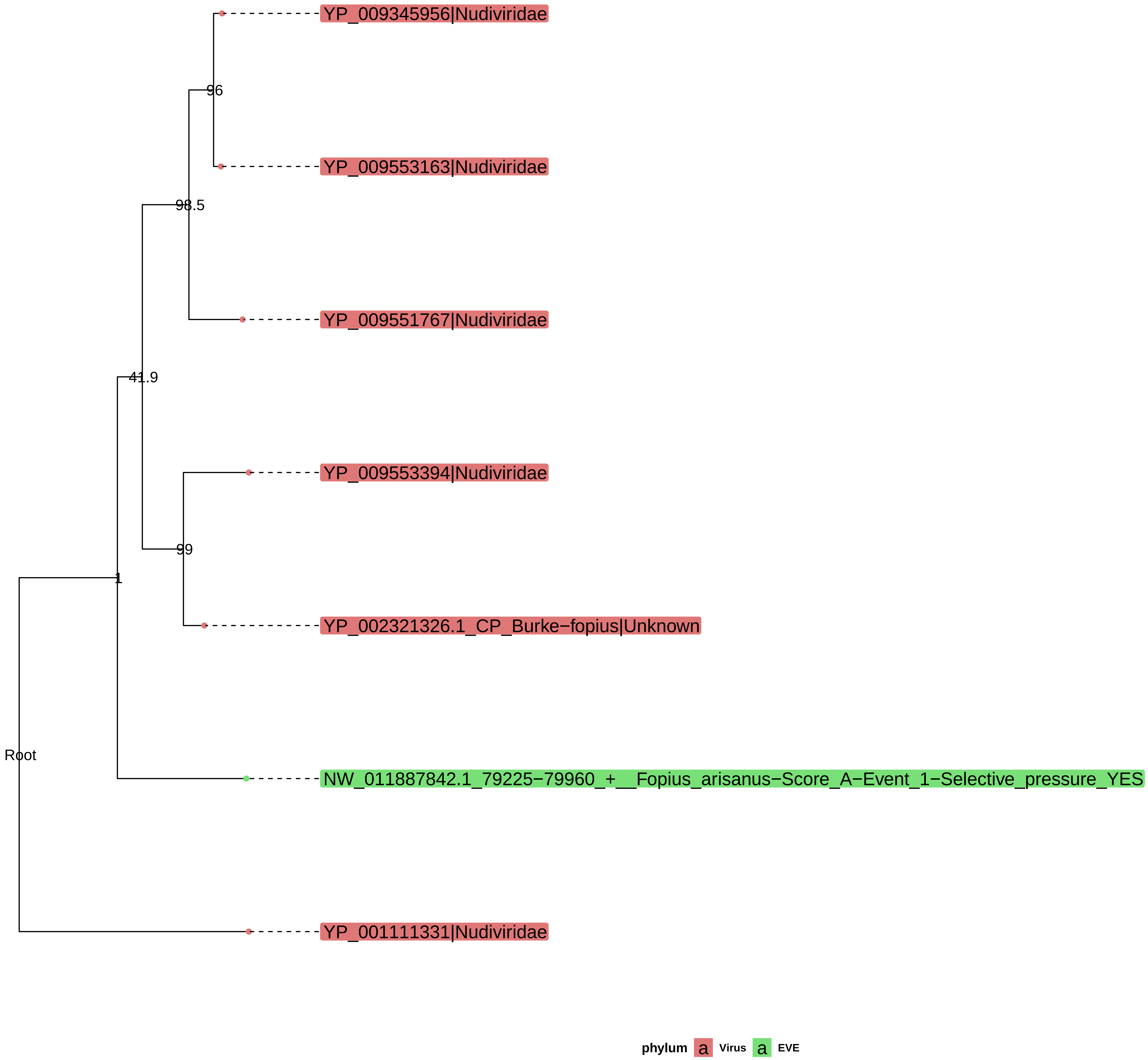

Cluster18349 − families involved : ['Nudiviridae'] − putative protein : ['alpha/beta hydrolase fold']

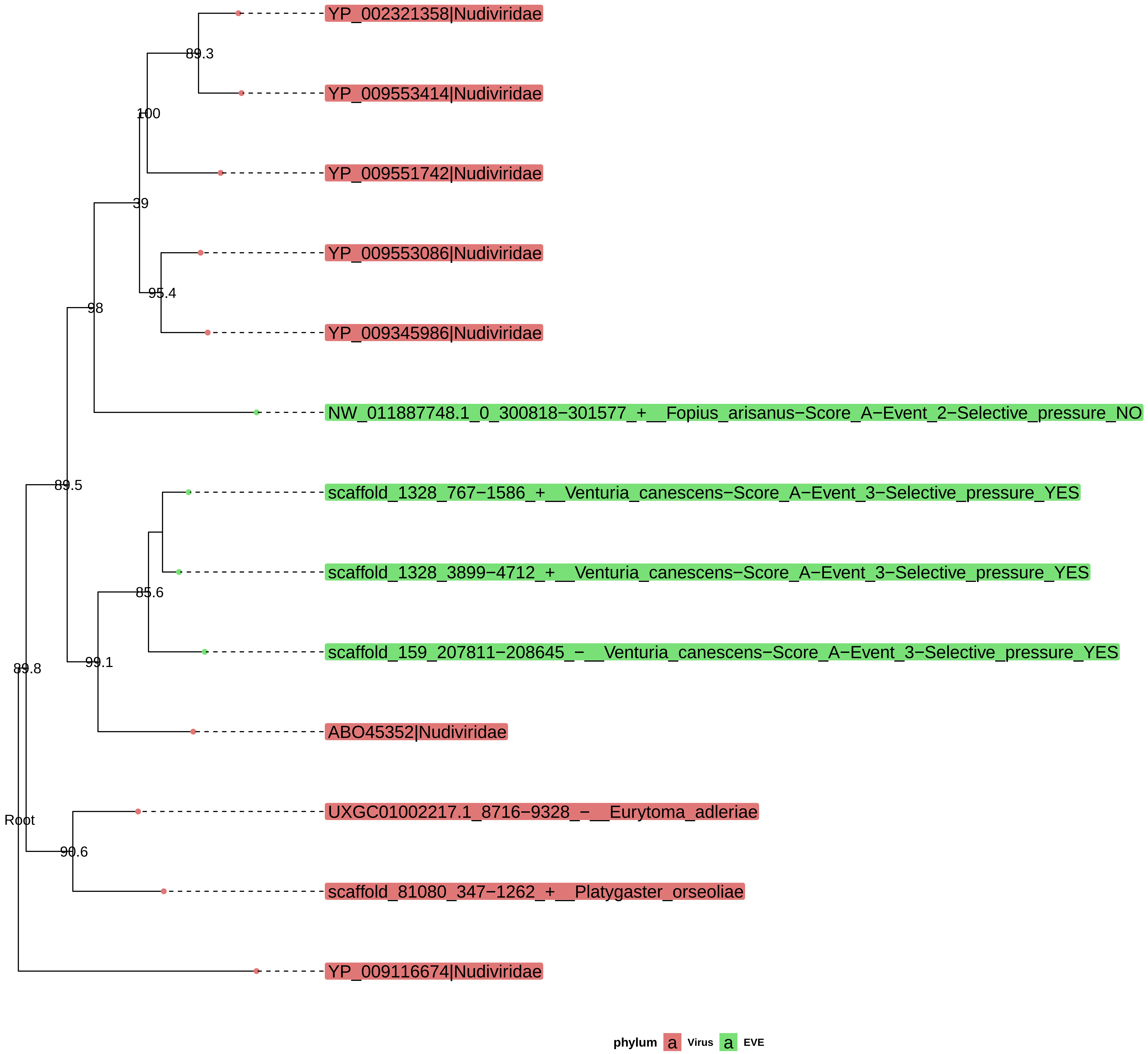

Cluster18059 − families involved : ['Nudiviridae'] − putative protein : ['GrBNV_gp35−like protein']

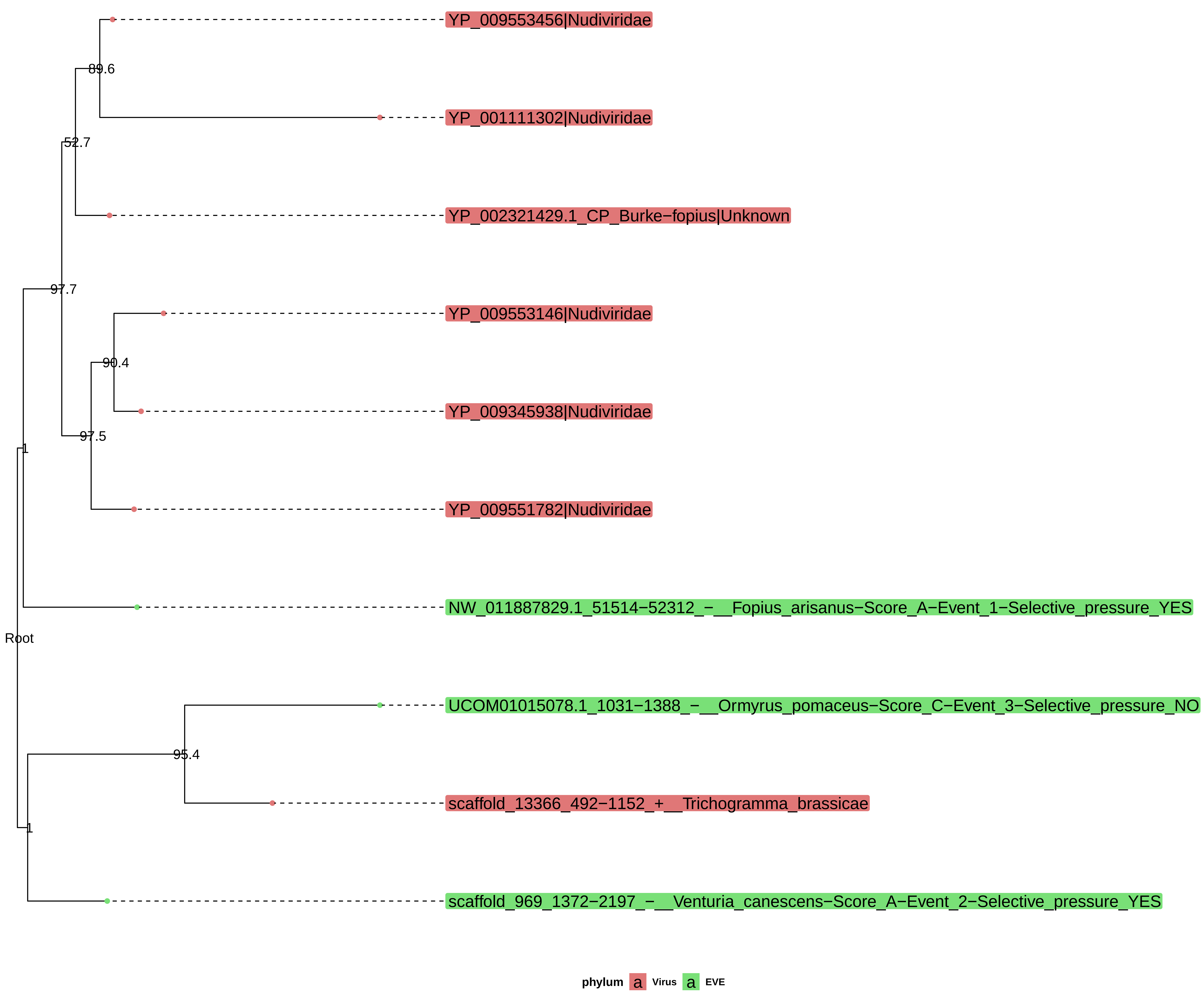

Cluster17704 − families involved : ['Nudiviridae', 'Apis_filamentous−like', 'Baculoviridae'] − putative protein : ['Per os infectivity factor 3']

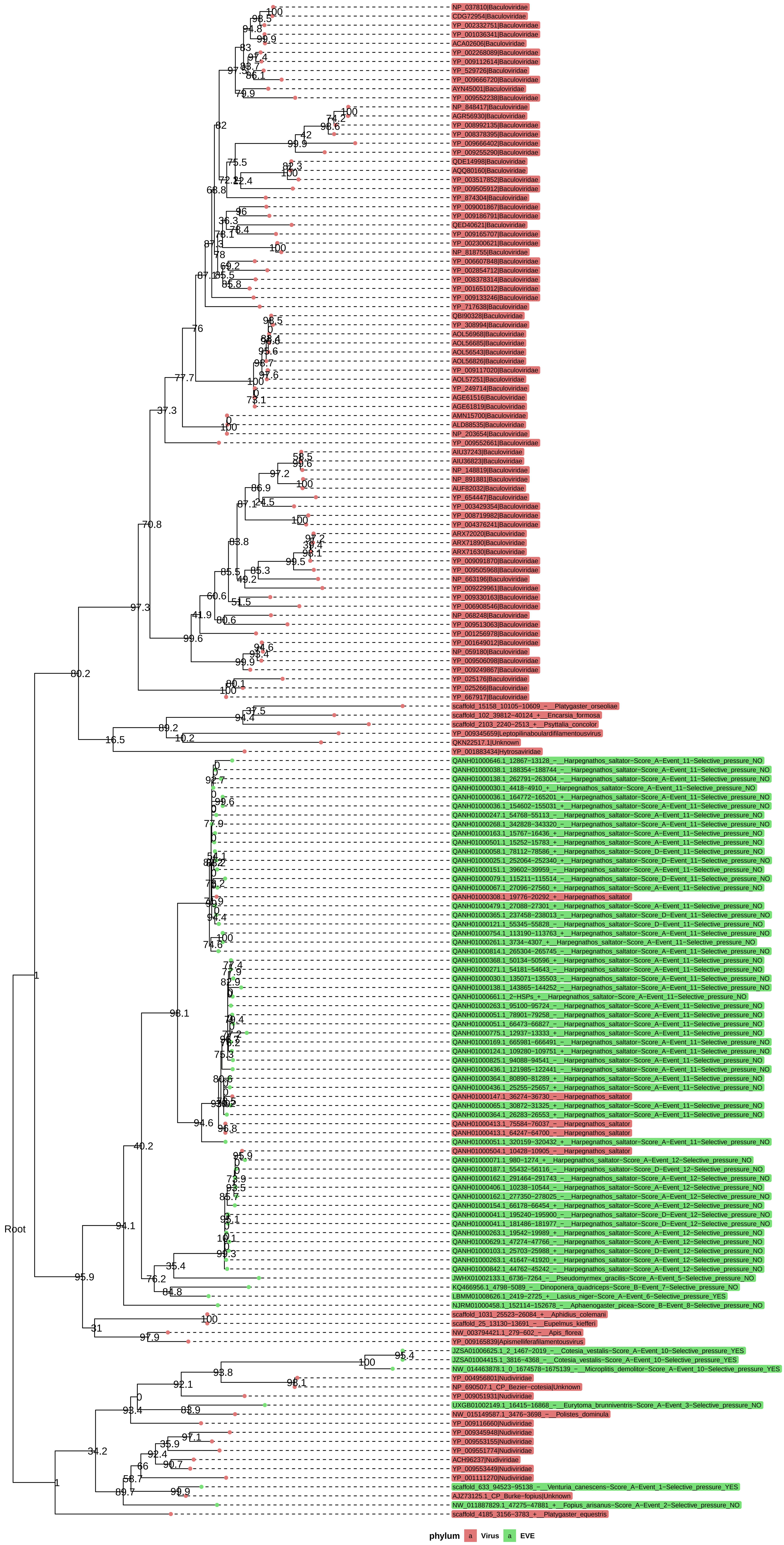

Cluster17609 − families involved : ['Nudiviridae'] − putative protein : ['Helicase']

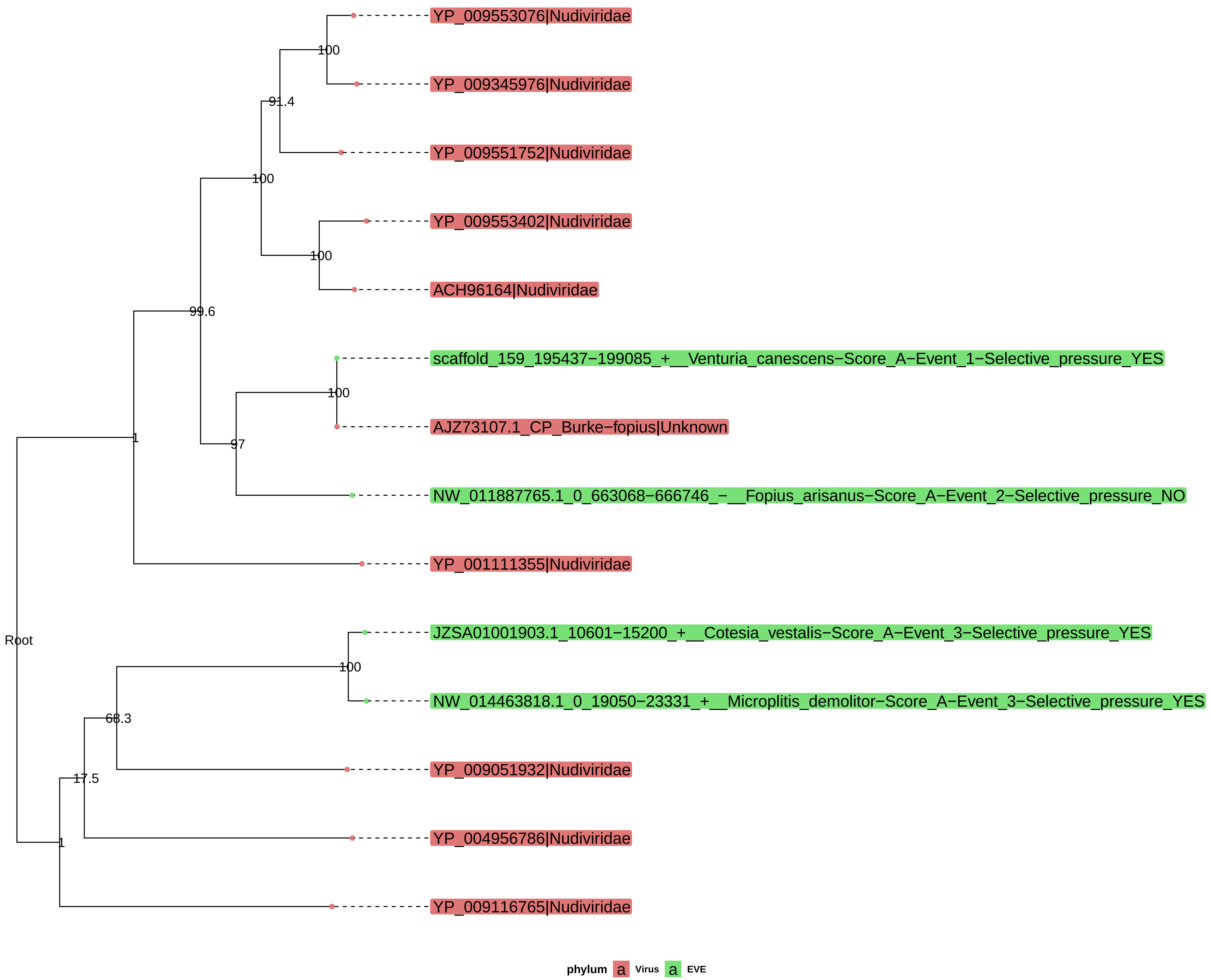

Cluster1694 − families involved : ['Nudiviridae', 'Parvoviridae'] − putative protein : ['ORF5']

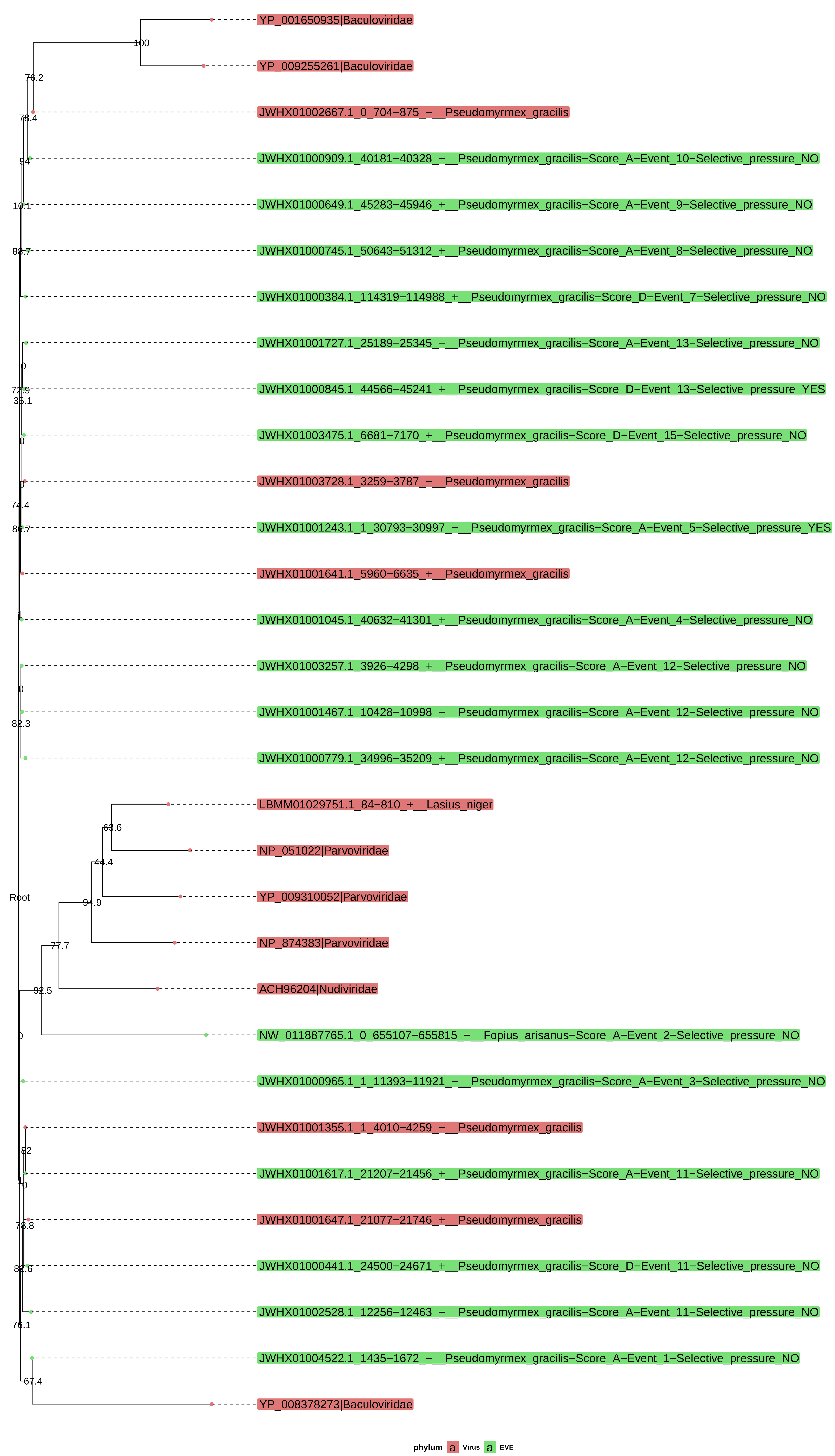

Cluster16413 − families involved : ['Nudiviridae'] − putative protein : ['GrBNV_gp76−like protein']

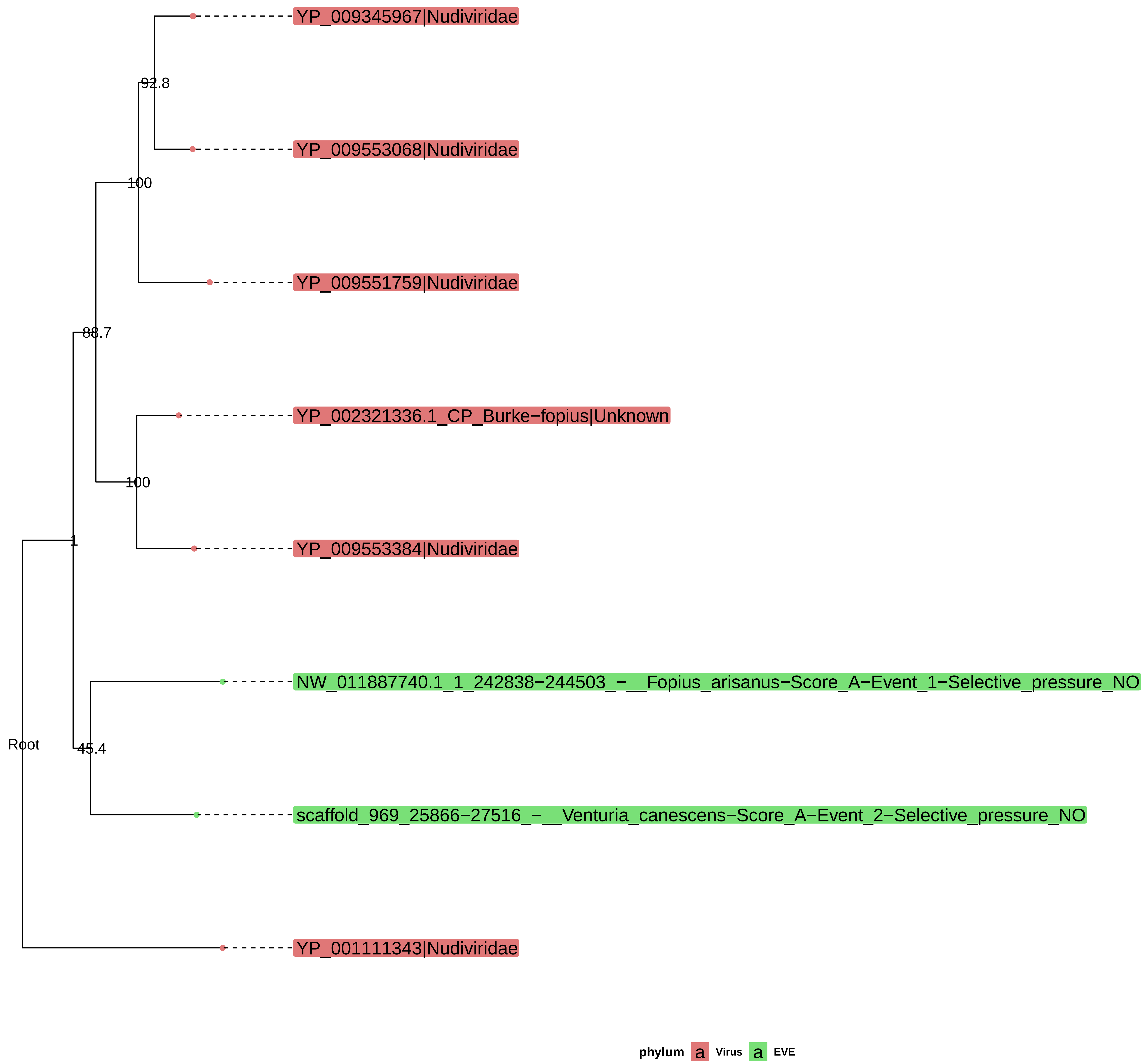

Cluster15164 − families involved : ['Nudiviridae'] − putative protein : ['LEF−9']

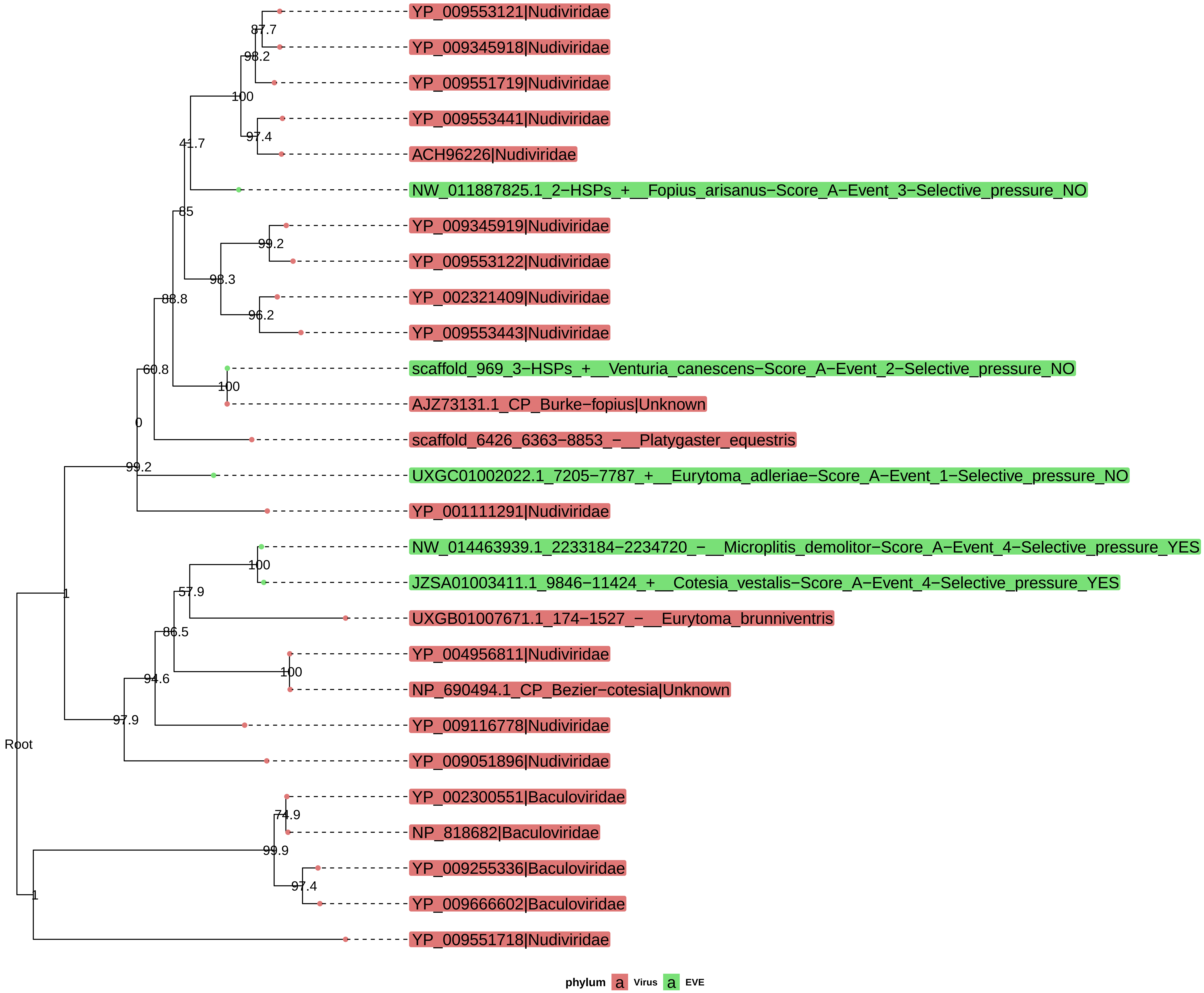

Cluster12087 − families involved : ['Nudiviridae'] − putative protein : ['Lef−5']

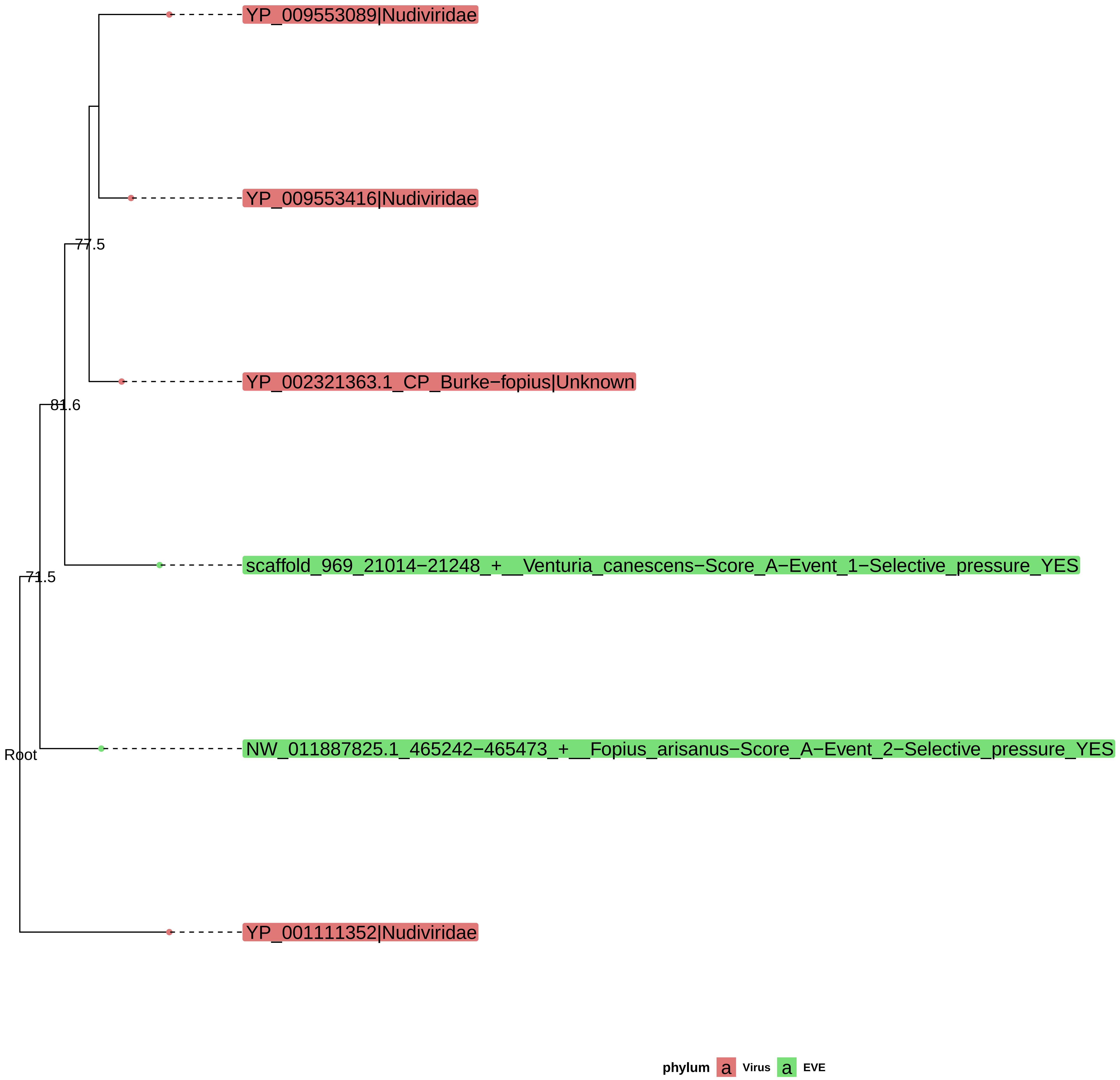

Cluster12013 − families involved : ['Nudiviridae'] − putative protein : ['ACH96159.1 GrBNV gp81−like protein']

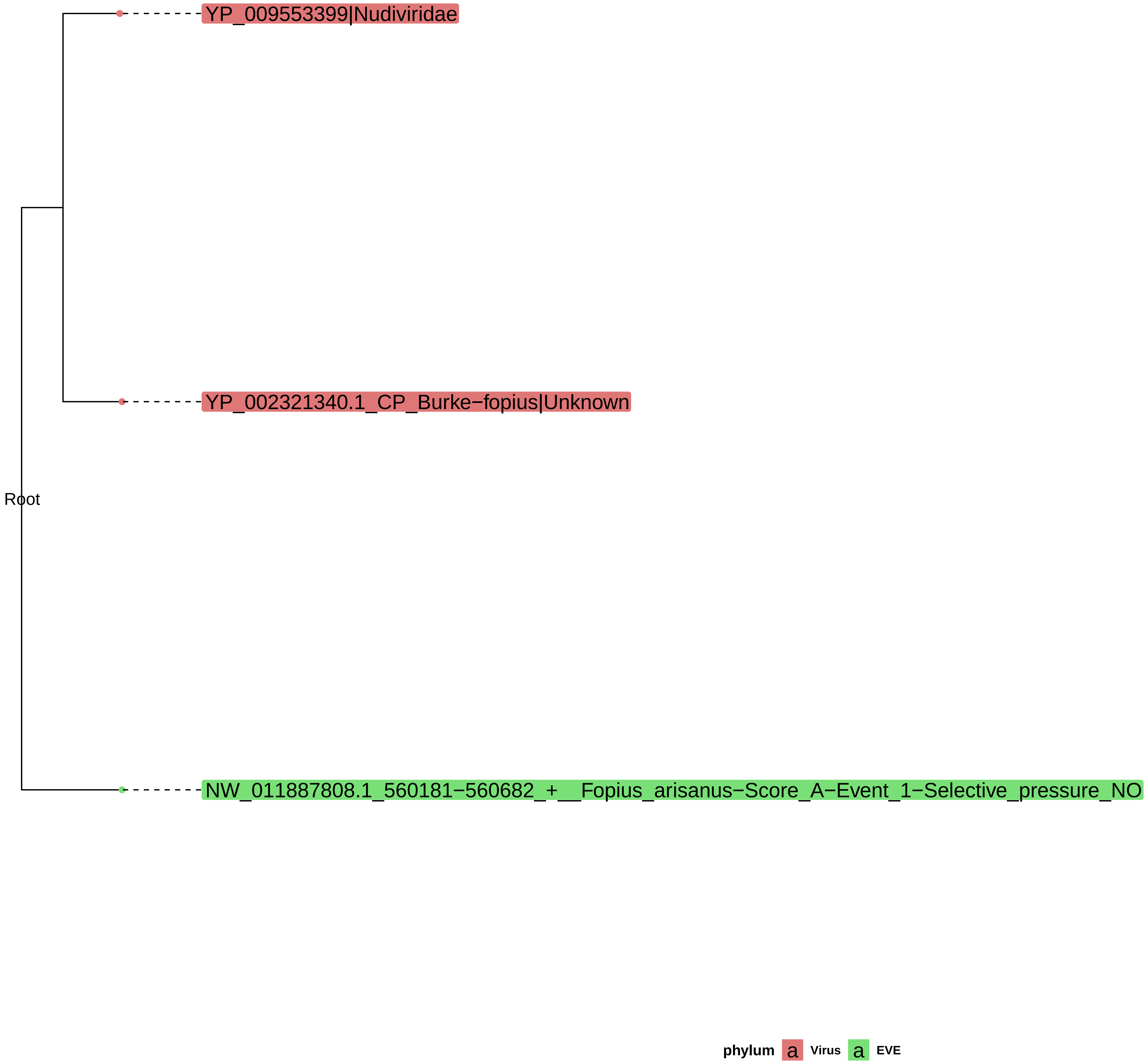

Cluster11825 − families involved : ['Nudiviridae'] − putative protein : ['GrBNV_gp58−like protein']

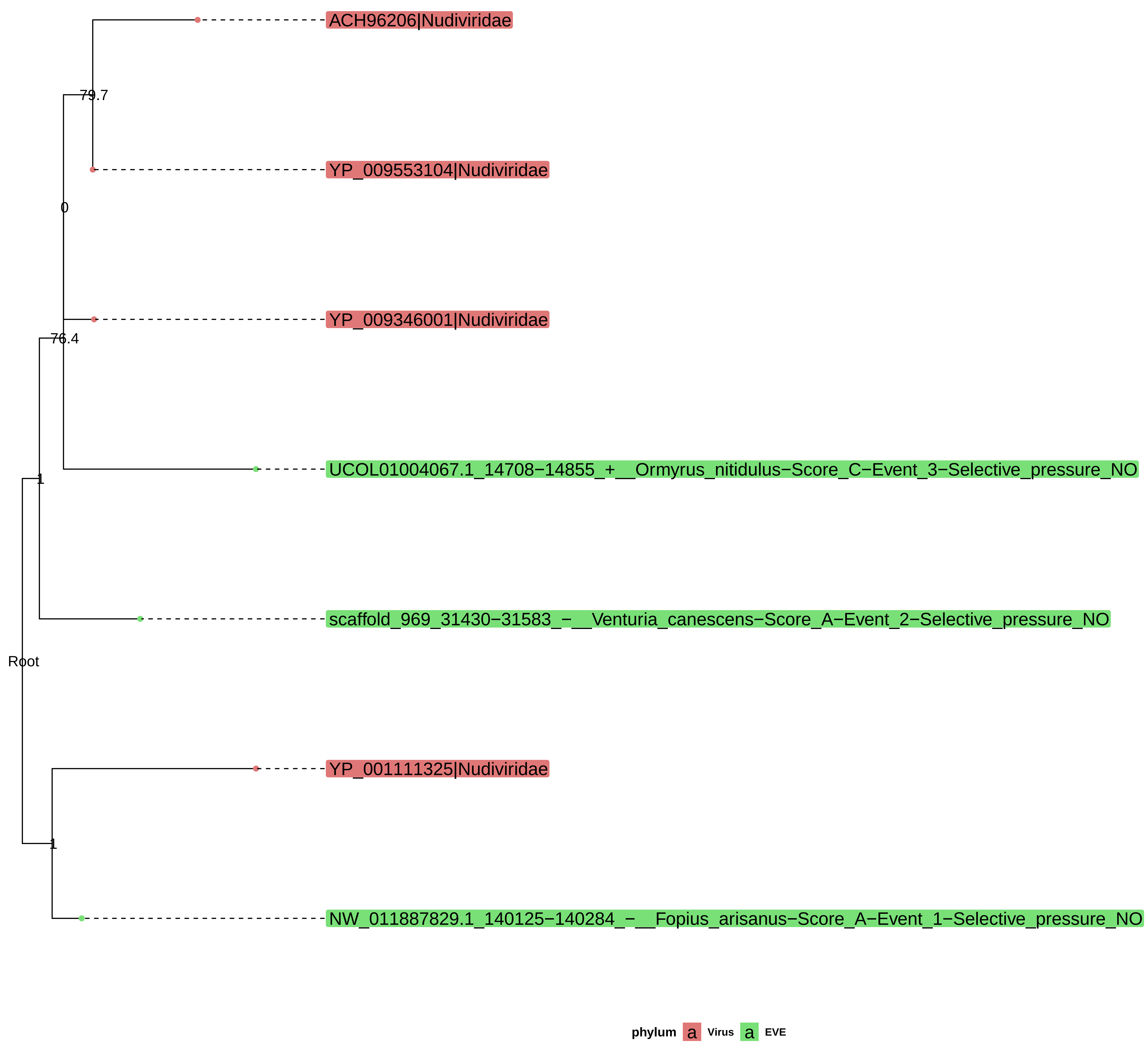

Cluster11455 − families involved : ['Nudiviridae'] − putative protein : ['GrBNV_gp23−like protein']

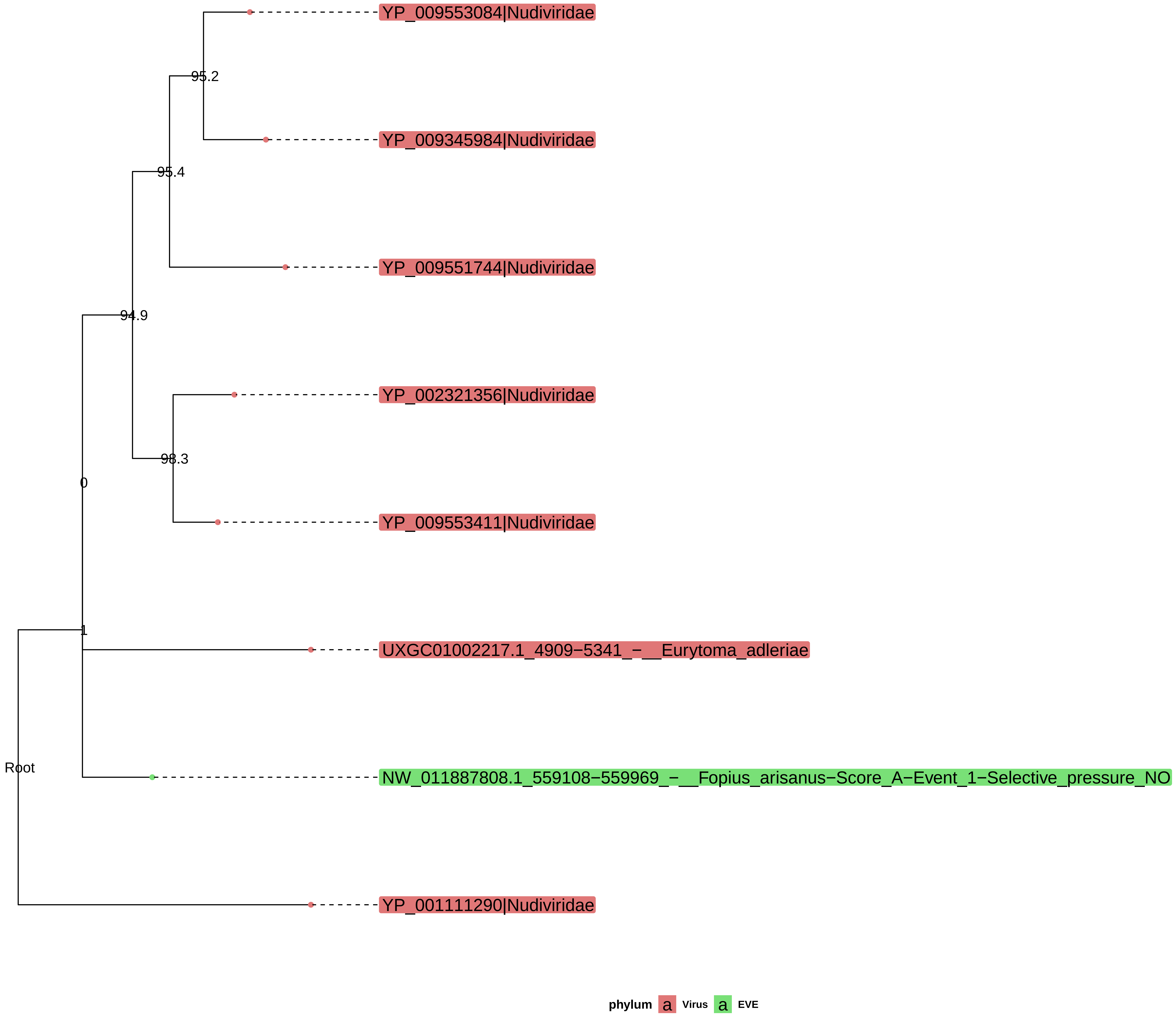

Cluster11238 − families involved : ['Nudiviridae'] − putative protein : ['GrBNV_gp97−like protein']

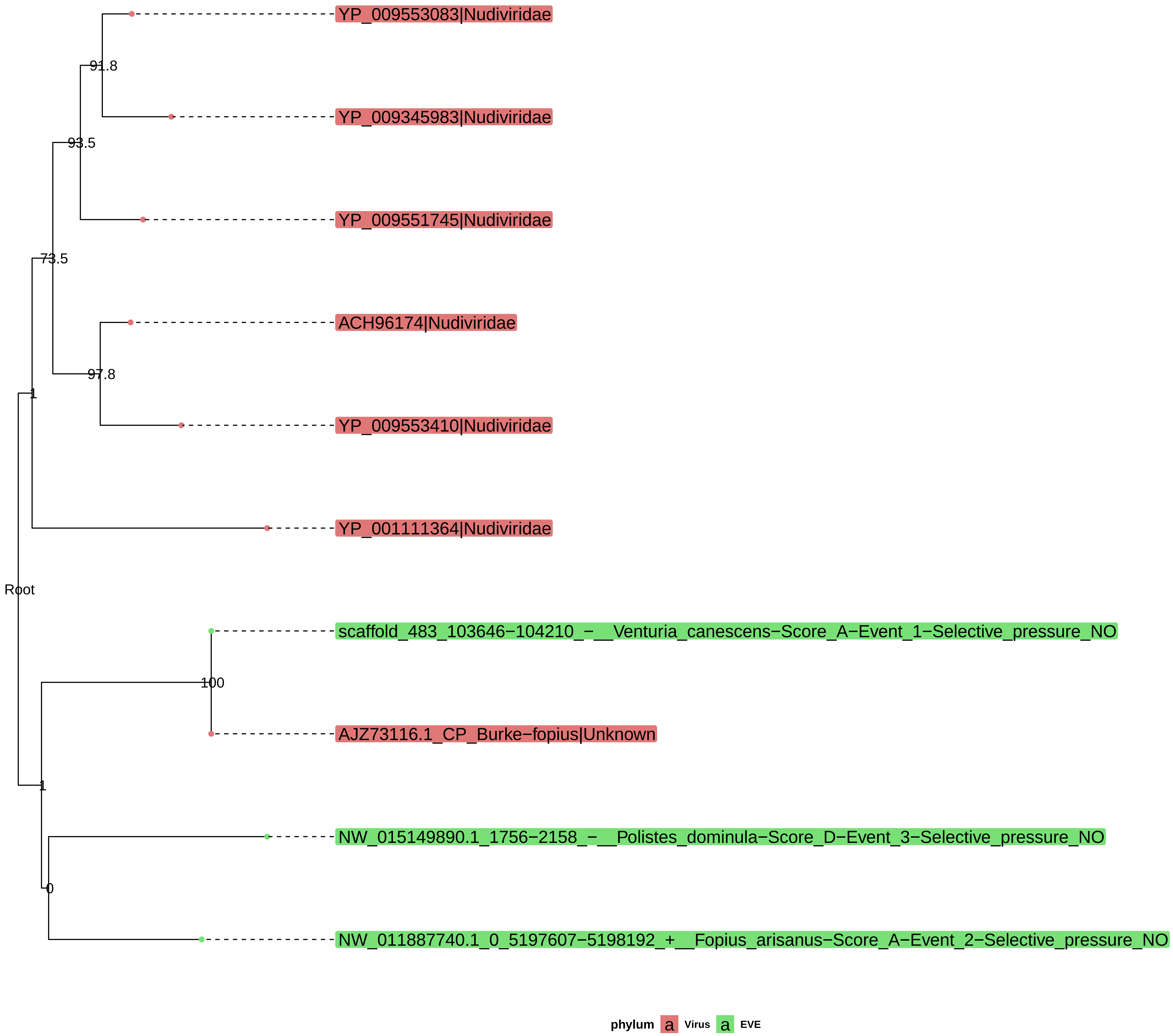

Cluster11092 − families involved : ['Nudiviridae'] − putative protein : ['GrBNV_gp41−like protein']

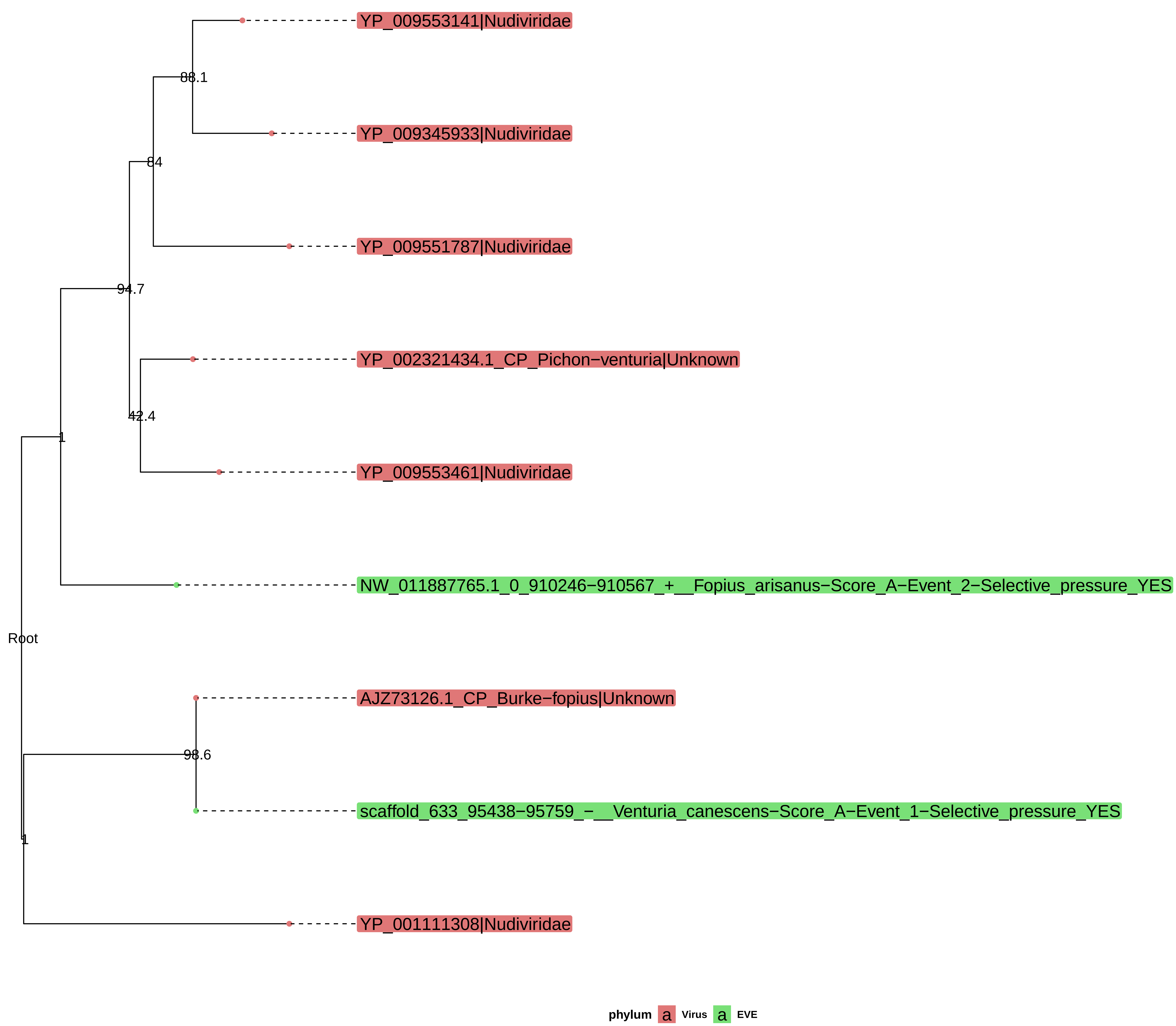

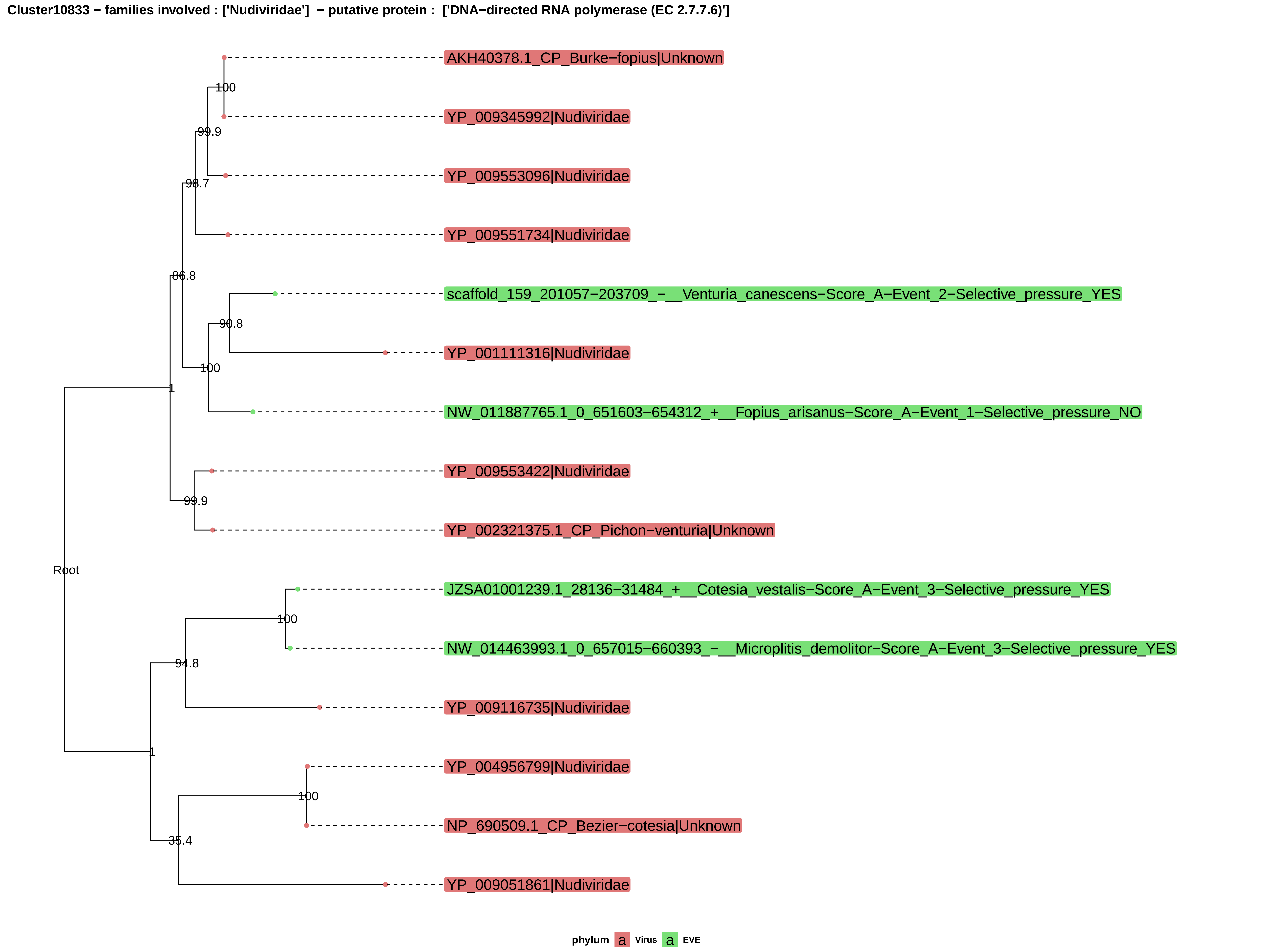

Cluster10833 − families involved : ['Nudiviridae'] − putative protein : ['DNA−directed RNA polymerase (EC 2.7.7.6)']
AKH40378.1_CP_Burke−fopius|Unknown
100
YP_009345992|Nudiviridae
99.9
YP_009553096|Nudiviridae
98.7
YP_009551734|Nudiviridae
86.8
scaffold_159_201057−203709_−__Venturia_canescens−Score_A−Event_2−Selective_pressure_YES
90.8
YP_001111316|Nudiviridae
100
1
NW_011887765.1_0_651603−654312_+__Fopius_arisanus−Score_A−Event_1−Selective_pressure_NO
YP_009553422|Nudiviridae
99.9
YP_002321375.1_CP_Pichon−venturia|Unknown
Root
JZSA01001239.1_28136−31484_+__Cotesia_vestalis−Score_A−Event_3−Selective_pressure_YES
100
NW_014463993.1_0_657015−660393_−__Microplitis_demolitor−Score_A−Event_3−Selective_pressure_YES
94.8
YP_009116735|Nudiviridae
1
YP_004956799|Nudiviridae
100
NP_690509.1_CP_Bezier−cotesia|Unknown
35.4
YP_009051861|Nudiviridae
phylum a Virus a EVE

Cluster10771 − families involved : ['Nudiviridae'] − putative protein : ['GrBNV_gp93−like protein']

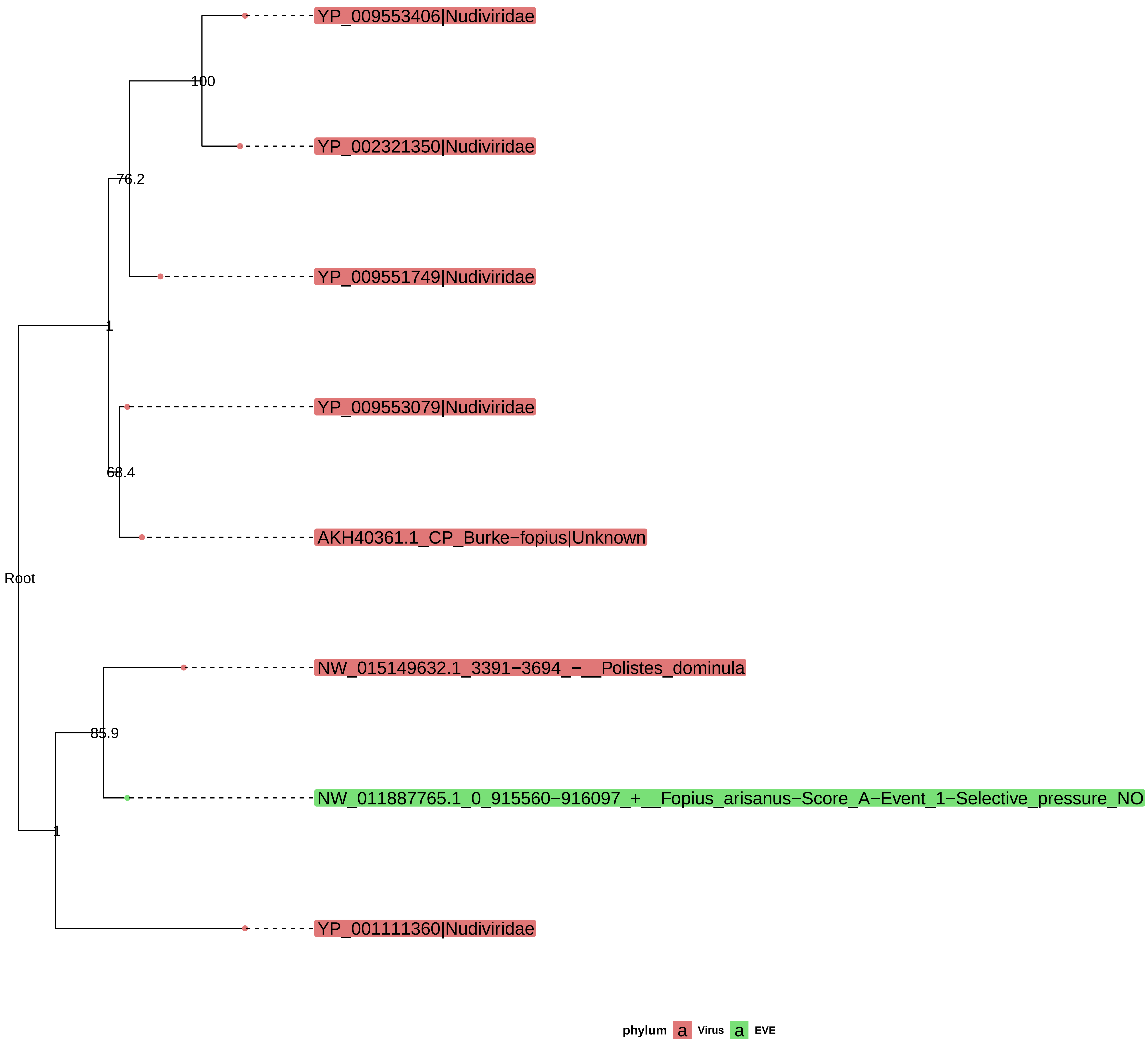

Cluster8973 − families involved : ['LbFV_like'] − putative protein : ['mRNA capping enzyme, catalytic domain']

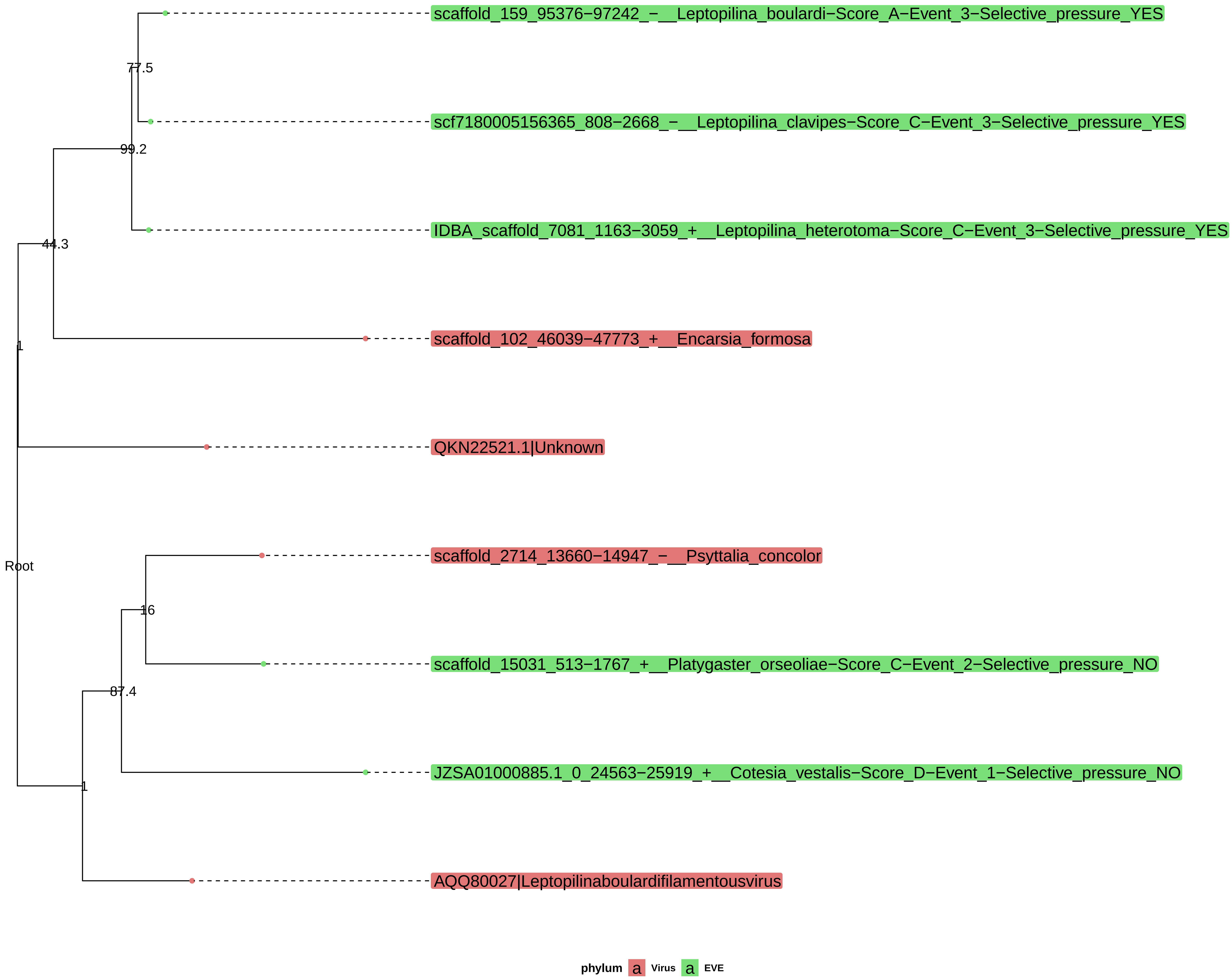

Cluster5269 − families involved : ['LbFV_like'] − putative protein : [nan]

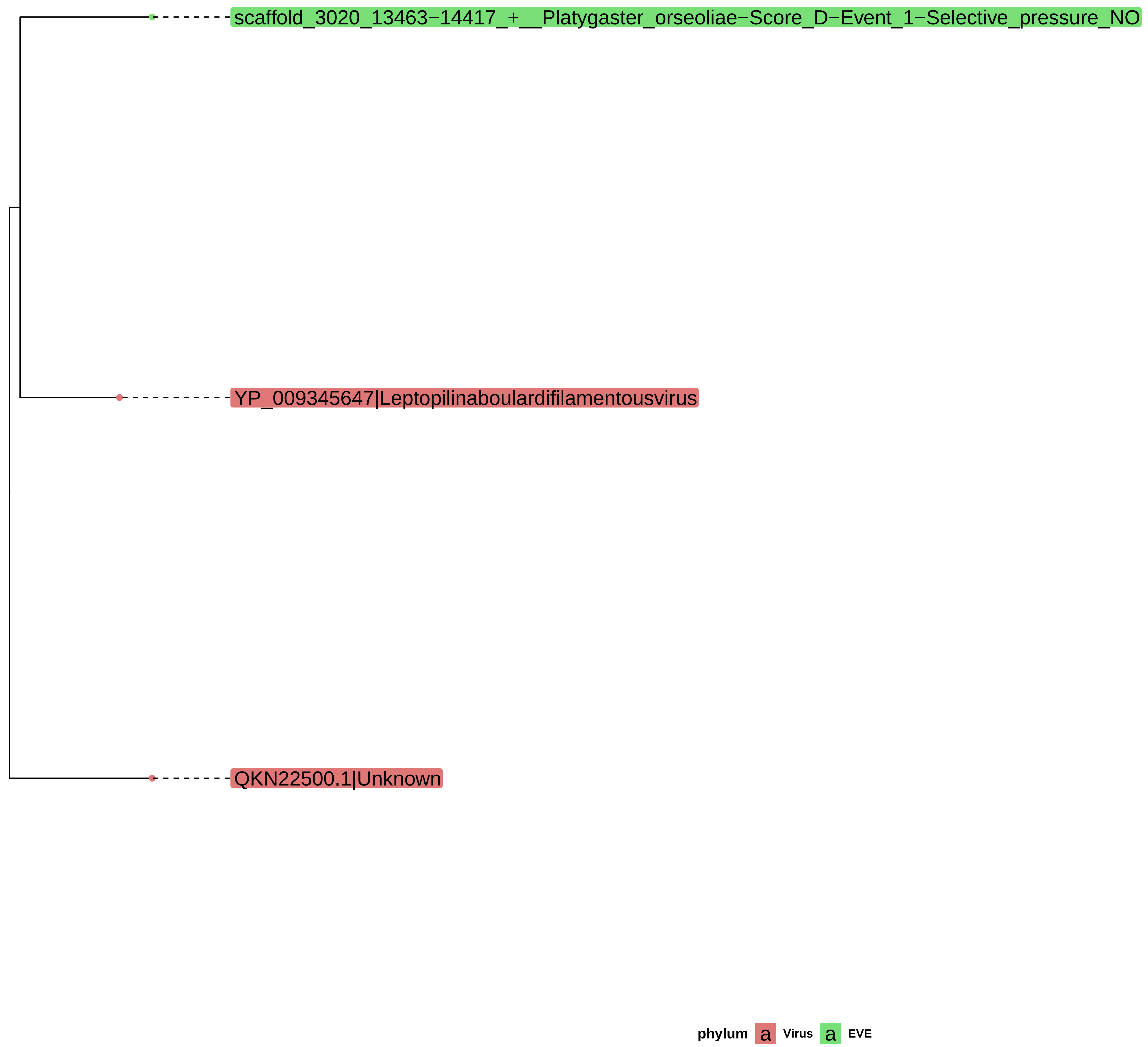

Cluster3614 − families involved : ['LbFV_like', 'Nudiviridae', 'Apis_filamentous−like', 'Baculoviridae'] − putative protein : ['PIF−1']

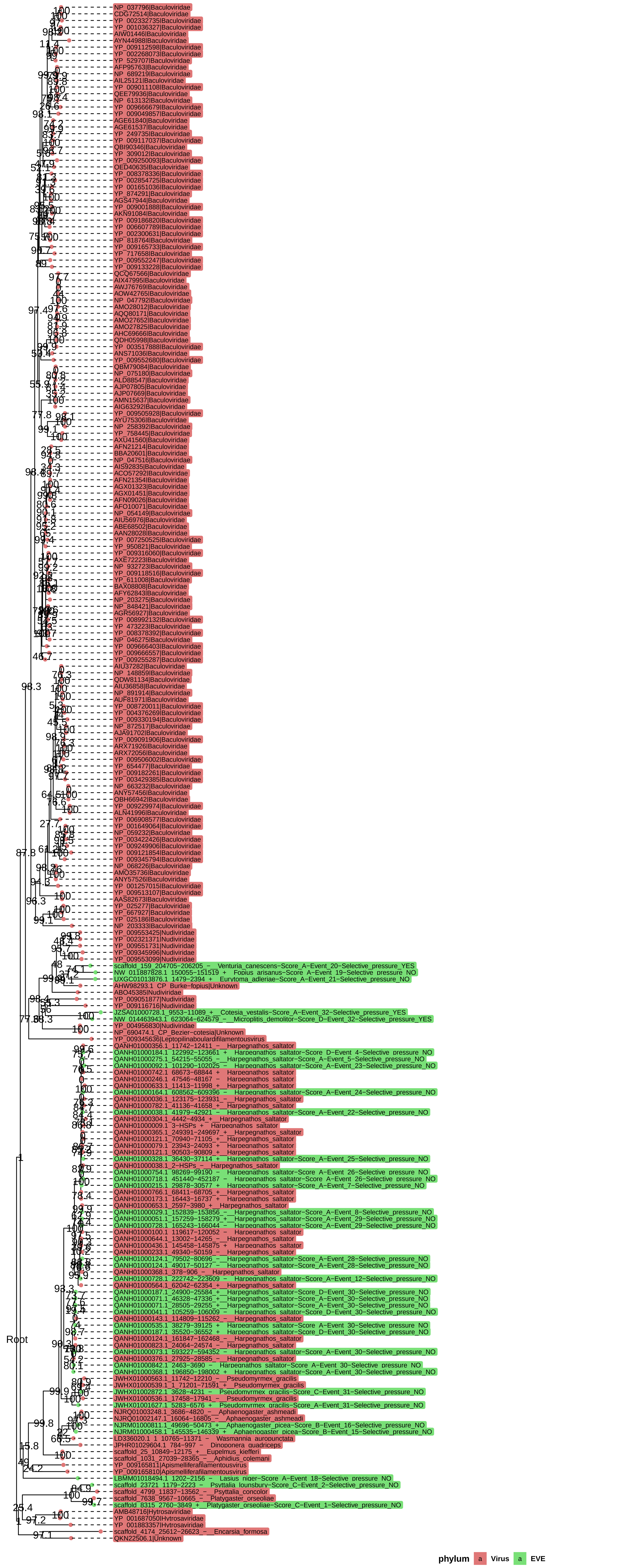

Cluster27203 − families involved : ['LbFV_like', 'Apis_filamentous−like'] − putative protein : ['DNA−directed DNA polymerase (EC 2.7.7.7)']

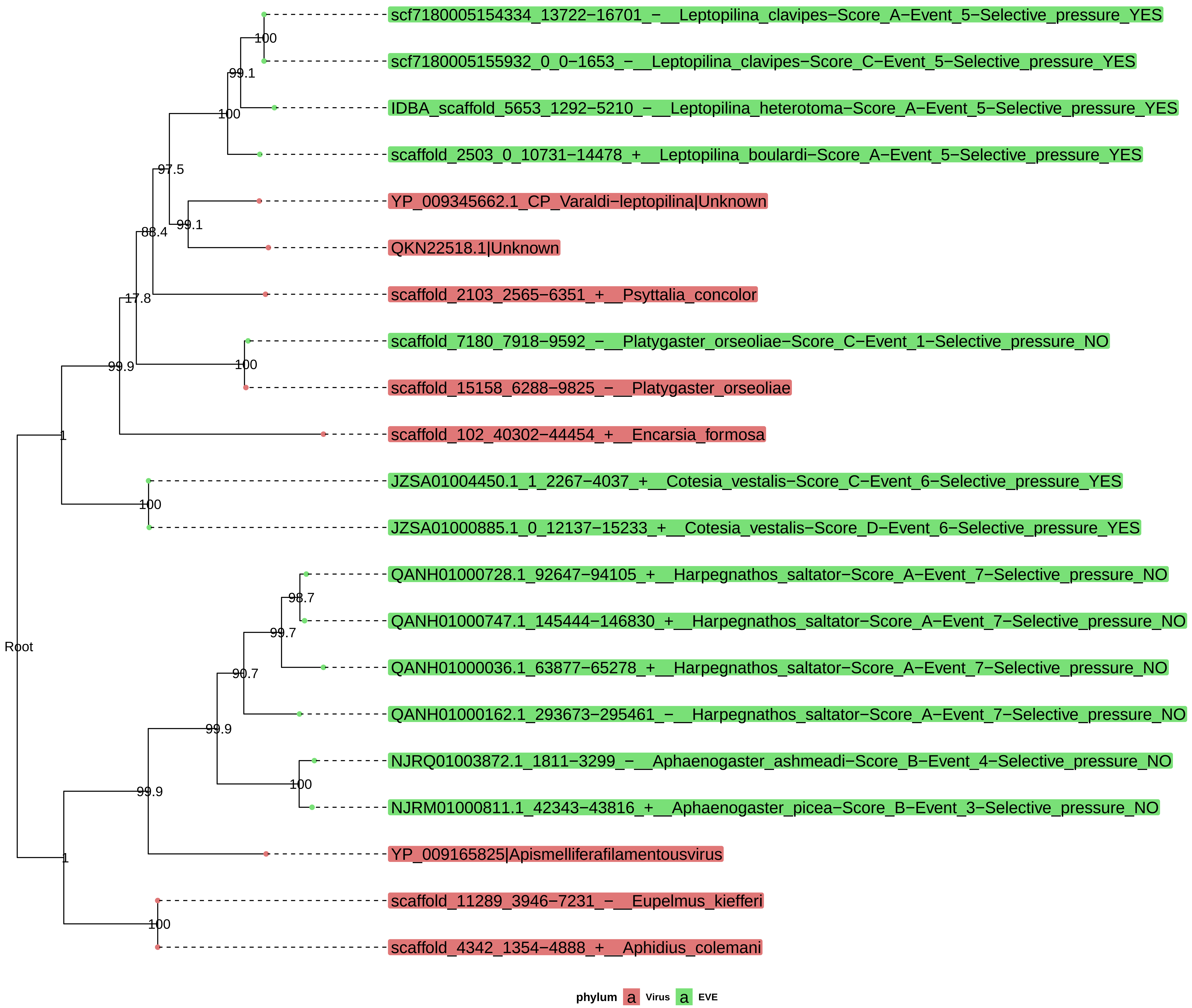

Cluster26675 − families involved : ['LbFV_like', 'Apis_filamentous−like'] − putative protein : ['EbsA−like protein']

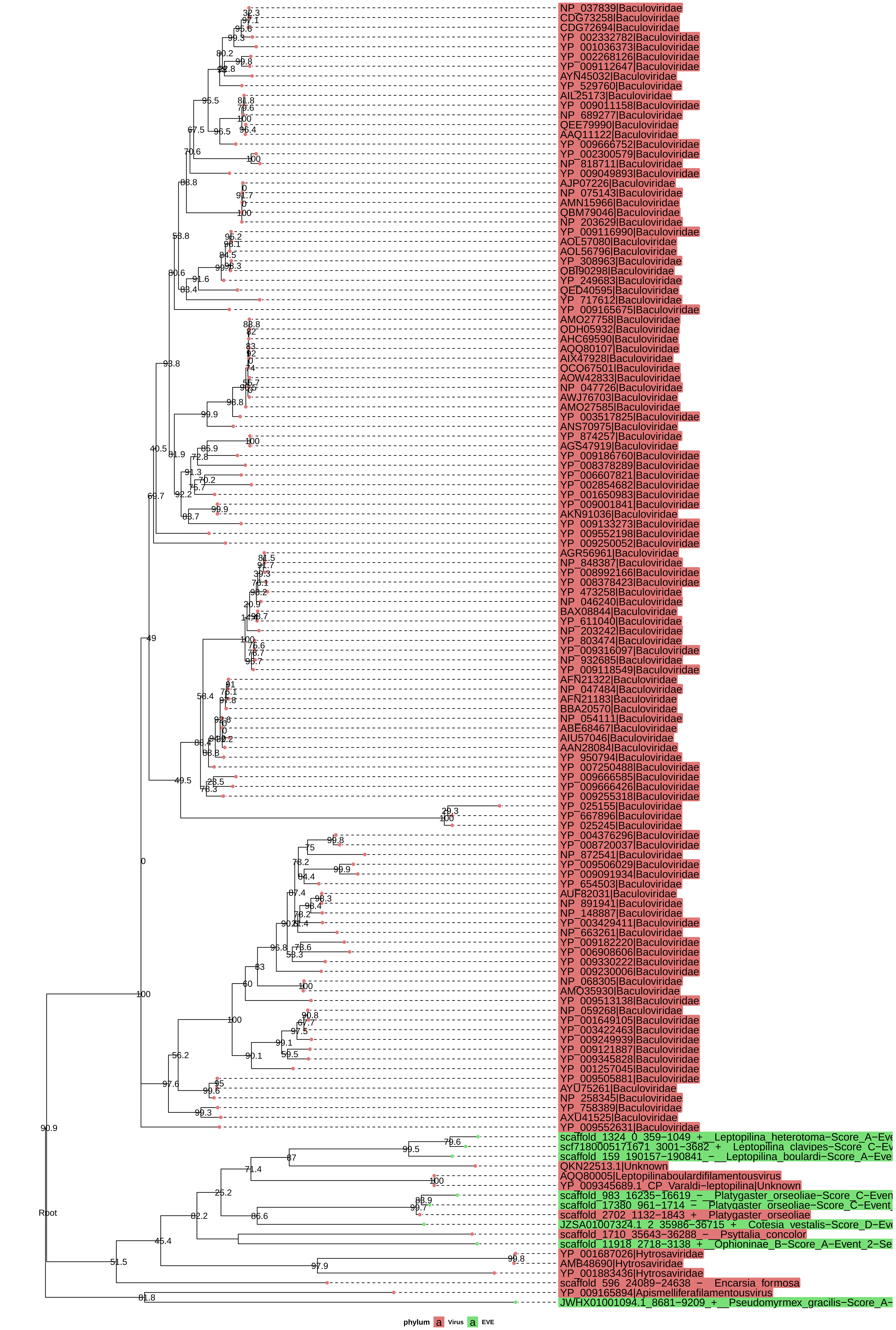

Cluster25710 − families involved : ['LbFV_like'] − putative protein : ['Putative ORF2']

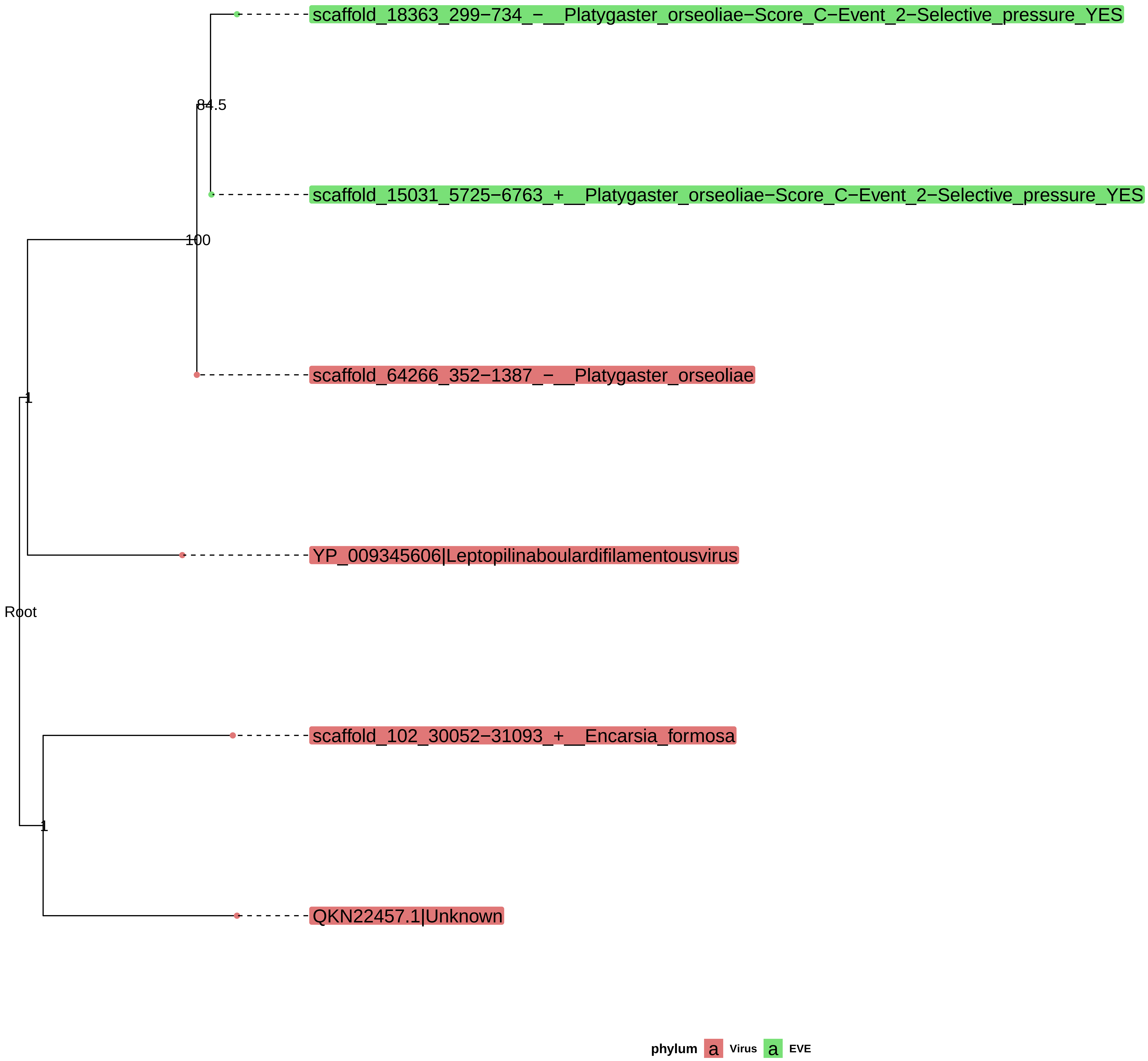

Cluster23517 − families involved : ['LbFV_like'] − putative protein : ['Putative ORF20']

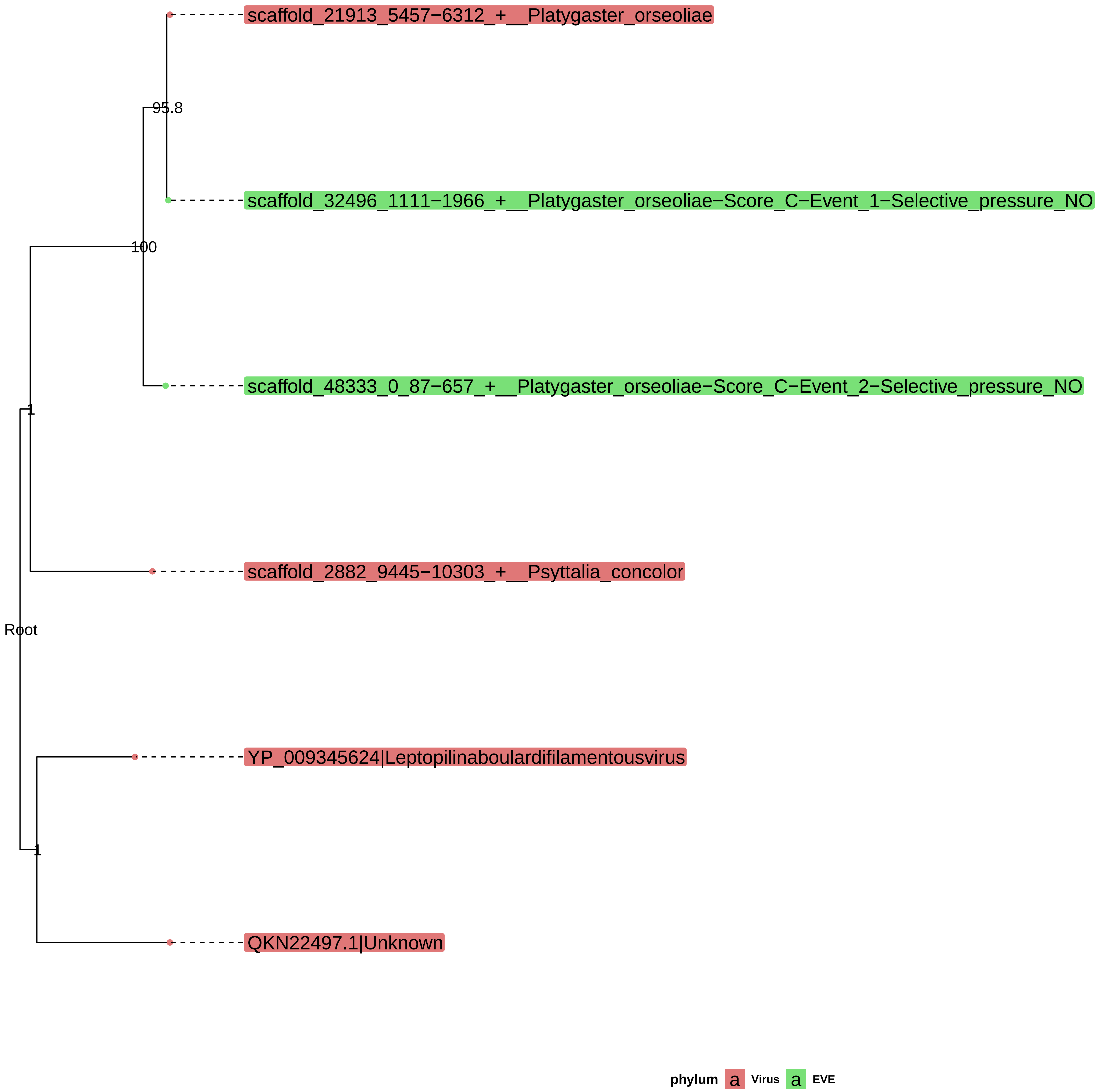

Cluster21657 − families involved : ['LbFV_like'] − putative protein : ['Putative lecithine cholesterol acyltransferase']

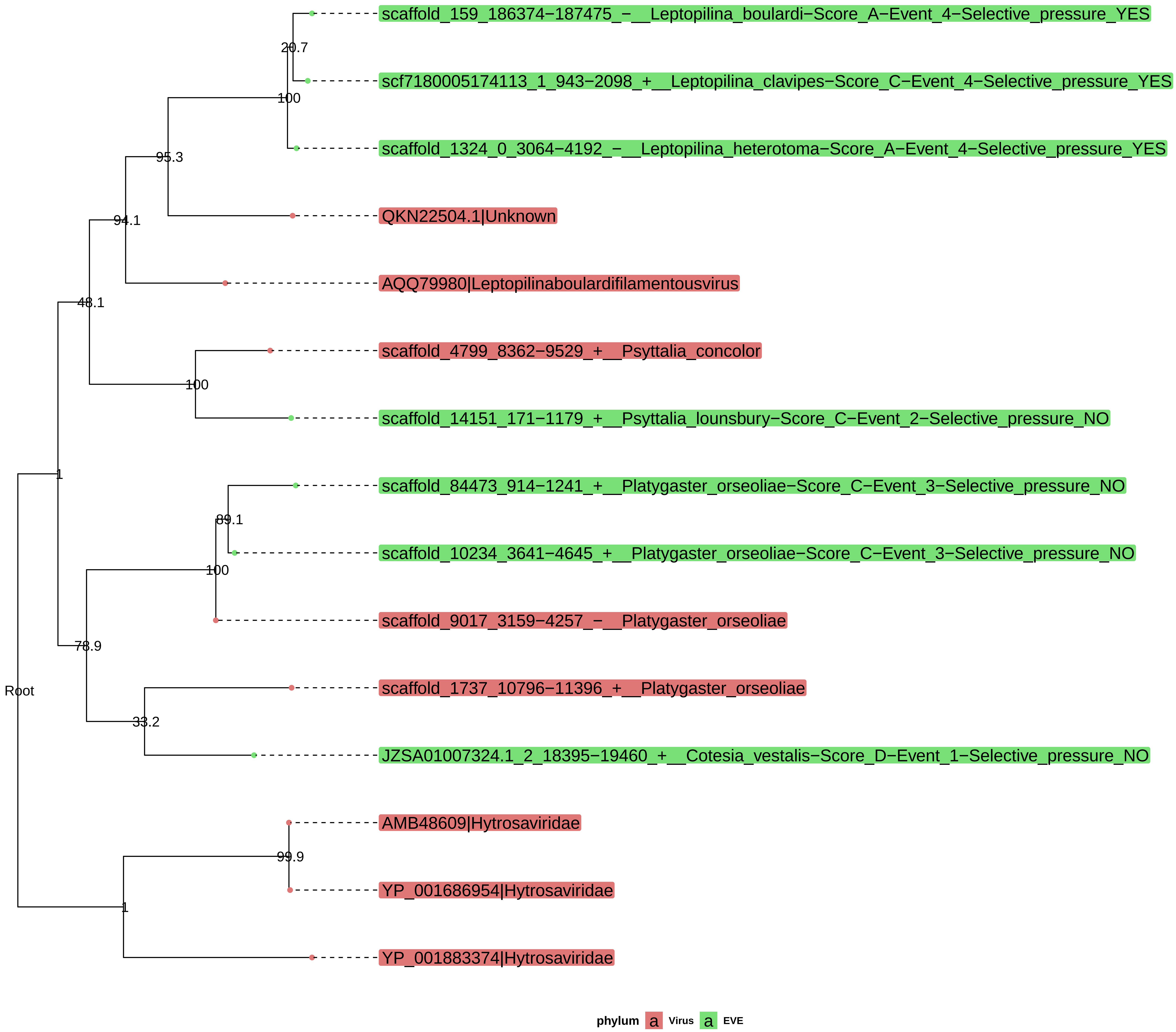

Cluster20217 − families involved : ['LbFV_like', 'Nudiviridae', 'Apis_filamentous−like', 'Baculoviridae'] − putative protein : ['p74']

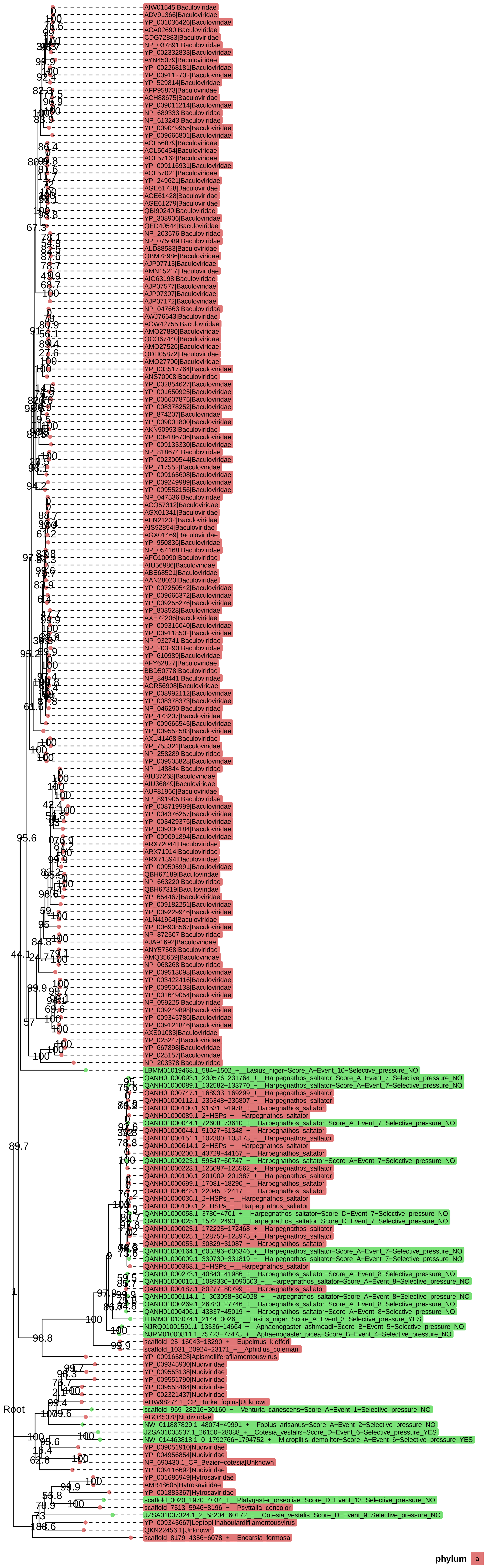

Cluster20069 − families involved : ['LbFV_like'] − putative protein : ['RNA polymerase Rpb1, domain 2']

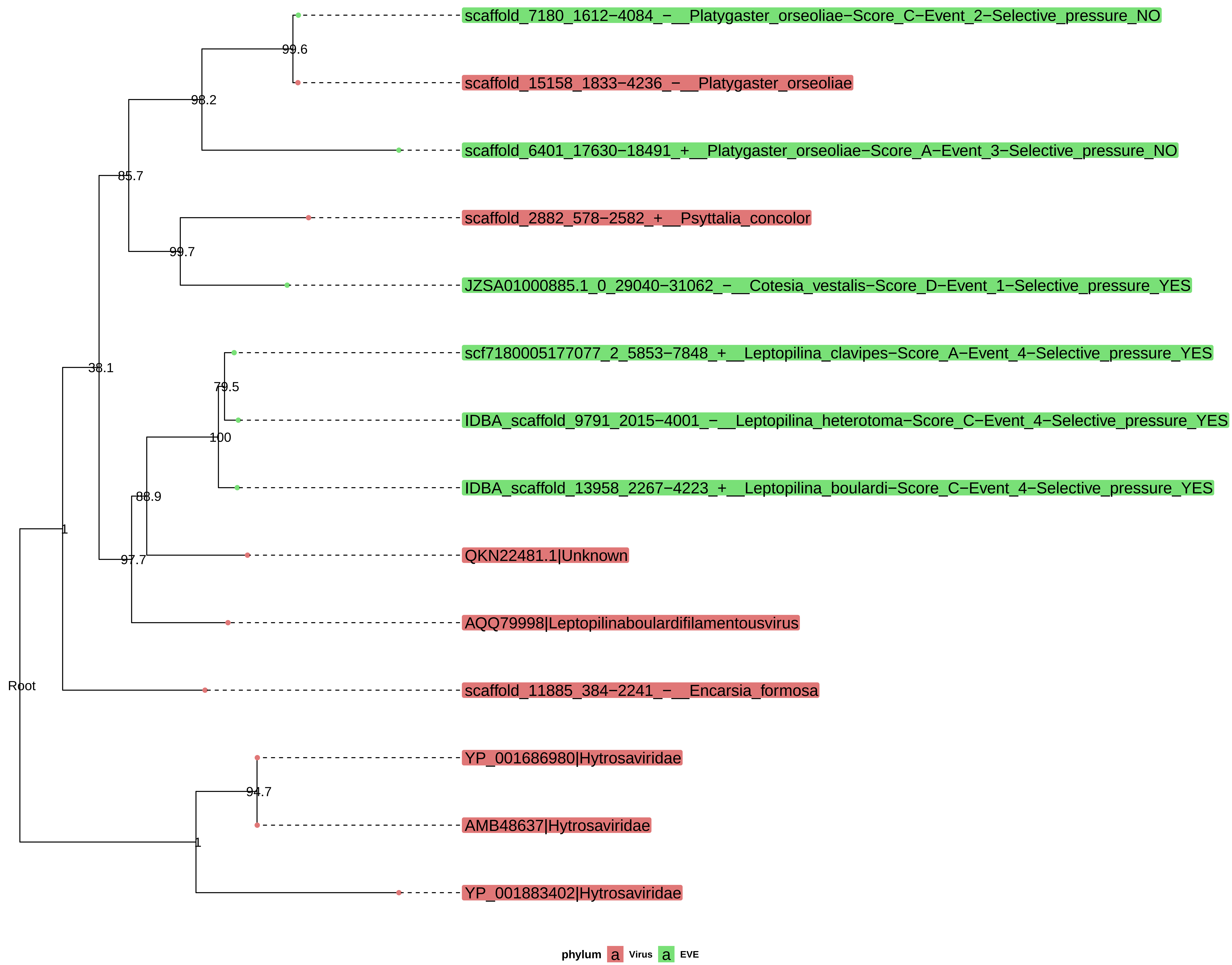

Cluster17683 − families involved : ['Baculoviridae'] − putative protein : ['Golgin subfamily A member 7/ERF4 family']

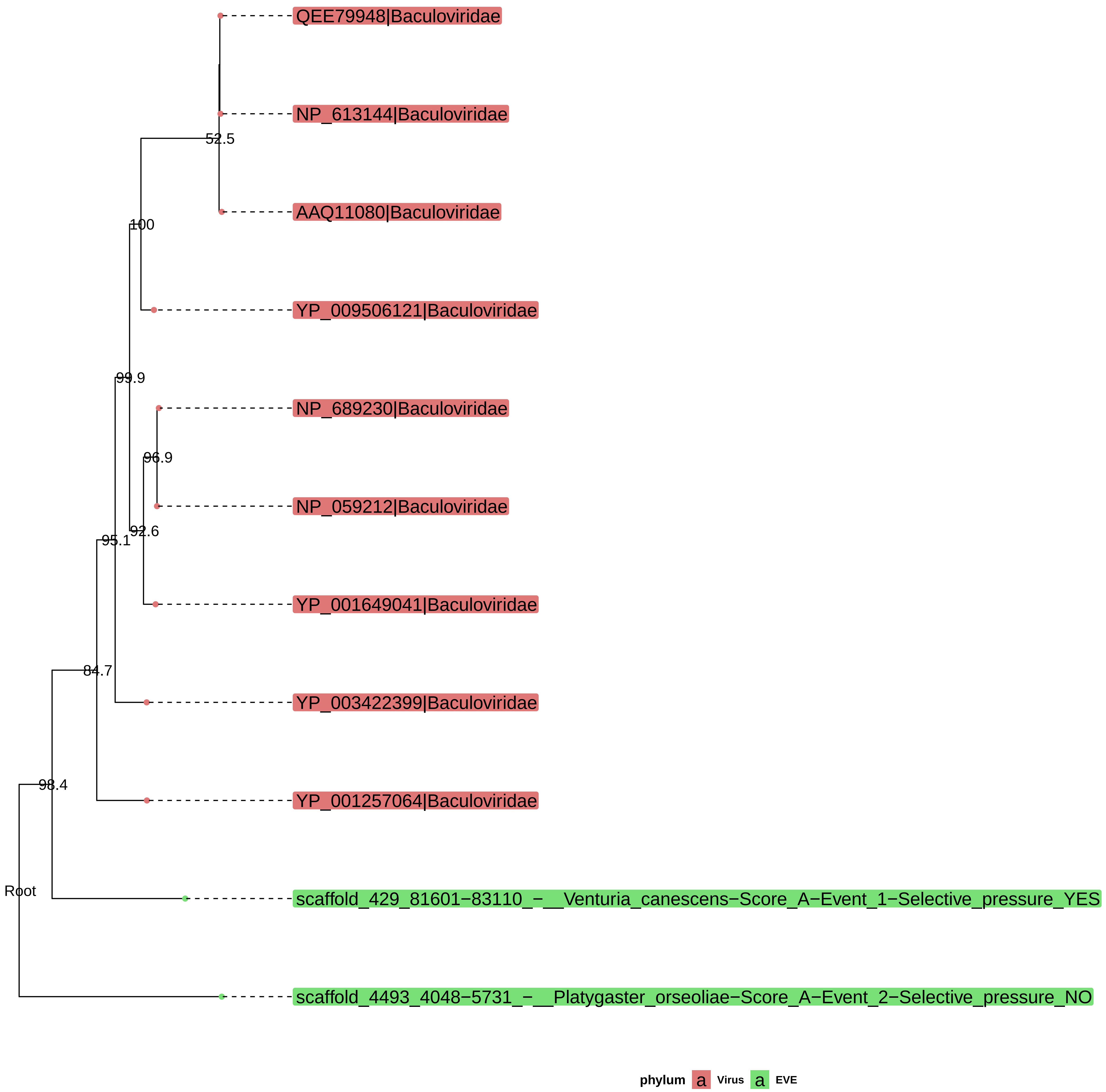

Cluster17071 − families involved : ['LbFV_like'] − putative protein : ['Cwf15/Cwc15 cell cycle control protein']

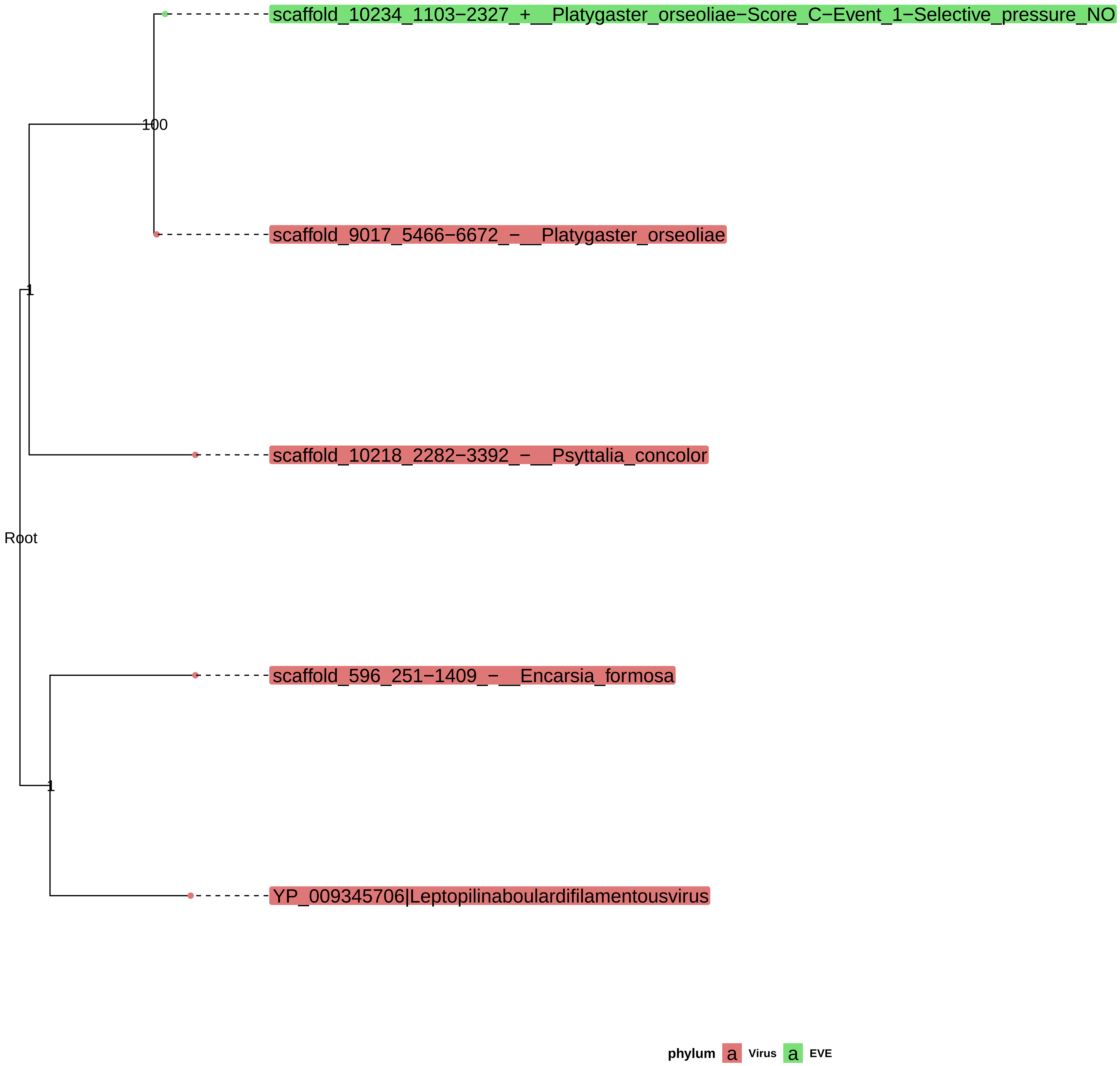

Cluster15514 − families involved : ['LbFV_like'] − putative protein : ['ATPase family associated with various cellular activities (AAA)']

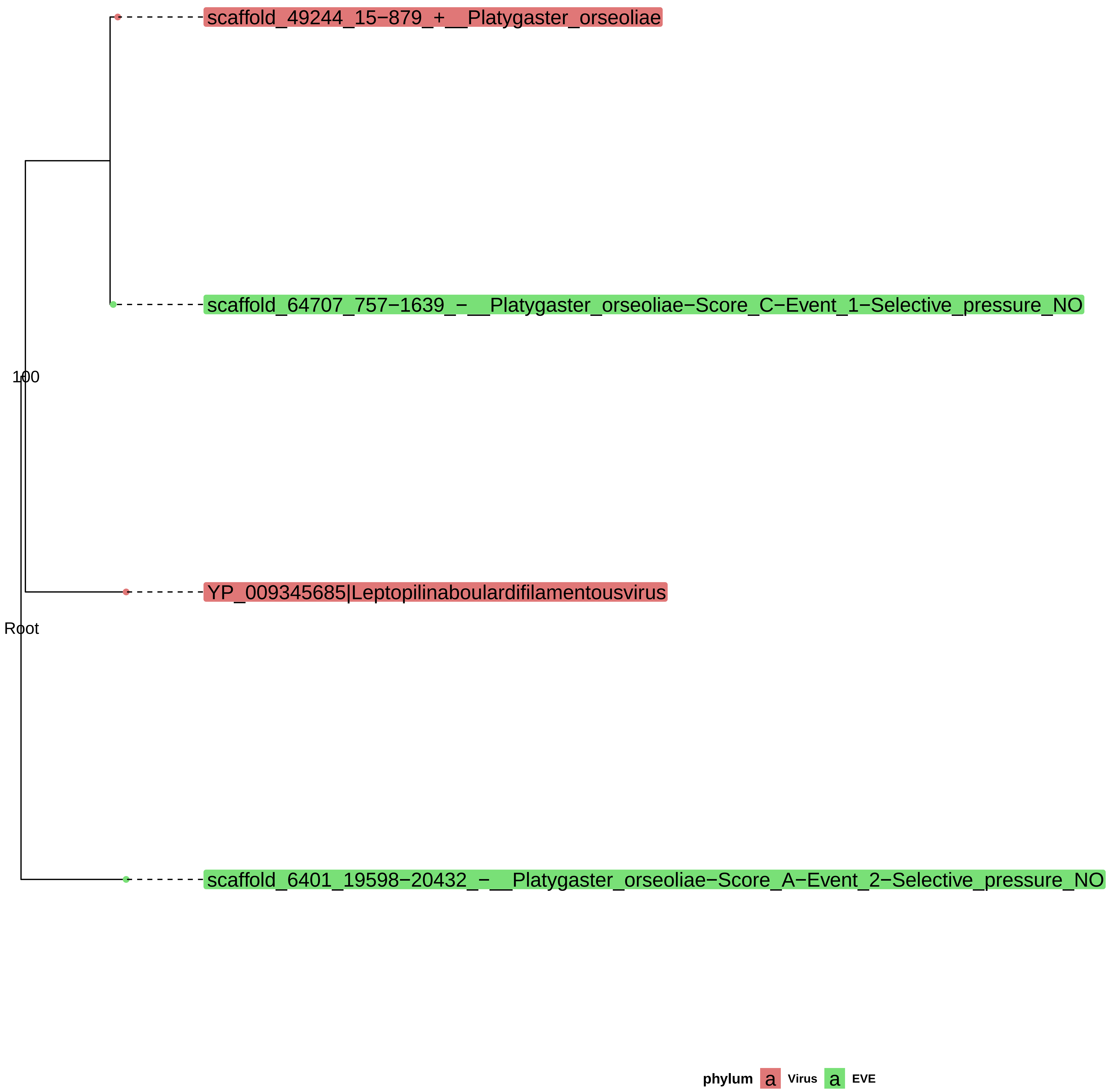

Cluster12545 − families involved : ['LbFV_like'] − putative protein : ['Nudix hydrolase domain−containing protein']

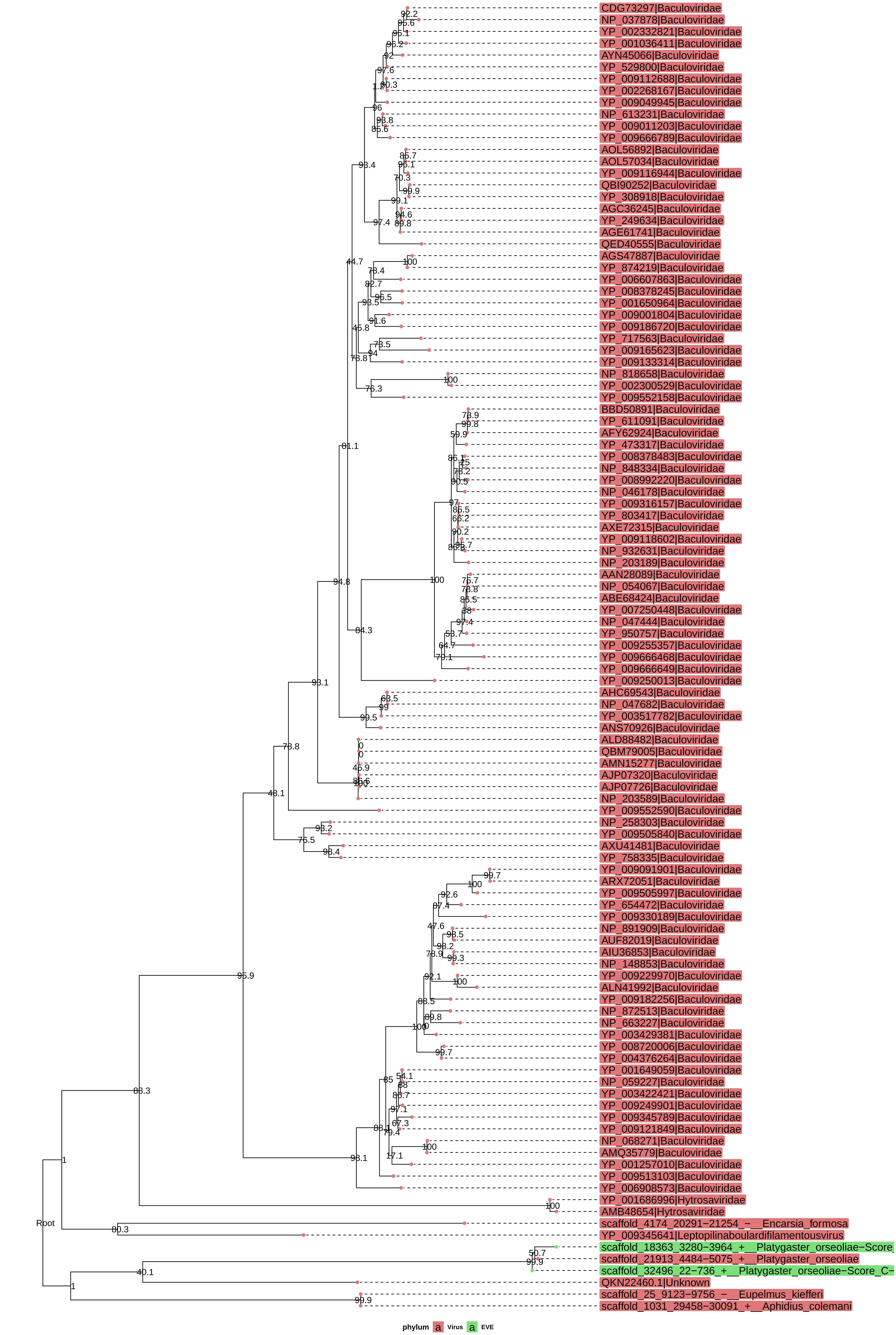

Cluster12537 − families involved : ['LbFV_like'] − putative protein : ['Neurogenesis glycoprotein']

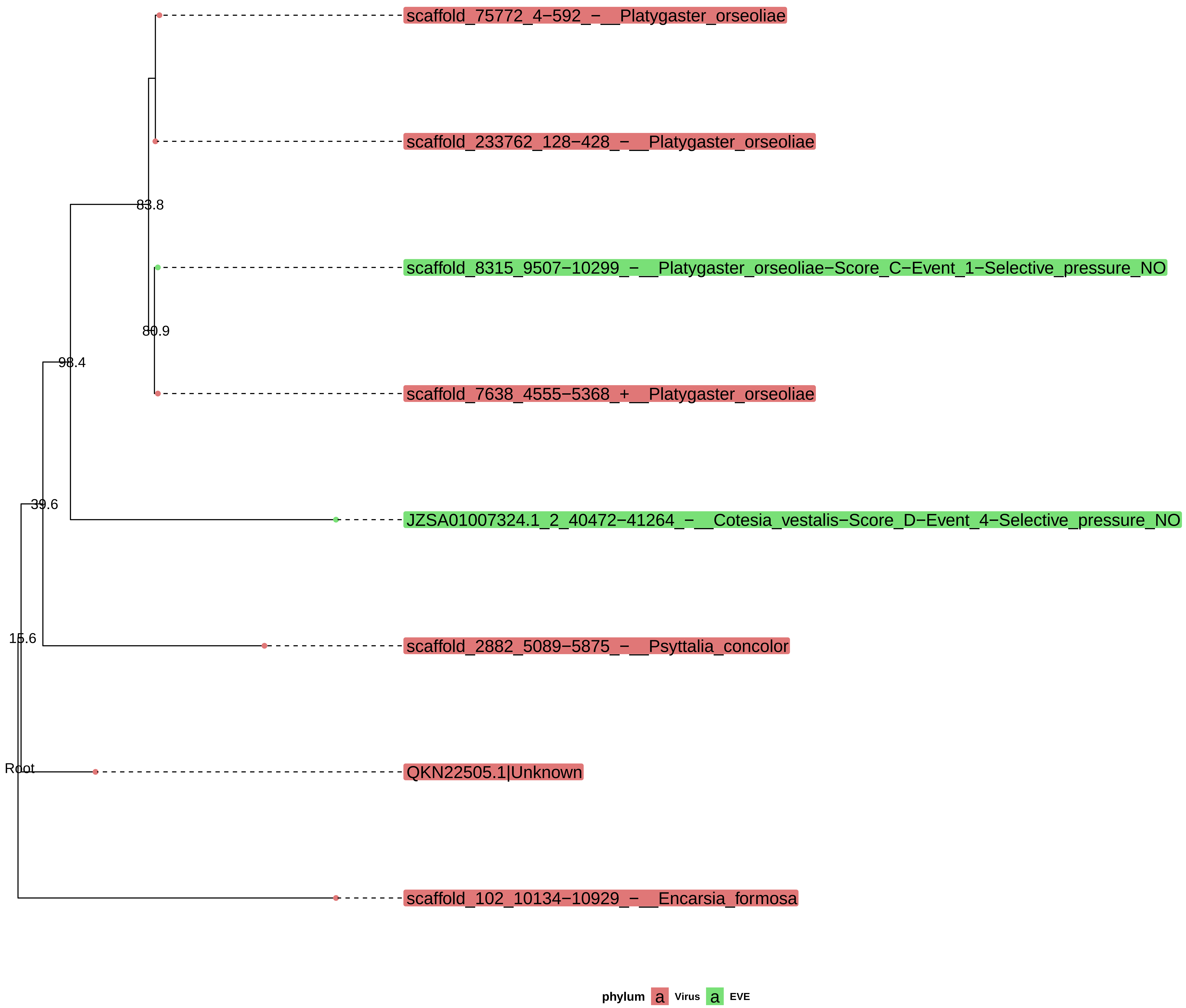

Cluster1043 − families involved : ['LbFV_like'] − putative protein : ['Early growth response N−terminal domain']

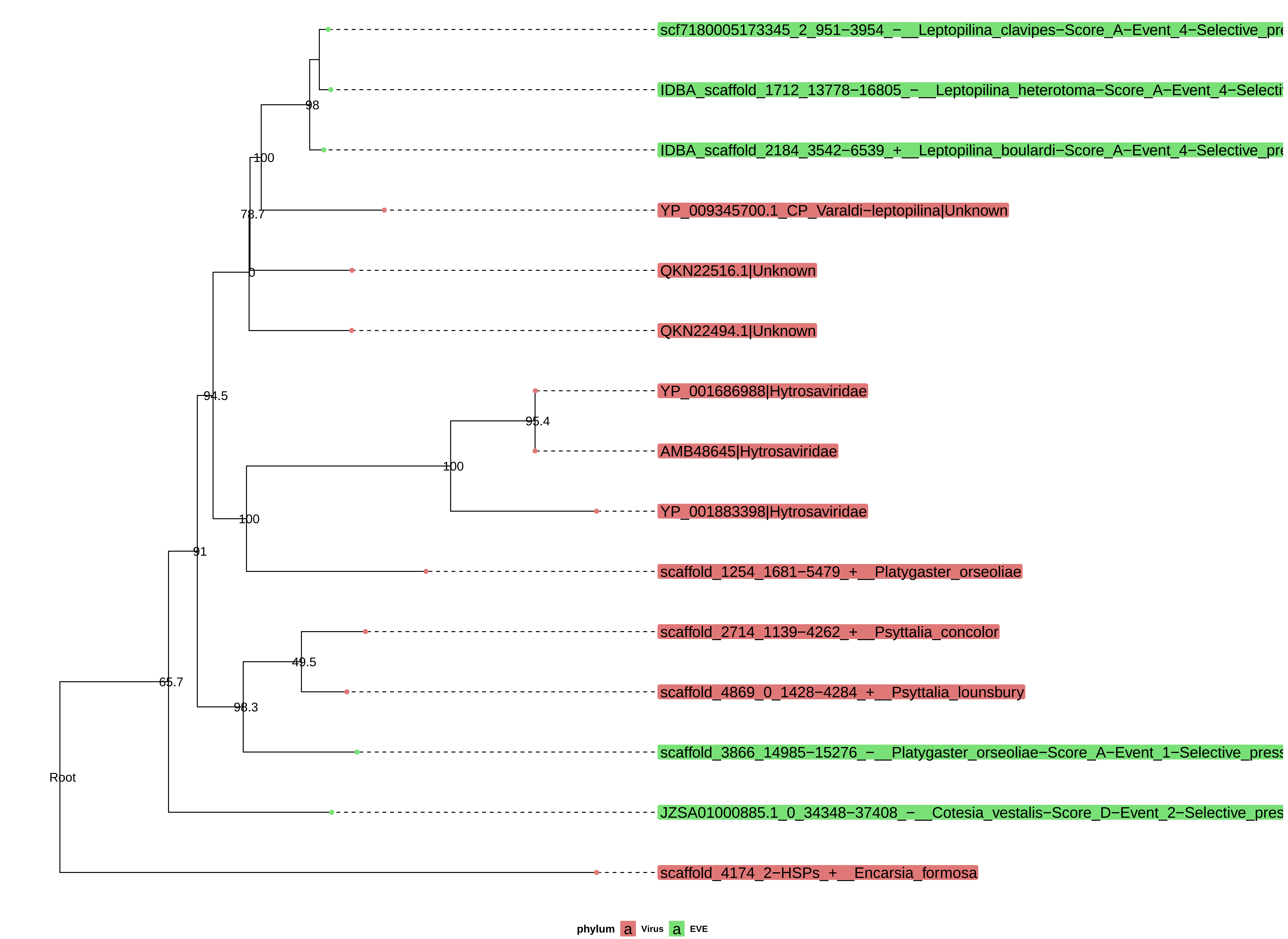

Cluster10416 − families involved : ['LbFV_like', 'Nudiviridae'] − putative protein : ['38K']

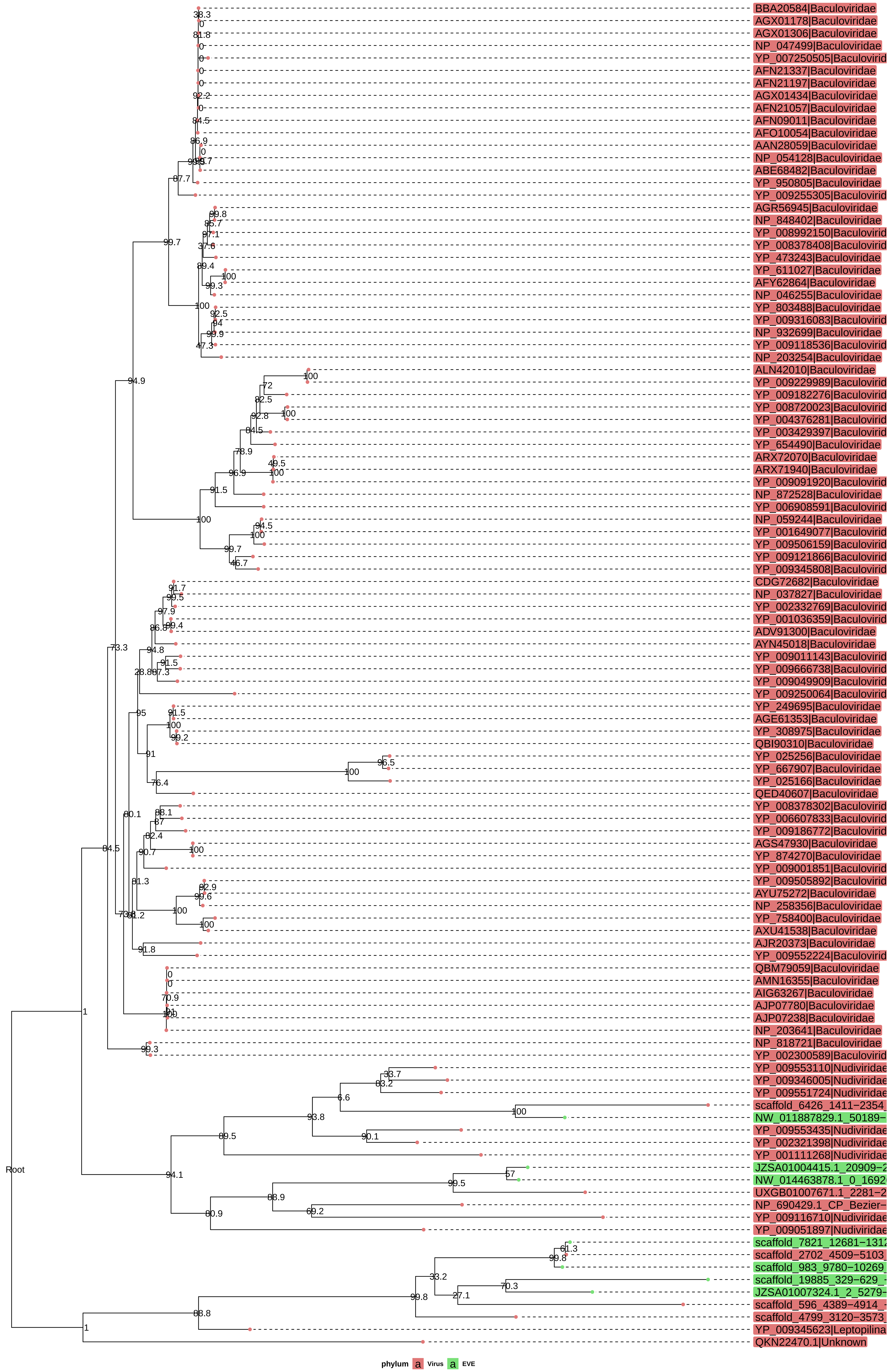

Cluster10025 − families involved : ['Baculoviridae'] − putative protein : ['Uncharacterized protein']

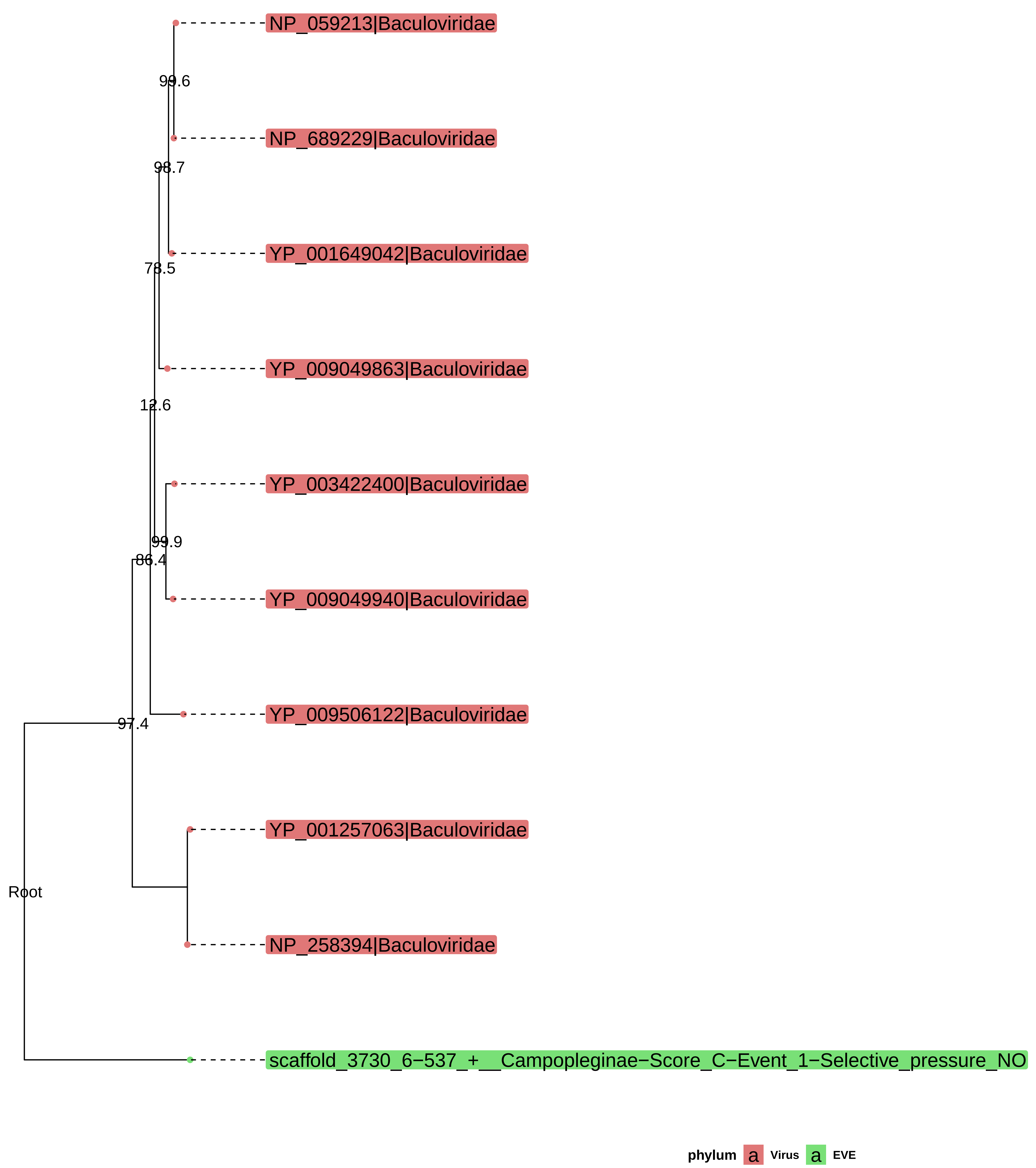

Cluster4844 − families involved : ['Baculoviridae', 'Poxviridae'] − putative protein : ['p94']

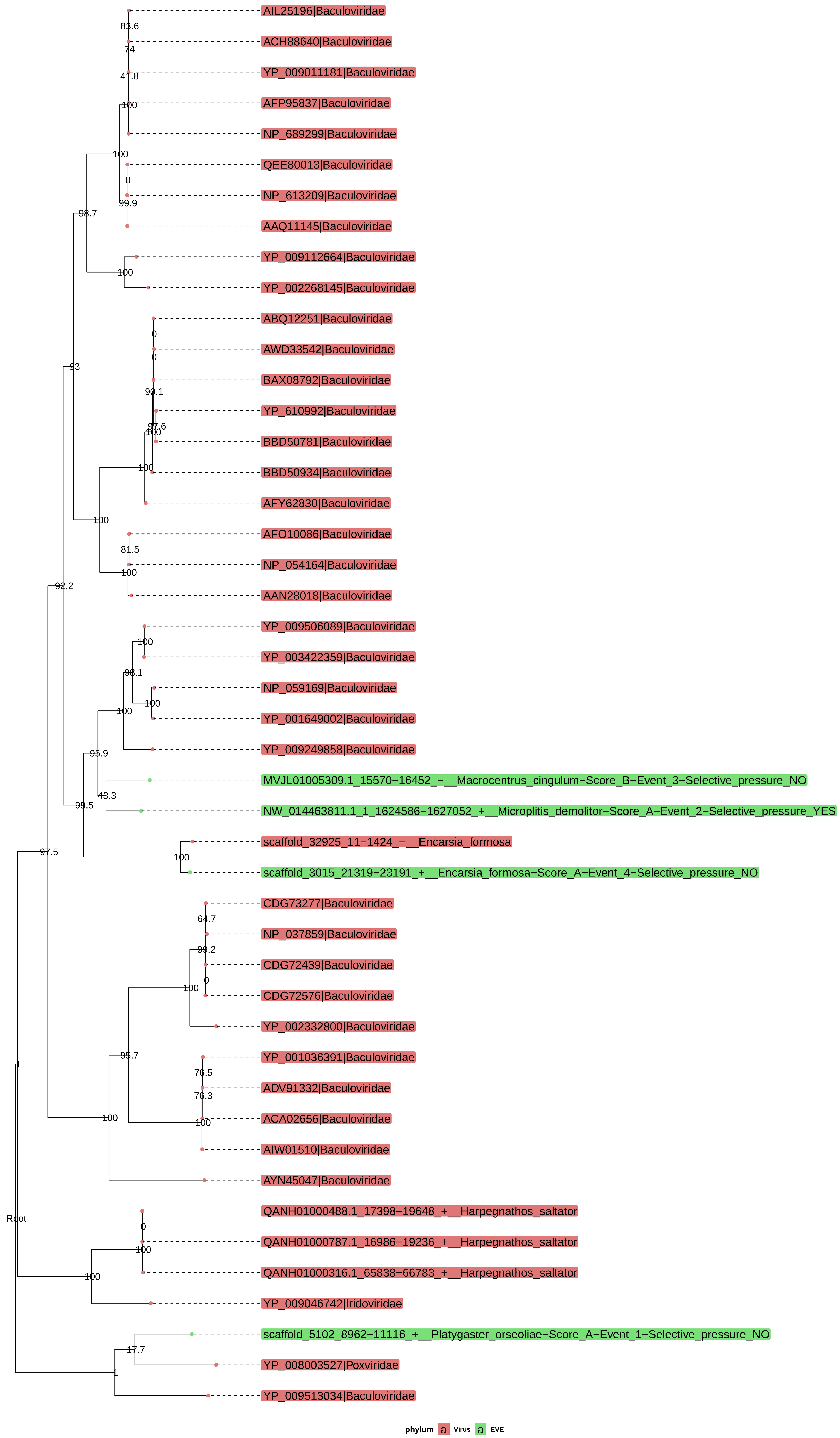

Cluster10412 − families involved : ['Baculoviridae'] − putative protein : ['Chitin−binding type−2 domain−containing protein']

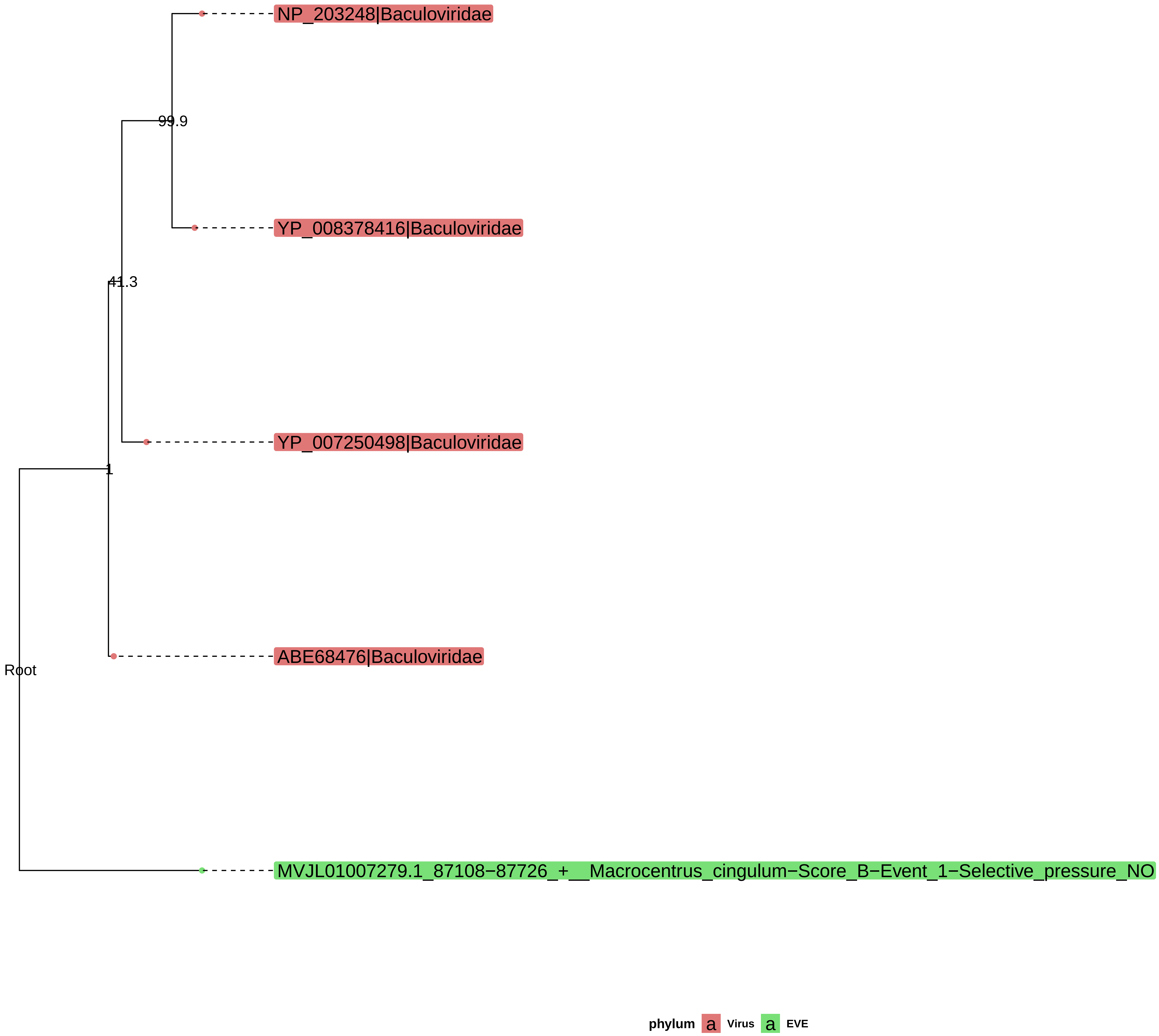

Cluster915 − families involved : ['Baculoviridae'] − putative protein : ['Major budded virus envelope glycoprotein']

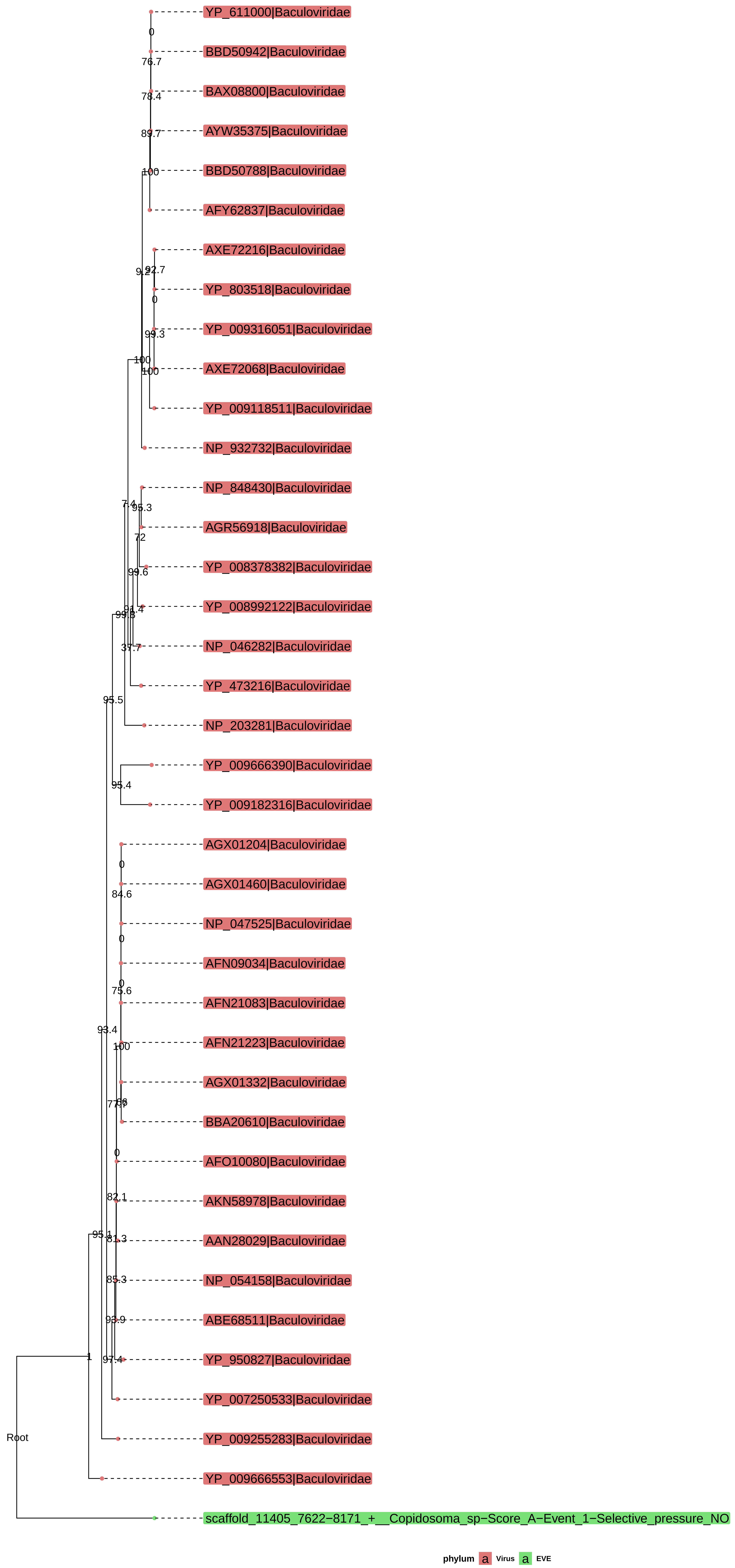

Supplement: Supplementary file 12. — Leafs highlighted in green represent endogenous viral elements (EVEs) (scored from A to D), while leafs highlighted in red represent free-living viruses or loci annotated as putative free-living sequences (scored from D to X). The letter at the end of the taxon label represents the endogenization score assigned to the candidate. The assigned viral family of the free-living genes appears next to the pipe. The numbers right next to the ‘Event’ refers to the assigned Event number. If EVEs were found under selection (either by RNAseq or dN/dS analysis), the end of the leaf name will be ‘Selective_pressure_YES,’ while if not, the name will be ‘Selective_pressure_NO.’ Ultra-Fast Bootstrap values can be found next to the nodes of the phylogenies. For each phylogeny, the putative consensus protein name as well as the putative viral family is given at the top of the figure. [file elife-85993-supp12.pdf]
